# Association analysis of transcriptome and quasi-targeted metabolomics reveals the regulation mechanism underlying broiler muscle tissue development at different levels of dietary guanidinoacetic acid

**DOI:** 10.3389/fvets.2024.1384028

**Published:** 2024-04-25

**Authors:** Jieyun Hong, Sayed Haidar Abbas Raza, Mengqian Liu, Mengyuan Li, Jinrui Ruan, Junjing Jia, Changrong Ge, Weina Cao

**Affiliations:** ^1^College of Animal Science and Technology, Yunnan Agricultural University, Kunming, China; ^2^Guangdong Provincial Key Laboratory of Food Quality and Safety/Nation-Local Joint Engineering Research Center for Machining and Safety of Livestock and Poultry Products, South China Agricultural University, Guangzhou, China; ^3^Yunnan Provincial Key Laboratory of Animal Nutrition and Feed, Yunnan Agricultural University, Kunming, China

**Keywords:** guanidinoacetic acid, broiler, myofiber, development, transcriptome, quasi-targeted metabolomics, meat quality

## Abstract

The development and characteristics of muscle fibers in broilers are critical determinants that influence their growth performance, as well as serve as essential prerequisites for the production of high-quality chicken meat. Guanidinoacetic acid (GAA) is a crucial endogenous substance in animal creatine synthesis, and its utilization as a feed additive has been demonstrated the capabilities to enhance animal performance, optimize muscle yield, and augment carcass quality. The objective of this study was to investigate the regulation and molecular mechanism underlying muscle development in broilers at different levels of GAA via multiple omics analysis. The 90 Cobb broilers, aged 1 day, were randomly allocated into three treatments consisting of five replicates of six chickens each. The control group was provided with a basal diet, while the Normal GAA and High GAA groups received a basal diet supplemented with 1.2 g/kg and 3.6 g/kg of GAA, respectively. After a feeding period of 42 days, the pectoralis muscles were collected for histomorphological observation, transcriptome and metabolomic analysis. The results demonstrated that the addition of 1.2 g/kg GAA in the diet led to an augmentation in muscle fiber diameter and up-regulation of IGF1, IHH, ASB2, and ANKRD2 gene expression. However, a high dose of 3.6 g/kg GAA in the diet potentially reversed the beneficial effects on chicken breast development by excessively activating the TGF-β signaling pathway and reducing nucleotide metabolite content. These findings would provide a theoretical foundation for enhancing the performance and meat quality of broilers by incorporating GAA as a feed additive.

## 1 Introduction

Enhanced sensory attributes and the production of premium meat have emerged as pivotal concerns in the contemporary meat and poultry industry. The myofiber serves as the fundamental functional unit of skeletal muscle, the development and properties of muscle fibers in broilers plays a crucial role in influencing growth performance and ensuring high-quality chicken production (1, 2).

Guanidinoacetic acid (GAA) is an amino acid that undergoes partial synthesis within animal systems, and serves as the primary endogenous substrate for creatine biosynthesis in both humans and animals. Importantly, creatine is also recognized as a conditionally essential nutrient, can be obtained through dietary intake or synthesized endogenously by organisms (3, 4). Furthermore, GAA exerts a positive influence on hormone secretion, substance metabolism, regulation of glycolysis, promotion of muscle fiber development, and modulation of the oxidation-antioxidant state and other physiological functions within the body (5). Currently, GAA is utilized as a feed additive in animal production to partially alleviate the decline in animal performance resulting from limited availability of animal protein raw materials, thereby enhancing muscle yield and carcass quality (6–8).

Currently, there is an escalating dearth of raw materials for crude protein feed, while with the continuous advancement of production technology, the cost associated with industrial synthesis of GAA is diminishing. Although previous studies have consistently demonstrated that the administration of GAA within a dosage range of 0.6–1.2 g/kg can significantly augment the brisket yield of broilers (9–11), but in actual production, there is a possibility that farmers may indiscriminately reduce the inclusion of protein feed by increasing the usage of GAA in order to achieve greater economic benefits, akin to the common practice of excessive addition of mineral supplements in animal feed. However, limited attention has been devoted to investigating the effects of high doses of GAA, particularly in relation to feed conversion rate (12). Furthermore, the recommended dosage of GAA is ranging from 0.6 to 1.2 g/kg, and primarily derived from empirical evidence on livestock performance; whereas, few studies have investigated the regulation of different GAA levels on meat quality traits and its subsequent influence on the economic efficiency of broiler breeding.

In this study, the regulations of normal dose and high-dose GAA on muscle fiber development and meat quality in broilers were investigated and compared from both gene expression and metabolite composition perspectives, and the molecular mechanisms underlying GAA’s regulation of muscle development and meat quality were elucidated. Therefore, our research aims to establish a theoretical basis for identifying key functional genes, signaling pathways, and metabolic substances related to poultry muscle development; provide new insights into the regulatory mechanism of GAA on muscle development and meat quality in broilers; as well as offer a new theoretical foundation for using GAA as a feed additive to improve performance.

## 2 Materials and methods

### 2.1 Animals and experimental design

A total of 90 healthy Cobb broilers (1-day-old) were purchased from a commercial hatchery (Shuncheng Co., Ltd., Hunan, China). With an average initial body weight, the broilers were randomly allocated into three treatments consisting of five replicates of six chickens each. The control group was provided with a basal diet, while the Normal GAA and High GAA groups received a basal diet supplemented with 1.2 g/kg and 3.6 g/kg of GAA, respectively. The sources of the GAA additive were obtained from Panheng Technology Co., Ltd. (Hebei, China), the basal diet was formulated in accordance with the NY/T 33-2004 Chicken Feeding Standard (13) and the NRC (1994) feeding standard (14). During a feeding period of 42 days, all broilers were provided with unrestricted access to feed and water in a temperature-controlled chamber maintained at 22°C, the composition and nutritional profile of the basal diet are presented in Tables 1, 2. Subsequently, the broilers were euthanized for the collection of breast muscle tissue samples, which were stored at −80°C until processing.

**Table 1:** Composition of the basal diet (%).

Ingredients	Brooding period	Breeding period
Corn	61.20	60.90
Soybean meal	30.16	25.22
Fish meal	3.60	0.00
Wheat bran	0.00	10.00
Soybean oil	1.10	0.00
CaHPO_4_	1.50	1.50
Limestone	0.70	0.60
Zeolite powder	0.41	0.46
*L*-Met	0.08	0.07
NaCl	0.25	0.25
Premix	1.00	1.00

The premix for each kilogram of the diet provides the following nutrients: vitamin A (VA) 15,000 U, VD3 3,300 U, VE 62.5 mg, VK 3.6 mg, VB1 3.0 mg, VB2 9.0 mg, VB6 6.0 mg, VB12 0.03 mg, folic acid 60 mg, niacin 60 mg, pantothenic acid 18 mg, biotin 0.36 mg, choline chloride 600 mg, Se 0.15 mg, I 0.35 mg, Cu 12 mg, Mn 60 mg, Fe 80 mg, Zn 75 mg, and other additives such as antibacterial growth promoting agents and antioxidants.

**Table 2:** Nutrient profile of the basal diet (%).

Nutrient components	Brooding period	Breeding period
Metabolizable energy/(MJ/kg)	12.13	11.63
Crude protein (%)	19.30	15.50
Lysine (%)	0.98	0.79
Methionine (%)	0.08	0.07
Calcium (%)	0.85	0.80
Available phosphorus (%)	0.37	0.37

The metabolizable energy was calculated, while the remaining portion was quantified.

### 2.2 Transcriptome sequencing (RNA-seq) analysis

The broiler breast muscle tissues were subjected to RNA extraction using RNAiso Reagent (Takara, China). Subsequently, the samples underwent mRNA sequencing at Novogene technology company (Beijing, China), and resulting in over 8 GB of high-quality data per sample. The integrity of RNA was evaluated utilizing the Bioanalyzer 2100 system (Agilent Technologies, CA, United States) with the RNA Nano 6000 Assay Kit.

The RNA samples were prepared using total RNA as the input material, library fragments were then purified utilizing the AMPure XP system (Beckman Coulter, Beverly, United States). After purification, polymerase chain reaction (PCR) was performed employing Phusion High-Fidelity deoxyriboNucleic acid (DNA) polymerase in conjunction with Universal PCR primers and Index (X) Primer. Subsequently, PCR products were purified using the AMPure XP system and library quality assessment was performed on the Agilent Bioanalyzer 2100 system. Finally, initial processing of raw data in fastq format was performed using custom Perl scripts, and subsequent analyses were exclusively conducted on the high-quality clean data.

The reference genome index was constructed using Hisat2 v2.0.5, and the paired-end clean reads were aligned to it with the same software version. Gene expression levels were quantified by Feature Counts v1.5.0-p3 based on mapped read counts for each gene locus. Differential expression analysis between two conditions was performed using the edgeR R package (version 3.22.5). The *p*-values were adjusted using the Benjamini & Hochberg method, significantly differential expression was defined based on a corrected *p*-value threshold of 0.05 and an absolute fold change greater than or equal to 2-fold. Differentially expressed genes (DEGs) were analyzed using the clusterProfiler R package, which incorporates gene length bias correction, to perform Gene Ontology (GO) enrichment analysis. GO terms with a corrected *p*-value below 0.05 were deemed significantly enriched by the DEGs. Kyoto Encyclopedia of Genes and Genomes (KEGG) serves as a comprehensive database resource facilitating the comprehension of high-level functionalities and utilities within biological systems, encompassing cells, organisms, and ecosystems. It primarily focuses on molecular-level information analysis, particularly large-scale molecular datasets generated through genome sequencing and other advanced experimental technologies.^1^ To evaluate the statistical enrichment of DEGs in KEGG pathways, we utilized the clusterProfiler R package.

### 2.3 Quasi-targeted metabolomics analysis

The broiler breast muscle tissues were prepared following the same procedures as described in a previous study (15). LC-MS/MS analyses were conducted using an ExionLC^™^ AD system (SCIEX) coupled with a QTRAP^®^ 6500+ mass spectrometer (SCIEX) at Novogene Co., Ltd. (Beijing, China). The samples were injected onto an Xselect HSS T3 column (2.1 × 150 mm, 2.5 μm) using a linear gradient over 20 min at a flow rate of 0.4 mL/min for the positive/negative polarity mode. The detection of the experimental sample was performed using multiple reaction monitoring based on the in-house database developed by Novogene. Genes exhibiting a false discovery rate (FDR) value of <0.05 were considered as DEGs.

Subsequently, DEGs underwent GO and KEGG enrichment analysis, the enrichment of terms with a *p*-value <0.05 was considered statistically significant. The data files obtained from HPLC-MS/MS analysis were processed using SCIEX OS Version 1.4 for peak integration and correction, where the area of each peak represented the relative content of the corresponding substance. Metabolites were annotated using the KEGG database (see text foot note 1) and Human Metabolome Database (HMDB) database.^2^ Univariate analysis (*t*-test) was employed to determine statistical significance by calculating *p*-values. Significantly differential metabolites (SDMs) were defined as those exhibiting VIP >1, *p*-value <0.05, and fold change ≥2 or FC ≤0.5. Volcano plots generated by ggplot2 in R language were utilized to filter out metabolites of interest based on Log2(FC) and −log10(*p*-value).

### 2.4 Association analysis of transcriptome and quasi-targeted metabolomics

The method of multiple omics analysis as described previously (16). The pairwise Pearson’s correlation coefficients were computed to assess the association between DEGs and SDMs. The resulting correlation coefficients were visualized using the R package “complex heat map” through heatmap plots.

### 2.5 Immunofluorescence

The breast muscle tissue frozen sections from Cobb broilers were incubated in 4% paraformaldehyde for 20 min, followed by permeabilization with 0.1% Triton X-100 for 10 min. Non-specific binding was blocked using 5% BSA for 30 min. Subsequently, the frozen sections or cells were incubated with a primary antibody against IGF1 (Abcam, England) at a temperature of 37°C for a duration of 2 h. Following this, the frozen sections were stained with Cy3-conjugated (or FITC-conjugated) Donkey Anti-Rabbit IgG (Sangon Biotech, China) at a dilution of 1:100 in 2% BSA for a period of 30 min. All washes were performed using 1 × PBS.

### 2.6 Real-time quantitative PCR analysis

The breast muscle tissue of Cobb broilers was used for total RNA extraction using the RNA simple Total RNA Kit (DP419, Tiangen, China). The concentration and purity of the extracted RNA were determined by UV spectrophotometry. Subsequently, cDNA synthesis was performed using the PrimeScript^™^ RT Reagent kit with gDNA Eraser (TaKaRa, China). Quantitative PCR (qPCR) analysis was conducted in triplicate wells on a 7,500 Real Time PCR System (Applied Biosystems, United States), employing the SYBR Green PCR Master Mix kit (TaKaRa, China). The target gene sequences Indian hedgehog (IHH), ankyrin repeat and SOCS box containing 2 (ASB2), ankyrin repeat domain 2 (ANKRD2), and the reference gene glyceraldehyde-3-phosphate dehydrogenase (GAPDH) were obtained from the NCBI website and imported into Primer 5.0 software for primer design. Specific primers were designed accordingly as listed in Table 3. Gene expression analysis was carried out using the 2^−ΔΔCT^ method (17).

**Table 3:** Specific primers for *IHH*, *ASB2*, *ANKRD2* and *GAPDH* in the qPCR experiments.

Gene/Accession No.	Primer sequences (5′ to 3′)	Product length (bp)
*IHH*/NM_204957.3	F: GTCAGACAGGGACCGCAACAAG	125 bp
	R: CAGCCGAGTGCTCTGACTTGACG	
*ASB2*/XM_040672449.2	F: CGGCGGCAAACACCCAACGACA	231 bp
	R: GCCAAGGGTAGAGGTGGTAGGG	
*ANKRD2*/MG870384.1	F: GAACCTGGCTGGTAAGACTCCG	108 bp
	R: GCTGGGACCTCAGTCTCCTCTTG	
*GAPDH*/NM_204305.2	F: GCCCAGAACATCATCCCAGCGT	137 bp
	R: CGGCAGGTCAGGTCAACAACAG	

### 2.7 Statistical analysis

The data were presented as mean ± standard deviation (mean ± SD). Statistical analysis was performed using SAS v8.0 (SAS Institute, Cary, NC). Statistical significance was determined by one-way analysis of variance (ANOVA) test. A significance level of *p* < 0.05 was considered statistically significant (“*” denotes *p* < 0.05). Graphs were generated using GraphPad Prism 6 (GraphPad Software Incorporated, California, United States).

## 3 Results

### 3.1 The impact of different levels of GAA on the development of muscle fiber in broilers

**Figure 1:**
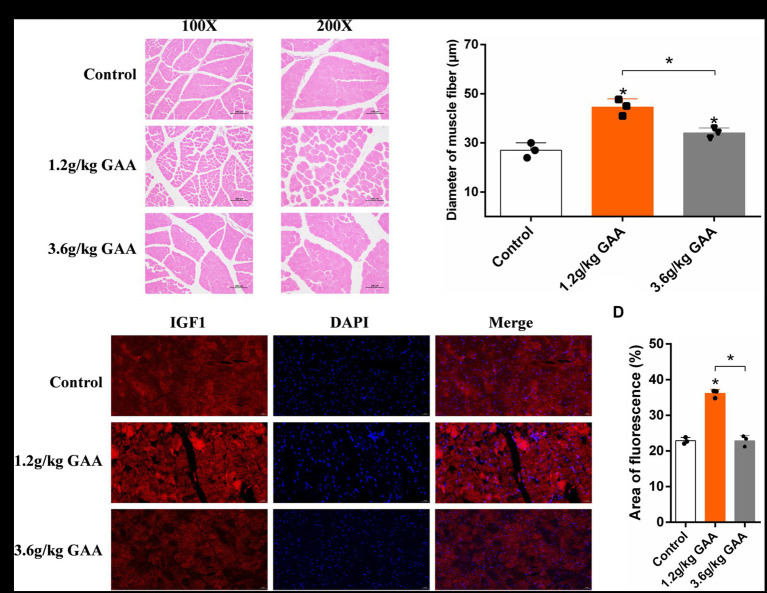
The impact of different levels of GAA on the development of muscle fiber in broilers. **(A)** HE-stained cross-section of broiler breast muscle tissue. **(B)** Diameter of muscle fibers in the breast muscle tissue of broilers. **(C)** The protein expression levels of IGF1 in breast muscle tissue of broilers were assessed using immunofluorescence staining. **(D)** Immunofluorescent detection of IGF1 protein expression levels in the breast muscle tissue of broilers. Data are expressed as mean ± standard deviation (mean ± SD), error bars represent the SD, “*” *p* < 0.05 compared with the control group (*n* = 3).

To investigate the impact of different levels of GAA on muscle fiber development in broiler breast muscles, broilers were fed diets containing 0 g/kg (control group), 1.2 g/kg (Normal group), and 3.6 g/kg (High group) of GAA from day 1 onwards, respectively. Subsequently, breast muscle samples from broilers were collected at 42 days of age, followed by histological examination using HE staining and immunofluorescence staining for insulin like growth factor 1 (IGF1). We observed a significant impact of different concentrations of GAA on the muscle fiber diameter in 42-day-old Cobb broilers by HE staining. Compared to the control group, both the 1.2 g/kg GAA group and the 3.6 g/kg GAA group exhibited significantly higher muscle fiber diameters (*p* < 0.05), and the muscle fiber diameter in the 3.6 g/kg GAA group exhibited a significantly reduced magnitude compared to that observed in the 1.2 g/kg GAA group (*p* < 0.05) (Figures 1A,B). Immunofluorescence staining analysis results of chest muscles from different treatment groups revealed that supplementation with 1.2 g/kg GAA significantly upregulated the protein expression of IGF1 in broiler chest muscles compared to the control group (*p* < 0.05), but there was no significant difference between the control group and the group supplemented with 3.6 g/kg GAA (*p* > 0.05) (Figures 1C,D).

### 3.2 RNA-Seq analysis of differentially expressed genes in broiler breast muscle

**Figure 2:**
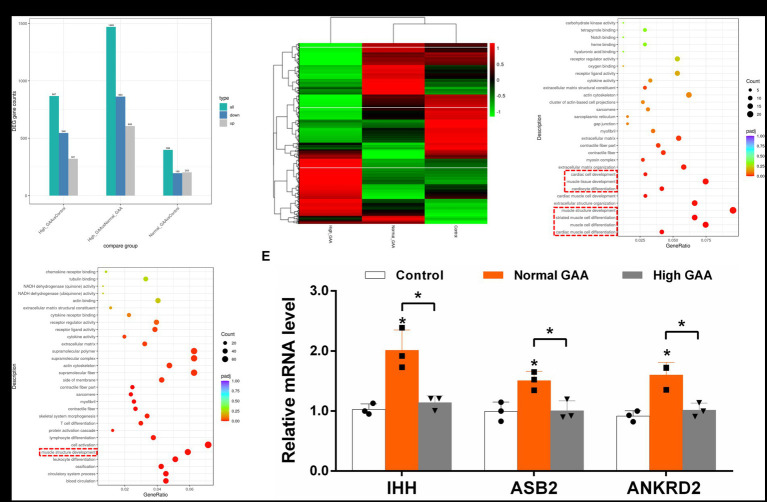
RNA-Seq analysis of DEGs in broiler breast muscle between different experimental groups. The “Control” group represents broilers fed a basal diet, while the “Normal GAA” group represents broilers fed a diet containing 1.2 g/kg of GAA, and the “High GAA” group represents broilers fed a diet containing 3.6 g/kg of GAA. **(A)** The number of DEGs for each comparison combination is presented. The color green represents the total count of DEGs, while blue indicates downregulated genes and grey denotes upregulated genes. **(B)** The clustering map of DEGs was generated, with red indicating genes exhibiting high expression levels and green representing genes displaying low expression levels. The color gradient from red to green corresponds to the logarithmic values of log2 (FPKM+1), ranging from highest to lowest. **(C)** Scatter plot depicting the GO functional enrichment of DEGs between the Normal GAA group and control group in RNA-seq analysis. **(D)** Scatter plot depicting the GO functional enrichment of DEGs between the High GAA group and Normal GAA group in RNA-seq analysis. **(E)** Relative mRNA expression levels of key myogenesis related genes IHH, ASB2, and ANKRD2 in different experimental groups. Data are expressed as mean ± standard deviation (mean ± SD), error bars represent the SD, “*” *p* < 0.05 compared with the control group (*n* = 3).

RNA-Seq analysis was utilized to investigate the systemic mechanisms underlying muscle development in broilers at different levels of GAA. Transcriptomes of breast muscles from broilers treated with 1.2 g/kg GAA (referred to as Normal GAA), 3.6 g/kg GAA (referred to as High GAA), or a control group were compared. Compared to the control group, Normal GAA feeding resulted in a transcriptional signature characterized by the downregulation of 195 genes and upregulation of 203 genes, whereas High GAA feeding led to a transcriptional signature characterized by the downregulation of 546 genes and upregulation of 321 genes. The High GAA group exhibited a distinct transcriptional signature characterized by the downregulation of 863 genes and upregulation of 606 genes, in comparison to the Normal GAA group (Figures 2A,B). The GO analysis revealed a significant enrichment of DEGs associated with muscle tissue development and muscle cell differentiation in the Normal GAA group compared to the control group (Figure 2C). Additionally, comparing the High GAA group to the Normal GAA group demonstrated an enrichment of DEGs involved in muscle structure development (Figure 2D). The most significant differences in the expression of myogenesis-related DEGs within each experimental group were compiled and presented in Table 4. We further quantified the mRNA expression levels of key myogenesis-related genes in the RNA-seq samples using qPCR analysis. The results revealed a significant upregulation of IHH, ASB2, and ANKRD2 in the Normal GAA group compared to both the control group and the High GAA group (Figure 2E; *p* < 0.05).

**Table 4:** The most significant differences in the expression of myogenesis related DEGs in each experimental group.

Gene full name	Symbol	Normal GAA vs. Control (fold change)	High GAA vs. Control (fold change)	High GAA vs. Normal GAA (fold change)
Ankyrin repeat and SOCS box containing 2	ASB2	2.33	NS	−2.68
Salt inducible kinase 1	SIK1	−1.41	NS	0.94
Myosin heavy chain 11	MYH11	1.61	NS	−0.81
Indian hedgehog	IHH	2.61	NS	−2.18
Ankyrin repeat domain 2	ANKRD2	1.43	NS	−1.66
Delta like canonical notch ligand 1	DLL1	0.83	NS	−0.69
Sarcoglycan delta	SGCD	0.76	1.20	0.44

### 3.3 KEGG enrichment analysis of differentially expressed genes

**Figure 3:**
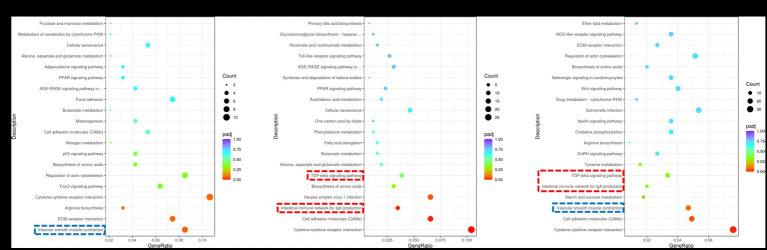
KEGG enrichment scatter plot of DEGs in RNA-seq analysis. The vertical axis represents the names of pathways, while the horizontal axis denotes enriched factors. The size of each data point corresponds to the number of DEGs within that pathway, and the color of points indicates distinct *Q*-value ranges. **(A)** Scatter plot depicting KEGG enrichment of DEGs between the Normal GAA group and control group. **(B)** Scatter plot depicting KEGG enrichment of DEGs between the High GAA group and control group. **(C)** Scatter plot depicting KEGG enrichment of DEGs between the High GAA group and Normal GAA group.

The coordinated regulation of biological processes within the muscle involves the activation of various genes. In this study, we performed pathway enrichment analysis of DEGs using the KEGG database. Our findings revealed significant associations between DEGs and key pathways, including vascular smooth muscle contraction, intestinal immune network for IgA production, TGF-β pathway and others (Figure 3). The expression regulation of core DEGs in the aforementioned pathways have been shown in Supplementary Figures S1–S6. The vascular smooth muscle contraction pathway was identified as the shared pathway between the Normal GAA vs. control group and High GAA vs. Normal group (Figures 3A,C). Meanwhile, both the intestinal immune network for IgA production pathway and TGF-β pathway were found to be common pathways in both the High GAA vs. control group and High GAA vs. Normal GAA group (Figures 3B,C). The findings suggest that administration of 3.6 g/kg GAA may induce an immune response, thereby counteracting myogenesis.

### 3.4 Quasi-targeted metabolomics analysis of significantly differential metabolites in broiler breast muscle

**Figure 4:**
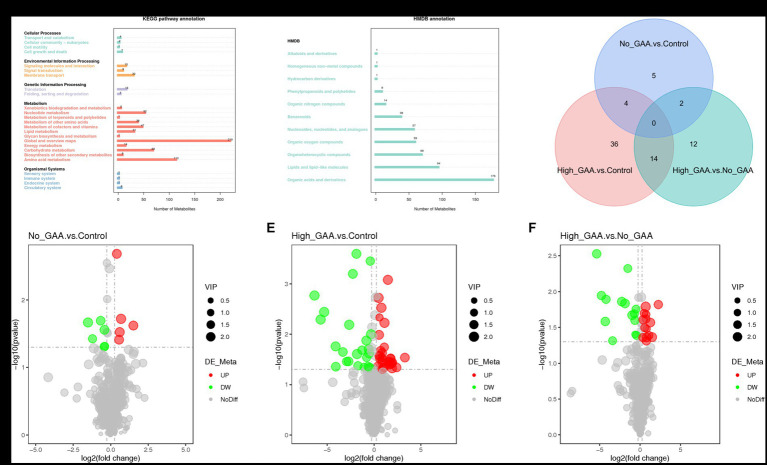
Screening of metabolites by quasi-targeted metabolomics analysis. **(A)** The annotation of 388 metabolites in KEGG database. **(B)** The annotation of 519 metabolites in HMDB database. **(C)** Venn diagram illustrating common metabolites (positive + negative ion modes). **(D)** The volcano map of SDMs comparing the Normal GAA group vs. Control group. **(E)** The volcano map of SDMs comparing the High GAA group vs. Control group. **(F)** The volcano map of SDMs comparing the High GAA group vs. Normal GAA group.

The annotation of 388 metabolites was performed using the KEGG database, revealing a predominant concentration of metabolites in pathways encompassing global and overview maps, amino acid metabolism, carbohydrate metabolism, nucleotide metabolism, cofactors and vitamins metabolism, other amino acids metabolism, as well as lipid metabolism within the category of metabolic processes (Figure 4A). The HMDB database annotated a total of 519 metabolites, predominantly comprising organic acids and derivatives, lipids and lipid-like molecules, organoheterocyclic compounds, organic oxygen compounds, as well as nucleosides, nucleotides, and analogues (Figure 4B). A standard for measuring SDMs was established based on the PLS-DA analysis, wherein variable importance in the projection (VIP) >1 and *p* < 0.05 were defined as criteria. In both positive and negative ion modes, a total of 73 SDMs were identified among the three groups, and the distribution of these SDMs between the groups is presented in Figure 4C. A total of 11 SDMs were identified in the comparison between Normal GAA and Control groups, with 5 exhibiting high abundances and 6 displaying low abundances (Figure 4D). In the High GAA group vs. Control group comparison, a total of 54 SDMs were detected, with 30 exhibiting high abundances and 24 displaying low abundances (Figure 4E). Furthermore, we observed a total of 28 SDMs in the comparison between High GAA and Normal GAA groups, with an equal distribution of 14 SDMs each for high and low abundances (Figure 4F).

**Figure 5:**
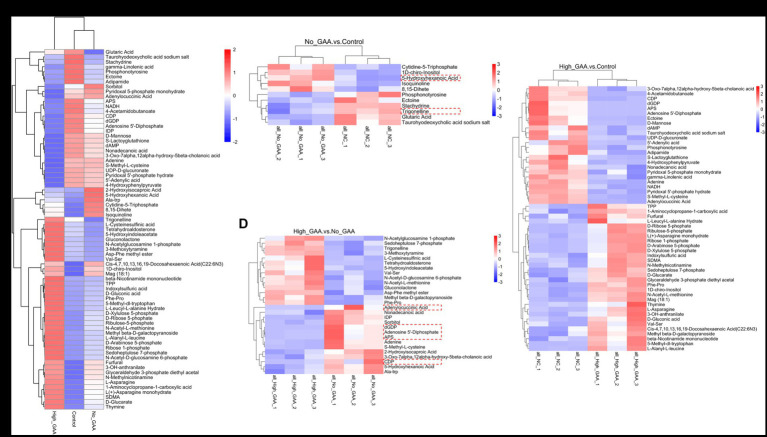
The clustering heatmap of SDMs. **(A)** The clustering heatmap of all SDMs (positive + negative ion modes). **(B)** The clustering heatmap of SDMs comparing the Normal GAA group vs. control group (positive + negative ion modes). **(C)** The clustering heatmap of SDMs comparing the High GAA group vs. control group (positive + negative ion modes). **(D)** The clustering heatmap of SDMs comparing the High GAA group vs. Normal GAA group (positive + negative ion modes).

The cluster analysis diagram in Figure 5A illustrates distinct clustering patterns of SDMs within the Control group, Normal GAA group, and High GAA group, respectively. Broilers in the Normal GAA group exhibited significantly elevated levels of 5-hydroxyhexanoic acid and reduced levels of trigonelline in muscle compared to the other groups (Figure 5B). A total of 14 common SDMs were identified in the High GAA group, with significantly lower abundance observed for the top 5 SDMs, namely deoxyguanosine diphosphate (dGDP), adenosine 5′-diphosphate, adenylocuccinic acid, adenylate kinase (APS), and cytidine diphosphate (CDP), compared to the other groups (Figures 5C,D).

**Figure 6:**
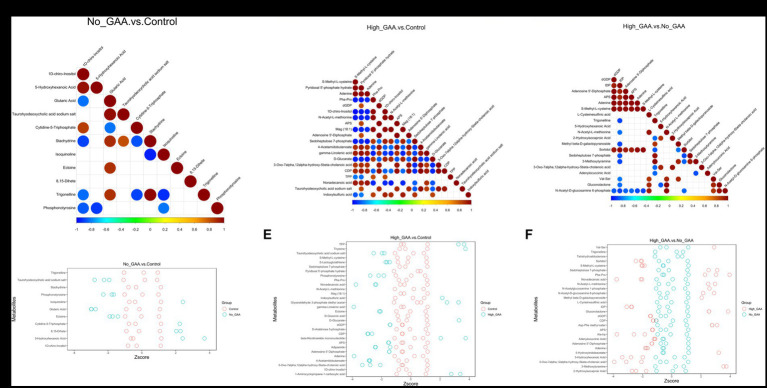
Correlation analysis of partial SDMs. **(A)** The correlation maps of 11 SDMs comparing the Normal GAA group vs. control group (positive + negative ion modes). **(B)** The correlation maps of 20 SDMs comparing the High GAA group vs. control group (positive + negative ion modes). **(C)** The correlation maps of 20 SDMs comparing the High GAA group vs. Normal GAA group (positive + negative ion modes). **(D)** The *Z*-score maps of 11 SDMs comparing the Normal GAA group vs. control group (positive + negative ion modes). **(E)** The *Z*-score maps of 20 SDMs comparing the High GAA group vs. control group (positive + negative ion modes). **(F)** The *Z*-score maps of 20 SDMs comparing the High GAA group vs. Normal GAA group (positive + negative ion modes).

During muscle tissue development and muscle cell differentiation, there was a significant correlation between the expression levels of different metabolites, and the correlation analysis of partial SDMs were presented in Figures 6A–C. Meanwhile, partial top SDMs were selected based on *p*-values to detect relative metabolite content at an equivalent level, with *Z*-score values of SDMs in each sample shown in Figures 6D–F. In addition to 8,15-dihete, the results revealed significant correlations among 10 SDMs comparing the Normal GAA group vs. control group, including 1D-chiro-inositol, 5-hydroxyhexanoic acid, glutaric acid, taurohyodeoxycholic acid sodium salt, cytidine-5-triphosphate, etc. However, no significant correlation was observed between 8,15-dihete and the remaining 10 SDMs, this suggests that 8,15-dihete may have an independent role (Figure 6A). The relative quantitative values of SDMs in the corresponding experimental samples of the Normal GAA group and control group were depicted in Figure 6D. We observed significant correlations among 20 SDMs when comparing the High GAA group with the control group, including S-Methyl-L-cysteine, pyridoxal 5′-phosphate hydrate, adenine, Phe-Pro, dGDP, etc. (Figure 6B). The relative quantitative values of SDMs in the corresponding experimental samples in these two groups were depicted in Figure 6E. The results also revealed significant correlations among 20 SDMs comparing the High GAA group vs. Normal GAA group, encompassing nucleotide metabolites such as dGDP, inosine diphosphate (IDP), adenosine 5′-diphosphate, APS, adenine, etc. (Figure 6C). Furthermore, Figure 6F depicted the relative quantitative values of SDMs in the corresponding experimental samples of High GAA and Normal GAA groups.

**Figure 7:**
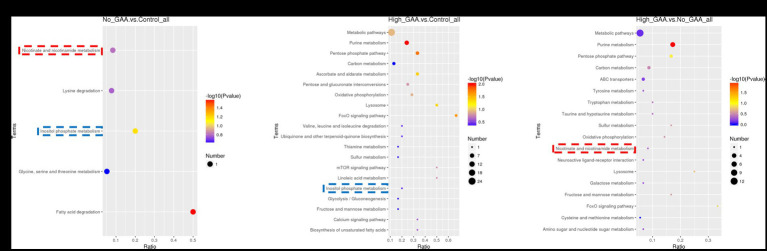
The KEGG enriched bubble pattern of SDMs. **(A)** The KEGG enriched bubble pattern of SDMs comparing the Normal GAA group vs. control group (positive + negative ion modes). **(B)** The KEGG enriched bubble pattern of SDMs comparing the High GAA group vs. control group (positive + negative ion modes). **(C)** The KEGG enriched bubble pattern of SDMs comparing the High GAA group vs. Normal GAA group (positive + negative ion modes).

The bubble pattern depicting the enrichment of SDMs across diverse signaling pathways within the KEGG database. A total of 5, 26, and 18 KEGG pathways were identified, which exhibited significant enrichment of SDMs in the Normal GAA vs. control, High GAA vs. control, and High GAA vs. Normal GAA groups, respectively (Figures 7A–C). We observed a significant association of the inositol phosphate metabolism pathway with both the Normal GAA vs. control and High GAA vs. control groups (Figures 7A,B); while the nicotinate and nicotinamide metabolism pathway was shared by both the Normal GAA vs. control and High GAA vs. Normal GAA groups (Figures 7A,C). The specific enrichment of core SDMs in these two pathways have been shown in Supplementary Figures S7–S10.

### 3.5 Association analysis of transcriptome and quasi-targeted metabolomics

**Figure 8:**
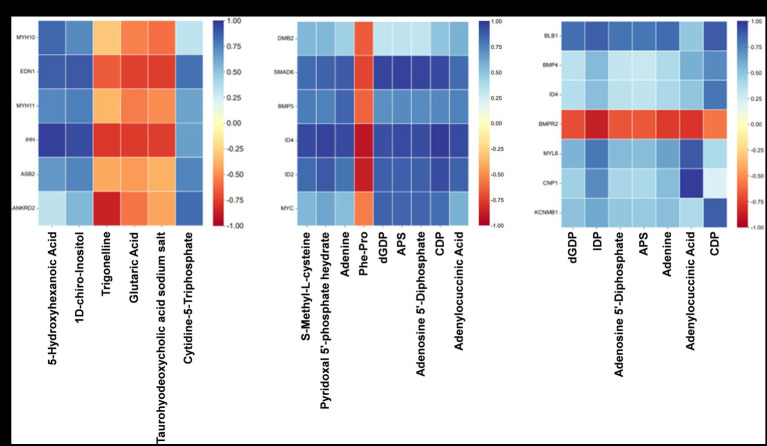
Association analysis of transcriptome and quasi-targeted metabolomics. **(A)** The correlation matrix heat map of partial DEGs and SDMs comparing the Normal GAA group vs. control group (positive + negative ion modes). **(B)** The correlation matrix heat map of partial DEGs and SDMs comparing the High GAA group vs. control group (positive + negative ion modes). **(C)** The correlation matrix heat map of partial DEGs and SDMs comparing the High GAA group vs. Normal GAA group (positive + negative ion modes).

To investigate the relationship between gene expression and metabolite abundance, a subset of DEGs and SDMs associated with vascular smooth muscle contraction, intestinal immune network for IgA production, and TGF-β pathway were selected from the top 100 DEGs and top 50 SDMs based on their ascending *p*-values. Subsequently, a correlation matrix heat map was constructed to visually represent the results of correlation analysis for core DEGs and SDMs (Figures 8A–C).

The findings suggest that normal GAA intake significantly regulates the expression of genes associated with muscle development compared to the control group. Notably, there were significant positive correlations observed between the expressions of DEGs related to muscle development and metabolite abundance, including 5-hydroxyhexanoic acid, 1D-chiro-Inositol, and cytidine-5-triphosphate. In contrast, trigonelline, glutaric acid, and taurohyodeoxycholic acid sodium salt exhibited a significantly negative association with muscle development-related DEGs (Figure 8A). Compared to the control group, a high GAA diet significantly regulated the expression of genes associated with the TGF-β pathway. Moreover, significant negative correlations were observed between the expressions of DEGs related to the TGF-β pathway and the levels of metabolites, including S-Methyl-L-cysteine, pyridoxal 5′-phosphate hydrate, adenine, dGDP, APS, adenosine 5′-diphosphate, CDP, and adenylocuccinic acid. Conversely, a significant positive association was found between the abundance of muscle metabolites Phe-Pro and DEGs (Figure 8B). Compared to the Normal GAA group, we observed significant regulation of genes associated with muscle development or the TGF-β pathway in the High GAA group. Furthermore, excluding the bone morphogenetic protein receptor type 2 (BMPR2) gene, significant positive correlations were found between the expressions of DEGs and the abundance of metabolites, including dGDP, IDP, adenosine 5′-diphosphate, APS, adenine, adenylocuccinic acid, and CDP (Figure 8C).

## 4 Discussion

GAA serves as a precursor for the biosynthesis of creatine in animals. Numerous studies have reported that supplementing broilers with 0.6–1.2 g/kg of GAA can significantly enhance their production performance (9–12). Our previous findings have demonstrated that broilers in the 1.2 g/kg GAA group and 3.6 g/kg GAA group exhibited significantly higher final weight and average daily gain compared to the control group (*p* < 0.05), while the feed-to-gain ratio was significantly lower than that observed in the control group (*p* < 0.05) (18). Meanwhile, both at 21 and 42 days of age, the breast muscle percentage and leg muscle percentage of broilers in the1.2 g/kg GAA group were significantly higher compared to those in the 3.6 g/kg GAA group (*p* < 0.05), whereas the leg muscle percentage of broilers in the group supplemented with 3.6 g/kg GAA was significantly lower than that observed in the control group (*p* < 0.05) (18). Furthermore, compared to the control group, supplementation with 1.2 g/kg GAA resulted in a significant increase in pH levels for both breast and leg muscles of broilers, and effectively reducing heterogeneity occurrence within breast muscles. However, supplementation with 3.6 g/kg GAA did not yield significant improvements in meat quality (19). The aforementioned findings are in line with the effect of GAA on muscle development in broilers as demonstrated by this study.

The activation of IGF1 genes facilitates protein synthesis and enhances muscle development (20). It has been reported that the inclusion of GAA at a dosage of 1.2 g/kg in broiler feed resulted in elevated levels of IGF1, potentially linked to enhanced muscle growth (9). The results of this study, in conjunction with HE staining and IGF1 immunofluorescence staining of muscle tissue, demonstrated that supplementation with 1.2 g/kg GAA significantly promotes the development of muscle fibers in broiler breast muscles. Conversely, no significant effect was observed with a higher dosage of 3.6 g/kg GAA, suggesting the aforementioned potential developmental promotion may be reversed. The *in vitro* study conducted on C2C12 cells demonstrated that GAA promotes myoblast differentiation by activating the AKT/mTOR/S6K signaling pathway via the regulation of miR-133a-3p and miR-1a-3p. Meanwhile, GAA supplementation enhances myotube growth by up-regulating the expression of myosin heavy chain (MyHC) protein, thereby increasing myotube thickness and cross-sectional area of the gastrocnemius muscle (21). In the field of research on skeletal muscle growth and development, myostatin (MSTN) is widely recognized as a crucial negative regulator with significant physiological functions (22), while GAA and its metabolite creatine induce the down-regulation of MSTN expression, thereby abolishing its inhibitory effect on muscle growth (23). Furthermore, the muscle fibers of the mammalian fetus undergo gradual maturation post-delivery, and muscle growth is dependent on modifications in both muscle fiber volume and transitions between distinct types of muscle fibers (24). Relevant research has demonstrated that dietary GAA supplementation promotes the transformation of skeletal muscle fiber types from fast-twitch to slow-twitch, by enhancing the expression of PPARG coactivator 1 alpha (PGC1α) and the activation of calcineurin/nuclear factor of activated T cells (CaN/NFAT) pathway in finishing pigs (25). In summary, GAA exerts regulatory control over muscle development genes and facilitates the transformation of muscle fiber types, thereby modulating the diameter of muscle fibers.

We performed RNA-Seq analysis to screen for DEGs associated with muscle development in broiler breast muscle across the three experimental groups. The key genes identified were IHH, ASB2, and ANKRD2. Previous studies have demonstrated the impact of skeletal components on muscle development and emphasized the strong association between IHH and enhanced muscular growth (26). The presence of the ANKRD2 gene in skeletal muscle fibers has been demonstrated, highlighting its involvement in the formation of carnosin-related stretch receptivity complex (27). Moreover, ANKRD2 not only governs nuclear transcription cofactors but also plays a pivotal role in muscle cell differentiation and myoblast proliferation (28). These studies suggest the involvement of IHH and ANKRD2 genes in muscle development, indicating that upregulating their expression has the potential to enhance muscle development. This aligns with our findings, where dietary supplementation of 1.2 g/kg GAA significantly increased the expression of IHH and ANKRD2 genes in the muscle tissue of 42-day-old Cobb broilers. Additionally, another relevant study demonstrated a negative correlation between ASB2 expression and muscle fiber expression, indicating that ASB2 is a gene associated with muscle mass, and up-regulation of its expression may alleviate fibrosis in muscle tissue (29). The present study demonstrates that dietary supplementation of 1.2 g/kg GAA significantly up-regulates the expression of the ASB2 gene in muscle tissue of 42-day-old Cobb broilers, suggesting its potential to enhance muscle development and reduce fibrosis occurrence. These findings are consistent with our previous research indicating that dietary supplementation of 1.2 g/kg GAA effectively improves broiler meat quality.

TGF-β is a multifunctional cytokine involved in various cellular processes, encompassing cell growth, differentiation, and apoptosis (30). Studies have revealed that factors such as nutritional status and inflammatory response can regulate the expression and activity of TGF-β, thereby exerting an influence on muscle development and meat quality. During myogenesis, the TGF-β signaling pathway plays a pivotal role in promoting both muscle cell proliferation and differentiation, while augmenting the number and size of muscle fibers (31–34). During the process of muscle development, slow-twitch and fast-twitch muscle fibers undergo differentiation in response to specific physiological demands (35). Interestingly, it has been reported that the activation of TGF-β signaling pathway exerts a stimulatory effect on the development of slow muscle fibers while concurrently suppressing the maturation of fast muscle fibers (36, 37). Through KEGG analysis, we identified the TGF-β pathway as a shared pathway in both the High GAA vs. control group and the High GAA vs. Normal GAA group. Given that chicken breast primarily consists of fast muscle fibers, excessive activation of the TGF-β signaling pathway may have detrimental effects on its development (38, 39). The KEGG results suggest that a dosage of 3.6 g/kg GAA could potentially induce activation of the TGF-β pathway, subsequently counteracting the myogenic effect.

Trigonelline, a plant alkaloid initially isolated from *Trigonella foenum-graecum* L., commonly known as fenugreek (40), is also a byproduct of niacin metabolism and constitutes approximately 1% of the dry weight in roasted coffee beans (41). The compound trigonelline has been demonstrated to possess sedative, antibacterial, antiviral activities. It also exhibits inhibitory effects on platelet aggregation and demonstrates anti-tumor properties. Moreover, trigonelline enhances memory retention and displays hypoglycemic, hypolipidemic, and antimigraine effects (42–48). The beneficial effects of trigonelline on diabetes have been extensively documented, encompassing its capacity to reduce blood glucose and lipid levels, enhance insulin sensitivity index and insulin content, up-regulate antioxidant enzyme activity, and attenuate lipid peroxidation (49). The inclusion of 1.2 g/kg GAA has been observed to decrease the concentrations of trigonelline in chicken breast, implying a potential mechanism through which GAA influences meat quality.

S-methyl-L-cysteine, a sulfur-containing amino acid present in garlic, has been reported to exhibit antilipidemic activity (50–52). dGDP, also known as deoxythymidine diphosphate, serves as an intermediate molecule during the process of DNA synthesis (53). Adenosine, a purine nucleoside involved in both DNA and RNA synthesis, plays a crucial role in cellular growth and development. 5’-Diphosphate is a nucleotide formed through the dephosphorylation of GTP (guanosine triphosphate) and participates in various biochemical processes such as energy metabolism, as well as DNA and RNA synthesis (54). APS, an adenylate kinase, plays a pivotal role as a key enzyme in cellular nucleotide metabolism (55). CDP, which stands for cytidine diphosphate, is a nucleotide species. Adenylocuccinic acid, also known as adenosine succinate, is a purine nucleotide derivative and serves as an intermediary in the biosynthesis of adenylate (56).

The nucleotides adenine, dGDP, APS, adenosine 5′-diphosphate, CDP, and adenylocuccinic acid play integral roles in energy metabolism, signal transduction, cycle regulation, and protein synthesis in muscle cells, and their involvement is pivotal in regulating the processes of muscle cell proliferation, differentiation, and apoptosis (57–60). An appropriate amount of GAA can enhance *in vivo* nucleotide synthesis, and form stable complexes with nucleotides to improve their utilization. In this study, we observed that elevated levels of GAA induced activation of the TGF-β pathway, and a negative correlation was identified between the expression of genes associated with the TGF-β pathway and the aforementioned metabolites. These findings suggest that an excess of GAA can activate the TGF-β signaling pathway, leading to down-regulation of potential metabolites expression levels, including S-methyl-L-cysteine, pyridoxal 5′-phosphate hydrate, adenine, dGDP, and APS. Consequently, this reduction in above metabolite levels may impede normal muscle development.

## 5 Conclusion

This study validates the impact of different levels of GAA on broiler breast muscle tissue development and elucidates the underlying molecular mechanisms through the association analysis of transcriptome and quasi-targeted metabolomics. Our findings demonstrate that a dietary inclusion of 1.2 g/kg GAA promotes broiler breast muscle development, while a higher dose of 3.6 g/kg GAA may counteract this improvement by over-activating the TGF-β signaling pathway and reducing nucleotide metabolites such as adenosine 5′-diphosphate, CDP, adenylocuccinic acid, among others. These results provide a foundation for identifying key functional genes, signaling pathways, and metabolites associated with poultry muscle development and offer a theoretical basis for enhancing performance and meat quality in broilers using GAA as a feed additive.

## Data availability statement

The datasets presented in this study can be found in online repositories. The names of the repository/repositories and accession number(s) can be found in the article/Supplementary material.

## Ethics statement

The animal studies were approved by Life Sciences Ethics Committee of Yunnan Agricultural University (Approval ID: 202203094). The studies were conducted in accordance with the local legislation and institutional requirements. Written informed consent was obtained from the owners for the participation of their animals in this study.

## Author contributions

JH: Data curation, Formal analysis, Software, Visualization, Writing – original draft. SR: Data curation, Software, Writing – review & editing. MLiu: Investigation, Methodology, Writing – original draft. MLi: Investigation, Methodology, Writing – original draft. JR: Investigation, Methodology, Writing – original draft. JJ: Conceptualization, Resources, Writing – review & editing. CG: Conceptualization, Project administration, Writing – review & editing. WC: Data curation, Funding acquisition, Investigation, Project administration, Validation, Writing – original draft.

## Footnotes

^1^http://www.genome.jp/kegg/

^2^http://www.hmdb.ca/
