# Supplementary material for: Association analysis of transcriptome and quasi-targeted metabolomics reveals the regulation mechanism underlying broiler muscle tissue development at different levels of dietary guanidinoacetic acid
Source: Front Vet Sci. 2024 Apr 25;11:1384028. doi: 10.3389/fvets.2024.1384028 (PMC11080945; doi:10.3389/fvets.2024.1384028)
Supplement: Supplementary file 1 [file Data_Sheet_1.docx]

Supplementary Material

Association analysis of transcriptome and quasi-targeted metabolomics reveals the regulation mechanism underlying broiler muscle tissue development at different levels of dietary guanidinoacetic acid

**Jieyun Hong^1, †^, Sayed Haidar Abbas Raza^3, †^, Mengqian Liu^1^, Mengyuan Li^1^, Jinrui Ruan^1^, Junjing Jia^1,2,*^, Changrong Ge^1,2,*^ and Weina Cao^1,2,*^**

^1^College of Animal Science and Technology, Yunnan Agricultural University, Kunming 650201, China

^2^Yunnan Provincial Key Laboratory of Animal Nutrition and Feed, Yunnan Agricultural University, Kunming 650201, China

^3^Guangdong Provincial Key Laboratory of Food Quality and Safety / Nation-Local Joint Engineering Research Center for Machining and Safety of Livestock and Poultry Products, South China Agricultural University, Guangzhou 510642, China

* Author to whom correspondence should be addressed.

† These authors contributed equally to this work.

*** Correspondence:** Weina Cao: [849989054@qq.com](mailto:849989054@qq.com)

# Supplementary Data

The raw data of transcriptome presented in the study are deposited in the Sequence Read Archive repository, accession number PRJNA1089708.

The raw result of quasi-targeted metabolomics analysis has been provided as supplementary materials in a zip file. Please download the file “Result-X101SC22030966-Z01-J001-B1-42 (quasi-targeted metabolomics).ZIP”.

# Supplementary Figures and Tables

## Supplementary Figures (1-10)


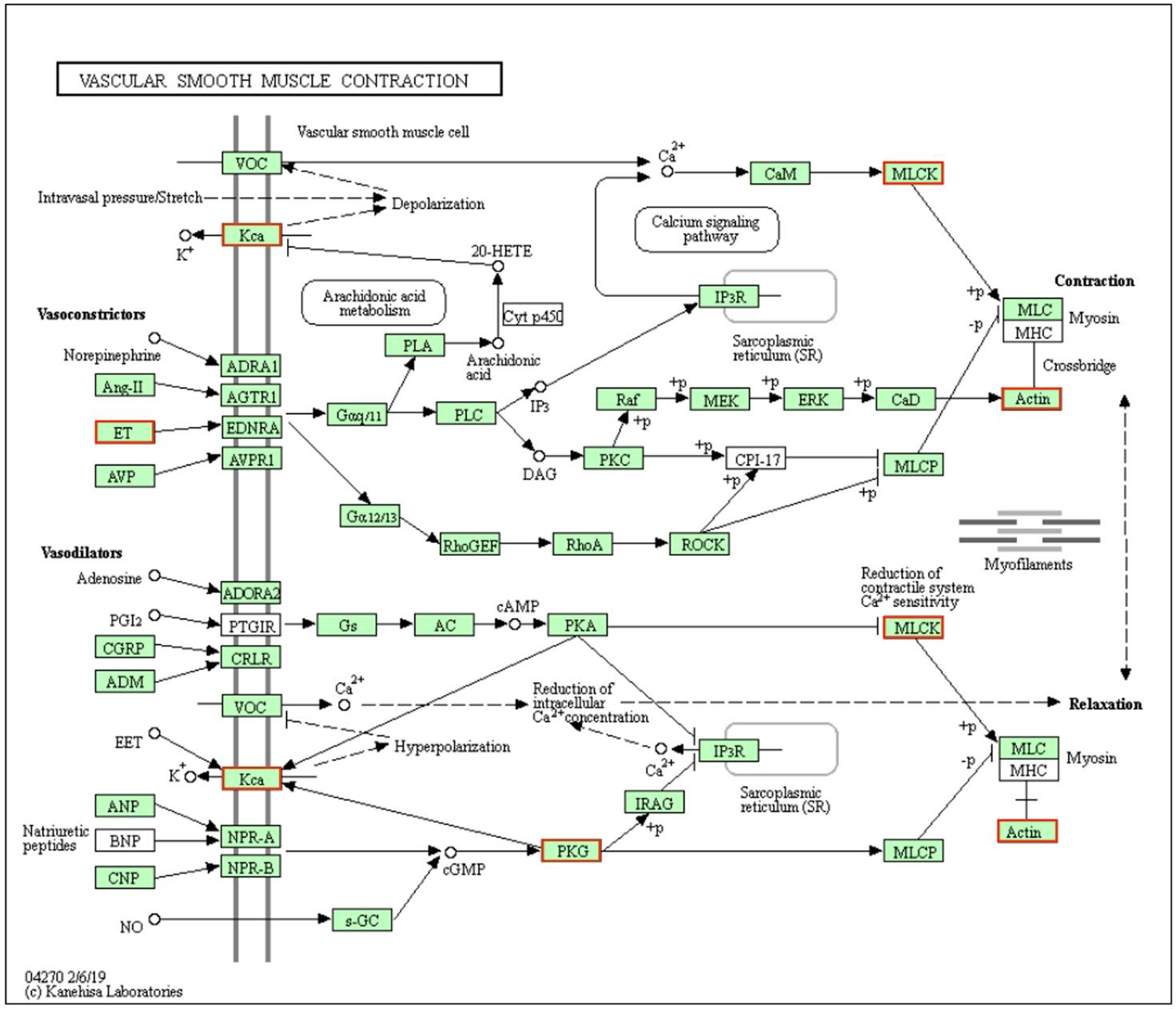


**Supplementary Figure 1.** KEGG enrichment pathway map of the expression regulation of core DEGs in vascular smooth muscle contraction pathway for Normal GAA group vs control group in RNA-seq analysis.


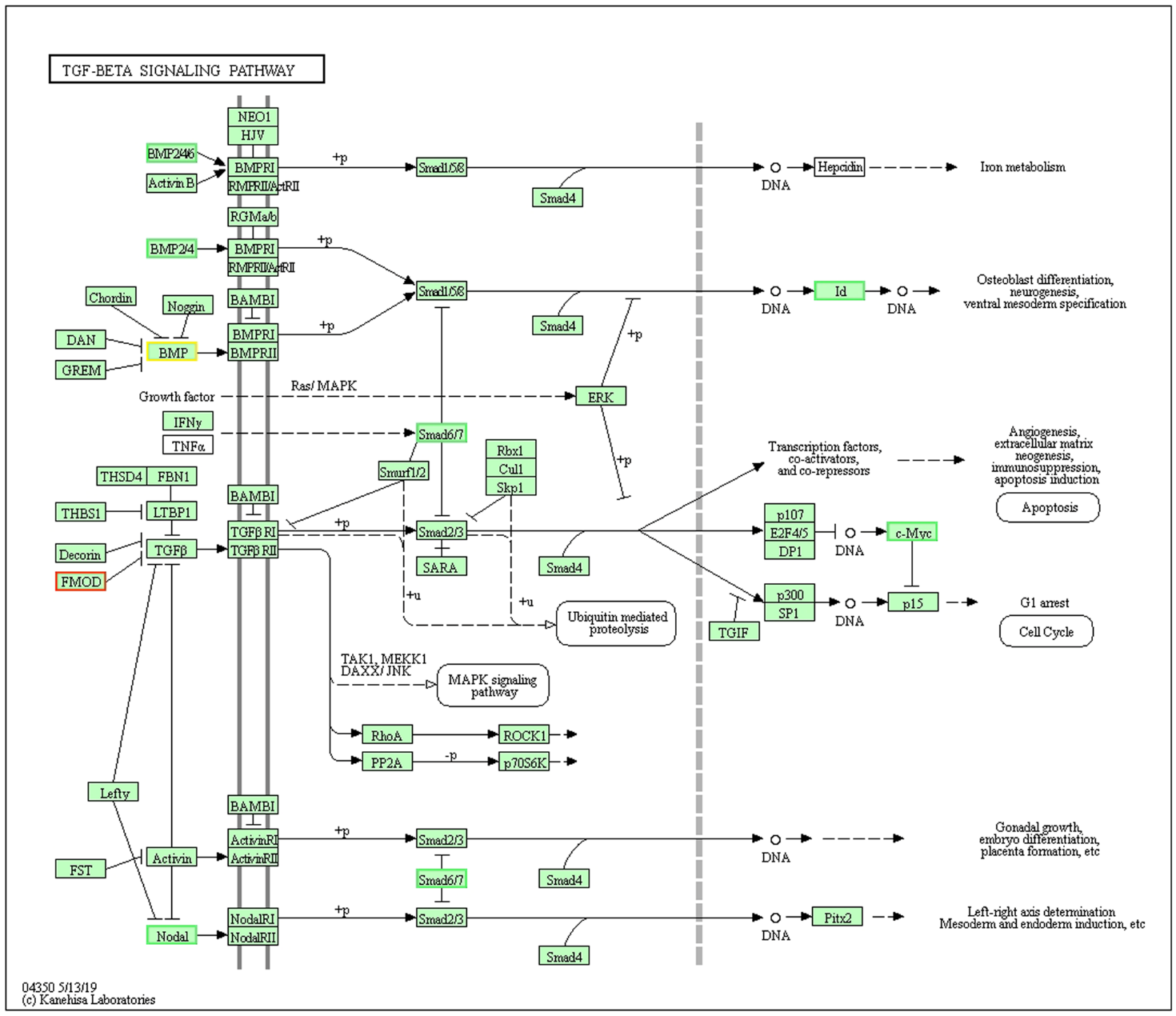


**Supplementary Figure 2.** KEGG enrichment pathway map of the expression regulation of core DEGs in TGF-β signaling pathway for High GAA group vs control group in RNA-seq analysis.


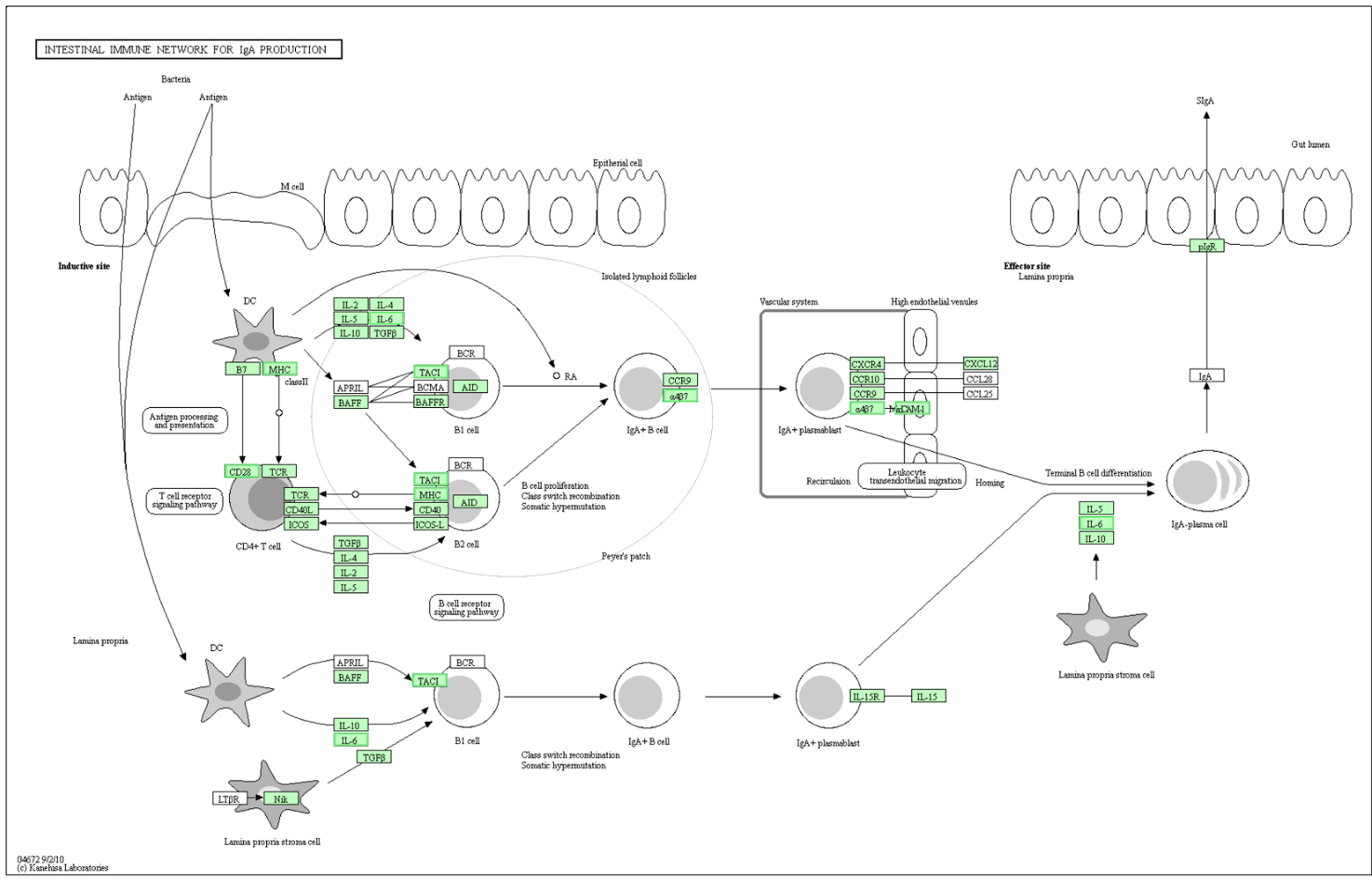


**Supplementary Figure 3.** KEGG enrichment pathway map of the expression regulation of core DEGs in intestinal immune network for IgA production pathway for High GAA group vs control group in RNA-seq analysis.


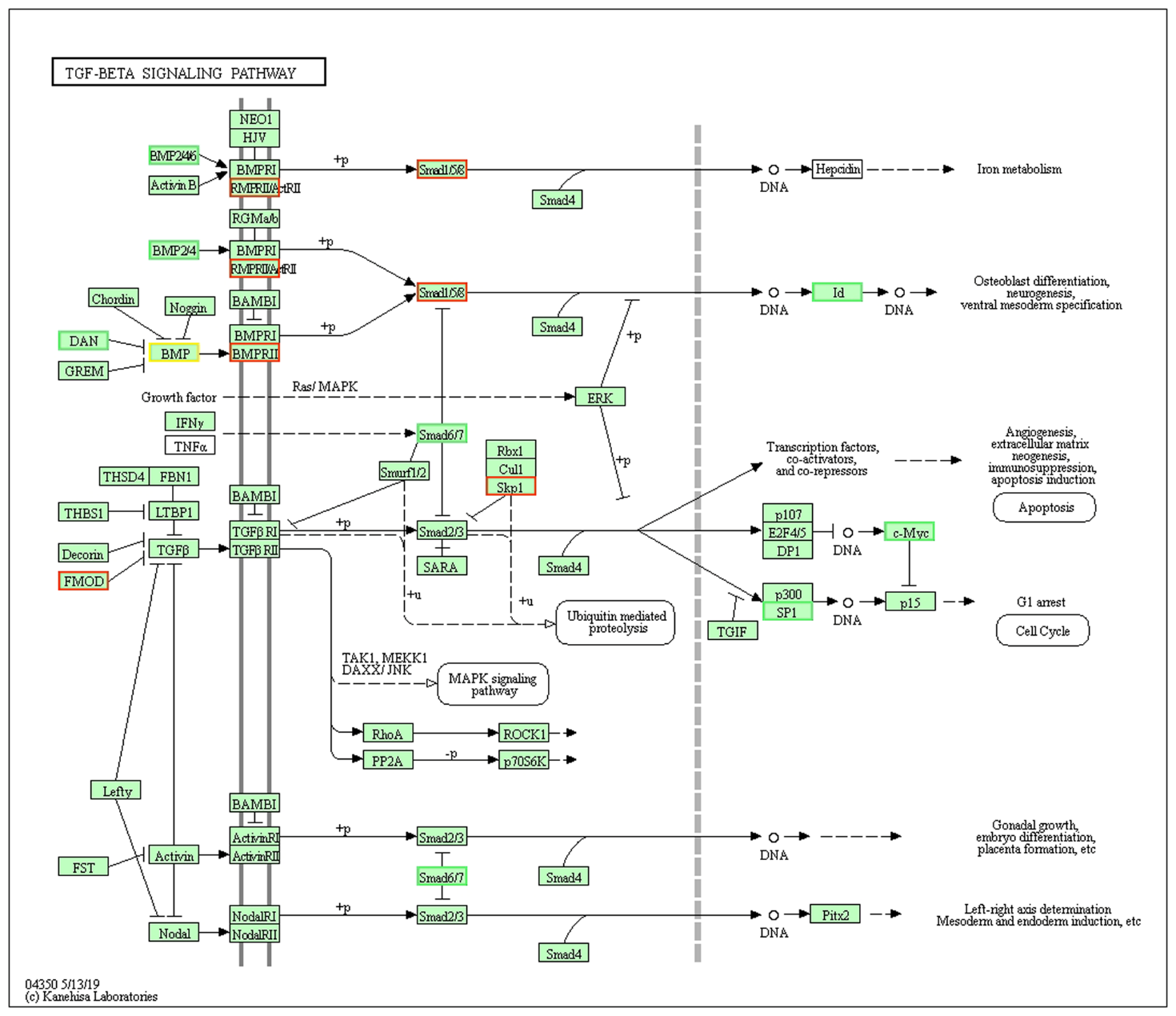


**Supplementary Figure 4.** KEGG enrichment pathway map of the expression regulation of core DEGs in TGF-β signaling pathway for High GAA group vs Normal GAA group in RNA-seq analysis.


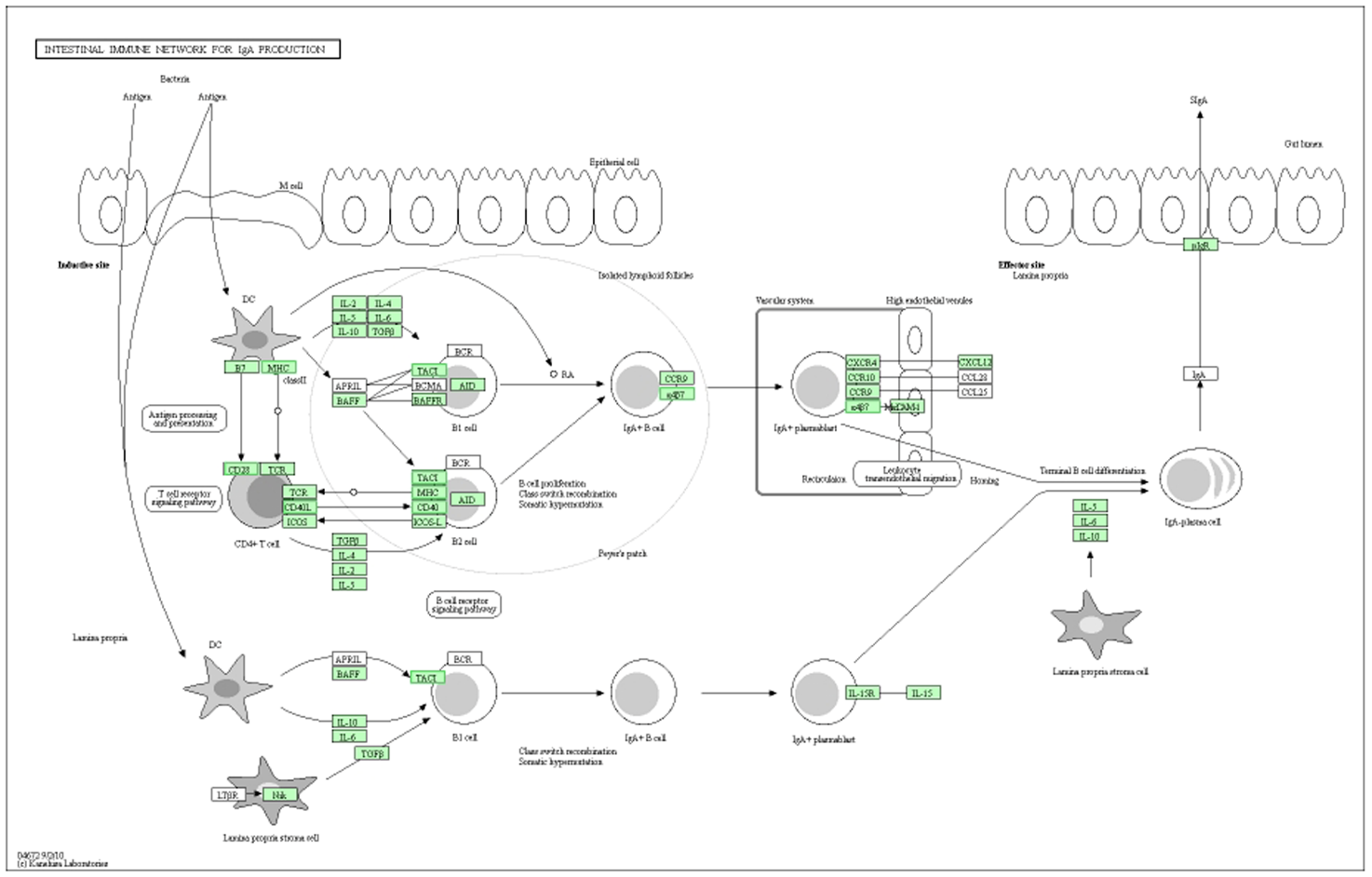


**Supplementary Figure 5.** KEGG enrichment pathway map of the expression regulation of core DEGs in intestinal immune network for IgA production pathway for High GAA group vs Normal GAA group in RNA-seq analysis.


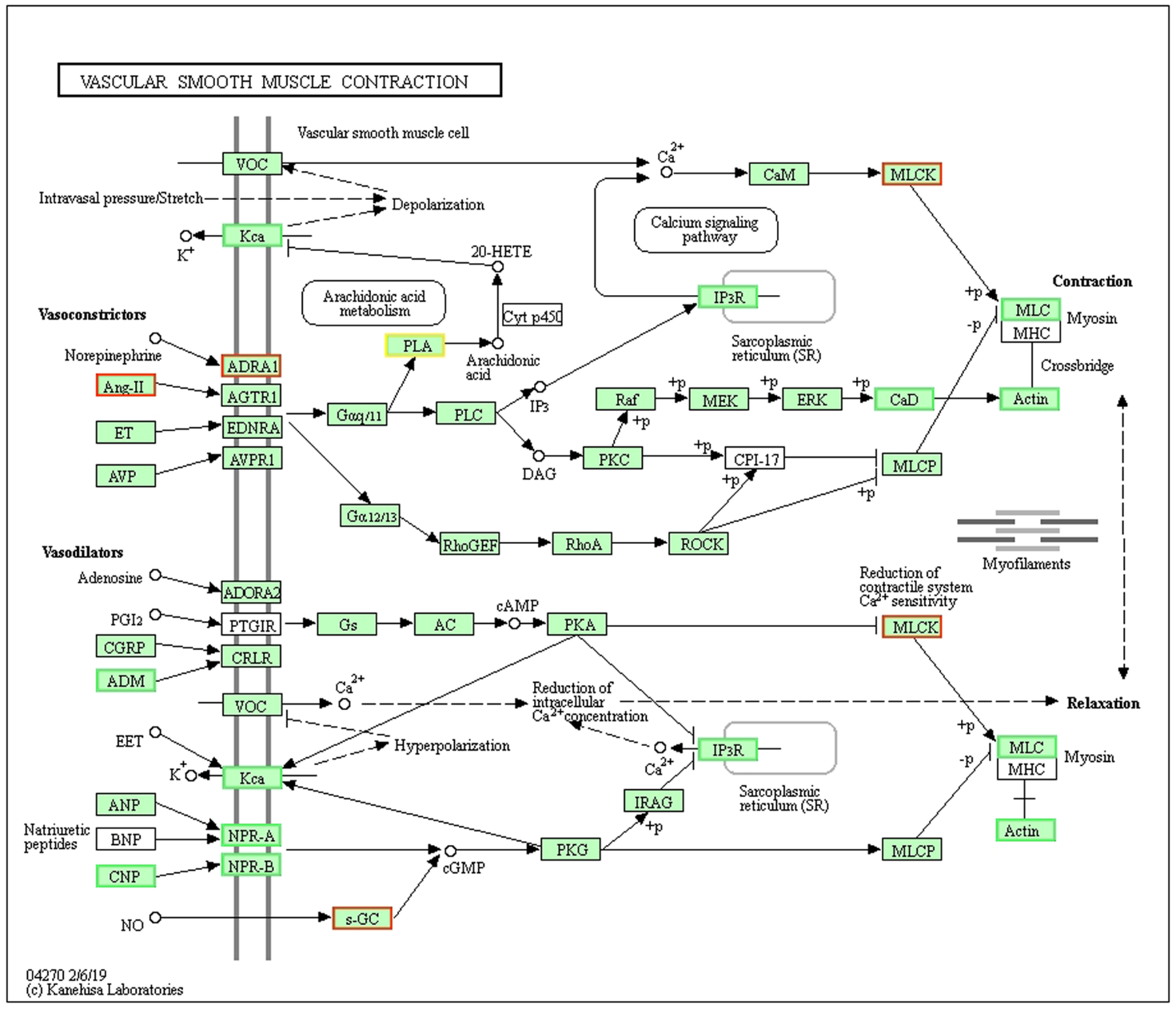


**Supplementary Figure 6.** KEGG enrichment pathway map of the expression regulation of core DEGs in vascular smooth muscle contraction pathway for High GAA group vs Normal GAA group in RNA-seq analysis.


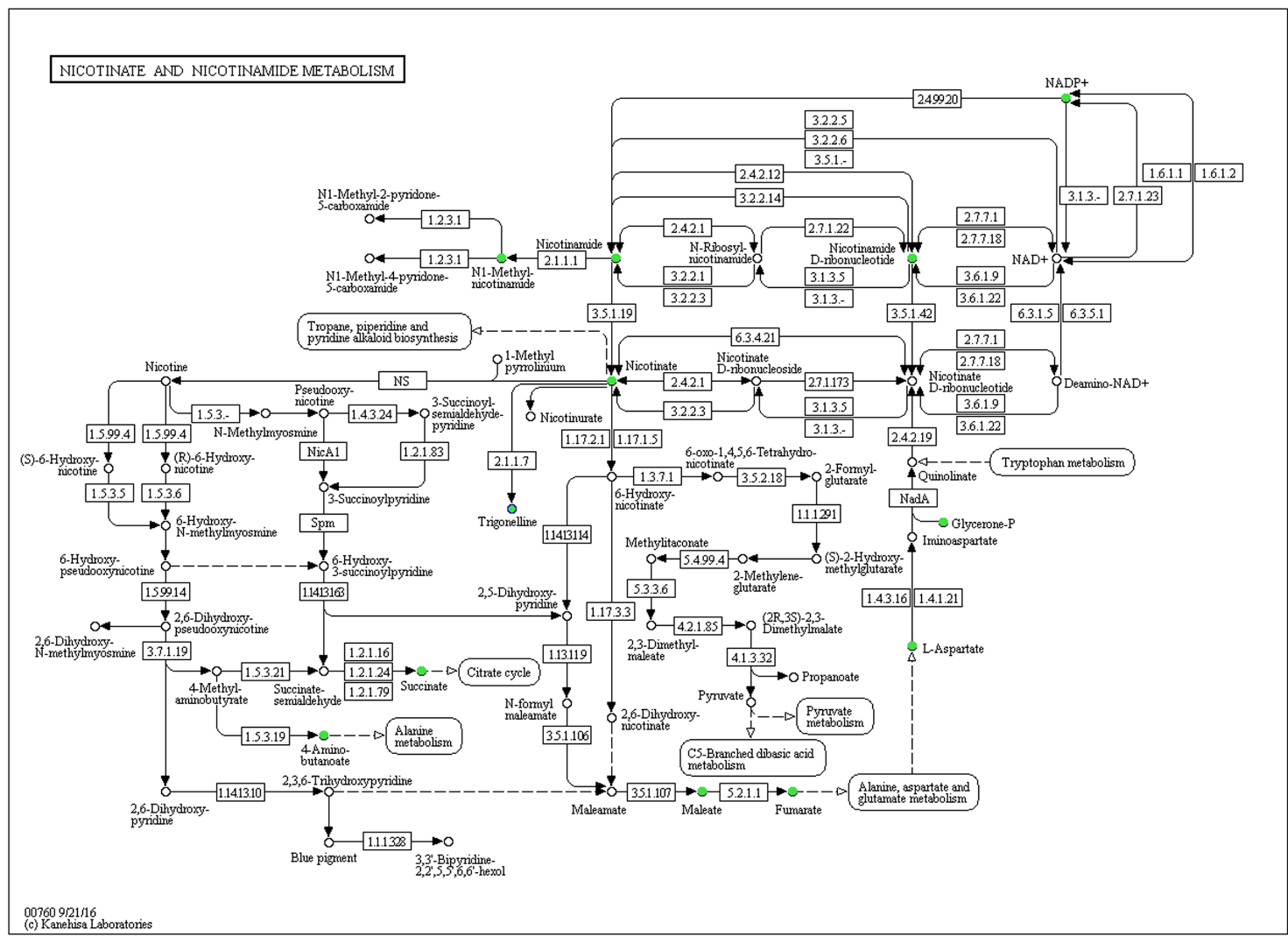


**Supplementary Figure 7.** KEGG enrichment pathway map of the specific enrichment of core SDMs in nicotinate and nicotinamide metabolism pathway for Normal GAA group vs control group in metabolomics analysis.


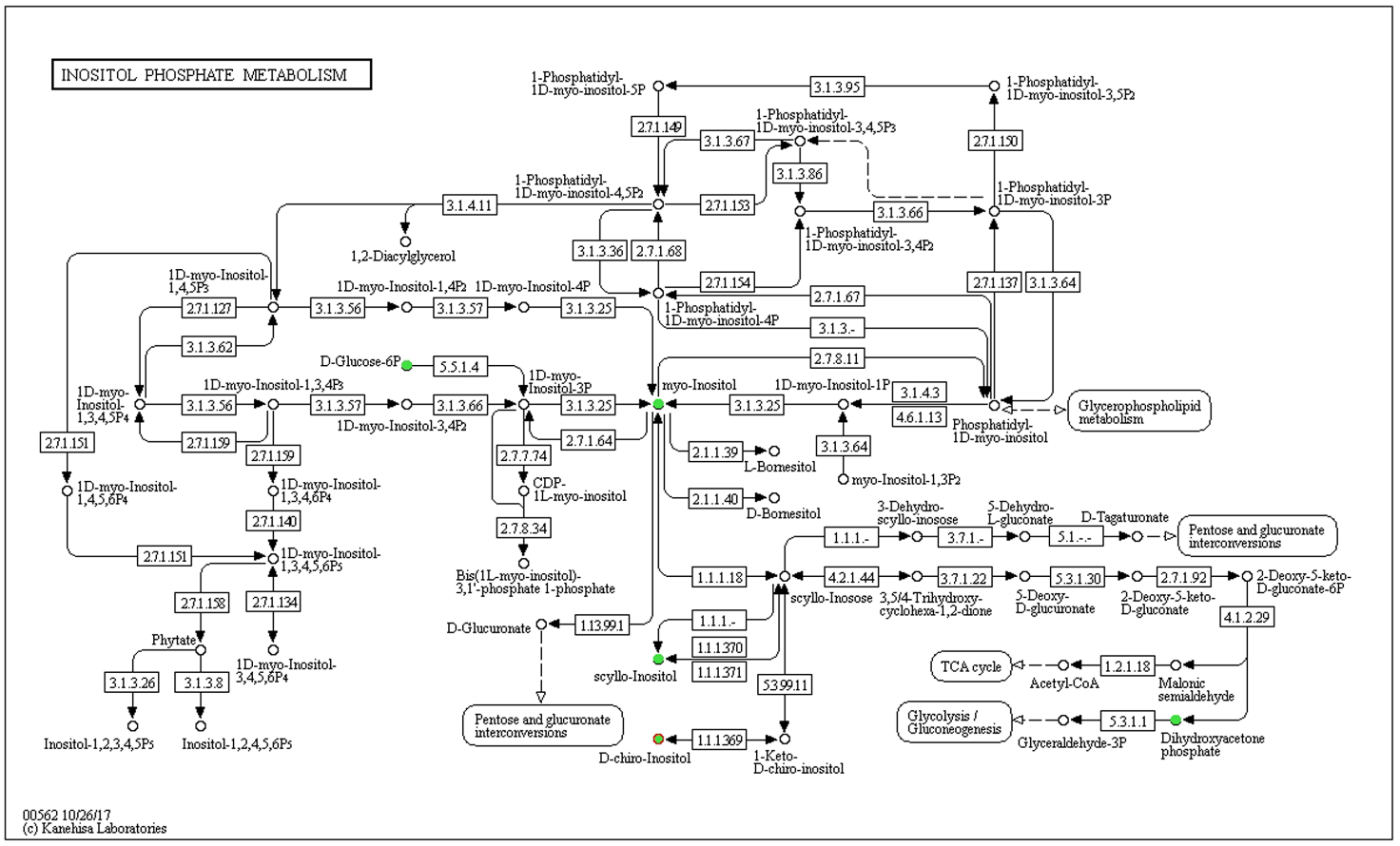


**Supplementary Figure 8.** KEGG enrichment pathway map of the specific enrichment of core SDMs in inositol phosphate metabolism pathway for Normal GAA group vs control group in metabolomics analysis.


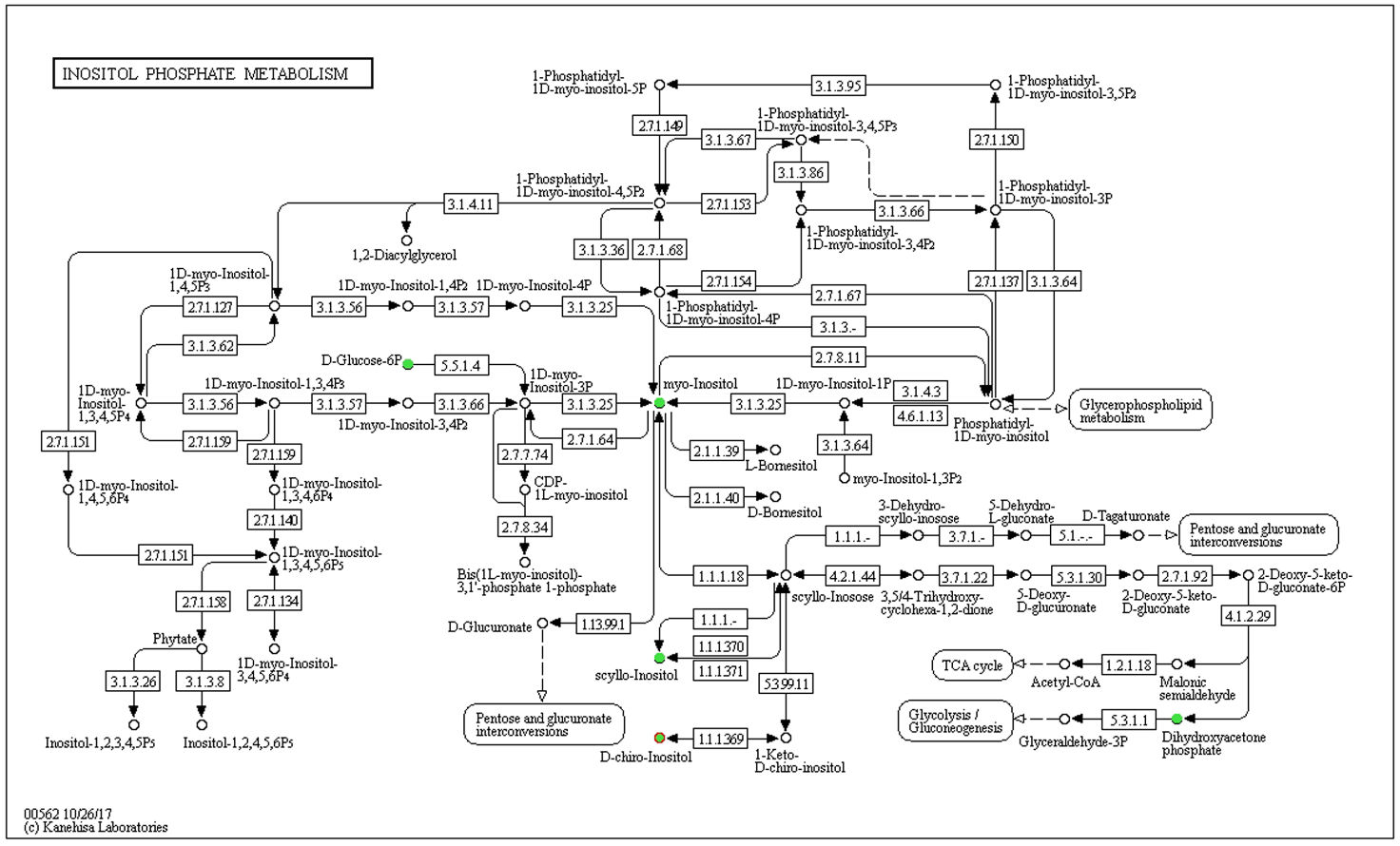


**Supplementary Figure 9.** KEGG enrichment pathway map of the specific enrichment of core SDMs in inositol phosphate metabolism pathway for High GAA group vs control group in metabolomics analysis.


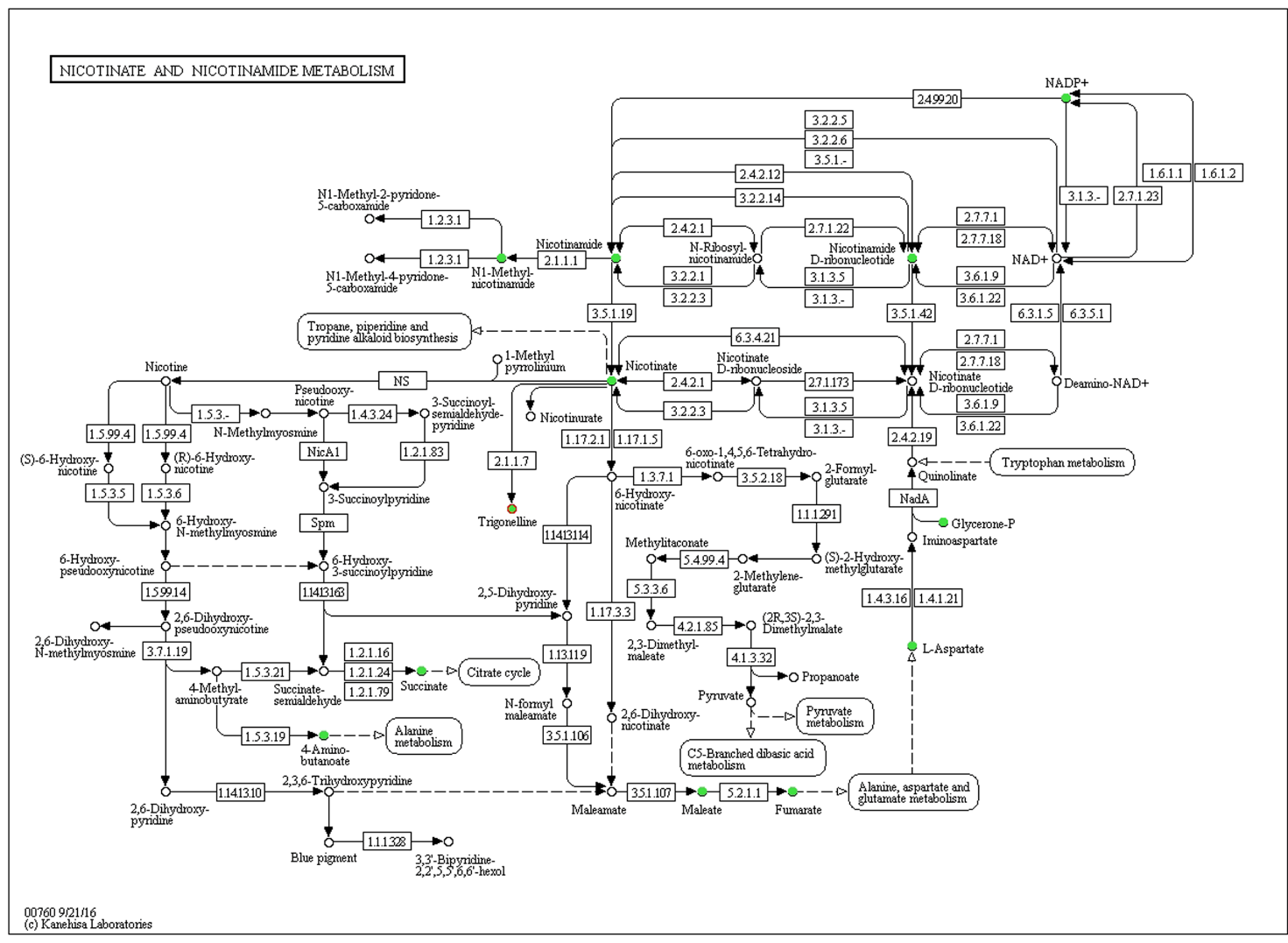


**Supplementary Figure 10.** KEGG enrichment pathway map of the specific enrichment of core SDMs in nicotinate and nicotinamide metabolism pathway for High GAA group vs Normal GAA group in metabolomics analysis.
