# Supplementary material for: Association analysis of transcriptome and quasi-targeted metabolomics reveals the regulation mechanism underlying broiler muscle tissue development at different levels of dietary guanidinoacetic acid
Source: Front Vet Sci. 2024 Apr 25;11:1384028. doi: 10.3389/fvets.2024.1384028 (PMC11080945; doi:10.3389/fvets.2024.1384028)
Supplement: Supplementary file 2 [file Data_Sheet_1.ZIP › Result-X101SC22030966-Z01-J001-B1-42 (quasi-targeted metabolomics)/2.MetAnnotation/2-MetAnnotation-readme.pdf]

MetAnnotation Readme

|-- 2. MetAnnotation 【代谢物注释结果目录】

| -- **KEGG** 【基于KEGG 数据库注释代谢物通路结果】

| |--meta_all_kegg_anno_category.xls 【KEGG 通路注释结果】

| |--meta_all.KEGG.Anno.{png,pdf} 【KEGG 注释结果统计图】

| |---meta_all.KEGG.Anno.xls 【代谢物KEGG 注释结果列表】

| -- **HMDB** 【基于HMDB 数据库注释代谢物分类结果】

| |--meta_all_hmdb_anno_category.xls 【HMDB 分类注释结果】

| |--meta_all.HMDB.Anno.{png,pdf} 【HMDB 分类注释结果统计图】

| |-- meta_all.HMDB.Anno.xls 【代谢物HMDB 注释结果列表】

| -- **Lipidmaps** 【基于Lipidmaps 数据库注释代谢物分类结果】

| |--meta_all_lipidmaps_anno_category.xls 【Lipidmaps 分类注释结果】

| |--meta_all.Lipidmaps.Anno.{png,pdf} 【Lipidmaps 分类注释结果统计图】

| |-- meta_all.Lipidmaps.Anno.xls 【代谢物Lipidmaps 注释结果列表】

KEGG

meta_all_kegg_anno_category.xls

第一列：KO_Pathway_Level1，KEGG 数据库相应pathway 的第一层级名称；

第二列：KO_Pathway_Level2，KEGG 数据库相应pathway 的第二层级名称；

第三列：Meta_Num，对应第二层级中注释到的代谢物数目；

第四列：Metabolites，代谢物ID

meta_all.KEGG.Anno. {png,pdf}

横坐标代表代谢物数目，纵坐标代表注释到的KEGG 通路；该图展示的是pathway 一级分类各二级分类对应的代谢物数目。

meta_all_kegg_anno.xls

第一列：ID，代谢物 ID；

第二列：Name，代谢物英文名称；

第三列：Kegg_ID，注释到的 kegg 数据库中对应的 ID；

第四列：Kegg_name，代谢物在 kegg 数据库中对应的名称；

第五列：formula，代谢物分子式；

第六列：Kegg_map，代谢通路对应的 mapID 和名称；

HMDB

meta_all_hmdb_anno_category.xls

第一列：SuperClass，HMDB 中的二级分类，如：有机酸、脂类等类别；

第二列：Metabolites_nums，该分类注释到的代谢物数目；

第三列：Metabolites，代谢物ID；

第四列：Metabolite_Descriptions，代谢物描述。

meta_all.HMDB.Anno.{png,pdf}

横坐标代表代谢物的数目，纵坐标代表注释到的 HMDB 分类；该图展示 HMDB 中二级分类(SuperClass)对应的代谢物数目。

meta_all_hmdb_anno.xls

第一列：ID，代谢物 ID；

第二列：Name，代谢物英文名称；

第三列：Formula，代谢物分子式；
第四列：HMDB_ID，代谢物在 HMDB 数据库中对应的 ID；
第五列：Kingdom，第一层级分类；
第六列：SuperClass，第二层级分类；
第七列：Class，第三层级分类；
第八列：SubClass，第四级分类；
第九列：DirectParent，化合物的化学类别；
第十列：Source，化合物来源；

Lipidmaps

meta_all_lipidmaps_anno_category.xls

第一列：CATEGORY，lipidmaps 分类信息（八大类脂质）；
第二列：MAIN_CLASS，八大类下的主层级分类；
第三列：Metabolites_nums，MAIN_CLASS 层级中注释到的代谢物数目；
第四列：Metabolites，代谢物 ID；
第五列：Metabolite_Descriptions，代谢物描述；

meta_all.Lipidmaps.Anno.{png,pdf}

横坐标代表代谢物数目，纵坐标代表注释到的LIPID MAPS 脂质分类；该图展示的是LIPID MAPS 中8 大脂质分类（Category）下的主层级分类（Main_Class）对应的（脂质）代谢物数目。

meta_all_lipidmaps_anno.xls

第一列：ID，代谢物 ID；
第二列：Name，代谢物英文名称；
第三列：Formula，代谢物分子式；
第四列：Lipidmaps_ID，Lipidmaps 数据库中对应的 ID；
第五列：COMMON_NAME，常用名；
第五列：SYSTEMATIC_NAME，系统命名；
第六列：SYNONYMS，化合物同义名称；
第七列：CATEGORY，分类信息（八大类脂质）；
第八列：MAIN_CLASS，八大类下的主层级分类；
第九列：SUB_CLASS，主层级分类下的子类；
