# Supplementary material for: Association analysis of transcriptome and quasi-targeted metabolomics reveals the regulation mechanism underlying broiler muscle tissue development at different levels of dietary guanidinoacetic acid
Source: Front Vet Sci. 2024 Apr 25;11:1384028. doi: 10.3389/fvets.2024.1384028 (PMC11080945; doi:10.3389/fvets.2024.1384028)
Supplement: Supplementary file 2 [file Data_Sheet_1.ZIP › Result-X101SC22030966-Z01-J001-B1-42 (quasi-targeted metabolomics)/2.MetAnnotation/HMDB/meta_all.HMDB.Anno.pdf]

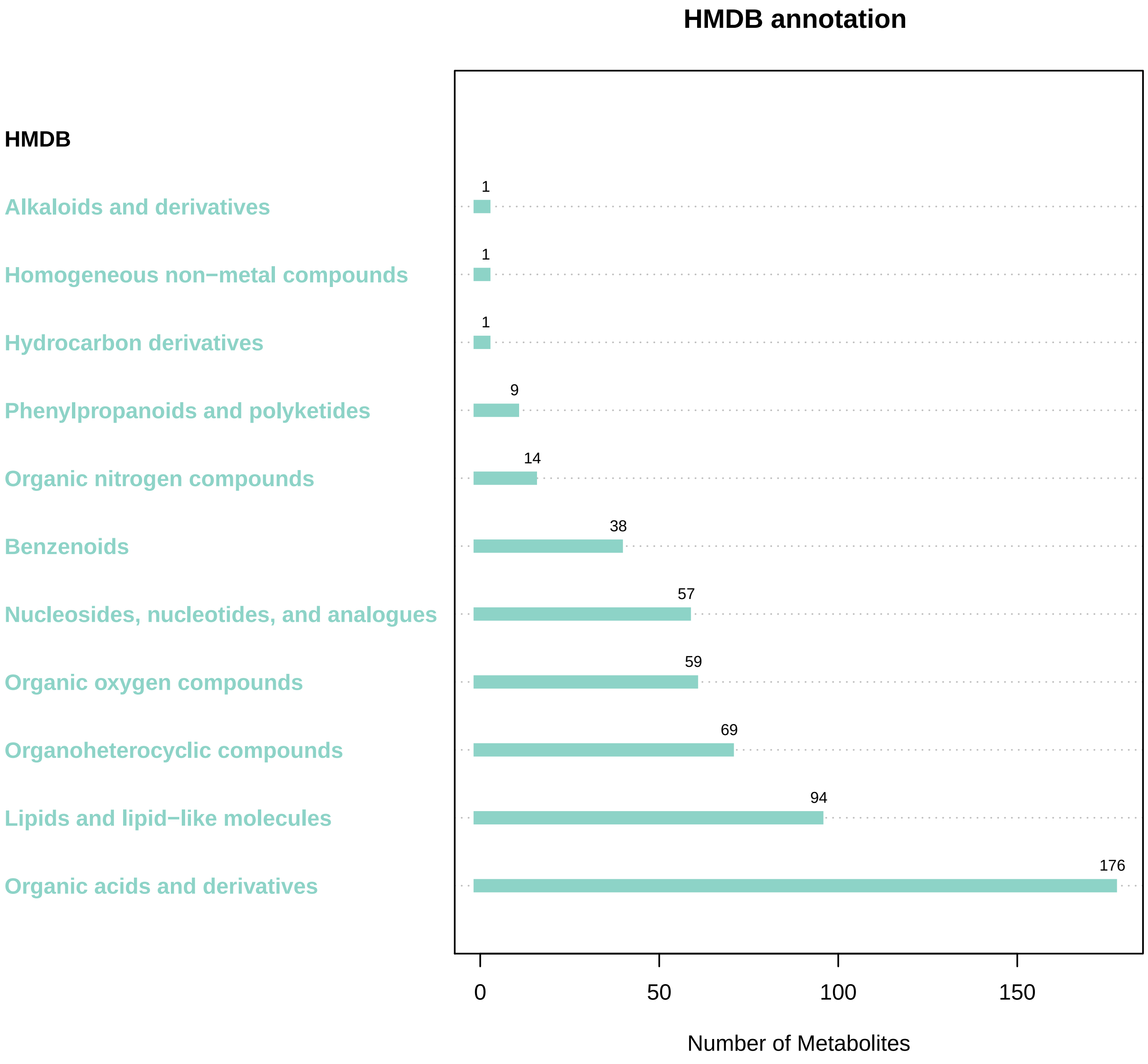
HMDB annotation
HMDB
Alkaloids and derivatives
Homogeneous non−metal compounds
Hydrocarbon derivatives
Phenylpropanoids and polyketides
Organic nitrogen compounds
Benzenoids
Nucleosides, nucleotides, and analogues
Organic oxygen compounds
Organoheterocyclic compounds
Lipids and lipid−like molecules
Organic acids and derivatives
1
1
1
9
14
38
57
59
69
94
176
0
50
100
150
Number of Metabolites
