# Supplementary material for: Association analysis of transcriptome and quasi-targeted metabolomics reveals the regulation mechanism underlying broiler muscle tissue development at different levels of dietary guanidinoacetic acid
Source: Front Vet Sci. 2024 Apr 25;11:1384028. doi: 10.3389/fvets.2024.1384028 (PMC11080945; doi:10.3389/fvets.2024.1384028)
Supplement: Supplementary file 2 [file Data_Sheet_1.ZIP › Result-X101SC22030966-Z01-J001-B1-42 (quasi-targeted metabolomics)/2.MetAnnotation/Lipidmaps/meta_all.Lipidmaps.Anno.pdf]

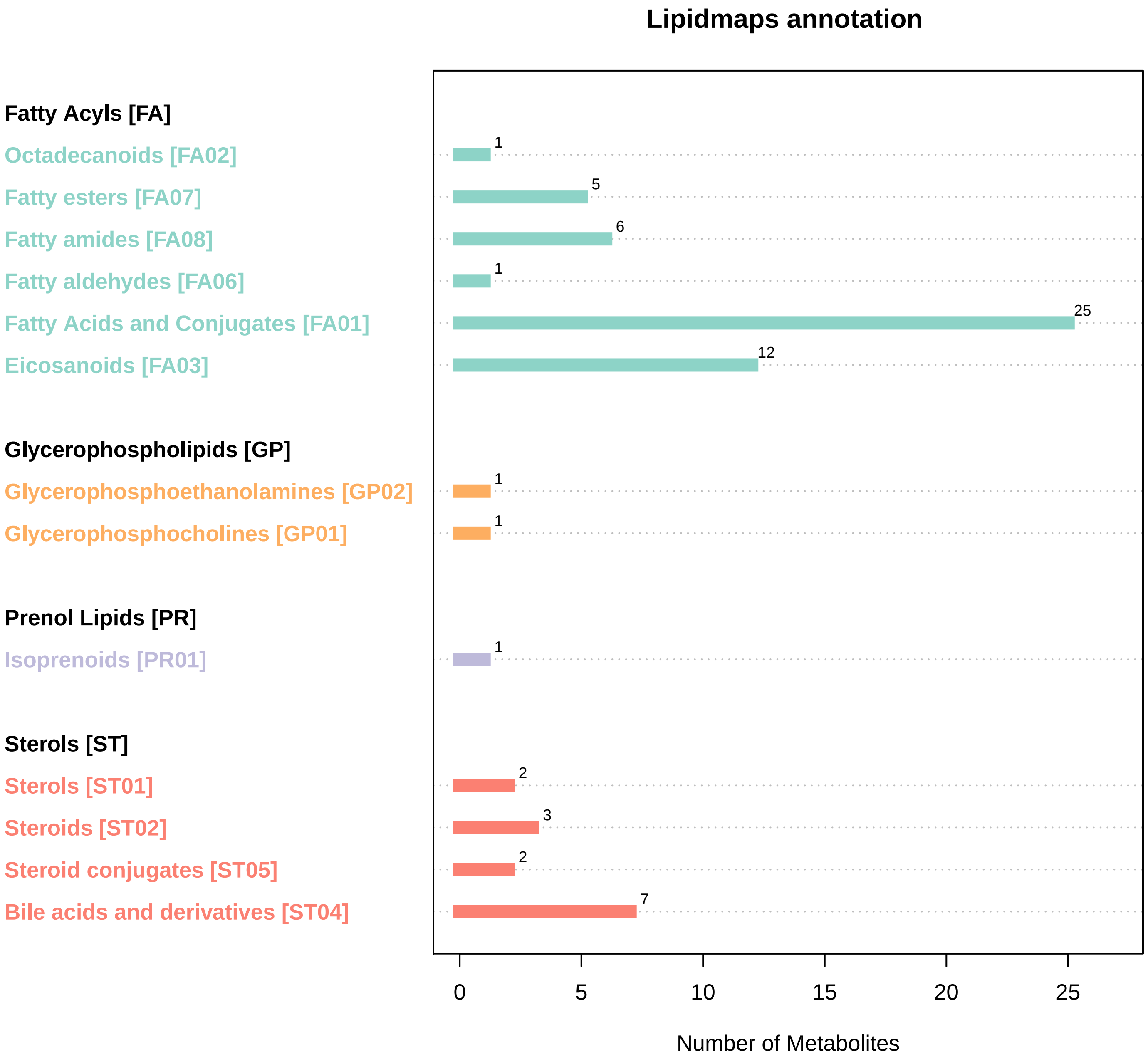

Lipidmaps annotation
Fatty Acyls [FA]
Octadecanoids [FA02]
Fatty esters [FA07]
Fatty amides [FA08]
Fatty aldehydes [FA06]
Fatty Acids and Conjugates [FA01]
Eicosanoids [FA03]
Glycerophospholipids [GP]
Glycerophosphoethanolamines [GP02]
Glycerophosphocholines [GP01]
Prenol Lipids [PR]
Isoprenoids [PR01]
Sterols [ST]
Sterols [ST01]
Steroids [ST02]
Steroid conjugates [ST05]
Bile acids and derivatives [ST04]
1
5
6
1
25
12
1
1
1
2
3
2
7
0
5
10
15
20
25
Number of Metabolites
