# Supplementary material for: Association analysis of transcriptome and quasi-targeted metabolomics reveals the regulation mechanism underlying broiler muscle tissue development at different levels of dietary guanidinoacetic acid
Source: Front Vet Sci. 2024 Apr 25;11:1384028. doi: 10.3389/fvets.2024.1384028 (PMC11080945; doi:10.3389/fvets.2024.1384028)
Supplement: Supplementary file 2 [file Data_Sheet_1.ZIP › Result-X101SC22030966-Z01-J001-B1-42 (quasi-targeted metabolomics)/3.MetDiffScreening/3-MetDiffScreening-readme.pdf]

MetDiffScreening Readme

|-- 3.MetDiffScreening　　【差异代谢物筛选结果目录】

| -- *.vs.*　　【样本比较对目录】
| |-- *.vs.*_all.xls　　【样本比较对定量分析结果】
| |-- *.vs.*_all_Diff.xls　　【样本比较对差异代谢物分析】
| |-- *.vs.*_all-PCA[.3D].{png,pdf}　　【样本比较对 PCA 分析】
| |-- *.vs.*_all-PCA-pcaloading.{png,pdf}　　【样本比较对 PCA loading 分析】
| |-- *.vs.*_all-PLSDA-{score,valid}.{png,pdf}【比较对 PLSDA 分析】
| |-- *.vs.*_all-PLSDA-loading.{png,pdf}　　【比较对 PLSDA loading 分析】
| |-- *.vs.*_all.xls.volcano.{png,pdf}　　【样本比较对火山图分析】

***.vs.*_all.xls（共有） and *.vs.*_all_Diff.xls（差异）**

第一列：Compound_ID，代谢物 ID；
第二列：Name，代谢物名称；
第三列：Chinese name，代谢物中文名称
第四列：RT [min]，保留时间；
第五列：Class(Chinese)，代谢物分类名称（中文）
第六列：Class(English)，代谢物分类名称（英文）
第七列：Formula，代谢物的分子式；
第八列：Molecular Weight，代谢物的相对分子量；
第九列-倒数第七列：不同样品定量值；
倒数第六列：FC，比较对差异倍数；
倒数第五列：log2FC，比较对差异倍数的 log2 值；
倒数第四列：Pvalue，比较对显著性 p-value；
倒数第三列：ROC，受试者工作特征曲线面积；
倒数第二列：VIP，变量重要性投影, 来反映每一个样品定量值对差异的贡献程度，一般设置 VIP>1；
倒数第一列：Up.Down，上调（up）或下调（down）；

***.vs.*_all-PCA[.3D].{png,pdf}**

样本比较对 PCA 分析[3D]图：通过 PCA 的方法，观察两组样本间的总体分布趋势。横坐标 PC1 和纵坐标 PC2 分别表示排名第一和第二的主成分的得分，不同颜色的散点表示不同实验分组的样本，椭圆为 95%的置信区间（生物学重复数目小于 4 时，无法展示 95%的置信椭圆）。

***.vs.*_all-pcaloading.{png,pdf}**

样本比较对 PCA loading 分析载荷图：载荷图（loading plot）的横坐标代表每个物质在第一主成分上的载荷大小（cosα），纵坐标代表每个物质在第二主成分上的载荷大小（cosβ）。载荷图本质上描述的是构成第一主成份和第二主成份的线性方程的系数，载荷的绝对值越大，对于主成份的影响就越大。

***.vs.*_all-PLSDA-score.{png,pdf}**

比较对 PLS-DA 得分散点图：运用偏最小二乘法回归建立代谢物表达量与样品类别之间的关系模型，来实现对样品类别的预测。横坐标为样本在第一主成分上的得分；

纵坐标为样本在第二主成分上的得分；R2Y 表示模型的解释率，Q2Y 用于评价 PLS-DA 模型的预测能力，且 R2Y 大于 Q2Y 时表示模型建立良好。

***.vs.*_all-PLSDA-valid.{png,pdf}**

比较对 PLS-DA 排序检验图：对模型进行排序检验，用以判别模型质量好坏及模型是否“过拟合”。 横坐标代表随机分组的 Y 与原始分组 Y 的相关性，纵坐标代表 R2 和 Q2 的得分；图中一个点表示一次检验。

***.vs.*_all-PLSDA- loading.{png,pdf}**

样本比较对 PCA loading 分析载荷图：载荷图（loading plot）的横坐标代表每个物质在第一主成分上的载荷大小（cosα），纵坐标代表每个物质在第二主成分上的载荷大小（cosβ）。载荷图本质上描述的是构成第一主成份和第二主成份的线性方程的系数，载荷的绝对值越大，对于主成份的影响就越大。

***.vs.*_all.xls.volcano.{png,pdf}**

样本比较对火山图：根据比较对间的差异代谢物的 VIP 值、P 值、FC 值绘制火山图，可直观显示差异代谢物的整体分布情况。横坐标表示代谢物在不同分组中的表达倍数变化(log2FC)，纵坐标表示差异显著性水平(-log10(p-value))，图中每个点代表一个代谢物，点的大小代表 VIP 值，显著上调的代谢物用红色点表示，显著下调的代谢物用绿色点表示。
