# Supplementary material for: Association analysis of transcriptome and quasi-targeted metabolomics reveals the regulation mechanism underlying broiler muscle tissue development at different levels of dietary guanidinoacetic acid
Source: Front Vet Sci. 2024 Apr 25;11:1384028. doi: 10.3389/fvets.2024.1384028 (PMC11080945; doi:10.3389/fvets.2024.1384028)
Supplement: Supplementary file 2 [file Data_Sheet_1.ZIP › Result-X101SC22030966-Z01-J001-B1-42 (quasi-targeted metabolomics)/4.MetDiffAnalysis/4-MetDiffAnalysis-readme.pdf]

MetDiffAnalysis Readme

|-- 4.MetDiffAnalysis 【差异代谢物分析结果目录】

| -- Venn_diff 【基于比较对的差异代谢物 venn 图】
| |--Diff_Venn_all.{png,svg} 【差异代谢物 venn 图】
| -- Heatmap_diff 【总差异代谢物热图结果】
| |--Diff_Heatmap_all{.cluster,cluster.detail}.{png,pdf} 【总差异代谢物聚类热图】
| |--Diff_Heatmap_all{.detail}.{png,pdf} 【总差异代谢物不聚类热图】
| -- *.vs.* 【比较的样本对目录】
| |-- *.vs.*_all.corr.xls 【差异代谢物相关性列表】
| |-- *.vs.*_all_Pvalue.xls 【基于相关性分析的 p 值列表】
| |-- *.vs.*_all_zscore.xls 【差异代谢物 zscore 分析列表】
| |-- *.vs.*_all_cluster_heatmap[,_detail].{png,pdf} 【差异代谢物聚类热图】
| |-- *.vs.*_all_heatmap[._detail].{png,pdf} 【差异代谢物不聚类热图】
| |-- *.vs.*_all_corr.{png,pdf} 【差异代谢物相关性分析图】
| |-- *.vs.*_all_zscore.{png,pdf} 【差异代谢物 z-score 图】
| |-- ROC_all 【差异代谢物 ROC 曲线图】

Diff_Heatmap_Name_all.xls

第一列：Compound_ID，代谢 ID；
第二列：Name，代谢物名称；
第三列-倒数第一列：样本对应的代谢物均值；

***.vs.*_all_corr.xls**

代谢物相关性分析表格；

***.vs.*_all_Pvalue.xls**

基于代谢物相关性分析的 P 值；

***.vs.*_all_zscore.xls**

代谢物 zscore 值表格；

Diff_Venn_ Intersection{ Union}.xls

Venn 图对应集合表格，"1"表示该代谢物在对应比较对中是差异代谢物，"0"则表示不是。

Diff_Heatmap_all{_cluster,cluster_detail}.{png,pdf}

总差异代谢物聚类热图：对各比较对之间的差异代谢物进行层次聚类分析，将差异代谢物相对定量值进行归一化转换并聚类。横向为代谢物的聚类，纵向为样本分组，聚类枝越短代表相似性越高。

Diff_Heatmap_all{,_detail}.{png,pdf}

总差异代谢物不聚类热图：将差异代谢物相对定量值进行归一化转换。横向为代谢物的聚类，纵向为样本分组。

***.vs.* _all_cluster_heatmap[,_detail].{png,pdf}**

差异代谢物聚类热图：对两组样本获得的差异代谢物进行层次聚类分析，得出同一比较对两组之间和组内代谢表达模式的差异情况。横向为代谢物的聚类，纵向为样本类型，聚类枝越短代表相似性越高。

***.vs.*_ all_heatmap[,_detail].{png,pdf}**

差异代谢物不聚类热图：将两组样本获得的差异代谢物相对定量值进行归一化转换，得出同一 比较对两组之间和组内代谢表达模式的差异情况。横向为代谢物的聚类，纵向为样本类型。

***.vs.*_all_corr.{png,pdf}**

差异代谢物相关性分析图：通过计算所有差异代谢物两两之间的皮尔逊相关系数，选取显著性水平 P-value 值从小到大排序的 Top20 的差异代谢物进行展示，可以查看代谢物与代谢物变化趋势的一致性。相关性最高为 1，为完全的正相关（红色），相关性最低为-1，为完全的负相关（蓝色），没有颜色的部分表示 P-value＞0.05。

***.vs.*_all_zscore.{png,pdf}**

差异代谢物 z-score 图：z-score（标准分数）是基于代谢物的相对含量转换而来的值，用于衡量同一水平面上代谢物的相对含量的高低。横坐标为 z-score 值，纵坐标为差异代谢物，每个圆圈代表一个样本。图中只展示了 Top30（按 p-value 值从小到大排序）的代谢物 Z-score 值。Z-score 超出 4 或-4 的样本无法展示。

ROC_all

差异代谢物 ROC 曲线图：ROC 曲线又叫受试者工作特征曲线或感受性曲线，根据一系列不同的二分类方式（分界值或决定域）绘制的曲线，差异代谢物的 ROC 曲线可用来评判潜在的生物标记物。横坐标为假阳性率（1-特异度），纵坐标为真阳性率（灵敏度）。
