# Supplementary figures and images for: Association analysis of transcriptome and quasi-targeted metabolomics reveals the regulation mechanism underlying broiler muscle tissue development at different levels of dietary guanidinoacetic acid (part 2 of 2)
Source: Front Vet Sci. 2024 Apr 25;11:1384028. doi: 10.3389/fvets.2024.1384028 (PMC11080945; doi:10.3389/fvets.2024.1384028)

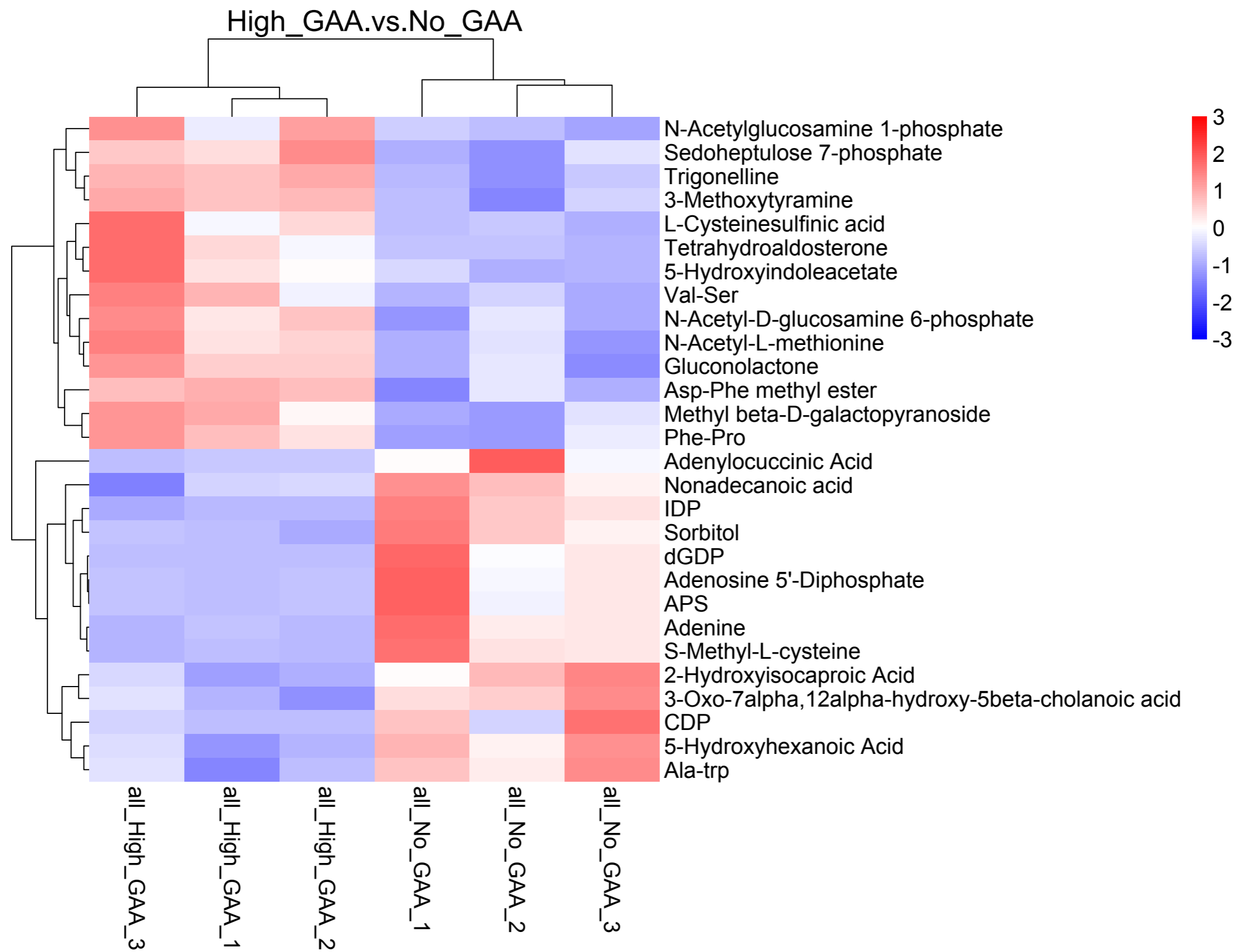

Supplement: Supplementary file 2 [file Data_Sheet_1.ZIP › Result-X101SC22030966-Z01-J001-B1-42 (quasi-targeted metabolomics)/4.MetDiffAnalysis/High_GAA.vs.No_GAA/High_GAA.vs.No_GAA_all_cluster_heatmap_detail.pdf]

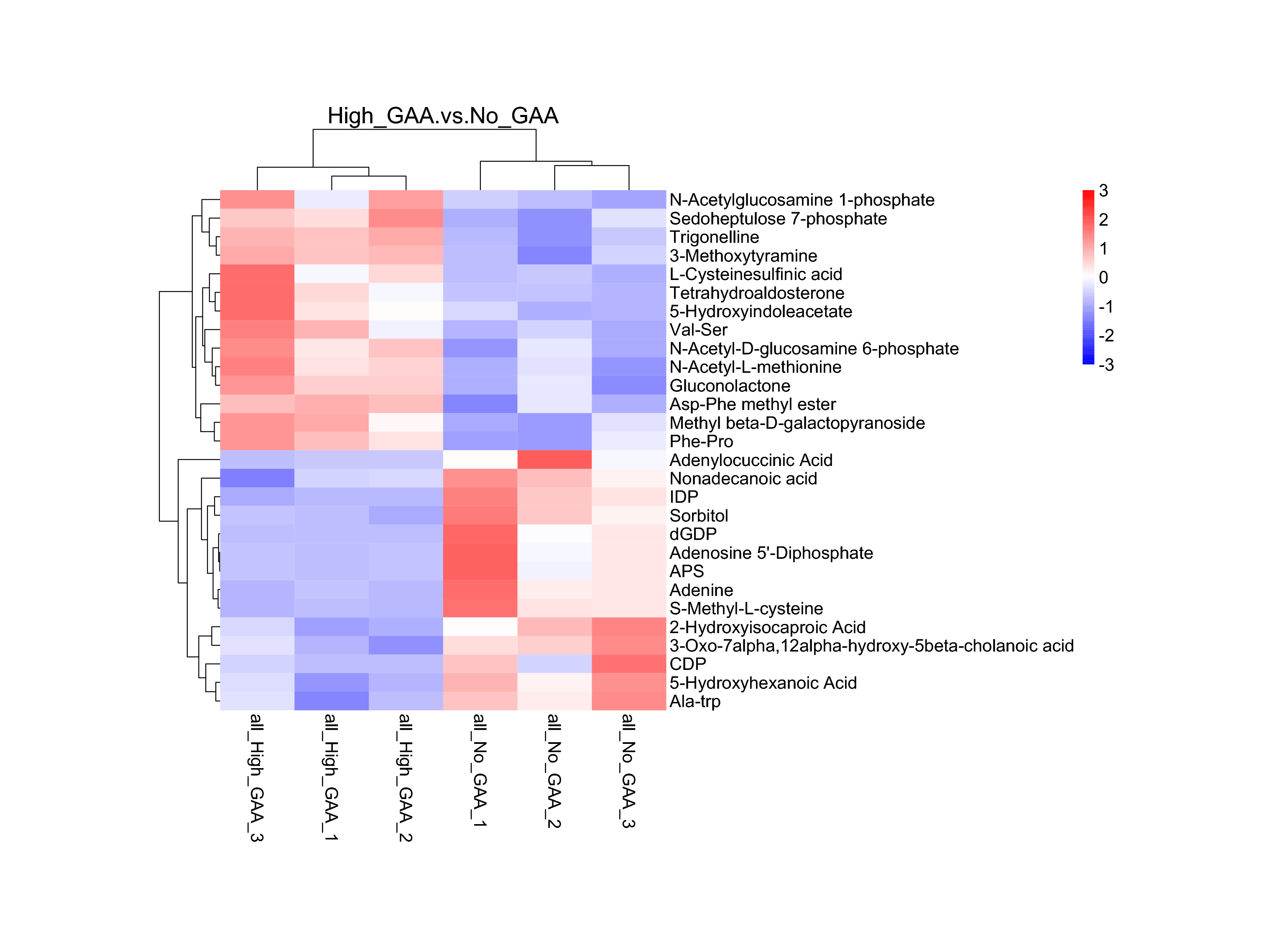

Supplement: Supplementary file 2 [file Data_Sheet_1.ZIP › Result-X101SC22030966-Z01-J001-B1-42 (quasi-targeted metabolomics)/4.MetDiffAnalysis/High_GAA.vs.No_GAA/High_GAA.vs.No_GAA_all_cluster_heatmap_detail.png]

High\_GAA.vs.No\_GAA

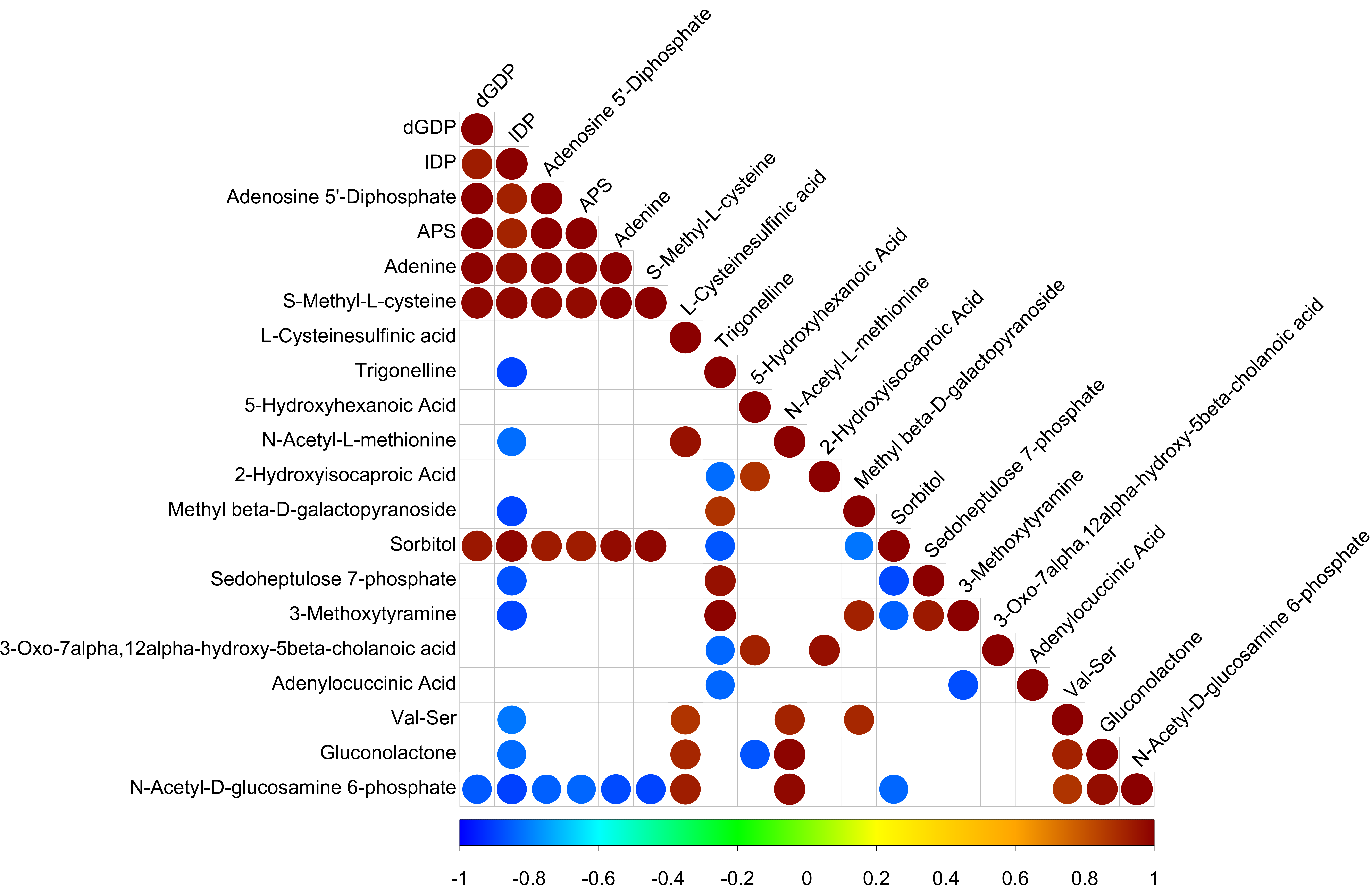

Supplement: Supplementary file 2 [file Data_Sheet_1.ZIP › Result-X101SC22030966-Z01-J001-B1-42 (quasi-targeted metabolomics)/4.MetDiffAnalysis/High_GAA.vs.No_GAA/High_GAA.vs.No_GAA_all_corr.pdf]

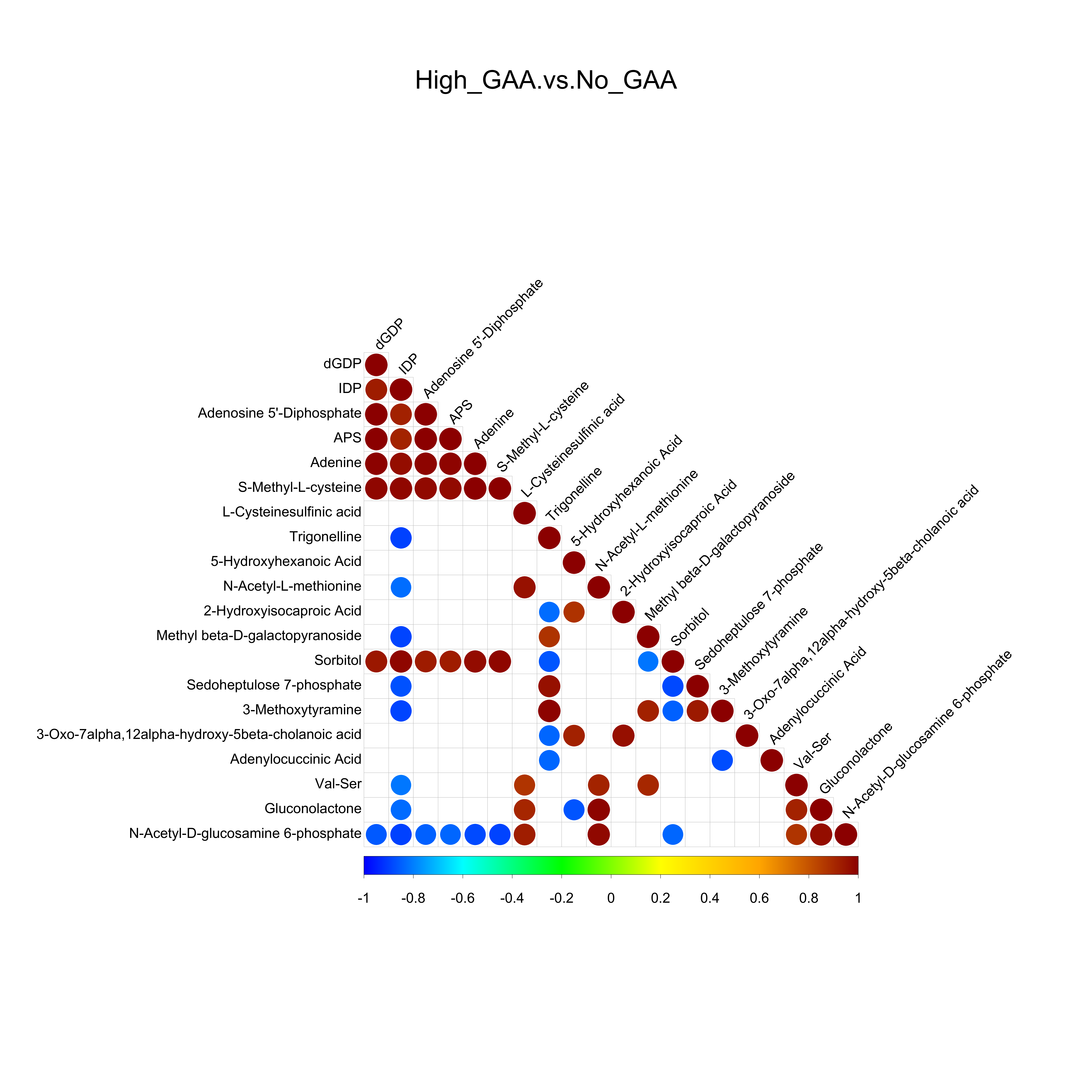

Supplement: Supplementary file 2 [file Data_Sheet_1.ZIP › Result-X101SC22030966-Z01-J001-B1-42 (quasi-targeted metabolomics)/4.MetDiffAnalysis/High_GAA.vs.No_GAA/High_GAA.vs.No_GAA_all_corr.png]

High\_GAA.vs.No\_GAA

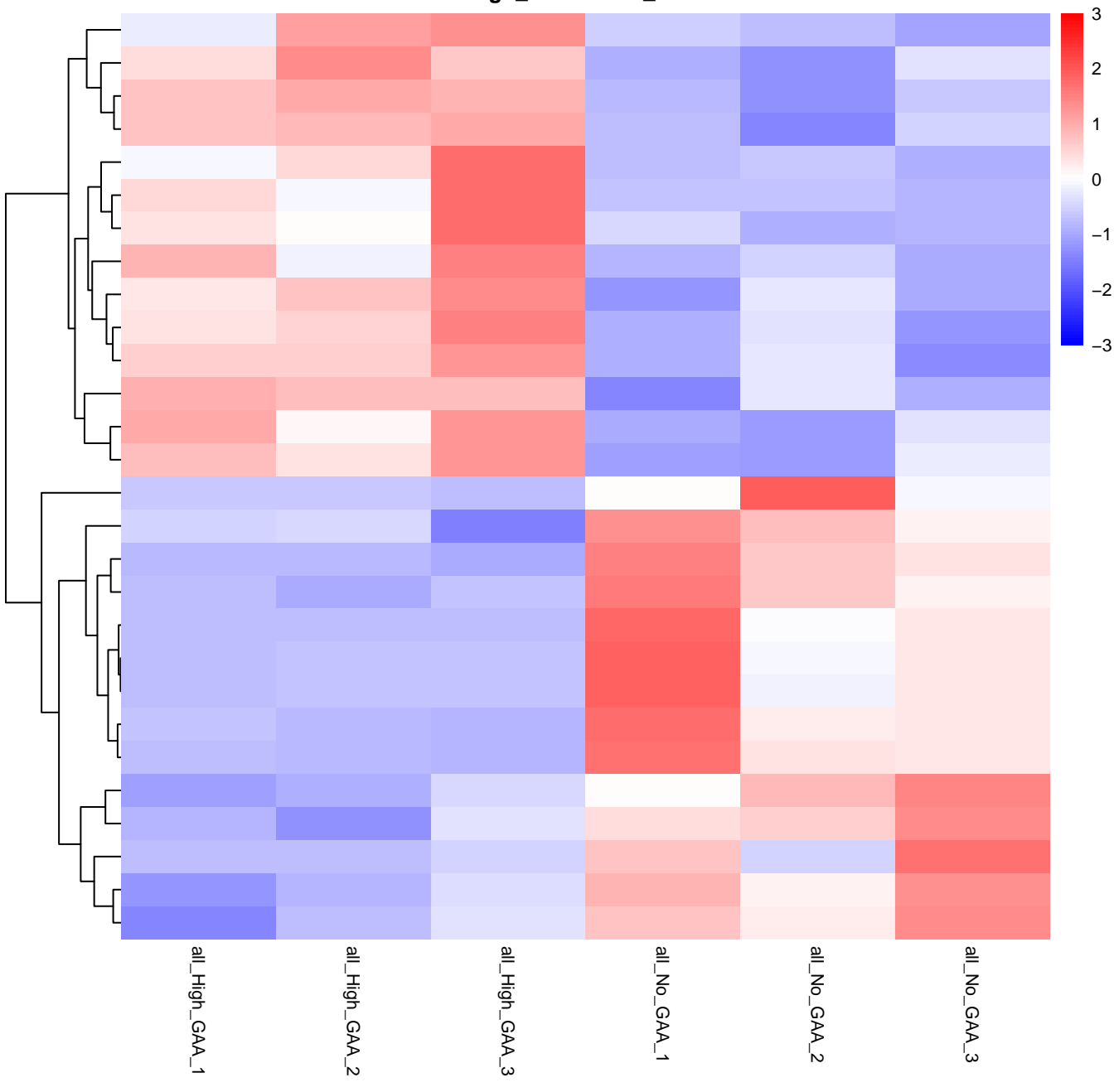

Supplement: Supplementary file 2 [file Data_Sheet_1.ZIP › Result-X101SC22030966-Z01-J001-B1-42 (quasi-targeted metabolomics)/4.MetDiffAnalysis/High_GAA.vs.No_GAA/High_GAA.vs.No_GAA_all_heatmap.pdf]

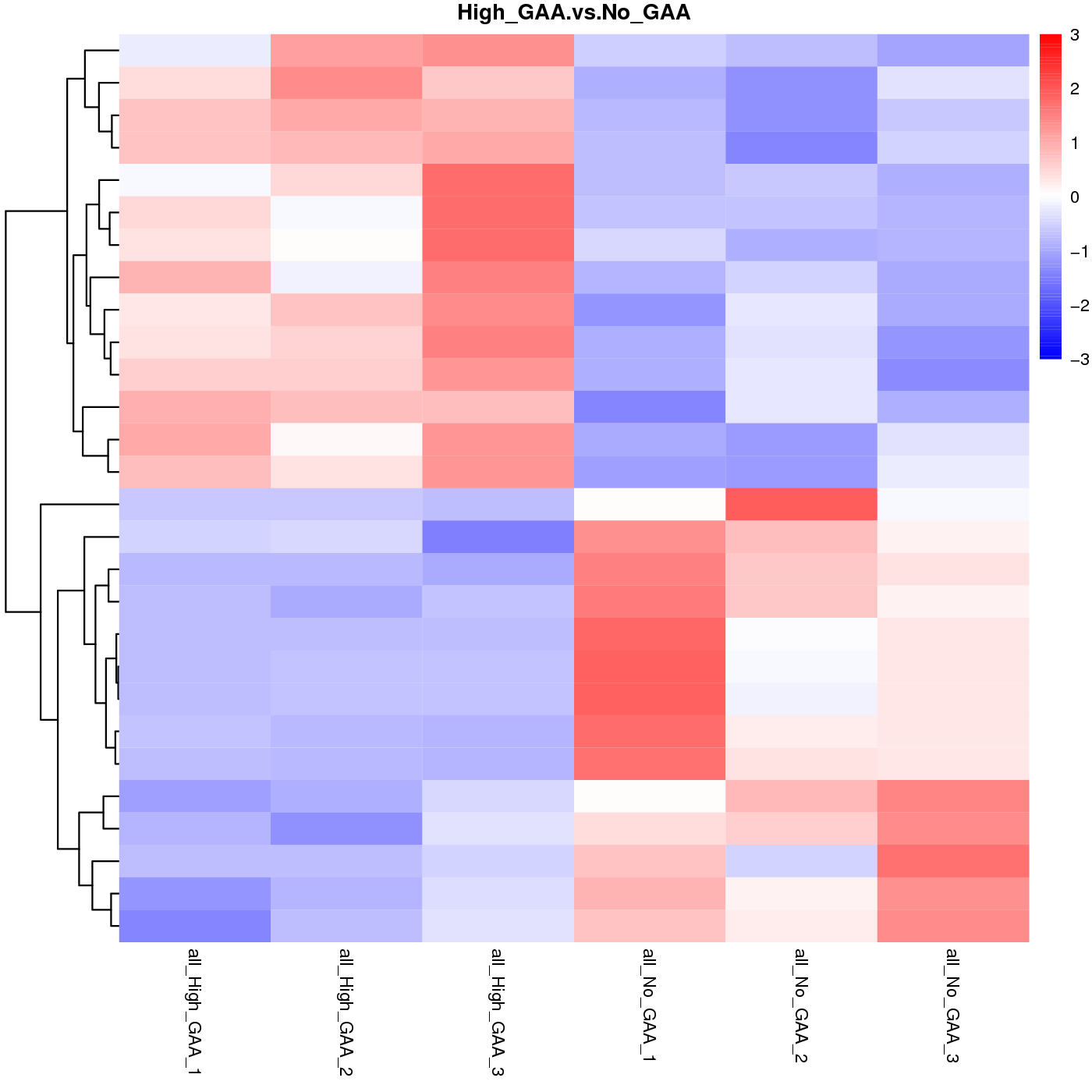

Supplement: Supplementary file 2 [file Data_Sheet_1.ZIP › Result-X101SC22030966-Z01-J001-B1-42 (quasi-targeted metabolomics)/4.MetDiffAnalysis/High_GAA.vs.No_GAA/High_GAA.vs.No_GAA_all_heatmap.png]

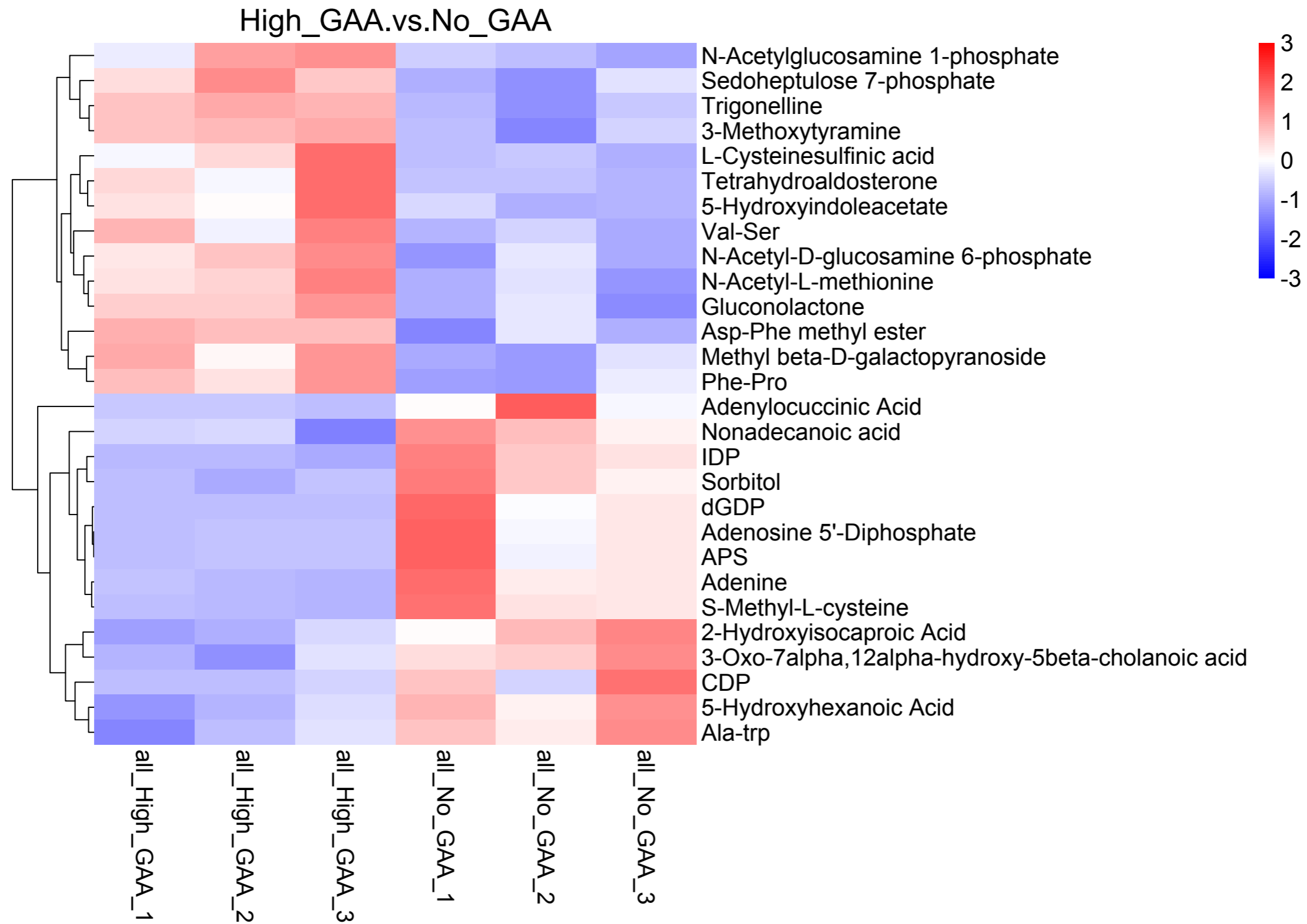

Supplement: Supplementary file 2 [file Data_Sheet_1.ZIP › Result-X101SC22030966-Z01-J001-B1-42 (quasi-targeted metabolomics)/4.MetDiffAnalysis/High_GAA.vs.No_GAA/High_GAA.vs.No_GAA_all_heatmap_detail.pdf]

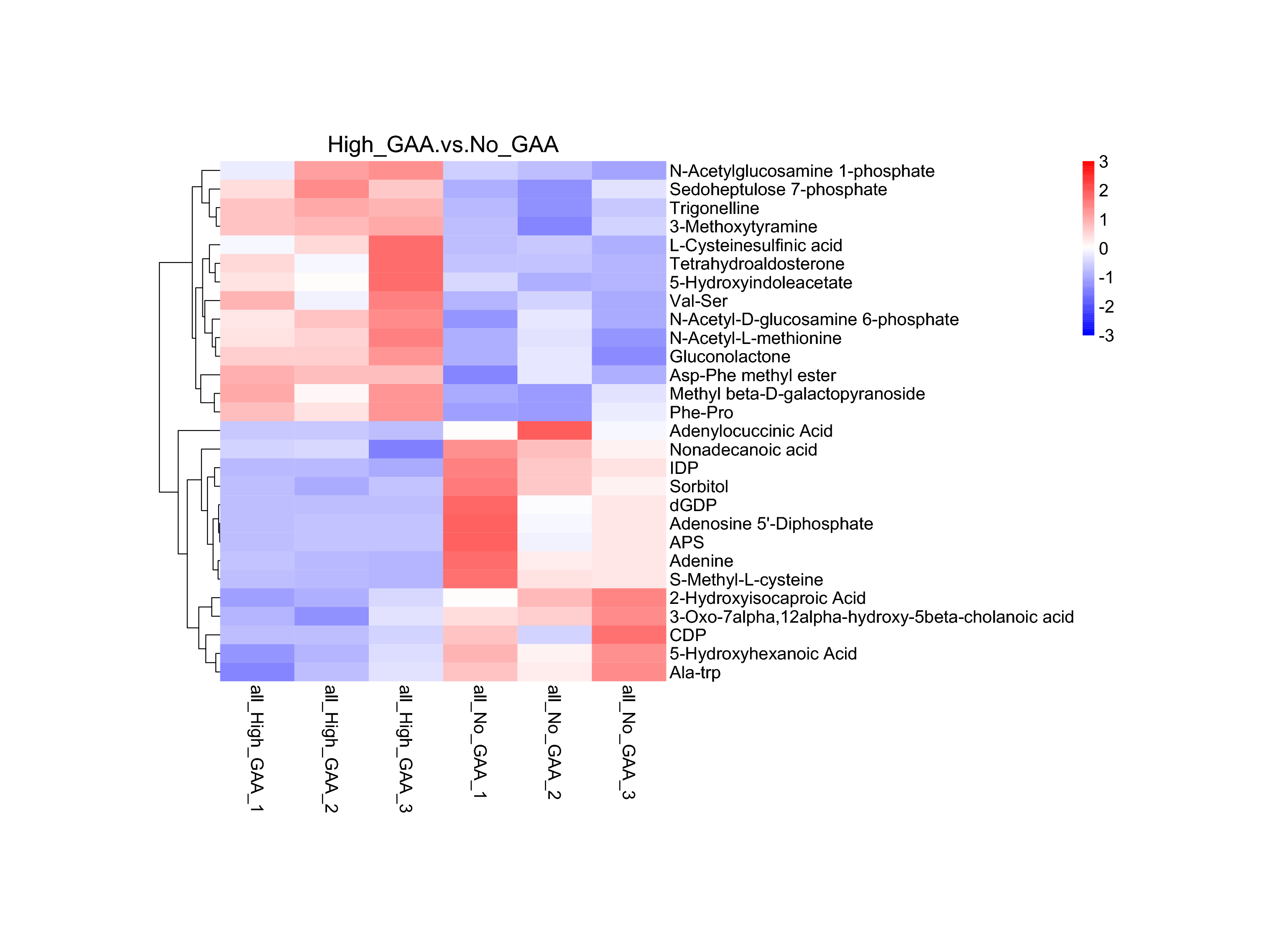

Supplement: Supplementary file 2 [file Data_Sheet_1.ZIP › Result-X101SC22030966-Z01-J001-B1-42 (quasi-targeted metabolomics)/4.MetDiffAnalysis/High_GAA.vs.No_GAA/High_GAA.vs.No_GAA_all_heatmap_detail.png]

High\_GAA.vs.No\_GAA

Metabolites

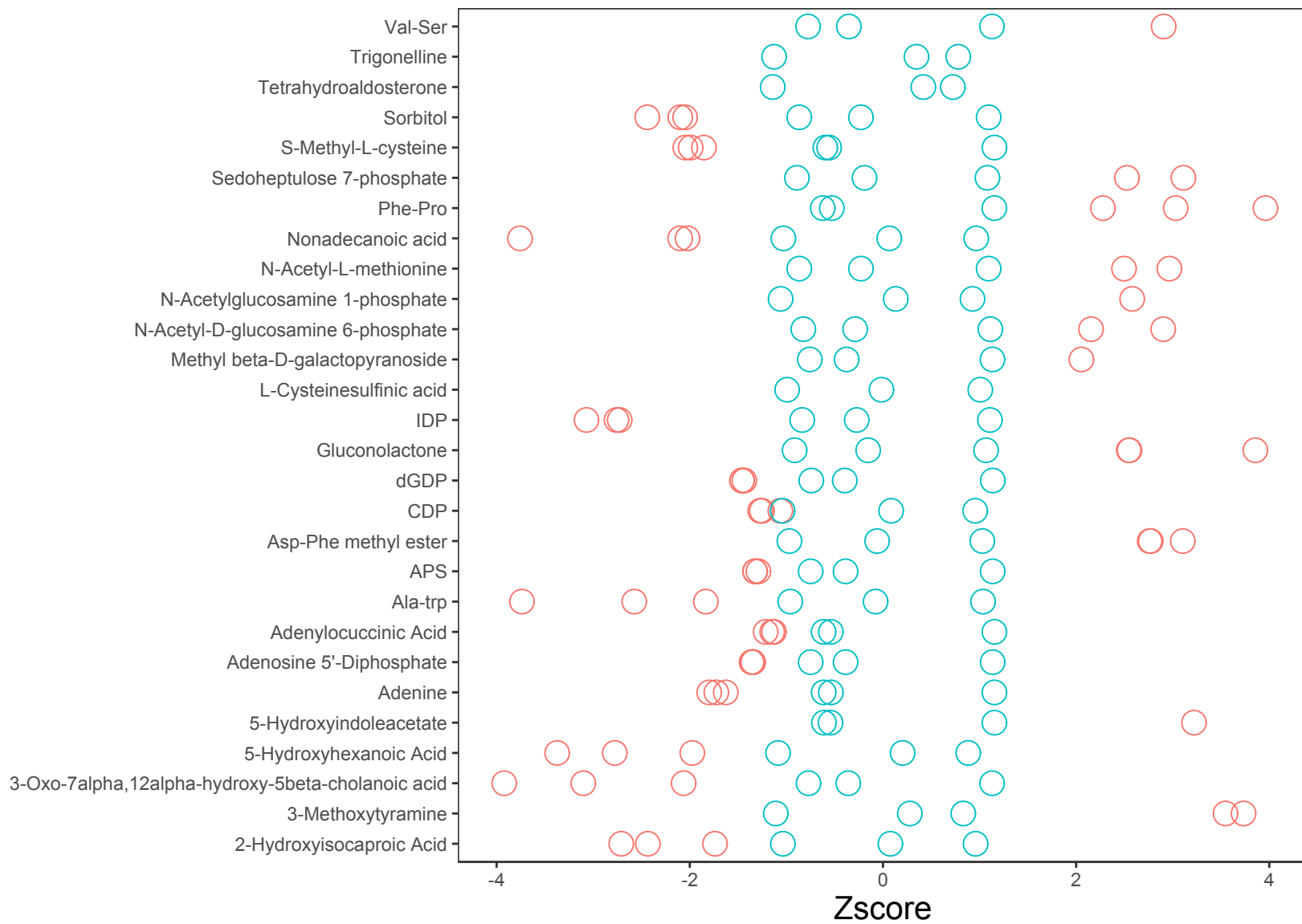

Supplement: Supplementary file 2 [file Data_Sheet_1.ZIP › Result-X101SC22030966-Z01-J001-B1-42 (quasi-targeted metabolomics)/4.MetDiffAnalysis/High_GAA.vs.No_GAA/High_GAA.vs.No_GAA_all_zscore.pdf]

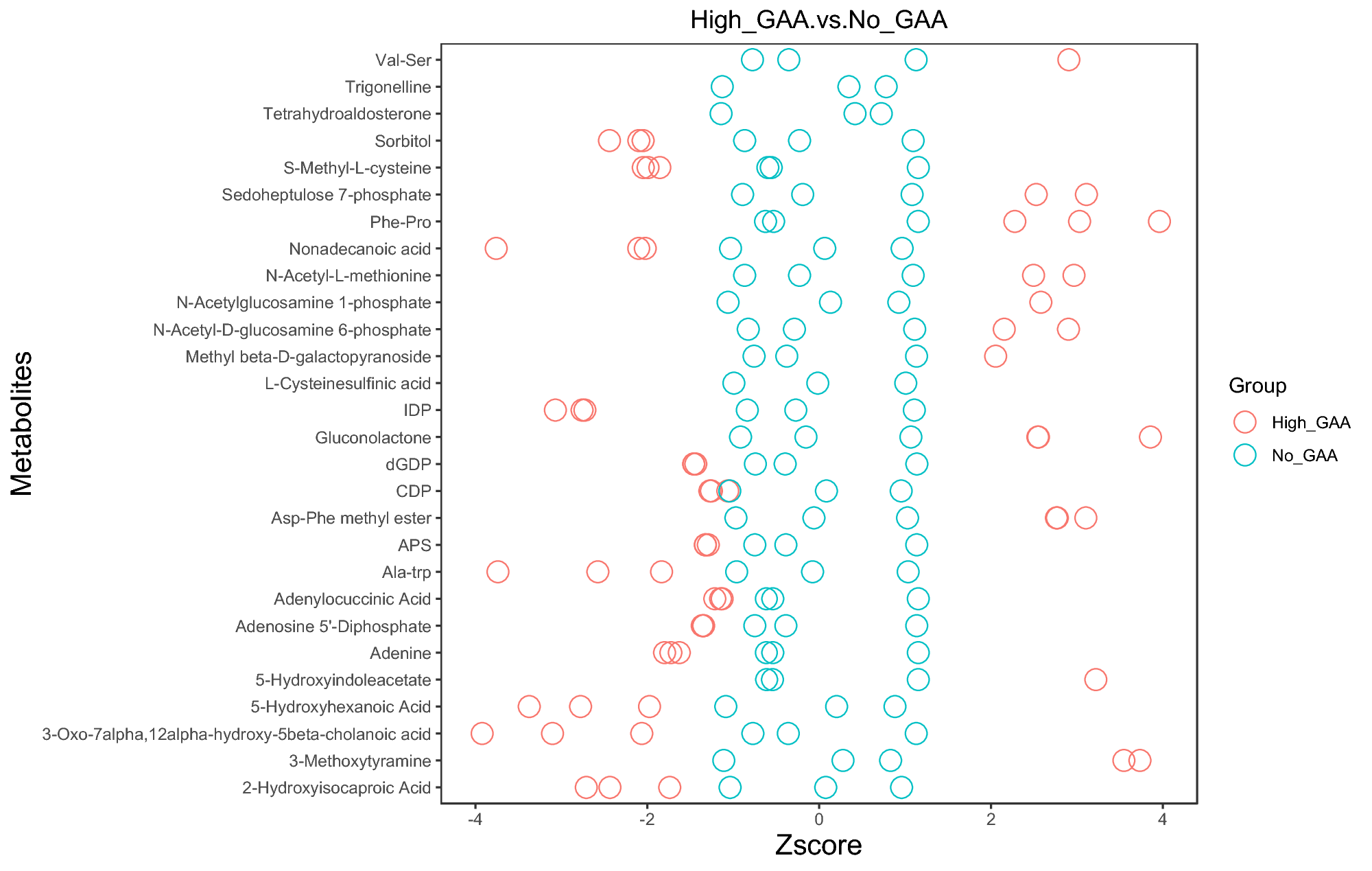

Supplement: Supplementary file 2 [file Data_Sheet_1.ZIP › Result-X101SC22030966-Z01-J001-B1-42 (quasi-targeted metabolomics)/4.MetDiffAnalysis/High_GAA.vs.No_GAA/High_GAA.vs.No_GAA_all_zscore.png]

High\_GAA.vs.No\_GAA

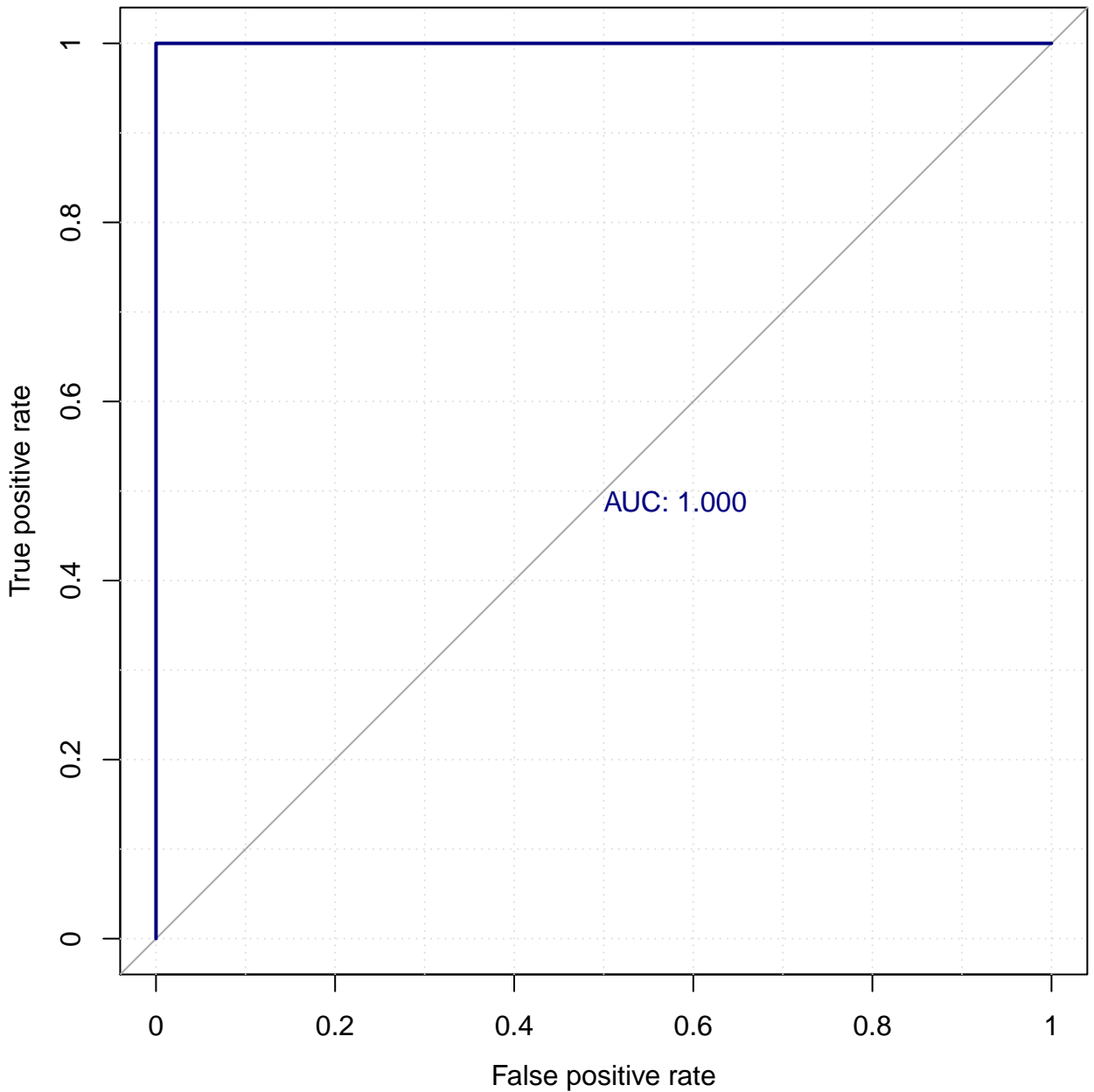

Supplement: Supplementary file 2 [file Data_Sheet_1.ZIP › Result-X101SC22030966-Z01-J001-B1-42 (quasi-targeted metabolomics)/4.MetDiffAnalysis/High_GAA.vs.No_GAA/ROC_all/Com_102_neg_ROC.pdf]

High\_GAA.vs.No\_GAA

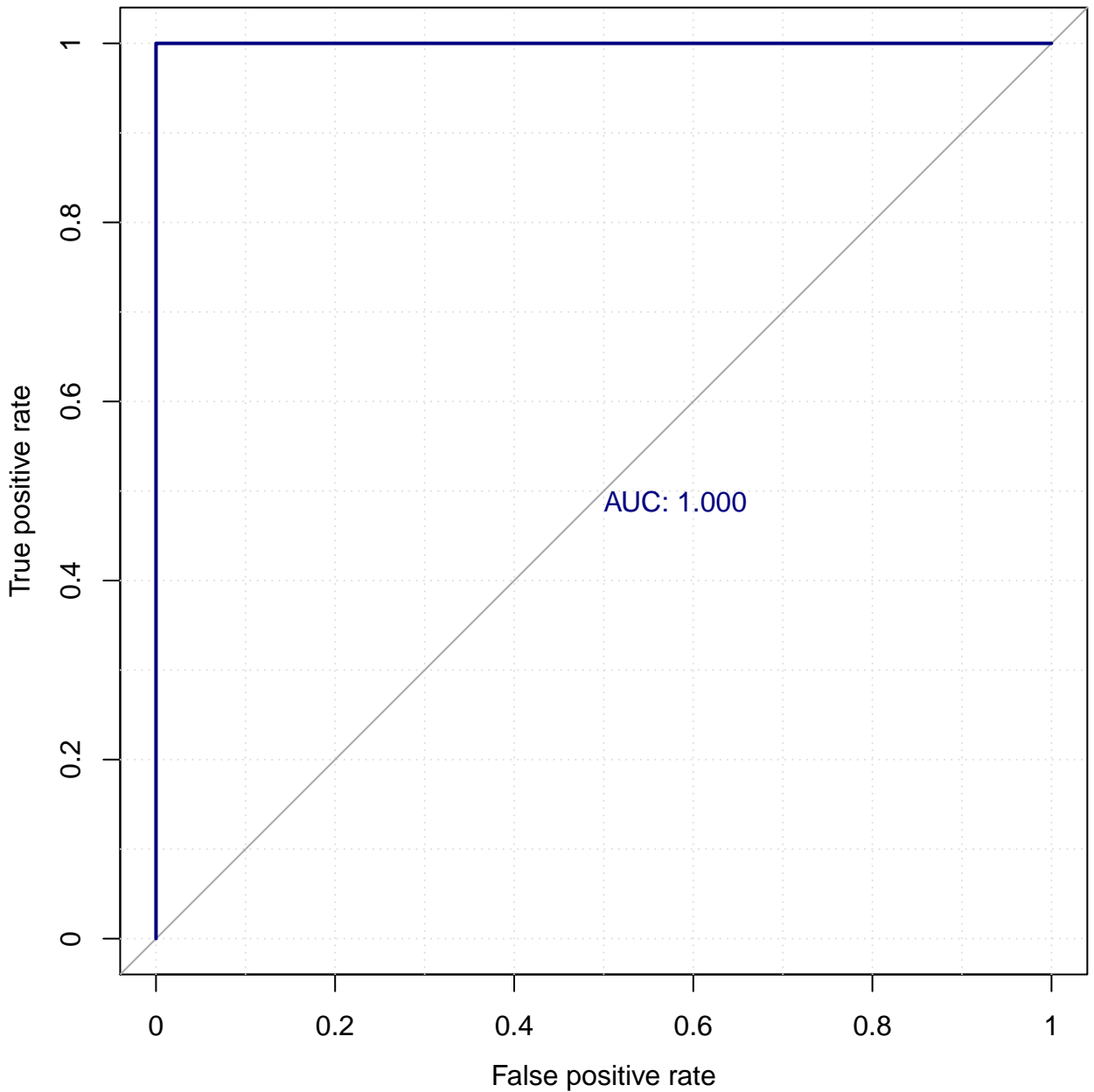

Supplement: Supplementary file 2 [file Data_Sheet_1.ZIP › Result-X101SC22030966-Z01-J001-B1-42 (quasi-targeted metabolomics)/4.MetDiffAnalysis/High_GAA.vs.No_GAA/ROC_all/Com_159_neg_ROC.pdf]

High\_GAA.vs.No\_GAA

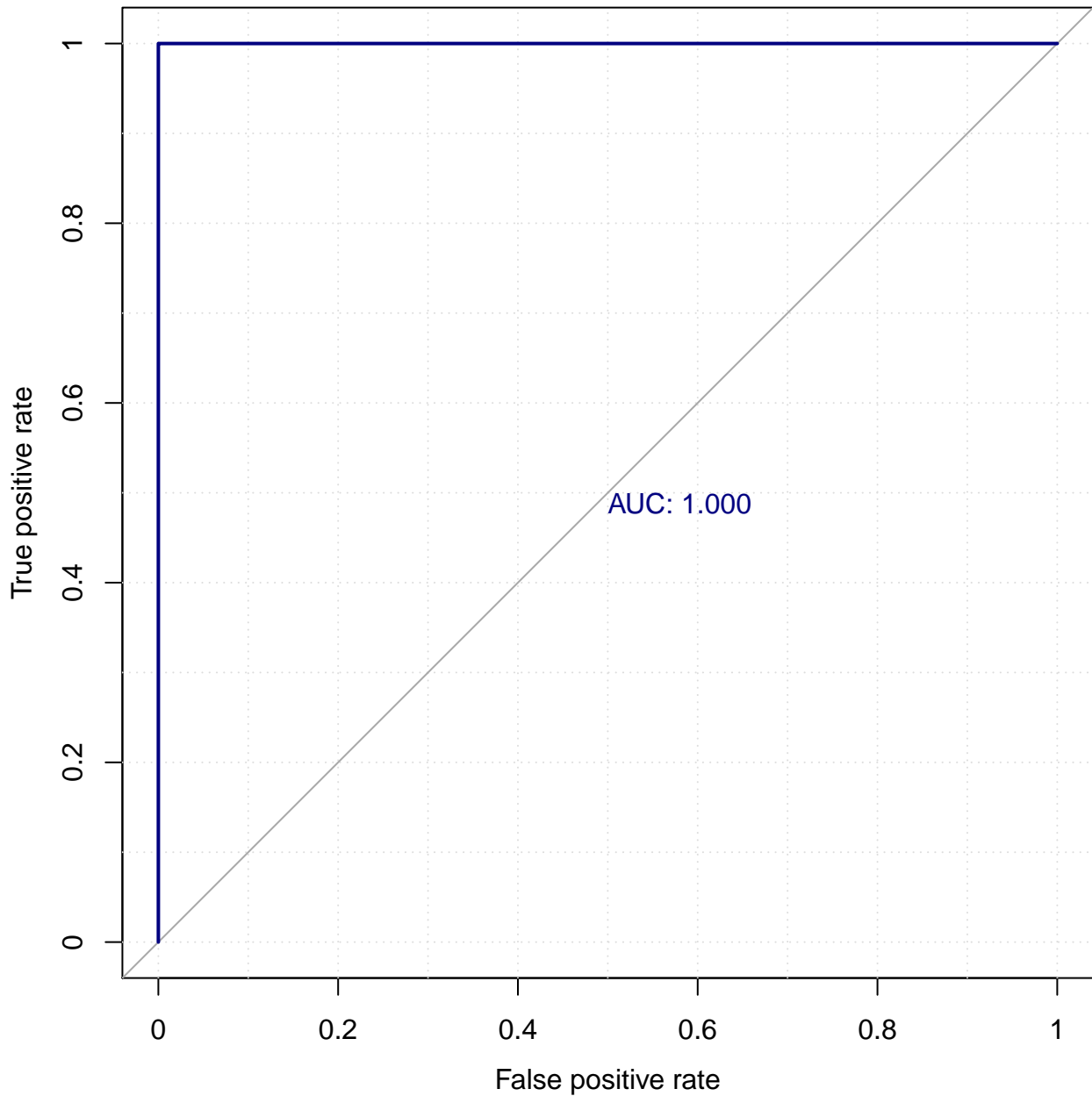

Supplement: Supplementary file 2 [file Data_Sheet_1.ZIP › Result-X101SC22030966-Z01-J001-B1-42 (quasi-targeted metabolomics)/4.MetDiffAnalysis/High_GAA.vs.No_GAA/ROC_all/Com_173_neg_ROC.pdf]

High\_GAA.vs.No\_GAA

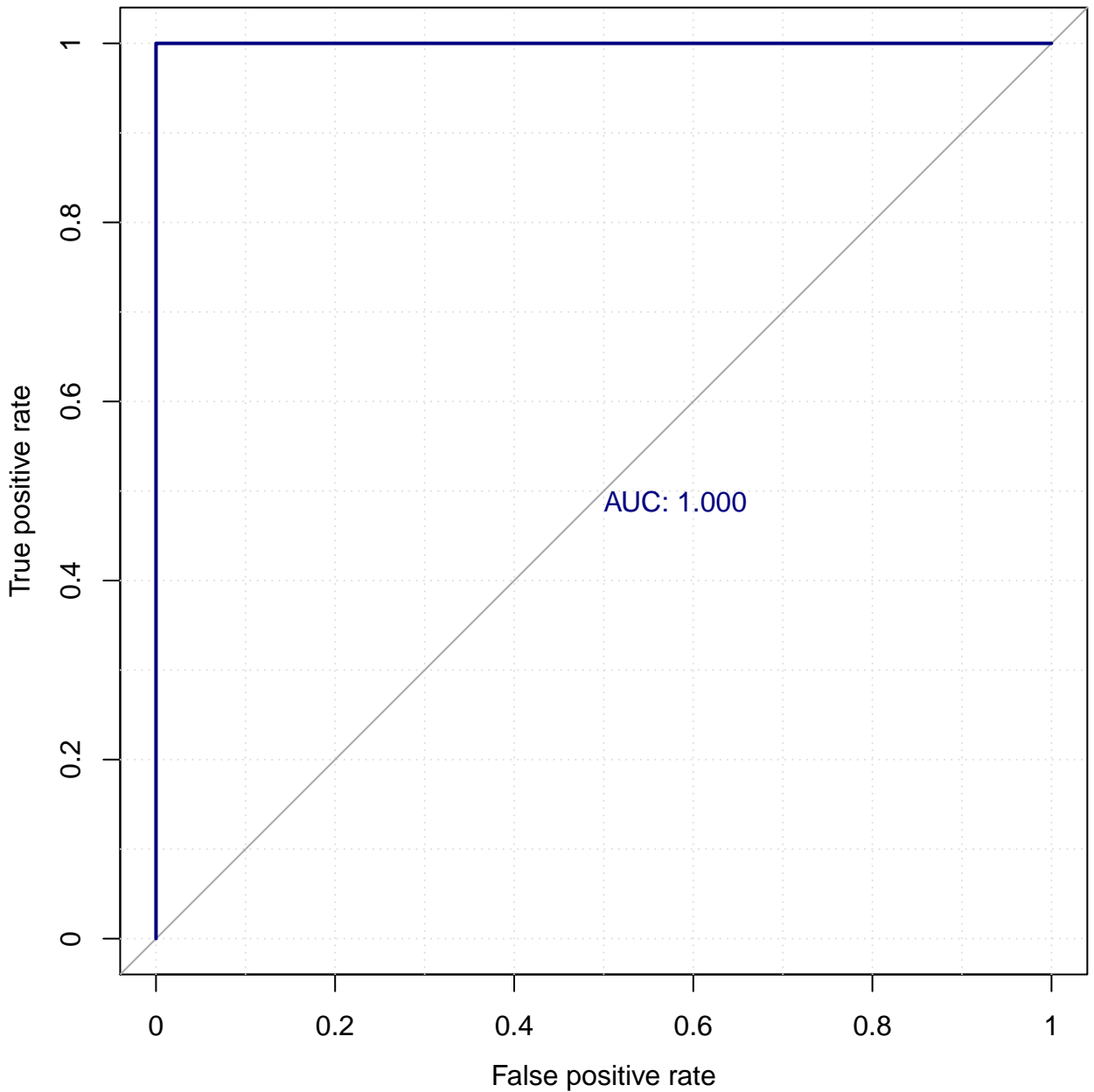

Supplement: Supplementary file 2 [file Data_Sheet_1.ZIP › Result-X101SC22030966-Z01-J001-B1-42 (quasi-targeted metabolomics)/4.MetDiffAnalysis/High_GAA.vs.No_GAA/ROC_all/Com_242_neg_ROC.pdf]

High\_GAA.vs.No\_GAA

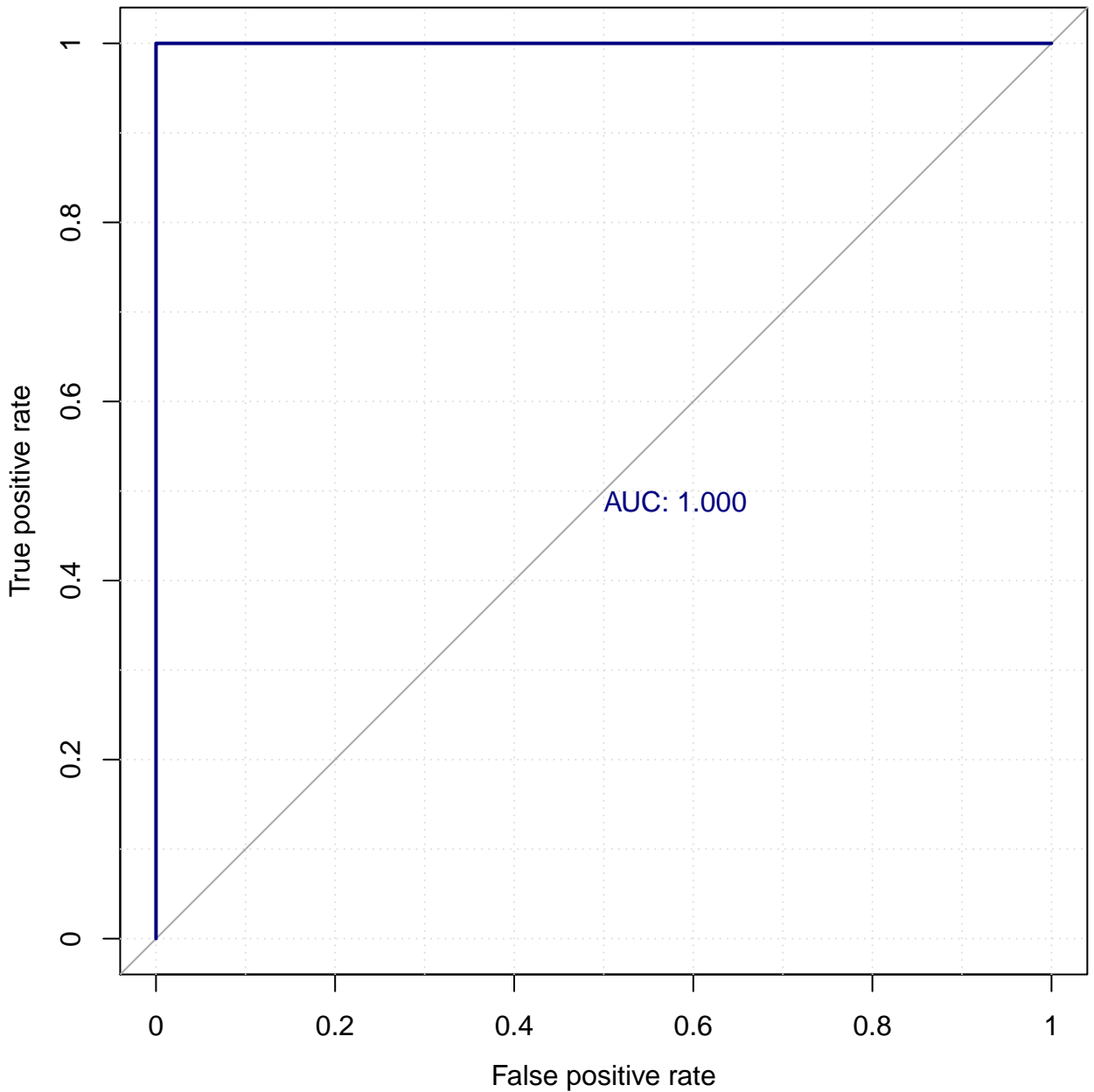

Supplement: Supplementary file 2 [file Data_Sheet_1.ZIP › Result-X101SC22030966-Z01-J001-B1-42 (quasi-targeted metabolomics)/4.MetDiffAnalysis/High_GAA.vs.No_GAA/ROC_all/Com_296_neg_ROC.pdf]

High\_GAA.vs.No\_GAA

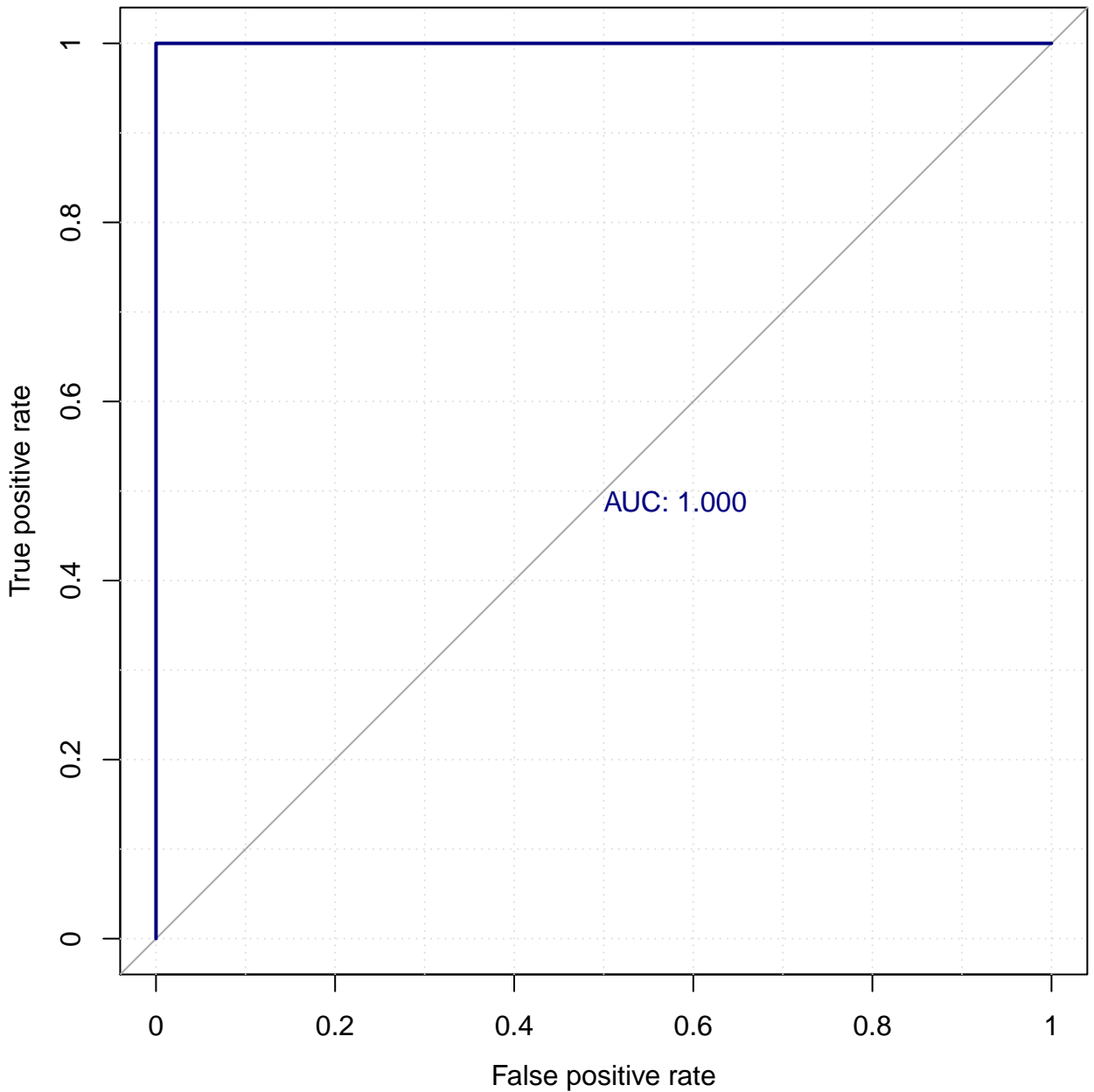

Supplement: Supplementary file 2 [file Data_Sheet_1.ZIP › Result-X101SC22030966-Z01-J001-B1-42 (quasi-targeted metabolomics)/4.MetDiffAnalysis/High_GAA.vs.No_GAA/ROC_all/Com_392_pos_ROC.pdf]

High\_GAA.vs.No\_GAA

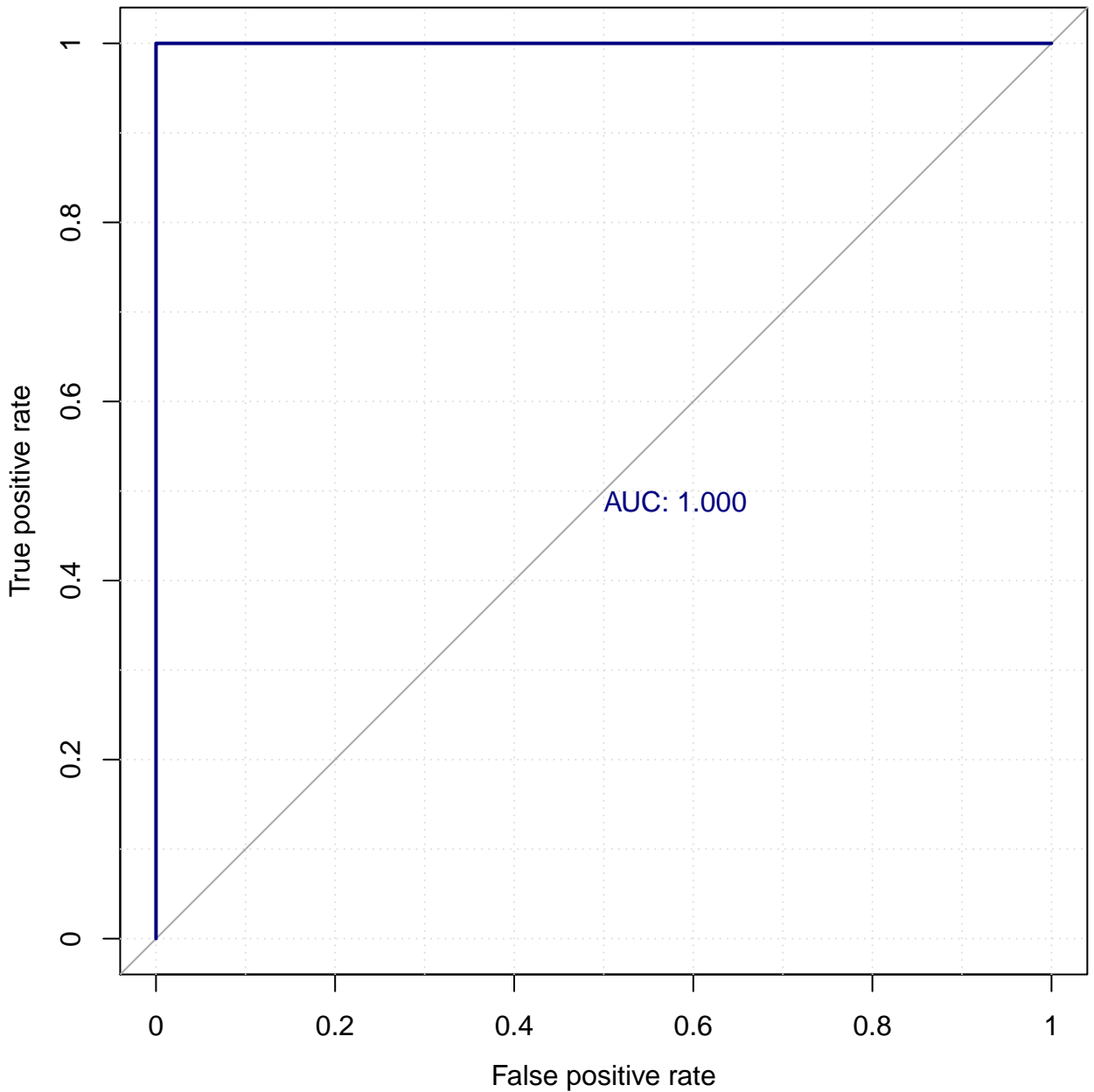

Supplement: Supplementary file 2 [file Data_Sheet_1.ZIP › Result-X101SC22030966-Z01-J001-B1-42 (quasi-targeted metabolomics)/4.MetDiffAnalysis/High_GAA.vs.No_GAA/ROC_all/Com_537_pos_ROC.pdf]

High\_GAA.vs.No\_GAA

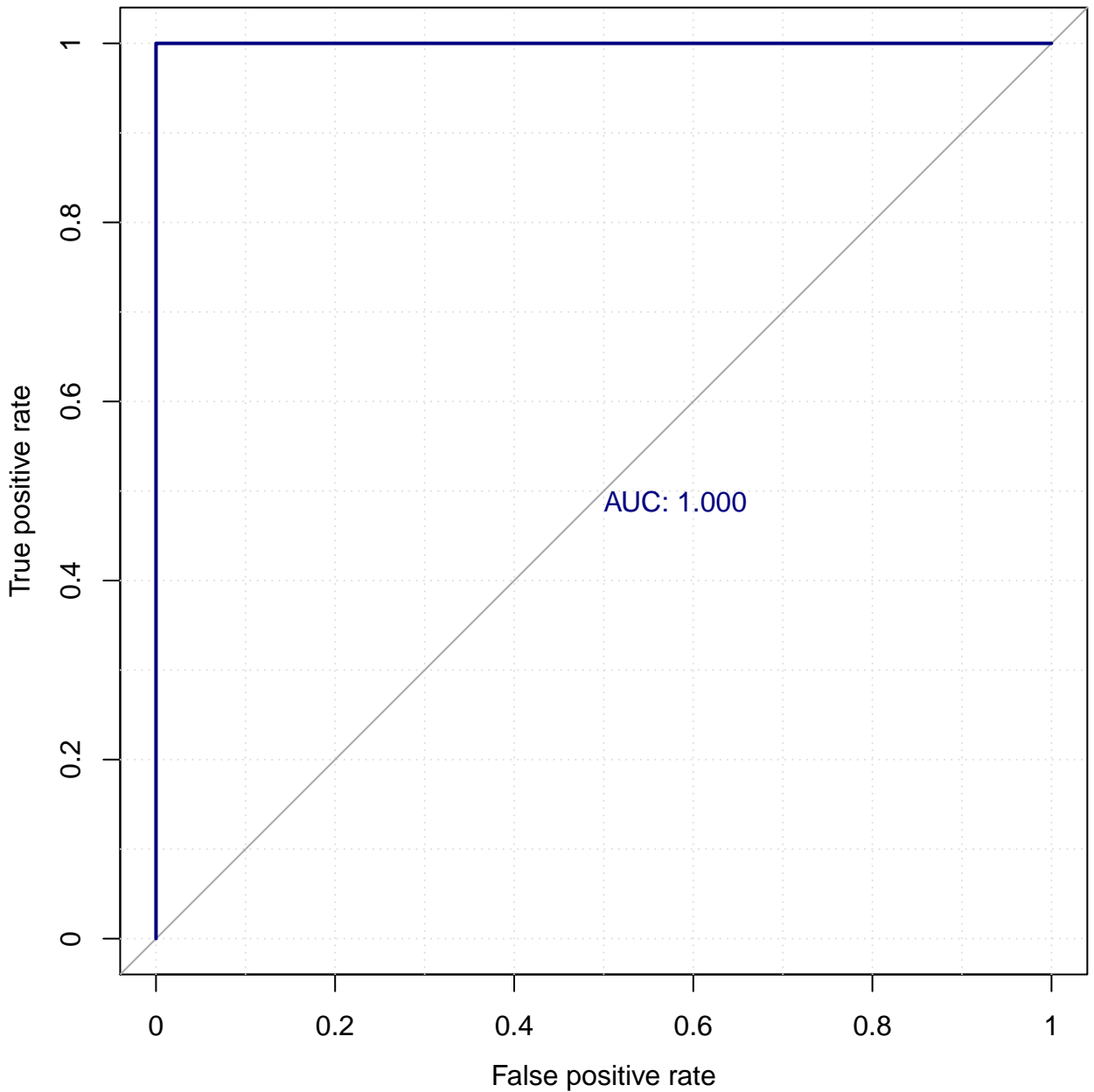

Supplement: Supplementary file 2 [file Data_Sheet_1.ZIP › Result-X101SC22030966-Z01-J001-B1-42 (quasi-targeted metabolomics)/4.MetDiffAnalysis/High_GAA.vs.No_GAA/ROC_all/Com_646_pos_ROC.pdf]
