# Supplementary figures and images for: Association analysis of transcriptome and quasi-targeted metabolomics reveals the regulation mechanism underlying broiler muscle tissue development at different levels of dietary guanidinoacetic acid (part 1 of 2)
Source: Front Vet Sci. 2024 Apr 25;11:1384028. doi: 10.3389/fvets.2024.1384028 (PMC11080945; doi:10.3389/fvets.2024.1384028)

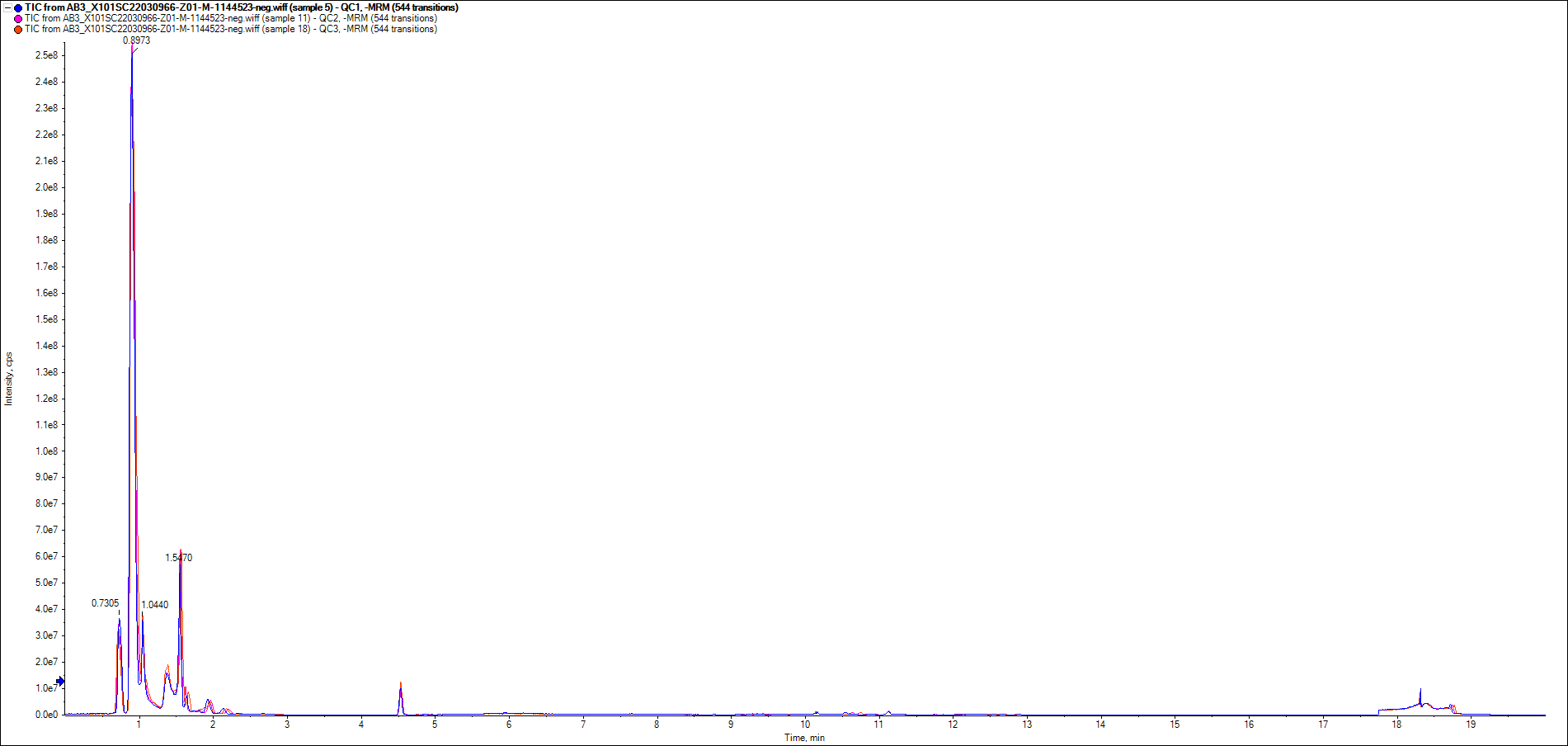

Supplement: Supplementary file 2 [file Data_Sheet_1.ZIP › Result-X101SC22030966-Z01-J001-B1-42 (quasi-targeted metabolomics)/1.MetQuant-QC/QC_TIC_neg.png]

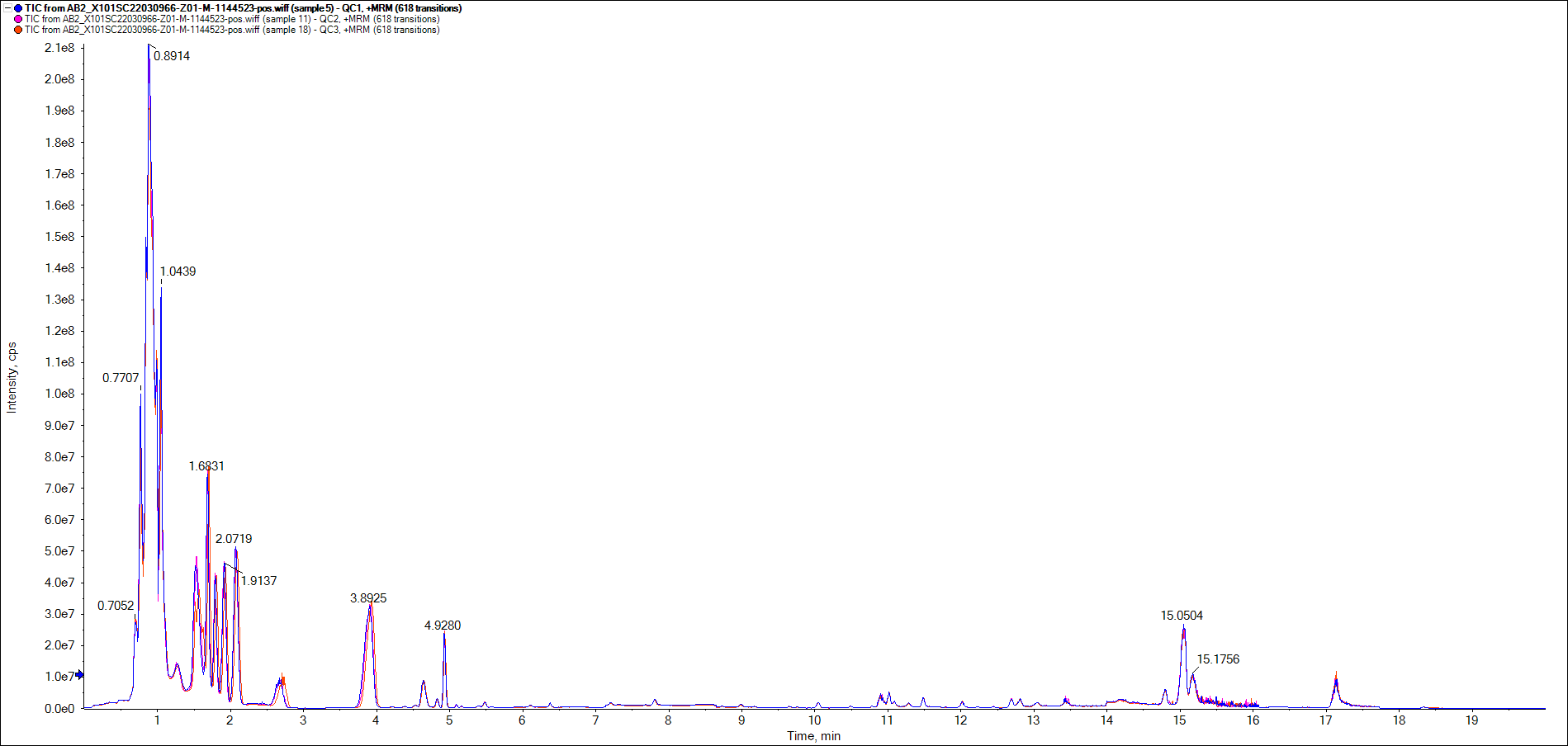

Supplement: Supplementary file 2 [file Data_Sheet_1.ZIP › Result-X101SC22030966-Z01-J001-B1-42 (quasi-targeted metabolomics)/1.MetQuant-QC/QC_TIC_pos.png]

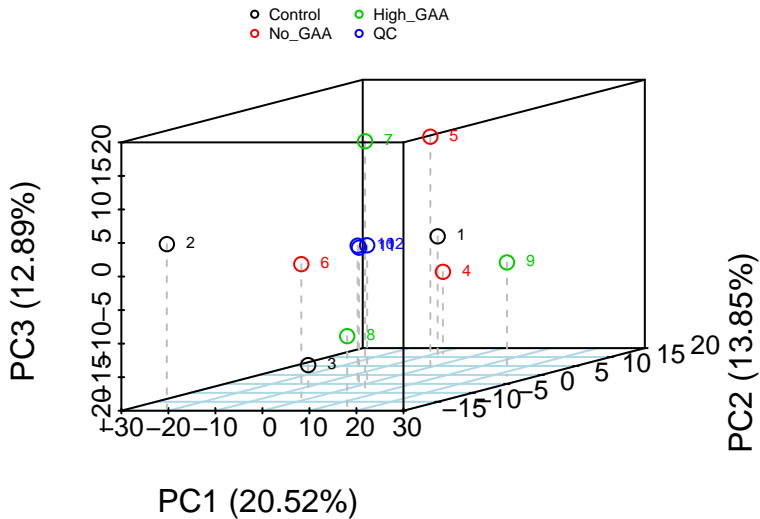

Supplement: Supplementary file 2 [file Data_Sheet_1.ZIP › Result-X101SC22030966-Z01-J001-B1-42 (quasi-targeted metabolomics)/1.MetQuant-QC/Samples_QC_all-PCA.3D.pdf]

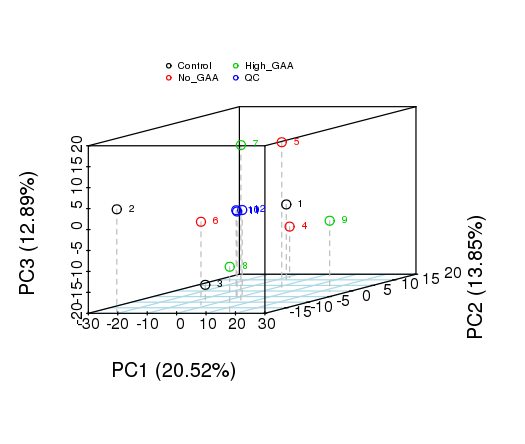

Supplement: Supplementary file 2 [file Data_Sheet_1.ZIP › Result-X101SC22030966-Z01-J001-B1-42 (quasi-targeted metabolomics)/1.MetQuant-QC/Samples_QC_all-PCA.3D.png]

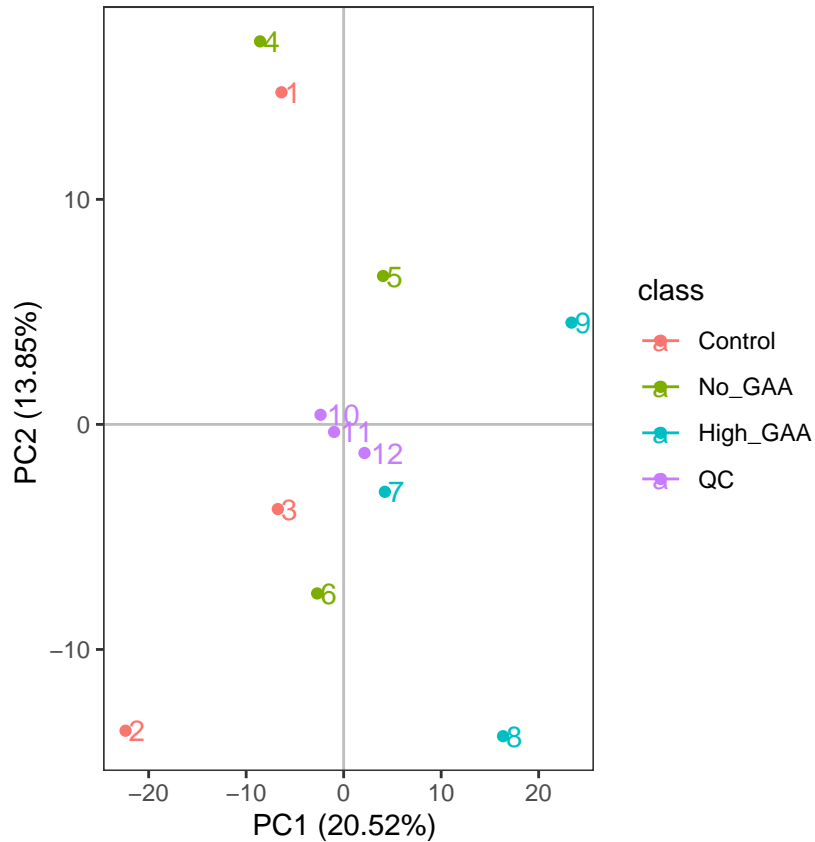

Supplement: Supplementary file 2 [file Data_Sheet_1.ZIP › Result-X101SC22030966-Z01-J001-B1-42 (quasi-targeted metabolomics)/1.MetQuant-QC/Samples_QC_all-PCA.pdf]

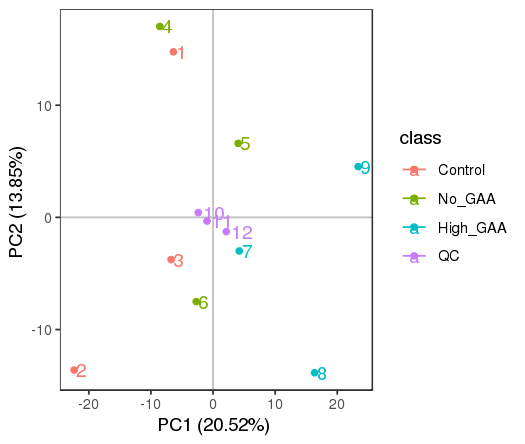

Supplement: Supplementary file 2 [file Data_Sheet_1.ZIP › Result-X101SC22030966-Z01-J001-B1-42 (quasi-targeted metabolomics)/1.MetQuant-QC/Samples_QC_all-PCA.png]

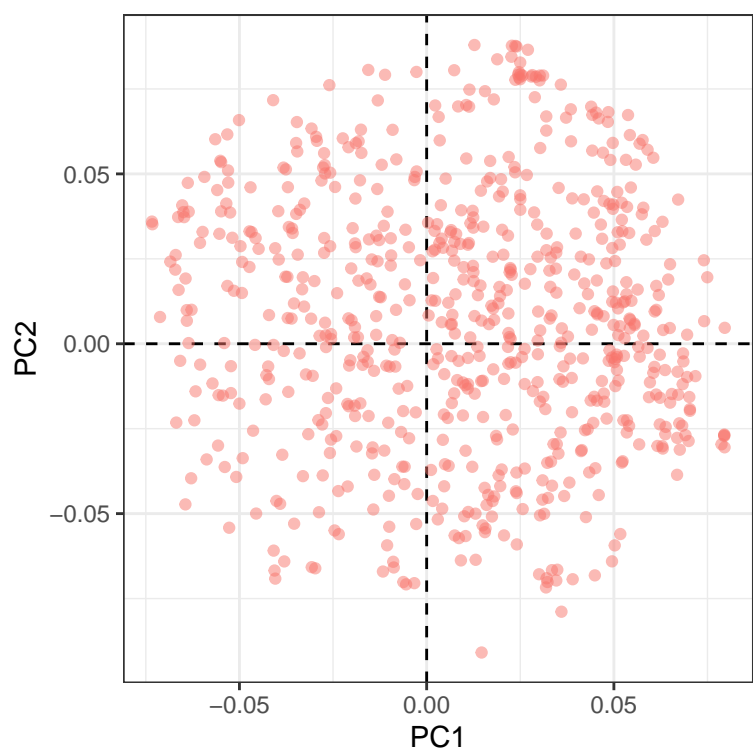

Supplement: Supplementary file 2 [file Data_Sheet_1.ZIP › Result-X101SC22030966-Z01-J001-B1-42 (quasi-targeted metabolomics)/1.MetQuant-QC/Samples_QC_all-pcaloading.pdf]

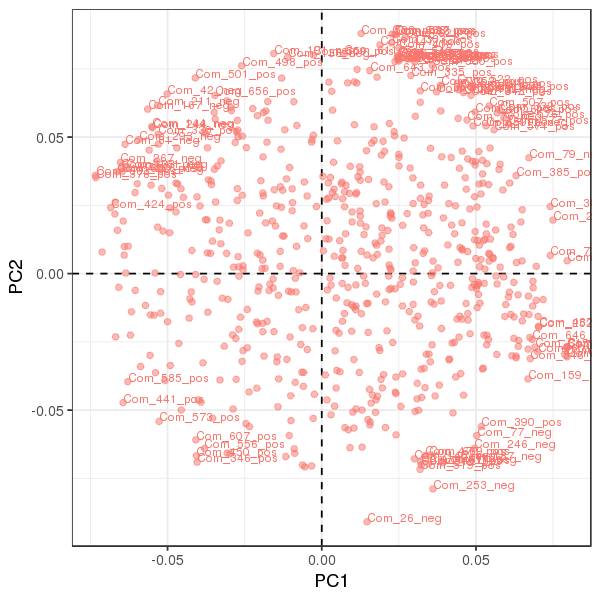

Supplement: Supplementary file 2 [file Data_Sheet_1.ZIP › Result-X101SC22030966-Z01-J001-B1-42 (quasi-targeted metabolomics)/1.MetQuant-QC/Samples_QC_all-pcaloading.png]

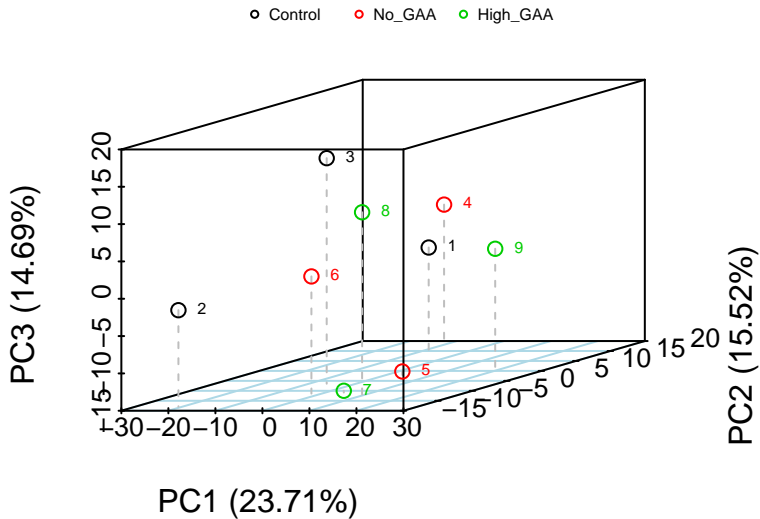

Supplement: Supplementary file 2 [file Data_Sheet_1.ZIP › Result-X101SC22030966-Z01-J001-B1-42 (quasi-targeted metabolomics)/1.MetQuant-QC/Samples_all-PCA.3D.pdf]

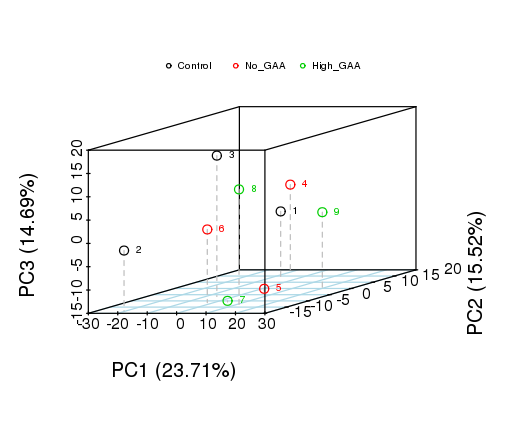

Supplement: Supplementary file 2 [file Data_Sheet_1.ZIP › Result-X101SC22030966-Z01-J001-B1-42 (quasi-targeted metabolomics)/1.MetQuant-QC/Samples_all-PCA.3D.png]

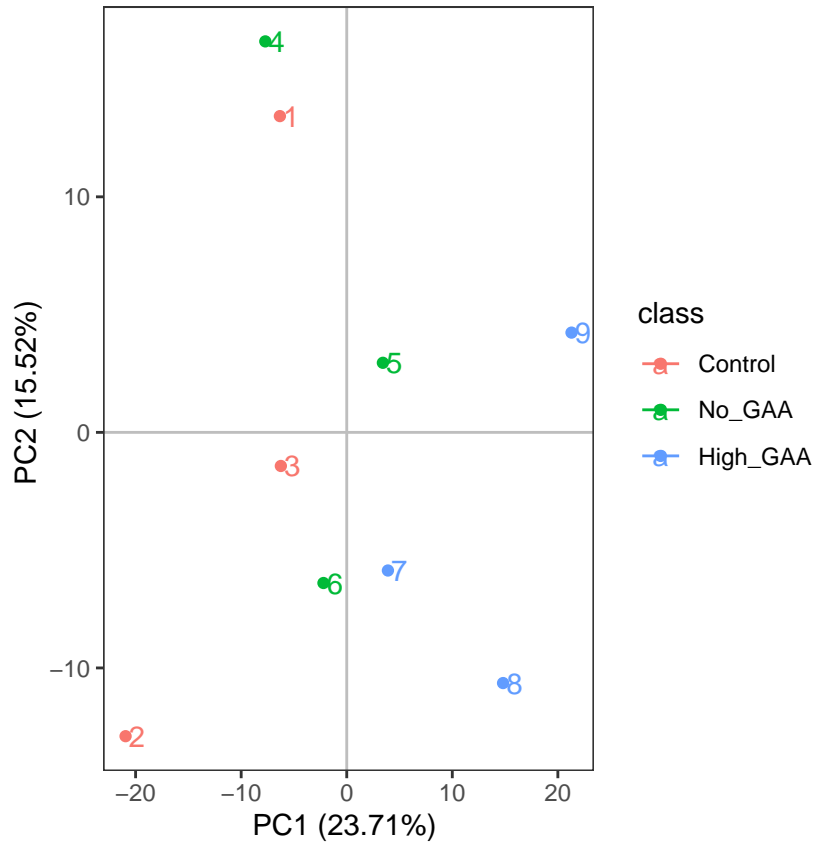

Supplement: Supplementary file 2 [file Data_Sheet_1.ZIP › Result-X101SC22030966-Z01-J001-B1-42 (quasi-targeted metabolomics)/1.MetQuant-QC/Samples_all-PCA.pdf]

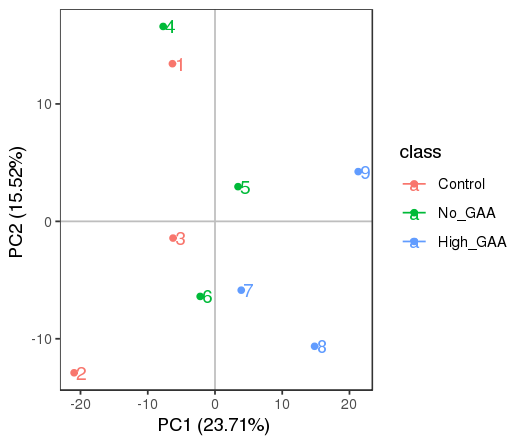

Supplement: Supplementary file 2 [file Data_Sheet_1.ZIP › Result-X101SC22030966-Z01-J001-B1-42 (quasi-targeted metabolomics)/1.MetQuant-QC/Samples_all-PCA.png]

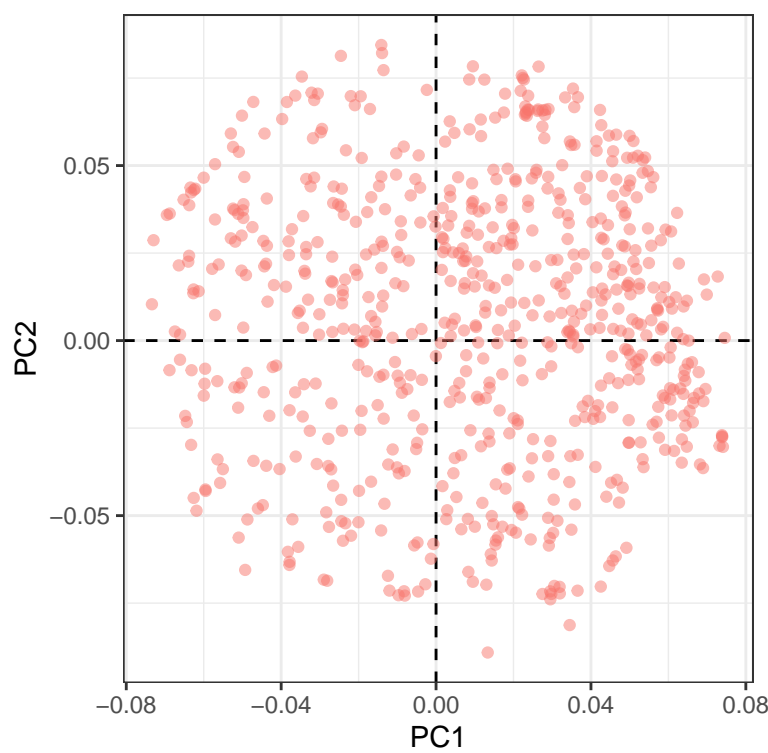

Supplement: Supplementary file 2 [file Data_Sheet_1.ZIP › Result-X101SC22030966-Z01-J001-B1-42 (quasi-targeted metabolomics)/1.MetQuant-QC/Samples_all-pcaloading.pdf]

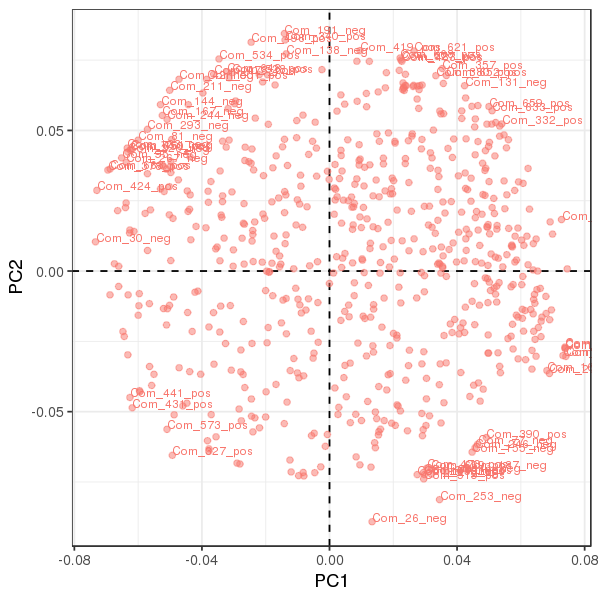

Supplement: Supplementary file 2 [file Data_Sheet_1.ZIP › Result-X101SC22030966-Z01-J001-B1-42 (quasi-targeted metabolomics)/1.MetQuant-QC/Samples_all-pcaloading.png]

Pearson correlation between all QC samples

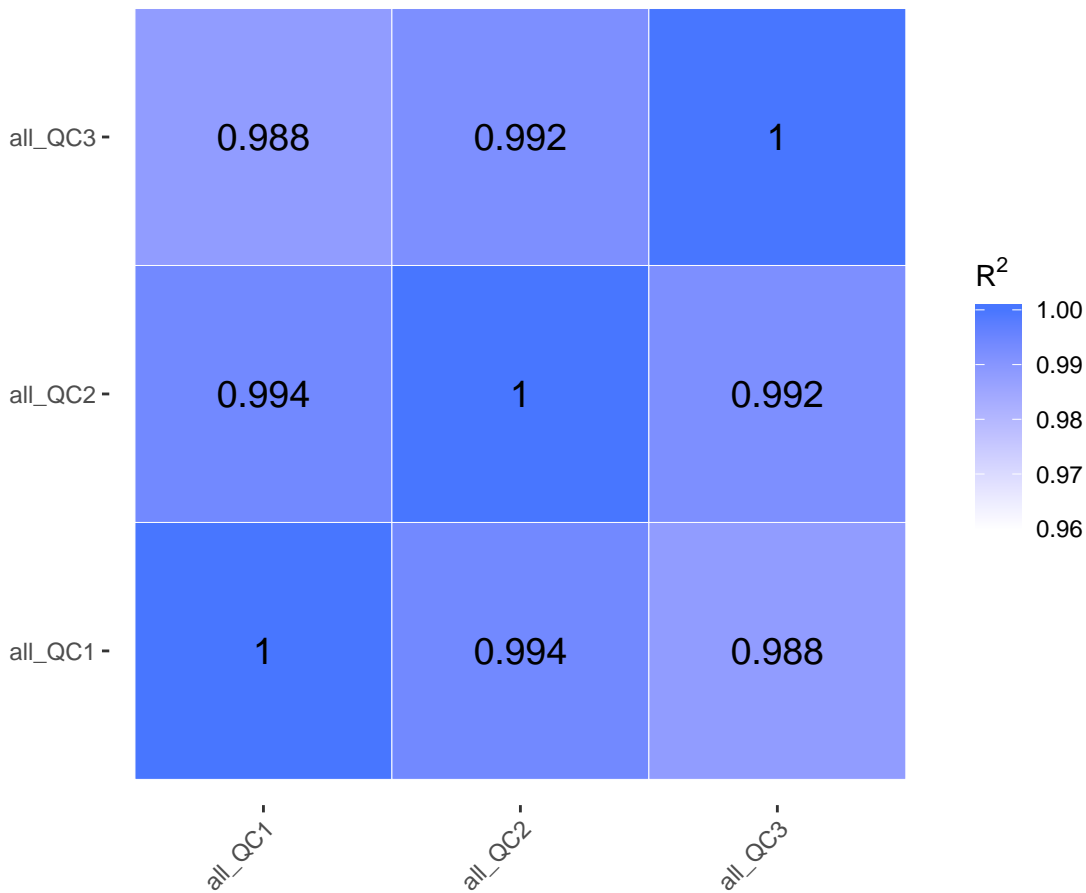

Supplement: Supplementary file 2 [file Data_Sheet_1.ZIP › Result-X101SC22030966-Z01-J001-B1-42 (quasi-targeted metabolomics)/1.MetQuant-QC/cor_pearson_all.pdf]

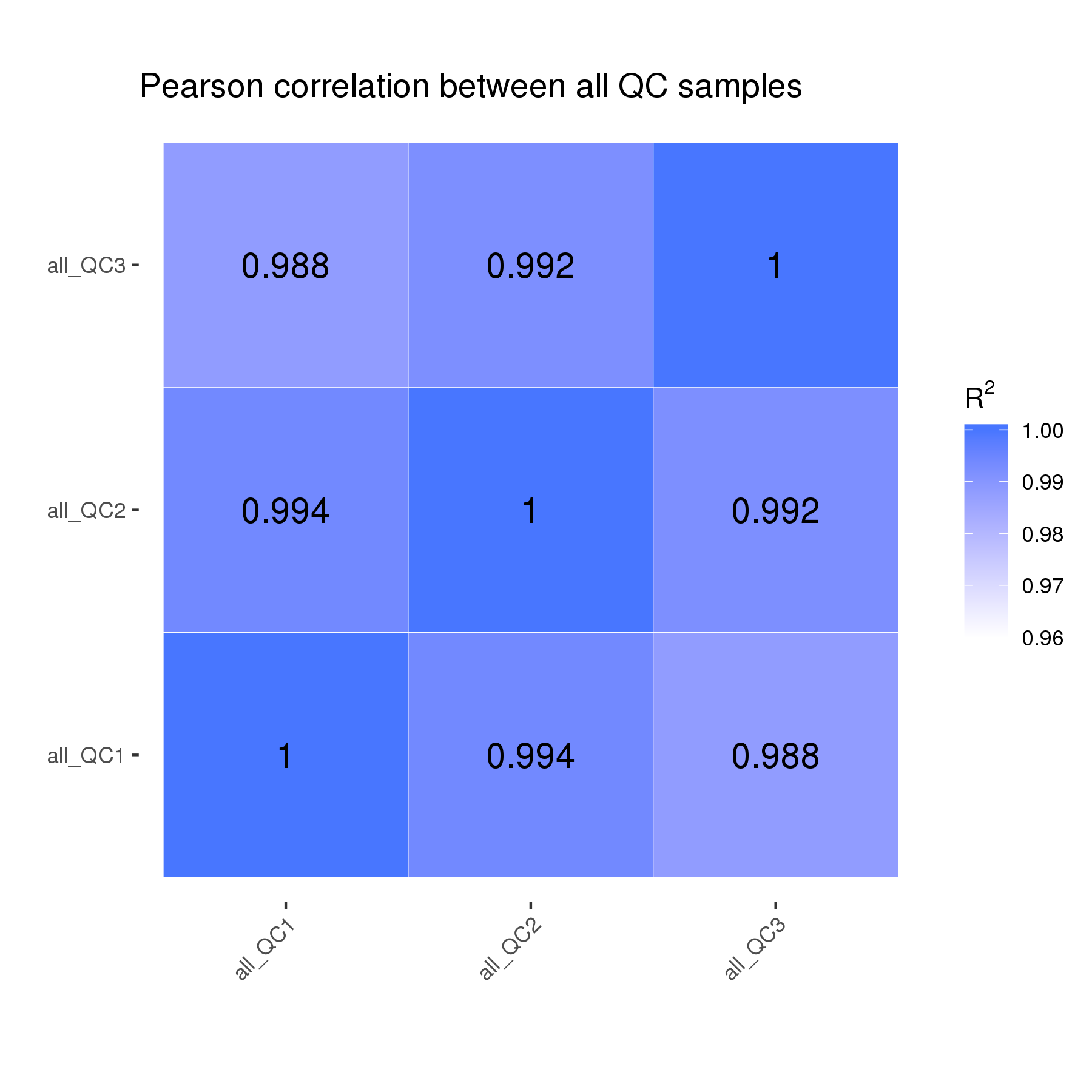

Supplement: Supplementary file 2 [file Data_Sheet_1.ZIP › Result-X101SC22030966-Z01-J001-B1-42 (quasi-targeted metabolomics)/1.MetQuant-QC/cor_pearson_all.png]

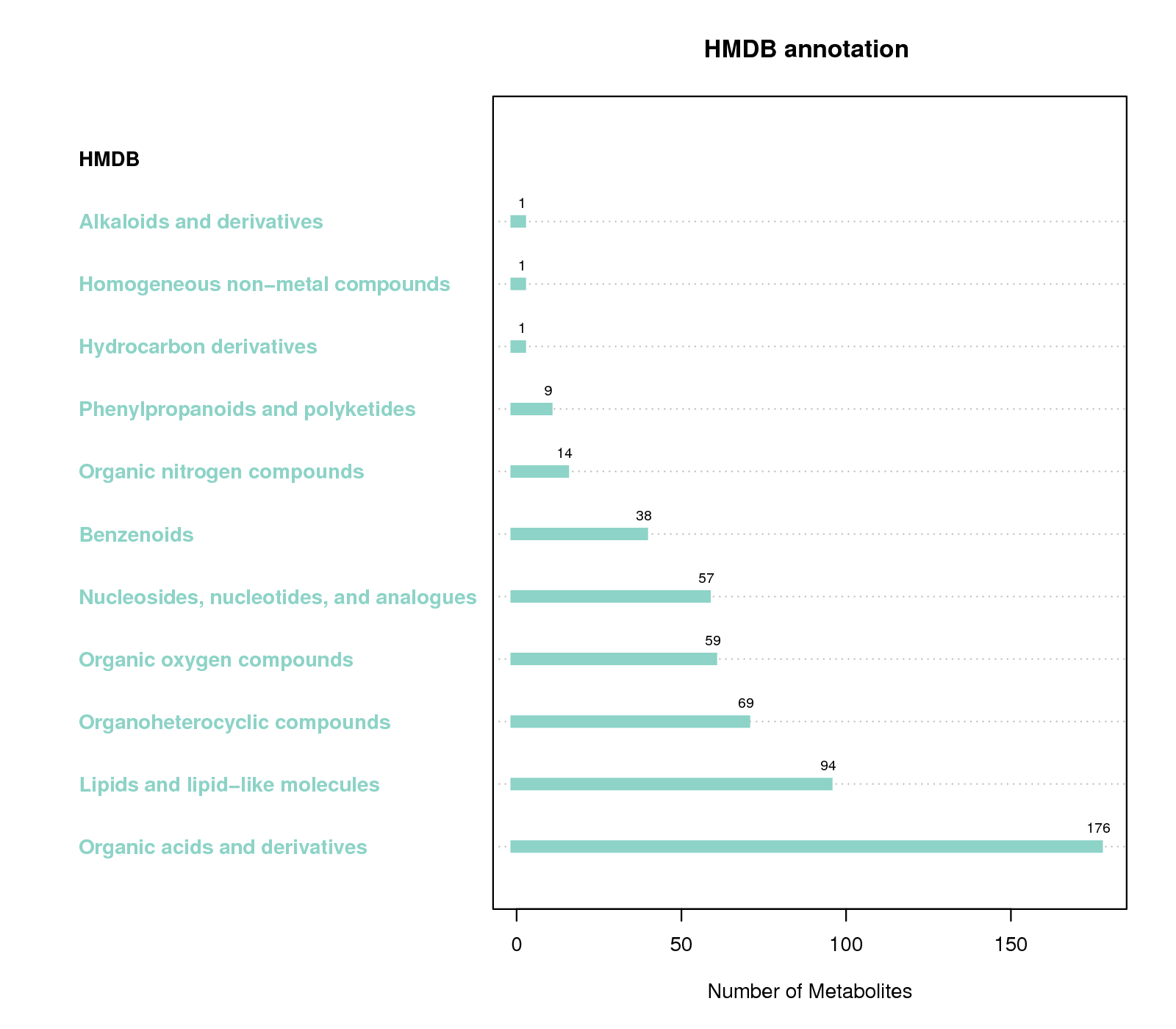

Supplement: Supplementary file 2 [file Data_Sheet_1.ZIP › Result-X101SC22030966-Z01-J001-B1-42 (quasi-targeted metabolomics)/2.MetAnnotation/HMDB/meta_all.HMDB.Anno.png]

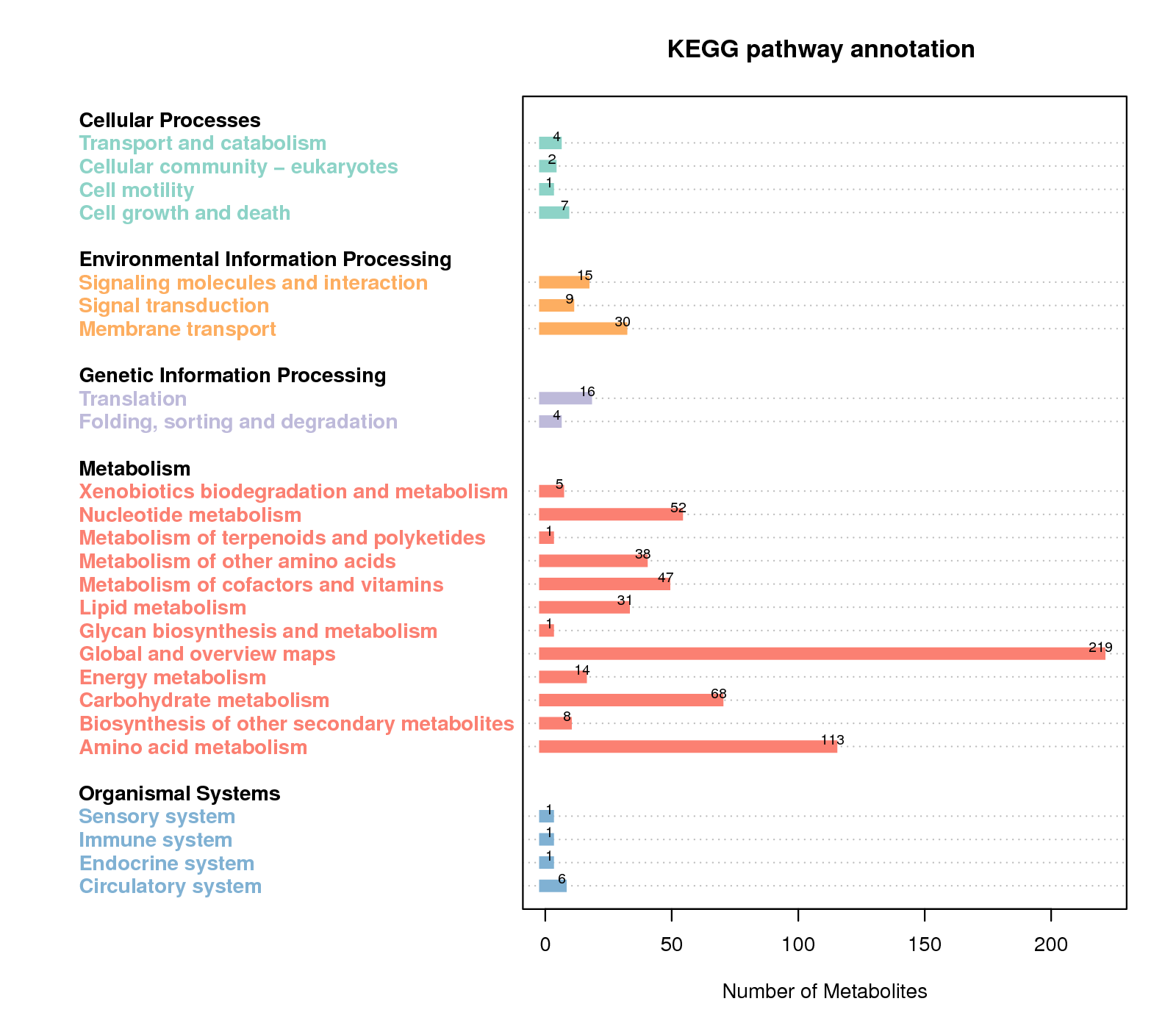

Supplement: Supplementary file 2 [file Data_Sheet_1.ZIP › Result-X101SC22030966-Z01-J001-B1-42 (quasi-targeted metabolomics)/2.MetAnnotation/KEGG/meta_all.KEGG.Anno.png]

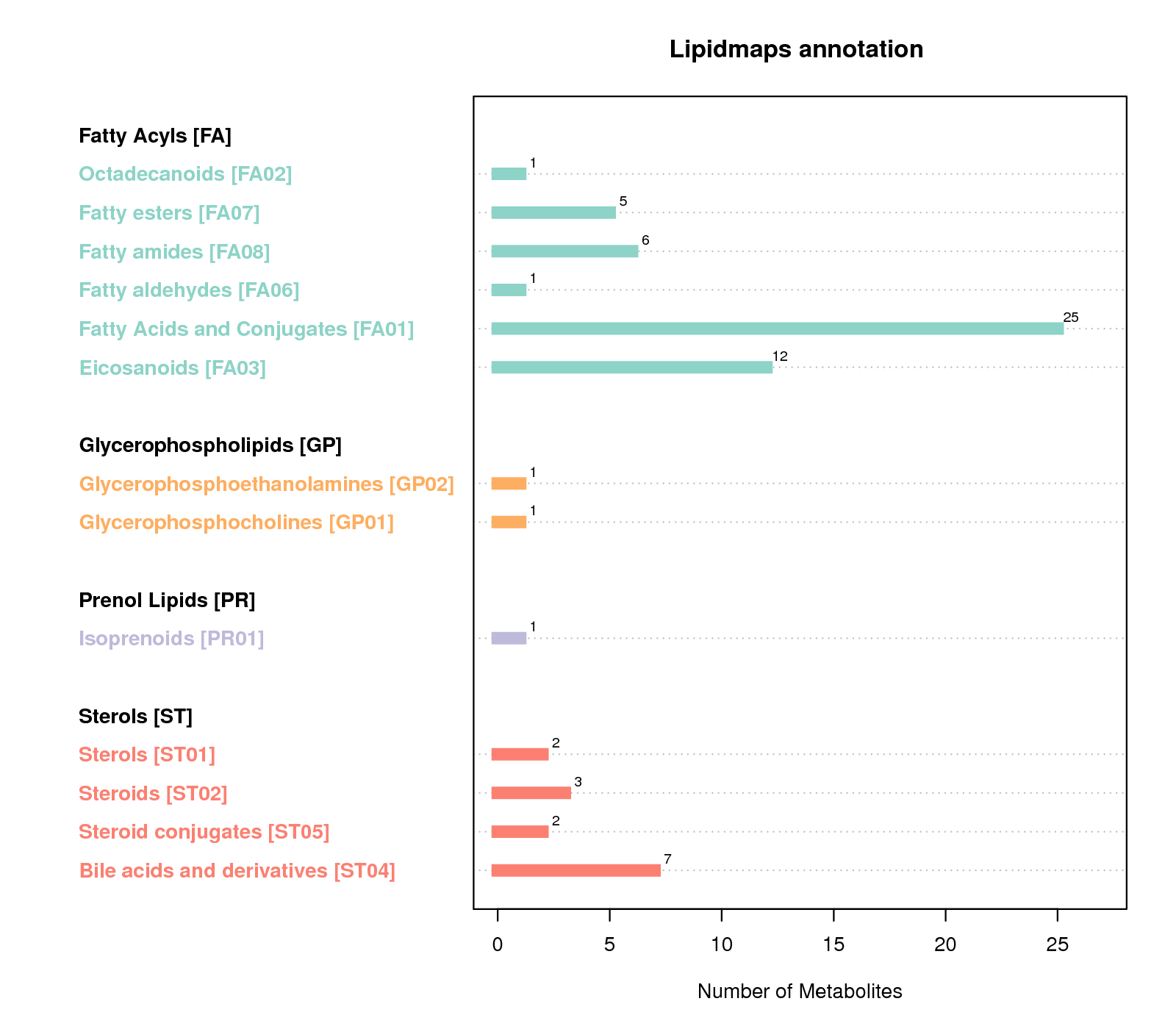

Supplement: Supplementary file 2 [file Data_Sheet_1.ZIP › Result-X101SC22030966-Z01-J001-B1-42 (quasi-targeted metabolomics)/2.MetAnnotation/Lipidmaps/meta_all.Lipidmaps.Anno.png]

# High\_GAA.vs.Control

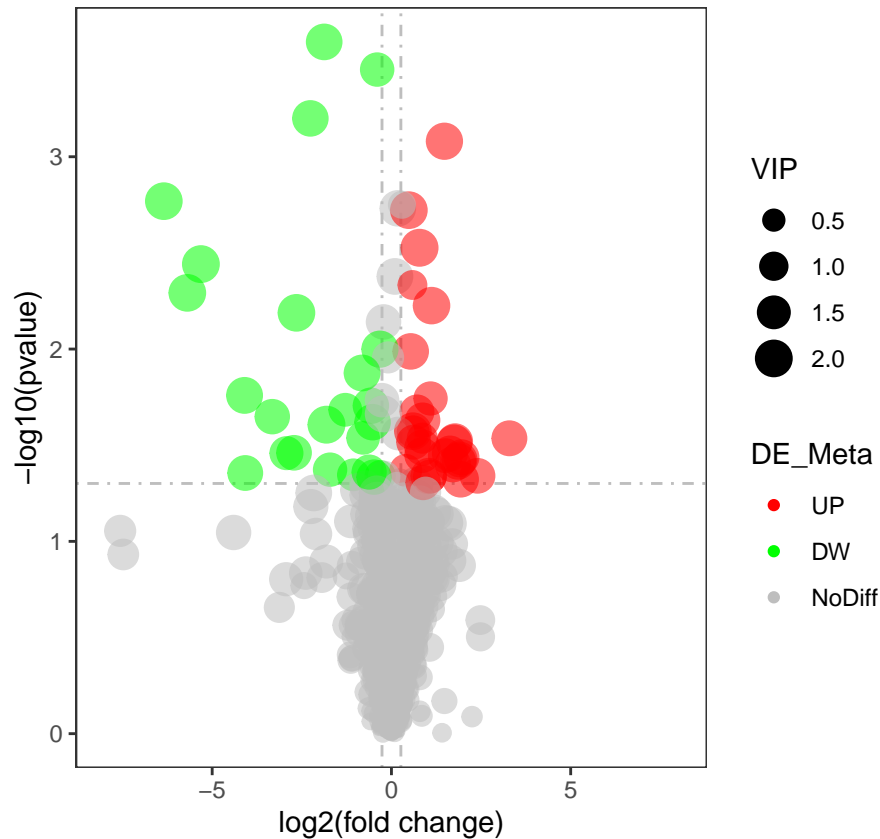

Supplement: Supplementary file 2 [file Data_Sheet_1.ZIP › Result-X101SC22030966-Z01-J001-B1-42 (quasi-targeted metabolomics)/3.MetDiffScreening/High_GAA.vs.Control/High_GAA.vs.Control_all.xls.volcano.pdf]

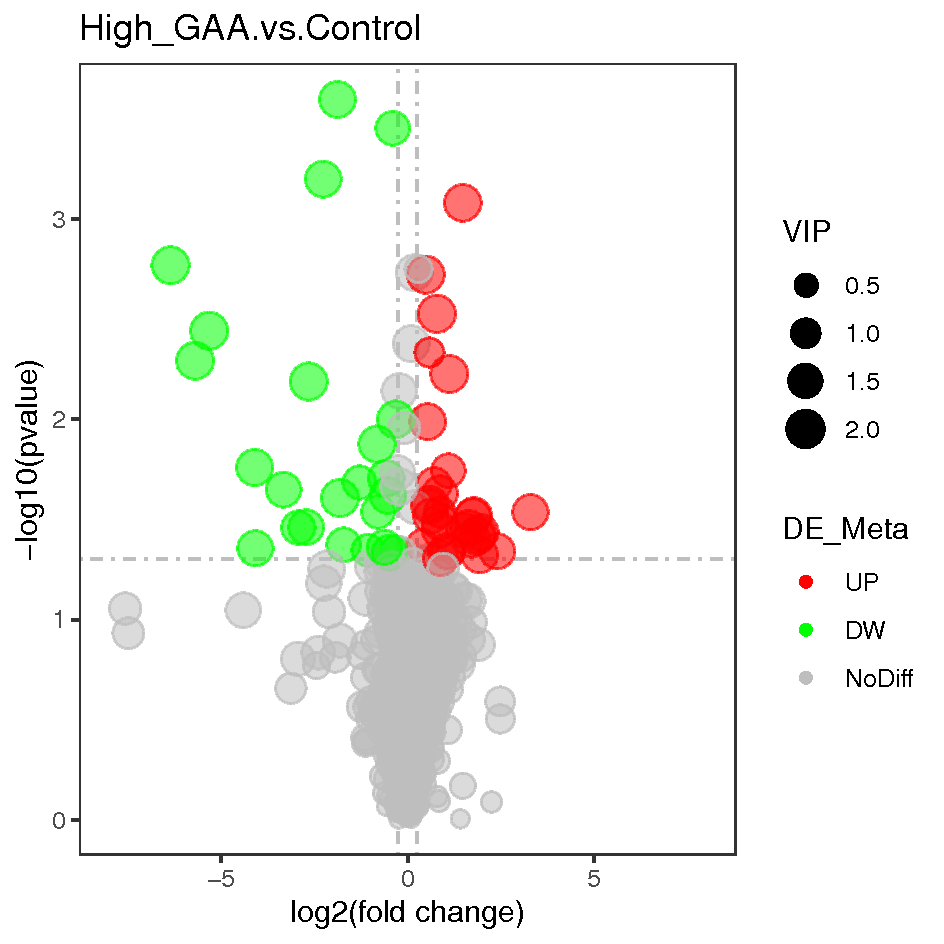

Supplement: Supplementary file 2 [file Data_Sheet_1.ZIP › Result-X101SC22030966-Z01-J001-B1-42 (quasi-targeted metabolomics)/3.MetDiffScreening/High_GAA.vs.Control/High_GAA.vs.Control_all.xls.volcano.png]

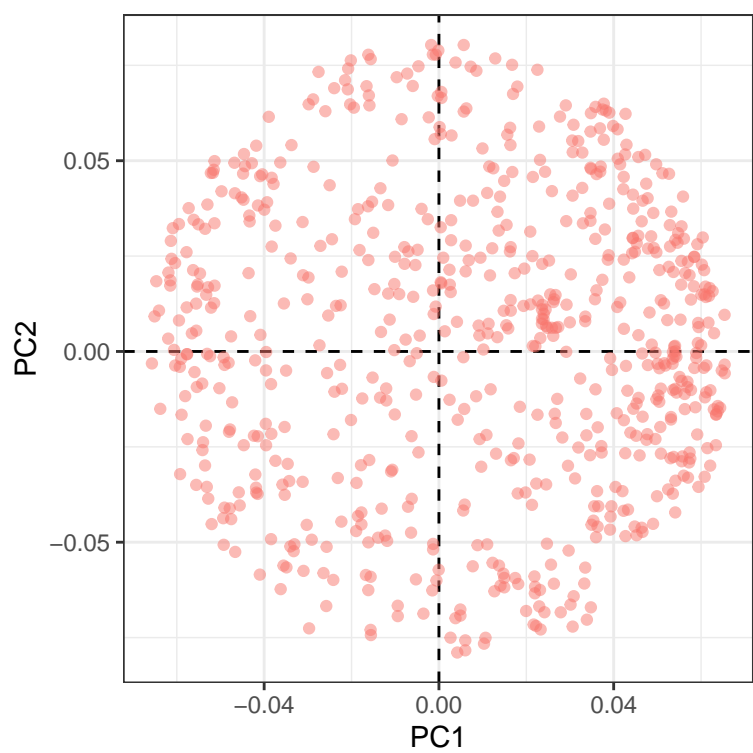

Supplement: Supplementary file 2 [file Data_Sheet_1.ZIP › Result-X101SC22030966-Z01-J001-B1-42 (quasi-targeted metabolomics)/3.MetDiffScreening/High_GAA.vs.Control/High_GAA.vs.Control_all_PCA-pcaloading.pdf]

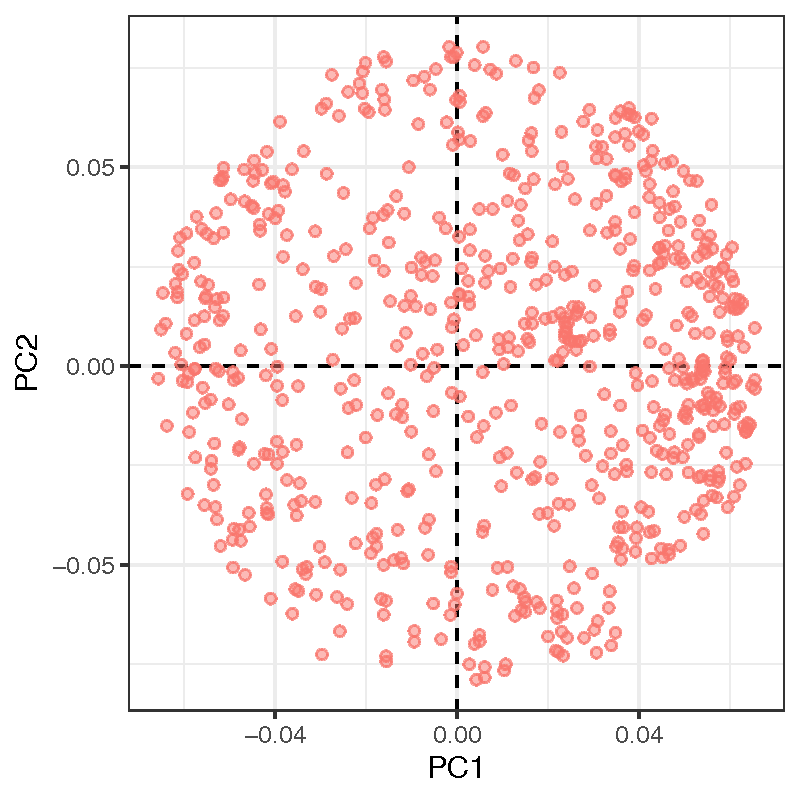

Supplement: Supplementary file 2 [file Data_Sheet_1.ZIP › Result-X101SC22030966-Z01-J001-B1-42 (quasi-targeted metabolomics)/3.MetDiffScreening/High_GAA.vs.Control/High_GAA.vs.Control_all_PCA-pcaloading.png]

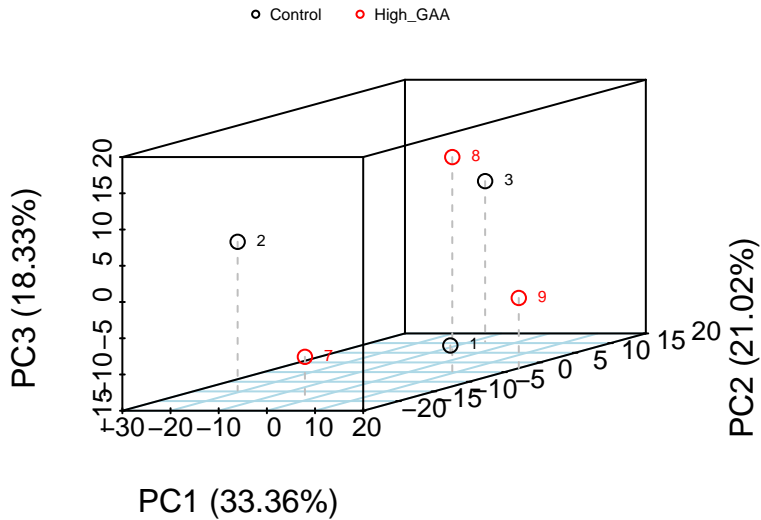

Supplement: Supplementary file 2 [file Data_Sheet_1.ZIP › Result-X101SC22030966-Z01-J001-B1-42 (quasi-targeted metabolomics)/3.MetDiffScreening/High_GAA.vs.Control/High_GAA.vs.Control_all_PCA.3D.pdf]

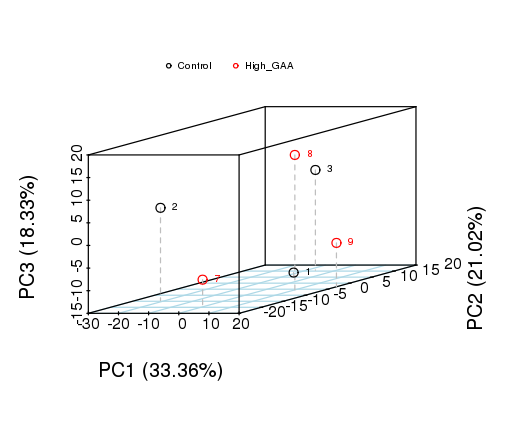

Supplement: Supplementary file 2 [file Data_Sheet_1.ZIP › Result-X101SC22030966-Z01-J001-B1-42 (quasi-targeted metabolomics)/3.MetDiffScreening/High_GAA.vs.Control/High_GAA.vs.Control_all_PCA.3D.png]

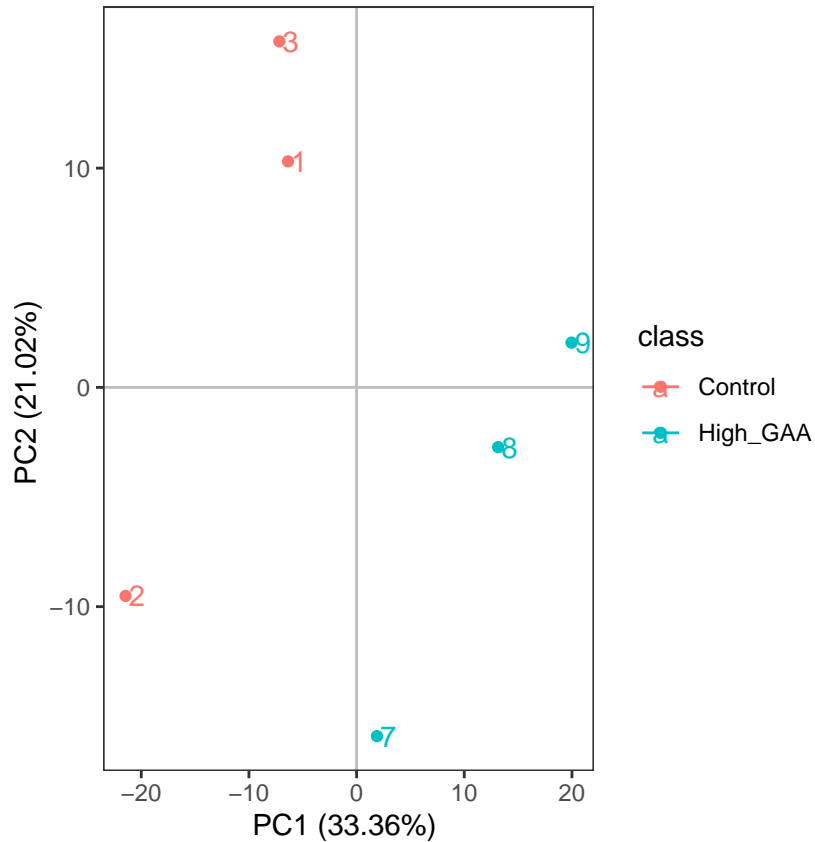

Supplement: Supplementary file 2 [file Data_Sheet_1.ZIP › Result-X101SC22030966-Z01-J001-B1-42 (quasi-targeted metabolomics)/3.MetDiffScreening/High_GAA.vs.Control/High_GAA.vs.Control_all_PCA.pdf]

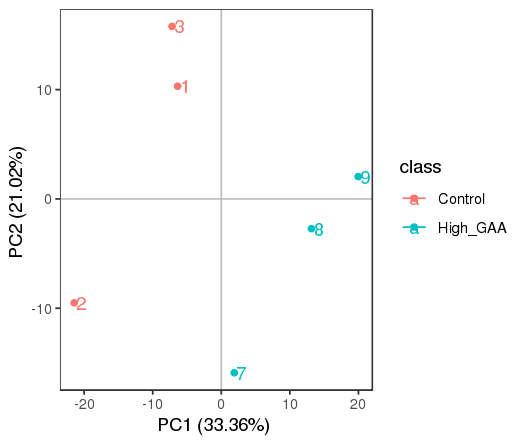

Supplement: Supplementary file 2 [file Data_Sheet_1.ZIP › Result-X101SC22030966-Z01-J001-B1-42 (quasi-targeted metabolomics)/3.MetDiffScreening/High_GAA.vs.Control/High_GAA.vs.Control_all_PCA.png]

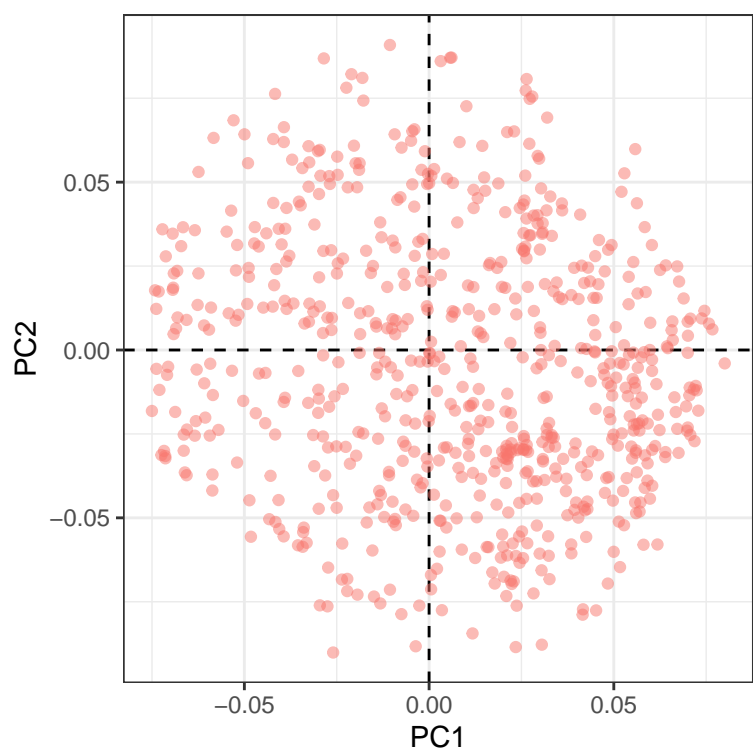

Supplement: Supplementary file 2 [file Data_Sheet_1.ZIP › Result-X101SC22030966-Z01-J001-B1-42 (quasi-targeted metabolomics)/3.MetDiffScreening/High_GAA.vs.Control/High_GAA.vs.Control_all_PLSDA-loading.pdf]

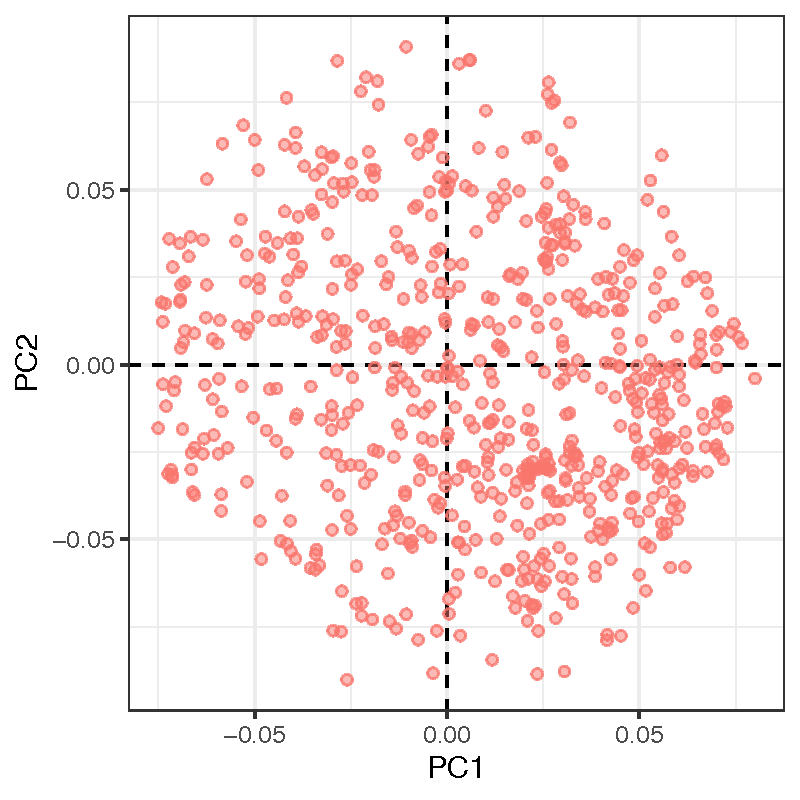

Supplement: Supplementary file 2 [file Data_Sheet_1.ZIP › Result-X101SC22030966-Z01-J001-B1-42 (quasi-targeted metabolomics)/3.MetDiffScreening/High_GAA.vs.Control/High_GAA.vs.Control_all_PLSDA-loading.png]

class    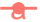 Control    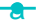 High\_GAA

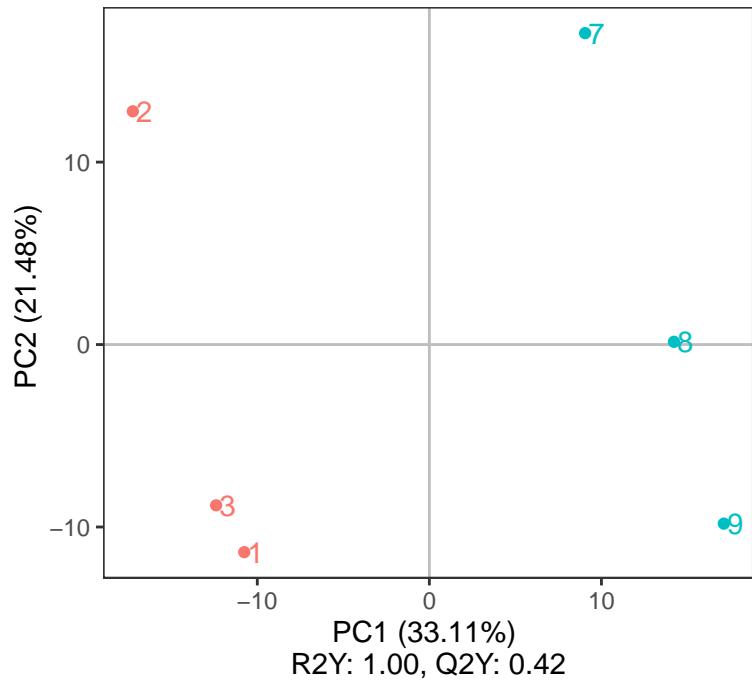

Supplement: Supplementary file 2 [file Data_Sheet_1.ZIP › Result-X101SC22030966-Z01-J001-B1-42 (quasi-targeted metabolomics)/3.MetDiffScreening/High_GAA.vs.Control/High_GAA.vs.Control_all_PLSDA-score.pdf]

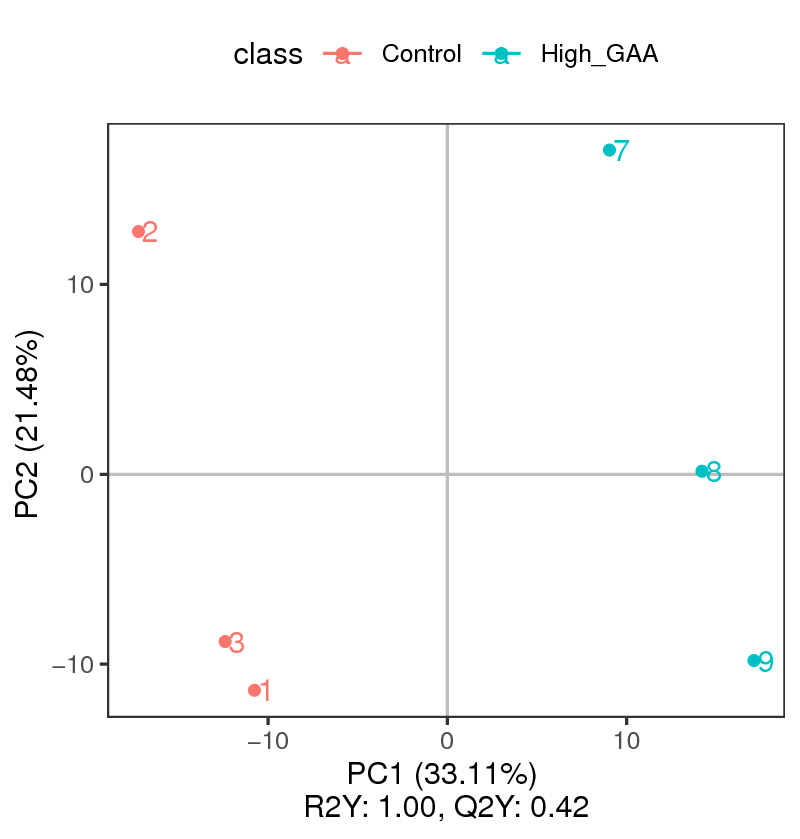

Supplement: Supplementary file 2 [file Data_Sheet_1.ZIP › Result-X101SC22030966-Z01-J001-B1-42 (quasi-targeted metabolomics)/3.MetDiffScreening/High_GAA.vs.Control/High_GAA.vs.Control_all_PLSDA-score.png]

High\_GAA\_Control  
Intercepts:  $R^2=(0.0, 0.99)$ ,  $Q^2=(0.0, -1.24)$

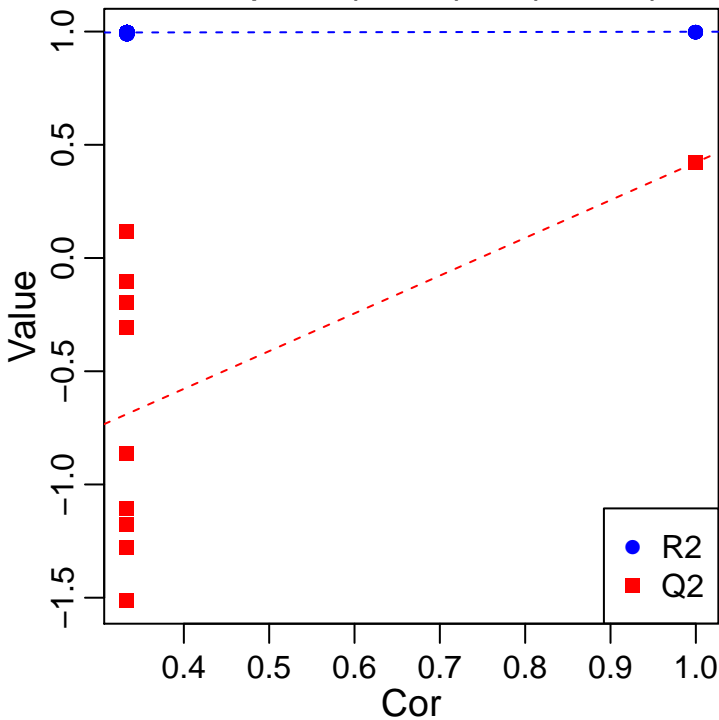

Supplement: Supplementary file 2 [file Data_Sheet_1.ZIP › Result-X101SC22030966-Z01-J001-B1-42 (quasi-targeted metabolomics)/3.MetDiffScreening/High_GAA.vs.Control/High_GAA.vs.Control_all_PLSDA-valid.pdf]

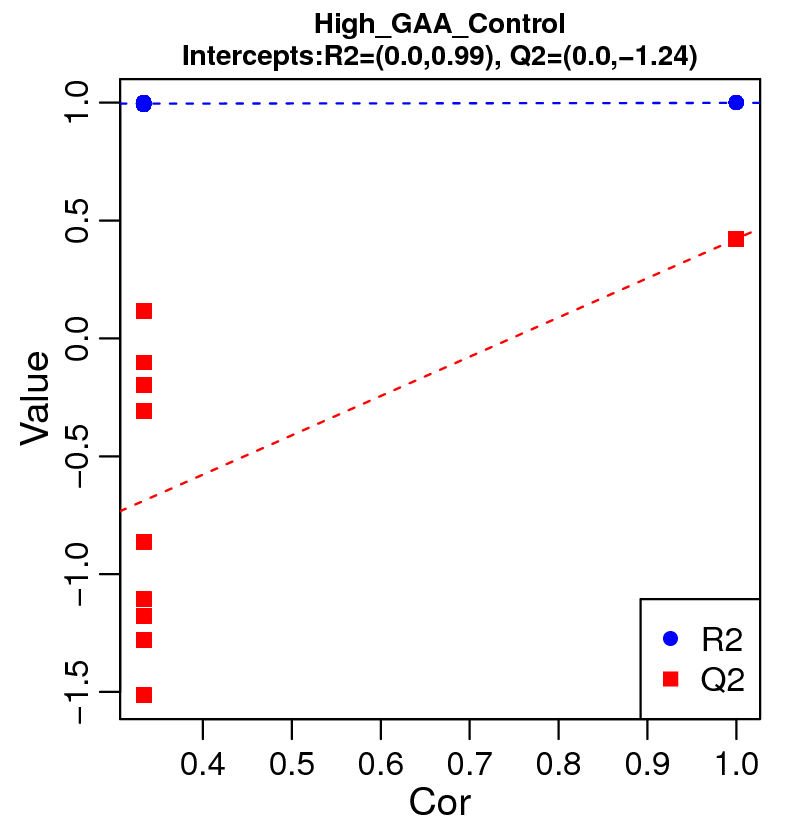

Supplement: Supplementary file 2 [file Data_Sheet_1.ZIP › Result-X101SC22030966-Z01-J001-B1-42 (quasi-targeted metabolomics)/3.MetDiffScreening/High_GAA.vs.Control/High_GAA.vs.Control_all_PLSDA-valid.png]

# High\_GAA.vs.No\_GAA

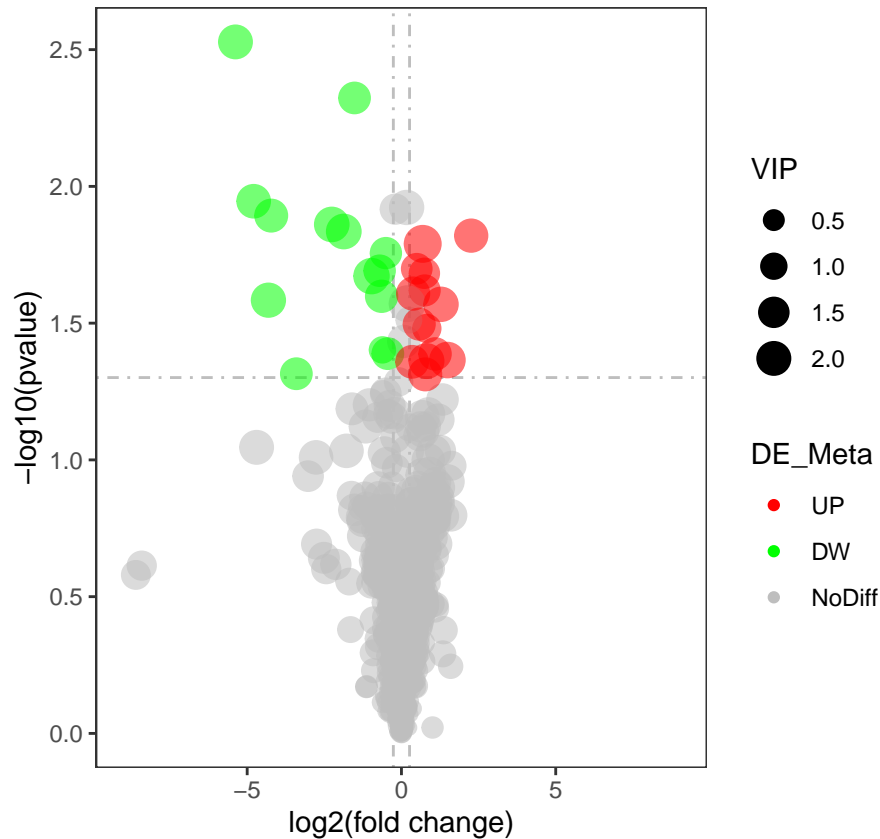

Supplement: Supplementary file 2 [file Data_Sheet_1.ZIP › Result-X101SC22030966-Z01-J001-B1-42 (quasi-targeted metabolomics)/3.MetDiffScreening/High_GAA.vs.No_GAA/High_GAA.vs.No_GAA_all.xls.volcano.pdf]

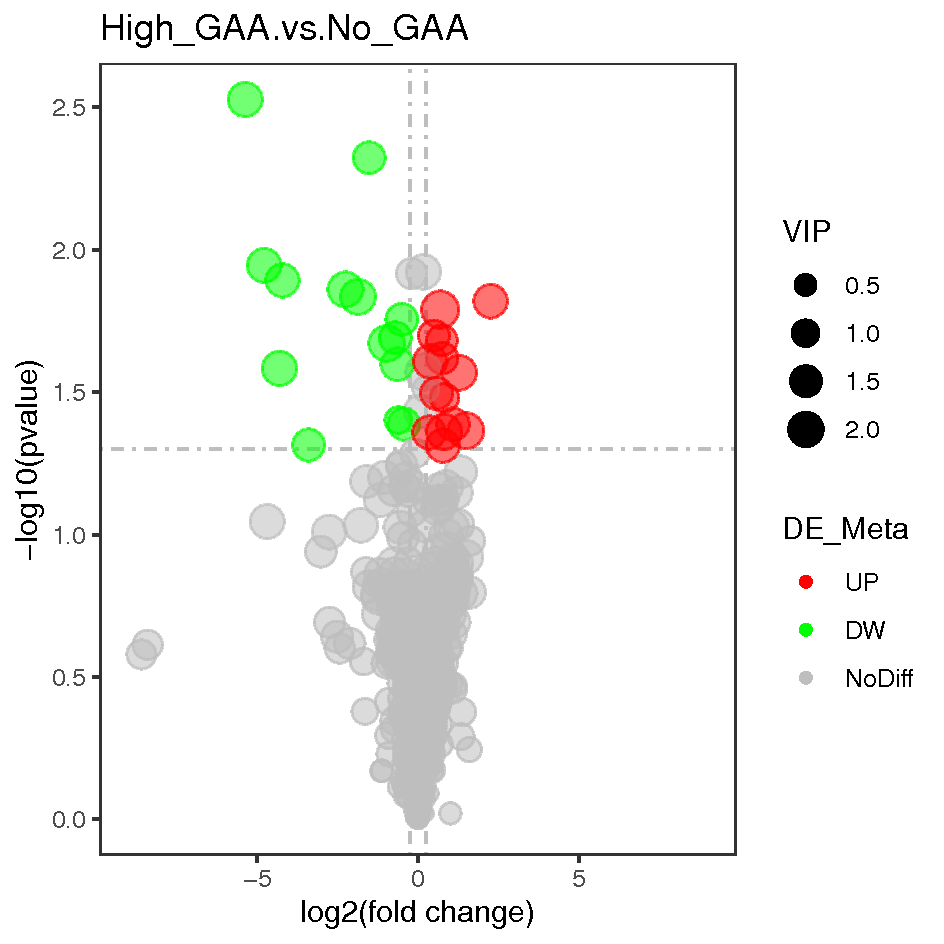

Supplement: Supplementary file 2 [file Data_Sheet_1.ZIP › Result-X101SC22030966-Z01-J001-B1-42 (quasi-targeted metabolomics)/3.MetDiffScreening/High_GAA.vs.No_GAA/High_GAA.vs.No_GAA_all.xls.volcano.png]

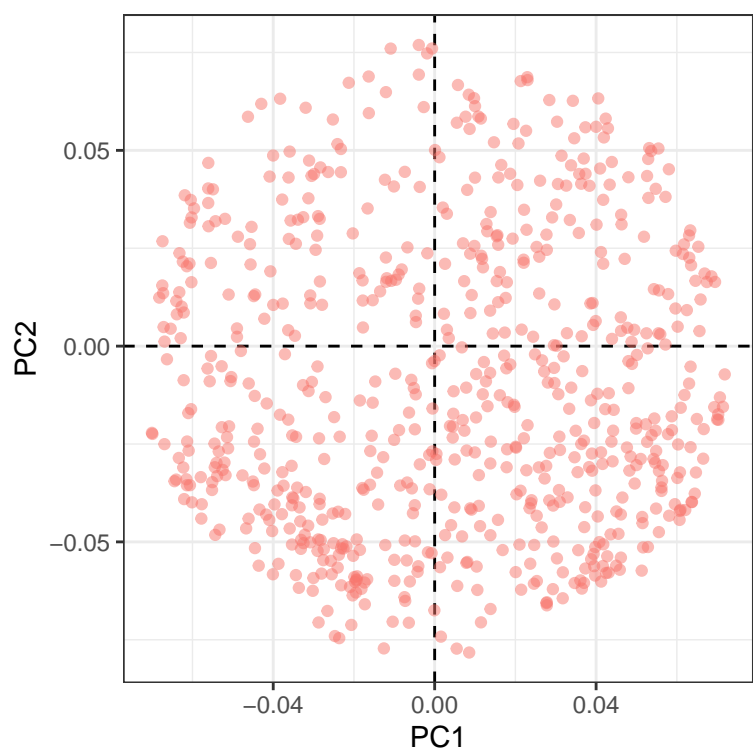

Supplement: Supplementary file 2 [file Data_Sheet_1.ZIP › Result-X101SC22030966-Z01-J001-B1-42 (quasi-targeted metabolomics)/3.MetDiffScreening/High_GAA.vs.No_GAA/High_GAA.vs.No_GAA_all_PCA-pcaloading.pdf]

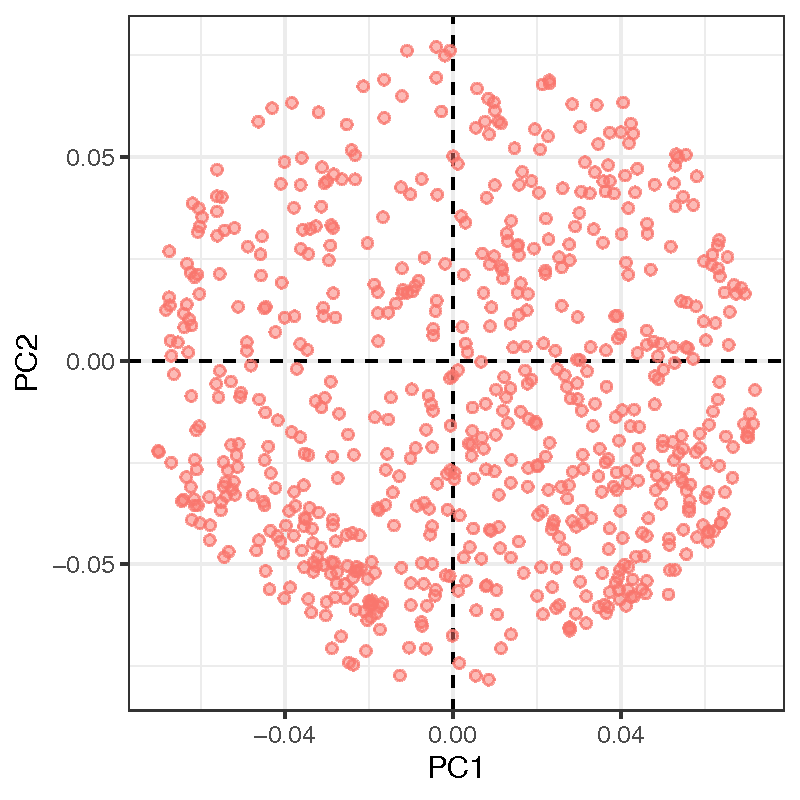

Supplement: Supplementary file 2 [file Data_Sheet_1.ZIP › Result-X101SC22030966-Z01-J001-B1-42 (quasi-targeted metabolomics)/3.MetDiffScreening/High_GAA.vs.No_GAA/High_GAA.vs.No_GAA_all_PCA-pcaloading.png]

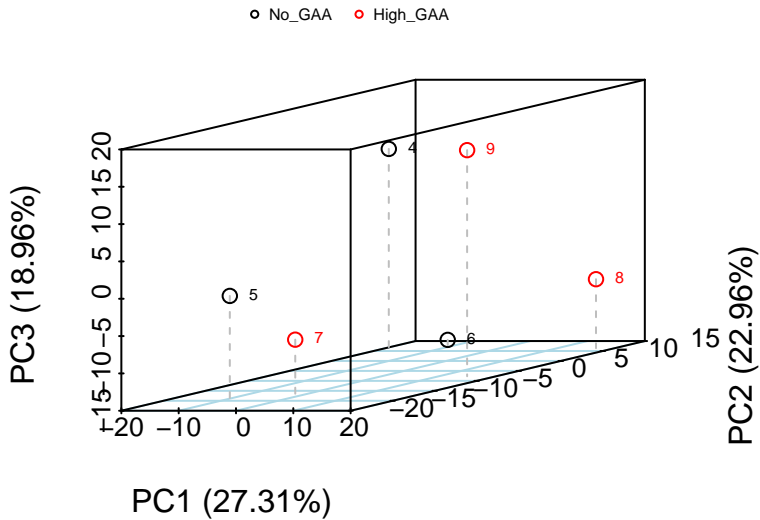

Supplement: Supplementary file 2 [file Data_Sheet_1.ZIP › Result-X101SC22030966-Z01-J001-B1-42 (quasi-targeted metabolomics)/3.MetDiffScreening/High_GAA.vs.No_GAA/High_GAA.vs.No_GAA_all_PCA.3D.pdf]

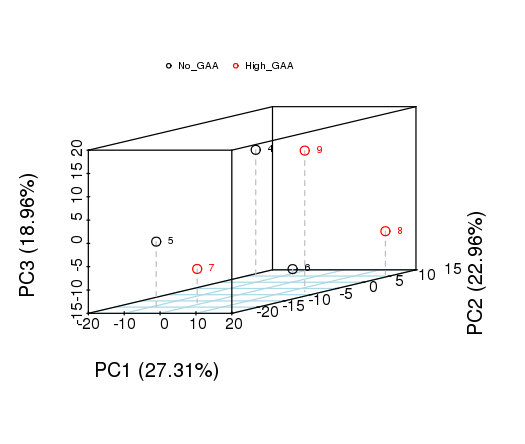

Supplement: Supplementary file 2 [file Data_Sheet_1.ZIP › Result-X101SC22030966-Z01-J001-B1-42 (quasi-targeted metabolomics)/3.MetDiffScreening/High_GAA.vs.No_GAA/High_GAA.vs.No_GAA_all_PCA.3D.png]

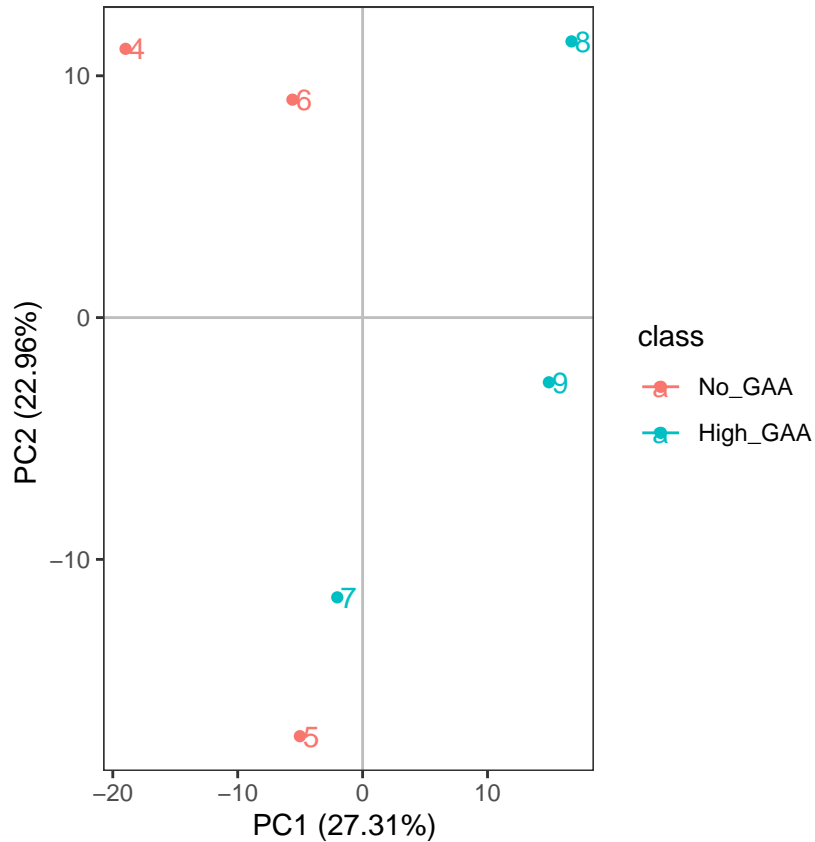

Supplement: Supplementary file 2 [file Data_Sheet_1.ZIP › Result-X101SC22030966-Z01-J001-B1-42 (quasi-targeted metabolomics)/3.MetDiffScreening/High_GAA.vs.No_GAA/High_GAA.vs.No_GAA_all_PCA.pdf]

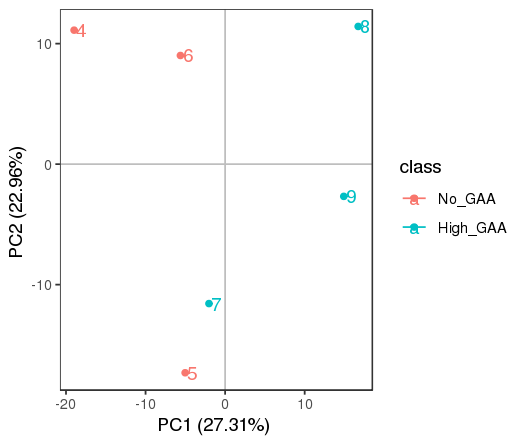

Supplement: Supplementary file 2 [file Data_Sheet_1.ZIP › Result-X101SC22030966-Z01-J001-B1-42 (quasi-targeted metabolomics)/3.MetDiffScreening/High_GAA.vs.No_GAA/High_GAA.vs.No_GAA_all_PCA.png]

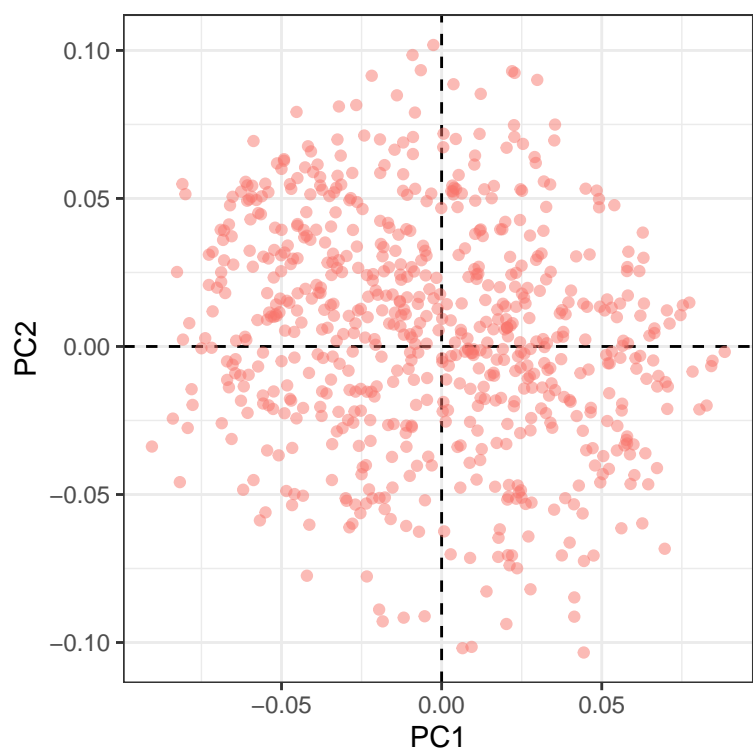

Supplement: Supplementary file 2 [file Data_Sheet_1.ZIP › Result-X101SC22030966-Z01-J001-B1-42 (quasi-targeted metabolomics)/3.MetDiffScreening/High_GAA.vs.No_GAA/High_GAA.vs.No_GAA_all_PLSDA-loading.pdf]

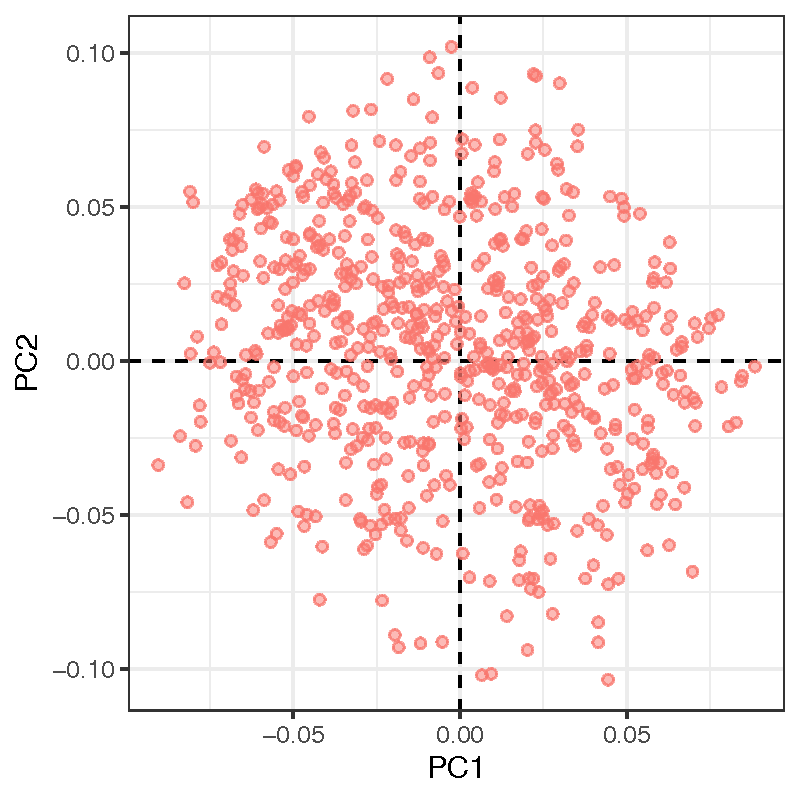

Supplement: Supplementary file 2 [file Data_Sheet_1.ZIP › Result-X101SC22030966-Z01-J001-B1-42 (quasi-targeted metabolomics)/3.MetDiffScreening/High_GAA.vs.No_GAA/High_GAA.vs.No_GAA_all_PLSDA-loading.png]

class    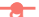 High\_GAA    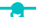 No\_GAA

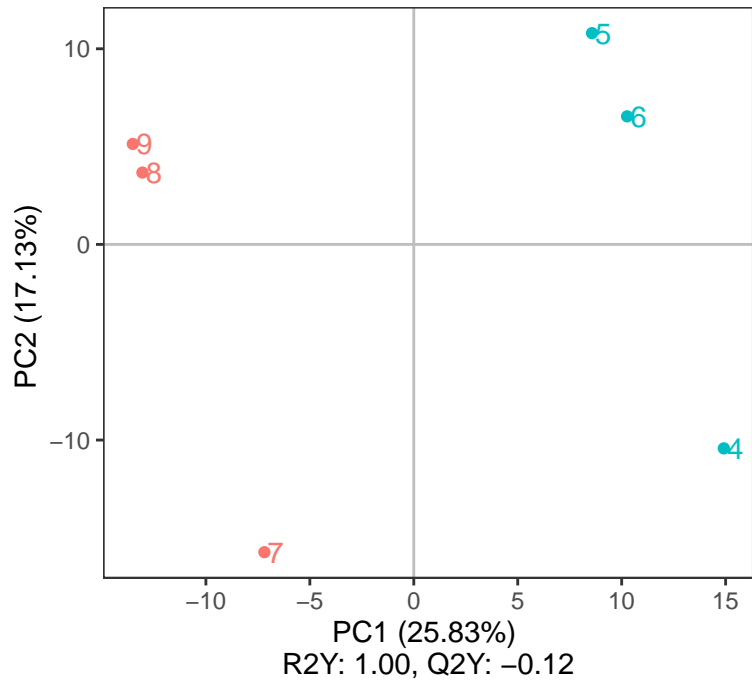

Supplement: Supplementary file 2 [file Data_Sheet_1.ZIP › Result-X101SC22030966-Z01-J001-B1-42 (quasi-targeted metabolomics)/3.MetDiffScreening/High_GAA.vs.No_GAA/High_GAA.vs.No_GAA_all_PLSDA-score.pdf]

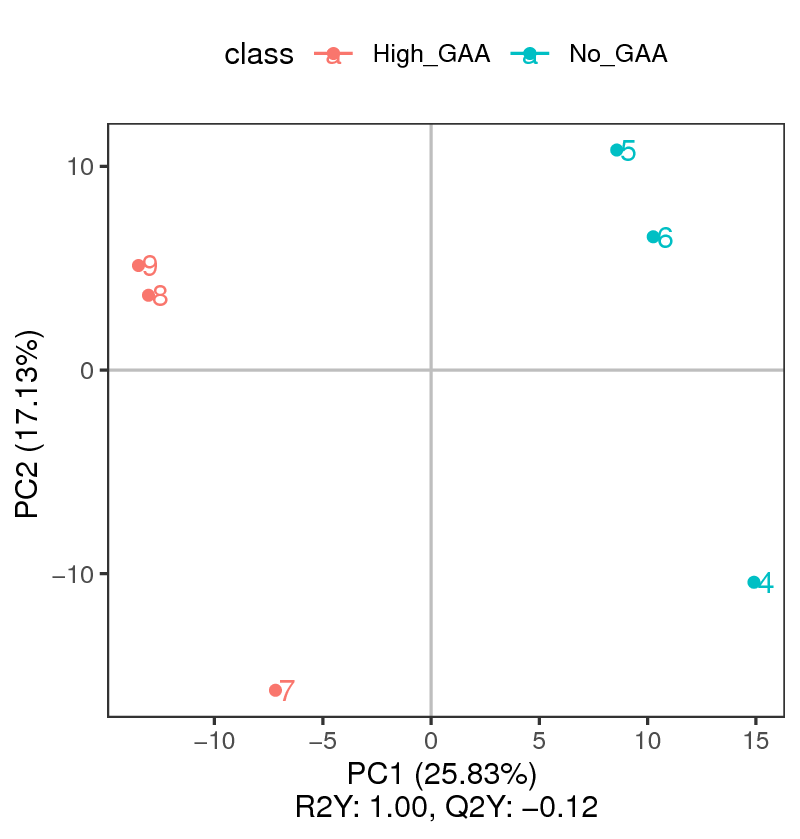

Supplement: Supplementary file 2 [file Data_Sheet_1.ZIP › Result-X101SC22030966-Z01-J001-B1-42 (quasi-targeted metabolomics)/3.MetDiffScreening/High_GAA.vs.No_GAA/High_GAA.vs.No_GAA_all_PLSDA-score.png]

High\_GAA\_No\_GAA  
Intercepts:R2=(0.0,1.00), Q2=(0.0,-0.80)

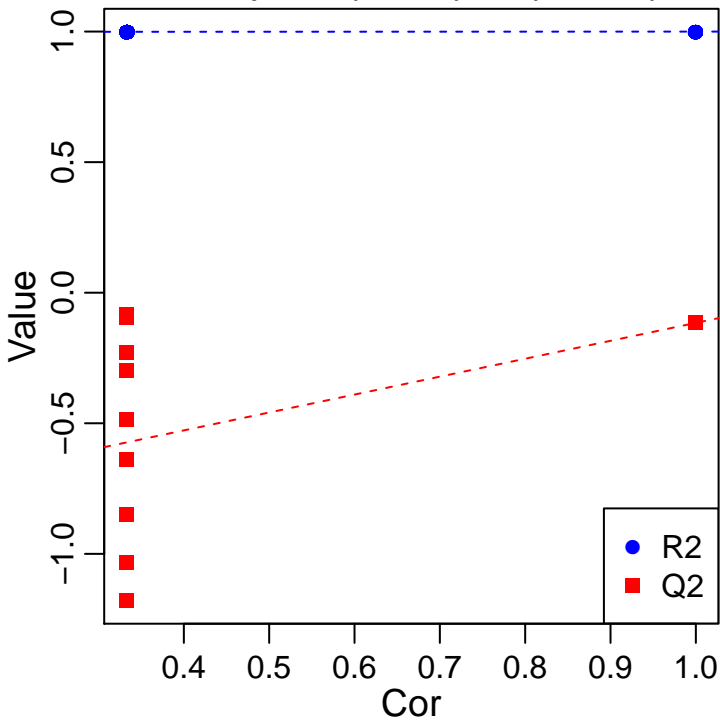

Supplement: Supplementary file 2 [file Data_Sheet_1.ZIP › Result-X101SC22030966-Z01-J001-B1-42 (quasi-targeted metabolomics)/3.MetDiffScreening/High_GAA.vs.No_GAA/High_GAA.vs.No_GAA_all_PLSDA-valid.pdf]

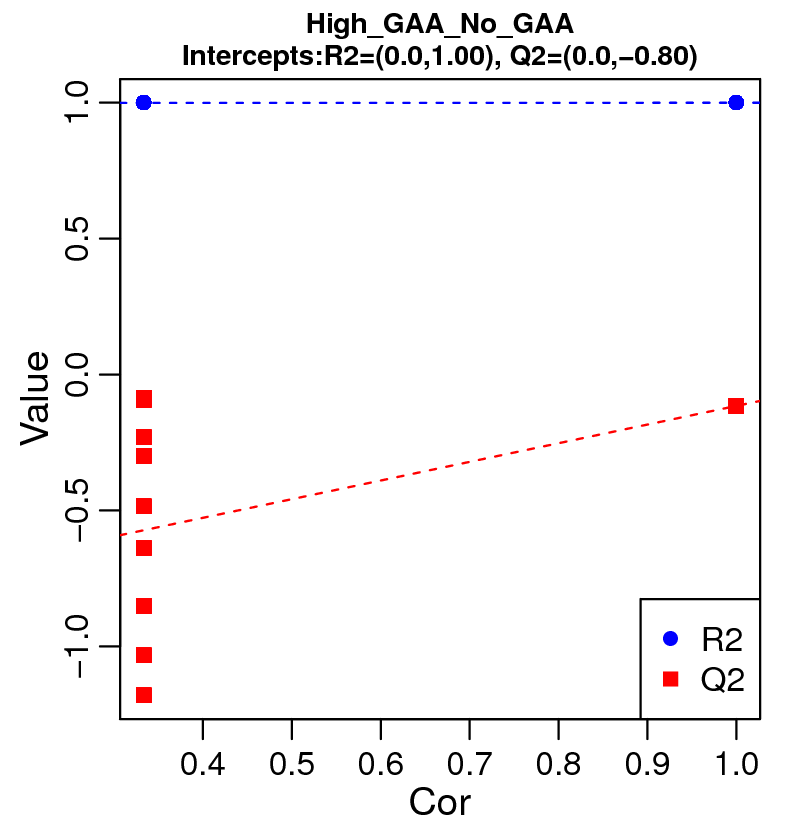

Supplement: Supplementary file 2 [file Data_Sheet_1.ZIP › Result-X101SC22030966-Z01-J001-B1-42 (quasi-targeted metabolomics)/3.MetDiffScreening/High_GAA.vs.No_GAA/High_GAA.vs.No_GAA_all_PLSDA-valid.png]

# No\_GAA.vs.Control

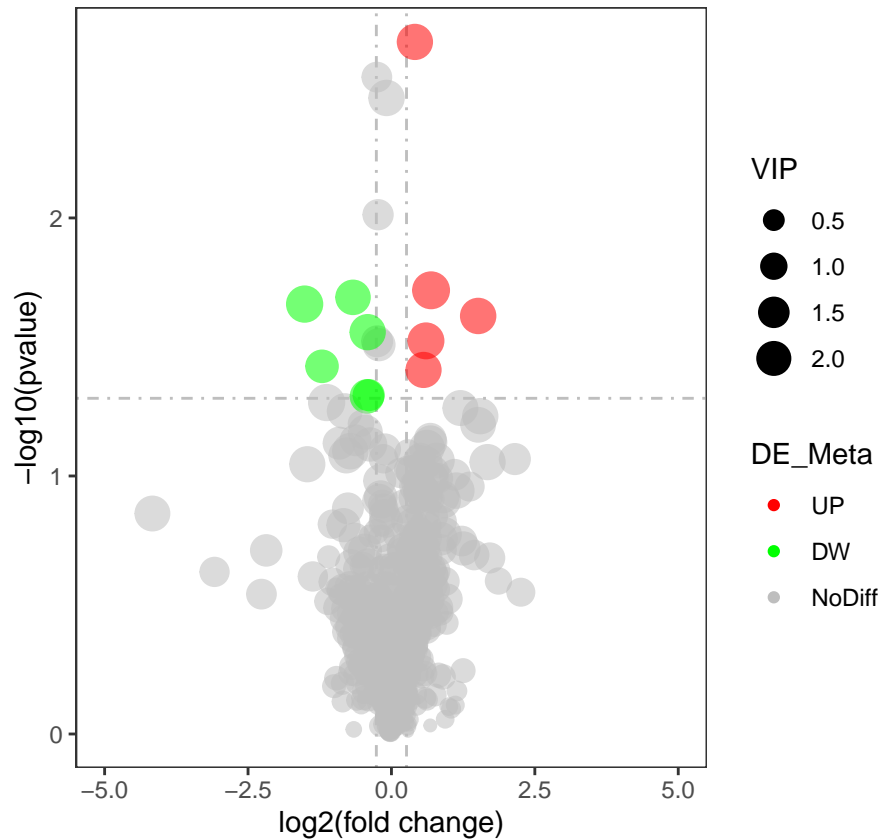

Supplement: Supplementary file 2 [file Data_Sheet_1.ZIP › Result-X101SC22030966-Z01-J001-B1-42 (quasi-targeted metabolomics)/3.MetDiffScreening/No_GAA.vs.Control/No_GAA.vs.Control_all.xls.volcano.pdf]

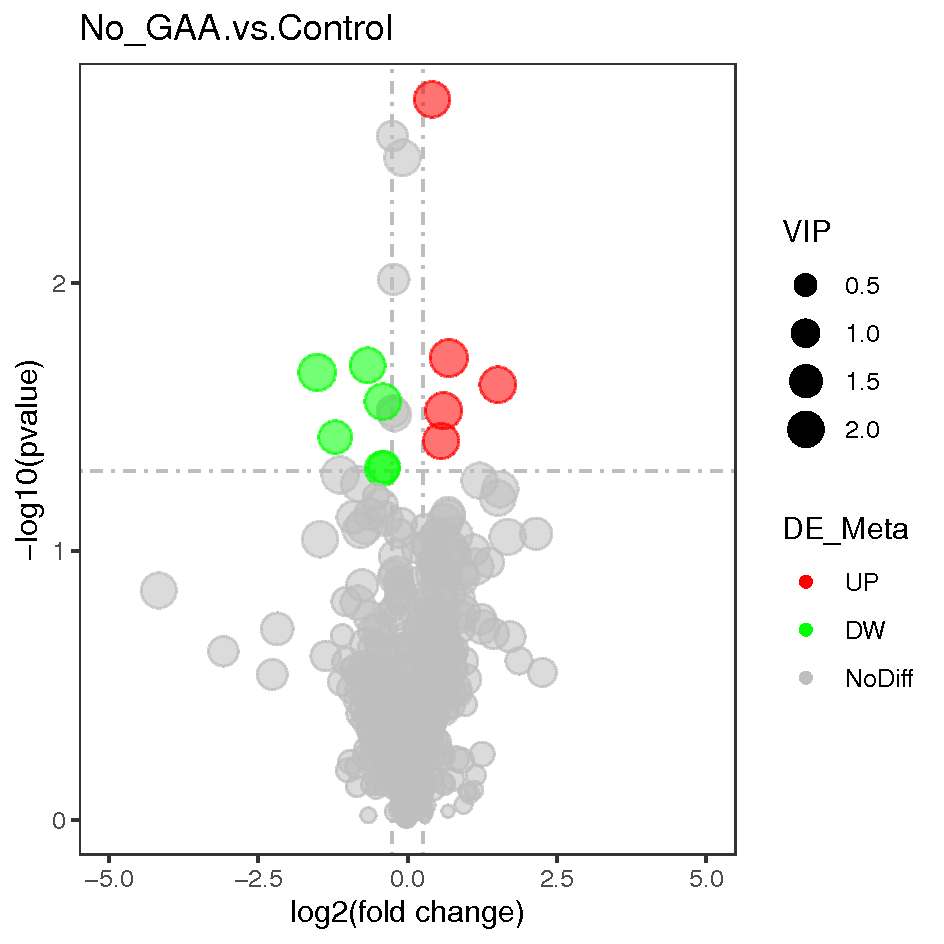

Supplement: Supplementary file 2 [file Data_Sheet_1.ZIP › Result-X101SC22030966-Z01-J001-B1-42 (quasi-targeted metabolomics)/3.MetDiffScreening/No_GAA.vs.Control/No_GAA.vs.Control_all.xls.volcano.png]

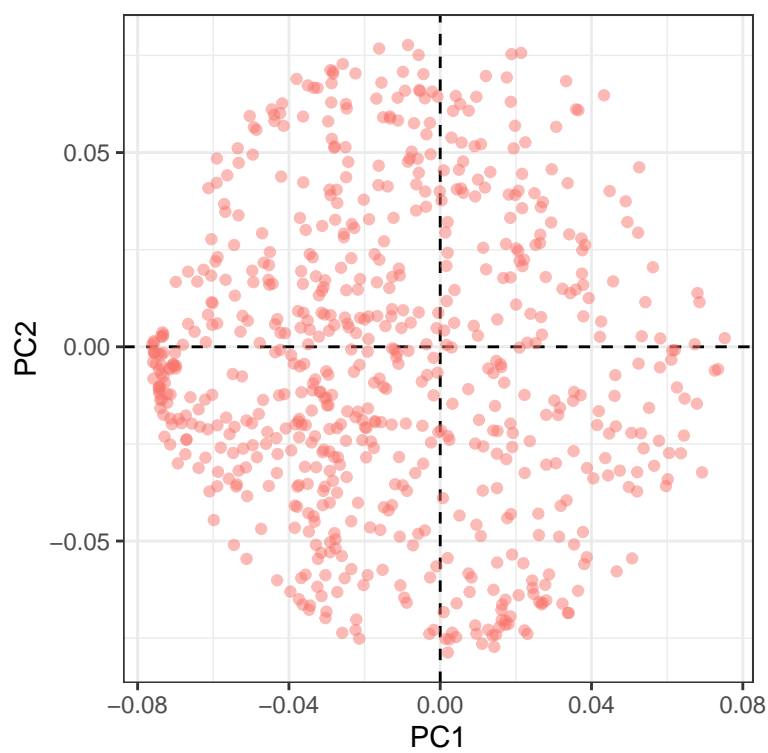

Supplement: Supplementary file 2 [file Data_Sheet_1.ZIP › Result-X101SC22030966-Z01-J001-B1-42 (quasi-targeted metabolomics)/3.MetDiffScreening/No_GAA.vs.Control/No_GAA.vs.Control_all_PCA-pcaloading.pdf]

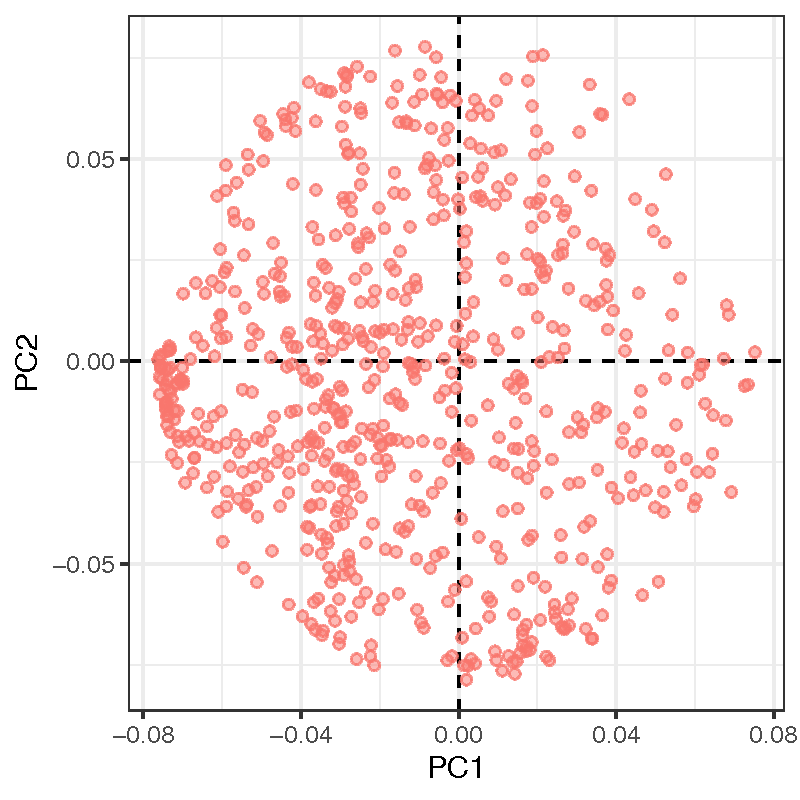

Supplement: Supplementary file 2 [file Data_Sheet_1.ZIP › Result-X101SC22030966-Z01-J001-B1-42 (quasi-targeted metabolomics)/3.MetDiffScreening/No_GAA.vs.Control/No_GAA.vs.Control_all_PCA-pcaloading.png]

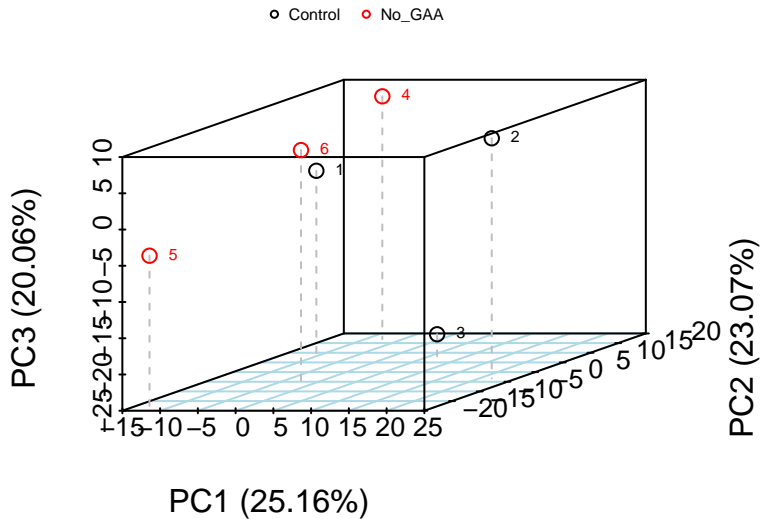

Supplement: Supplementary file 2 [file Data_Sheet_1.ZIP › Result-X101SC22030966-Z01-J001-B1-42 (quasi-targeted metabolomics)/3.MetDiffScreening/No_GAA.vs.Control/No_GAA.vs.Control_all_PCA.3D.pdf]

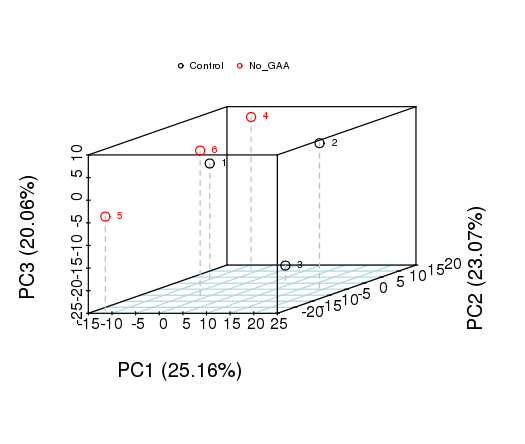

Supplement: Supplementary file 2 [file Data_Sheet_1.ZIP › Result-X101SC22030966-Z01-J001-B1-42 (quasi-targeted metabolomics)/3.MetDiffScreening/No_GAA.vs.Control/No_GAA.vs.Control_all_PCA.3D.png]

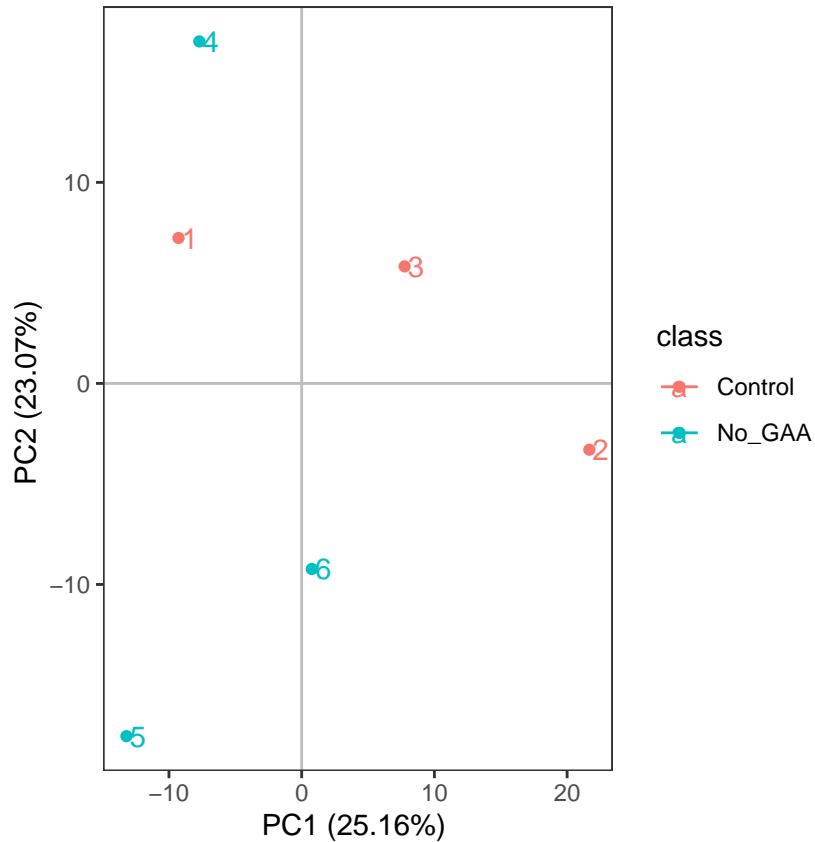

Supplement: Supplementary file 2 [file Data_Sheet_1.ZIP › Result-X101SC22030966-Z01-J001-B1-42 (quasi-targeted metabolomics)/3.MetDiffScreening/No_GAA.vs.Control/No_GAA.vs.Control_all_PCA.pdf]

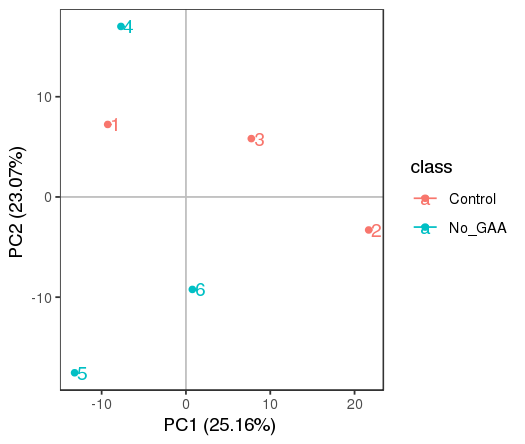

Supplement: Supplementary file 2 [file Data_Sheet_1.ZIP › Result-X101SC22030966-Z01-J001-B1-42 (quasi-targeted metabolomics)/3.MetDiffScreening/No_GAA.vs.Control/No_GAA.vs.Control_all_PCA.png]

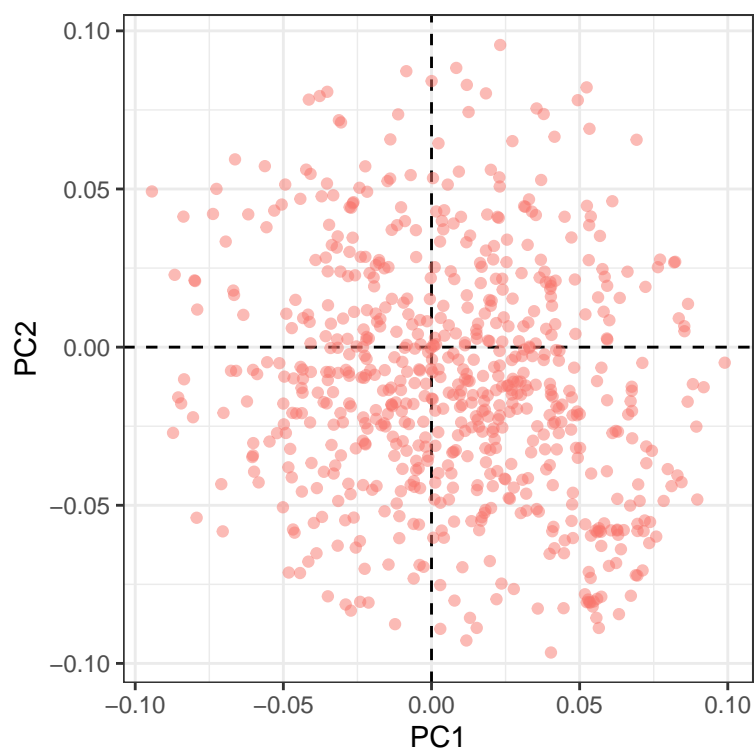

Supplement: Supplementary file 2 [file Data_Sheet_1.ZIP › Result-X101SC22030966-Z01-J001-B1-42 (quasi-targeted metabolomics)/3.MetDiffScreening/No_GAA.vs.Control/No_GAA.vs.Control_all_PLSDA-loading.pdf]

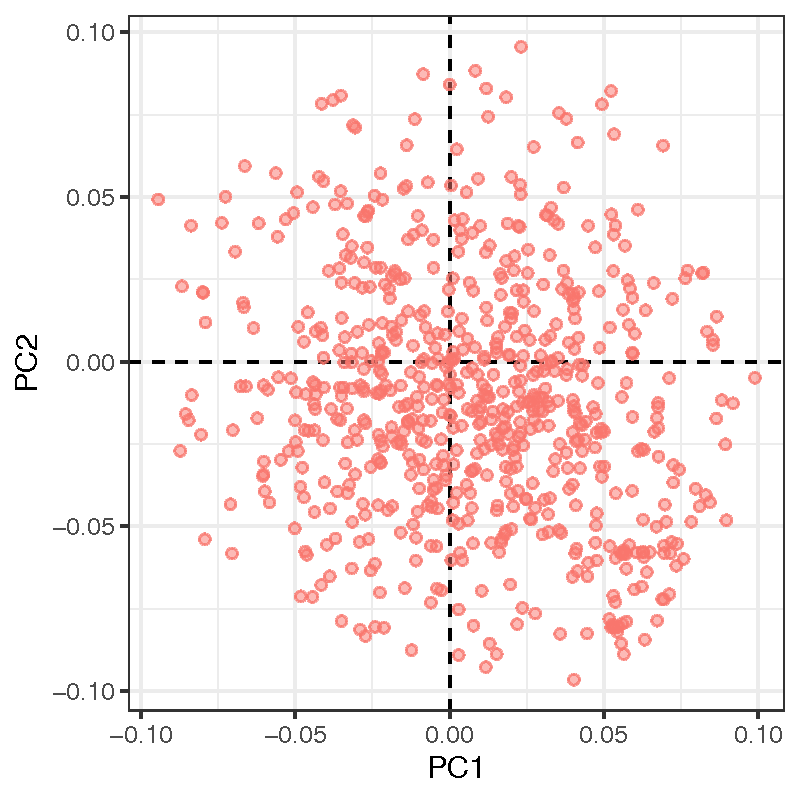

Supplement: Supplementary file 2 [file Data_Sheet_1.ZIP › Result-X101SC22030966-Z01-J001-B1-42 (quasi-targeted metabolomics)/3.MetDiffScreening/No_GAA.vs.Control/No_GAA.vs.Control_all_PLSDA-loading.png]

class    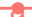 Control    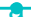 No\_GAA

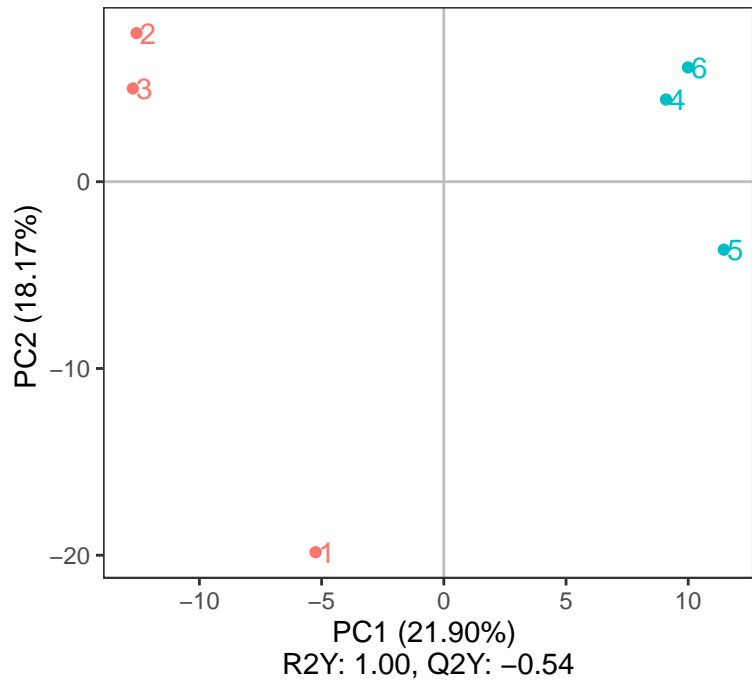

Supplement: Supplementary file 2 [file Data_Sheet_1.ZIP › Result-X101SC22030966-Z01-J001-B1-42 (quasi-targeted metabolomics)/3.MetDiffScreening/No_GAA.vs.Control/No_GAA.vs.Control_all_PLSDA-score.pdf]

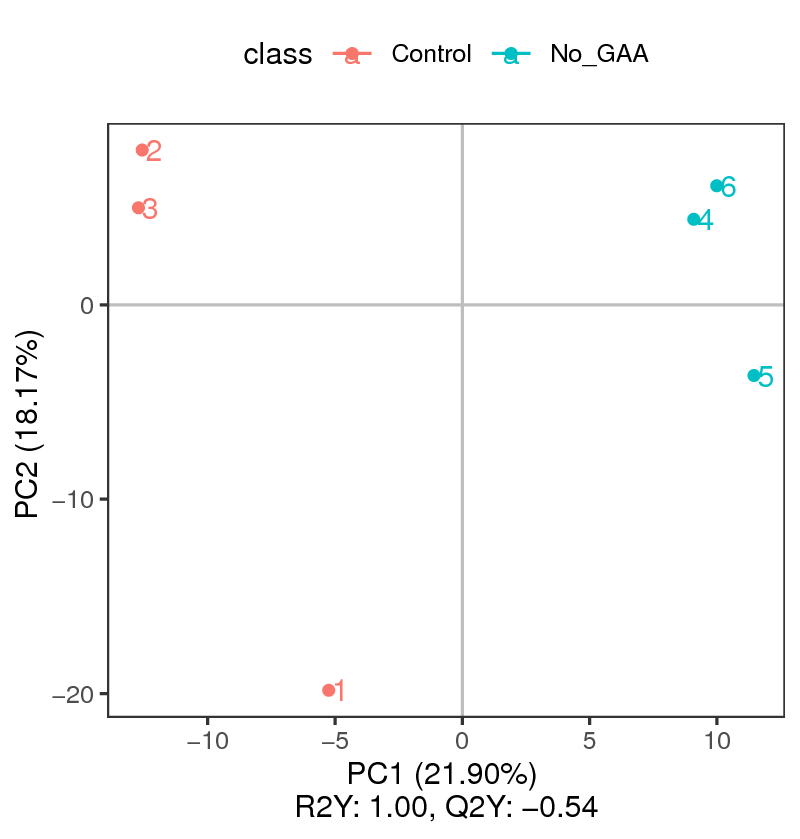

Supplement: Supplementary file 2 [file Data_Sheet_1.ZIP › Result-X101SC22030966-Z01-J001-B1-42 (quasi-targeted metabolomics)/3.MetDiffScreening/No_GAA.vs.Control/No_GAA.vs.Control_all_PLSDA-score.png]

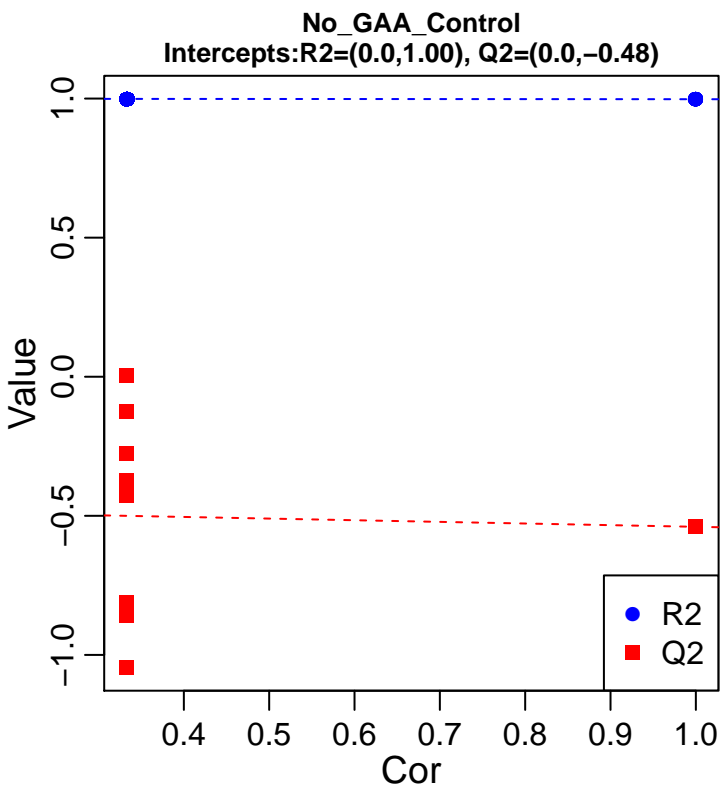

Supplement: Supplementary file 2 [file Data_Sheet_1.ZIP › Result-X101SC22030966-Z01-J001-B1-42 (quasi-targeted metabolomics)/3.MetDiffScreening/No_GAA.vs.Control/No_GAA.vs.Control_all_PLSDA-valid.pdf]

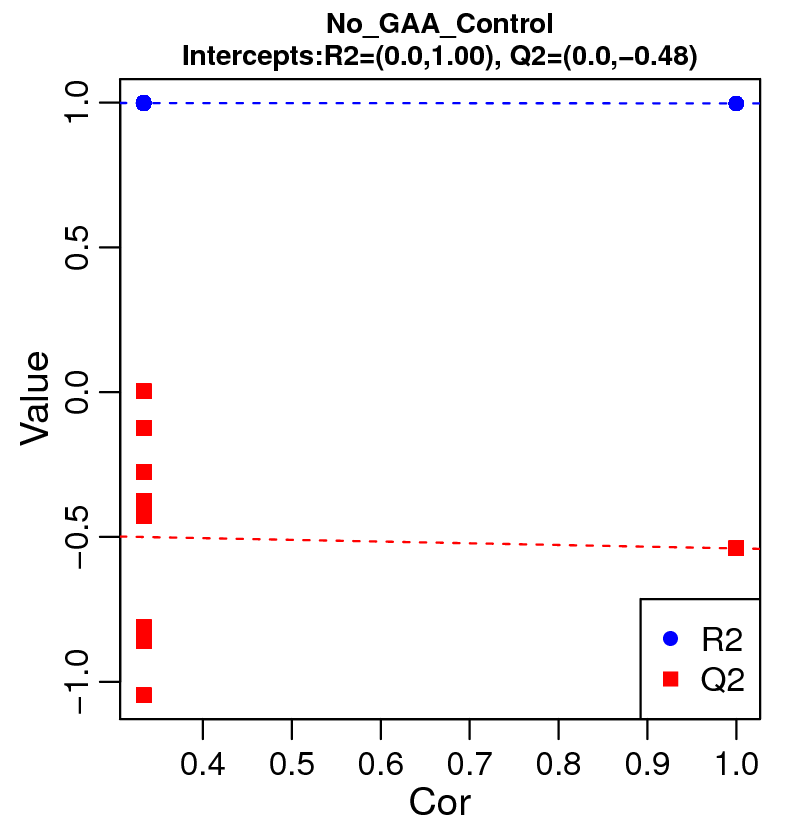

Supplement: Supplementary file 2 [file Data_Sheet_1.ZIP › Result-X101SC22030966-Z01-J001-B1-42 (quasi-targeted metabolomics)/3.MetDiffScreening/No_GAA.vs.Control/No_GAA.vs.Control_all_PLSDA-valid.png]

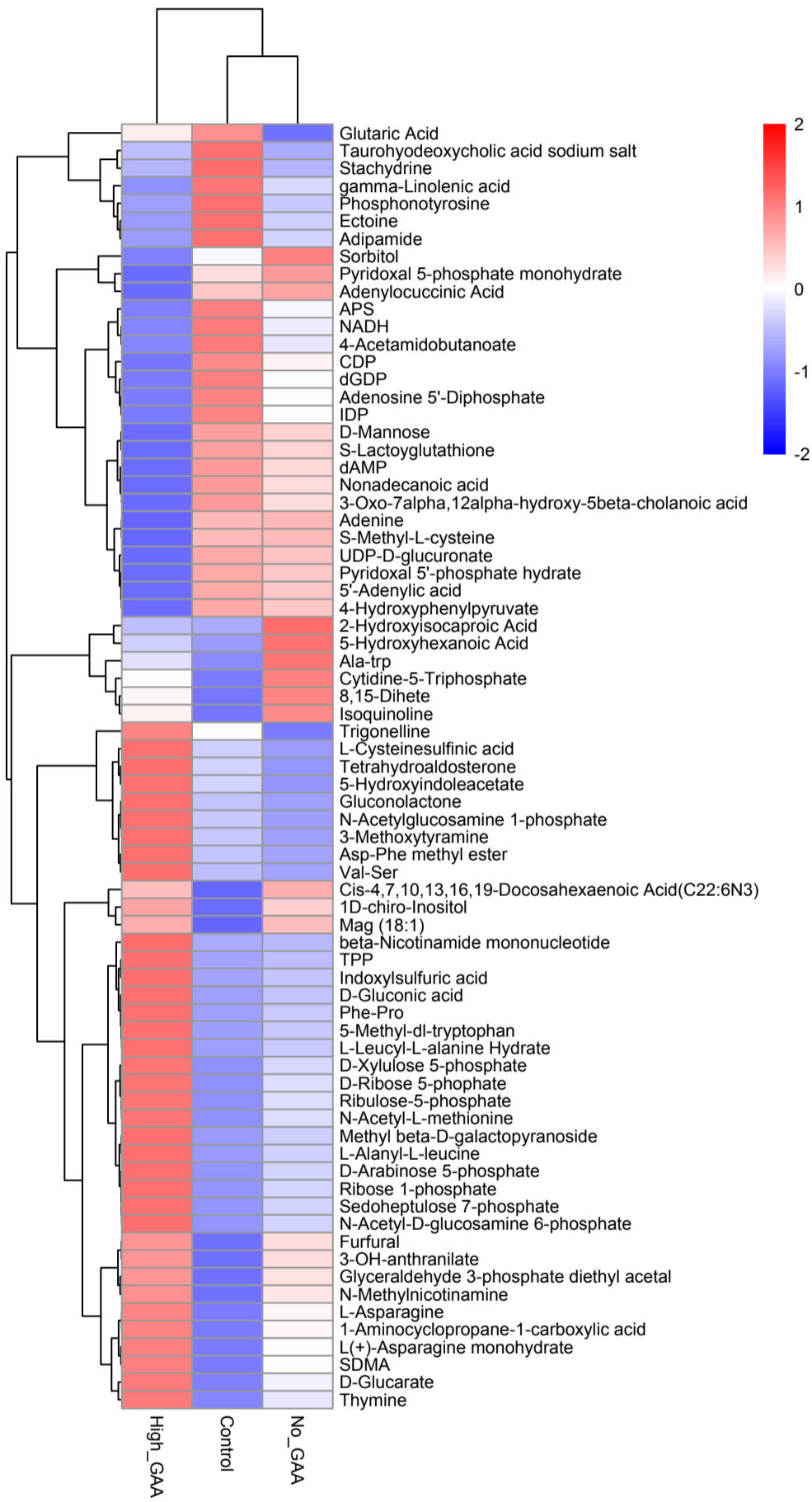

Supplement: Supplementary file 2 [file Data_Sheet_1.ZIP › Result-X101SC22030966-Z01-J001-B1-42 (quasi-targeted metabolomics)/4.MetDiffAnalysis/Heatmap_diff/Diff_Heatmap_all.cluster.detail.pdf]

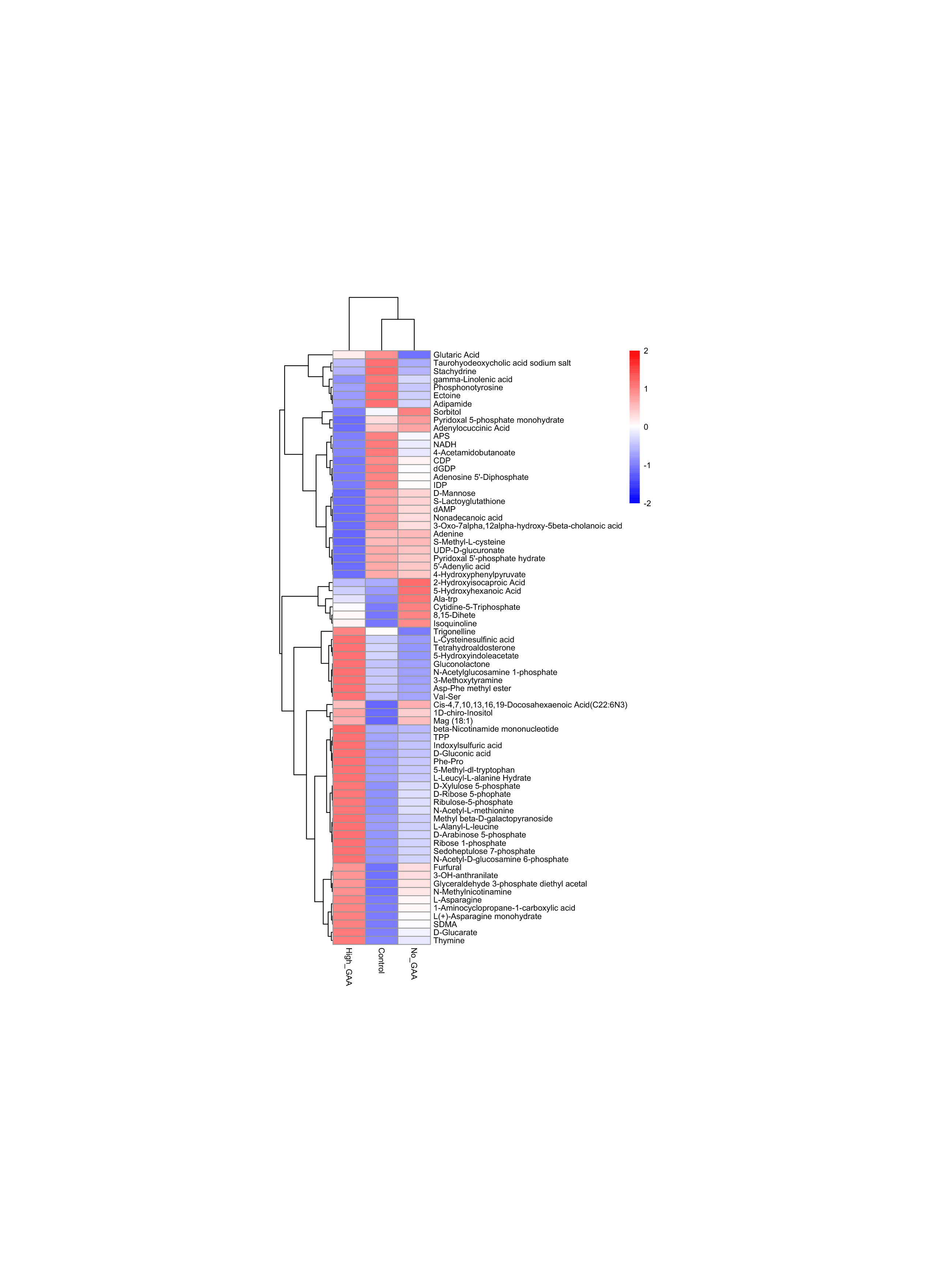

Supplement: Supplementary file 2 [file Data_Sheet_1.ZIP › Result-X101SC22030966-Z01-J001-B1-42 (quasi-targeted metabolomics)/4.MetDiffAnalysis/Heatmap_diff/Diff_Heatmap_all.cluster.detail.png]

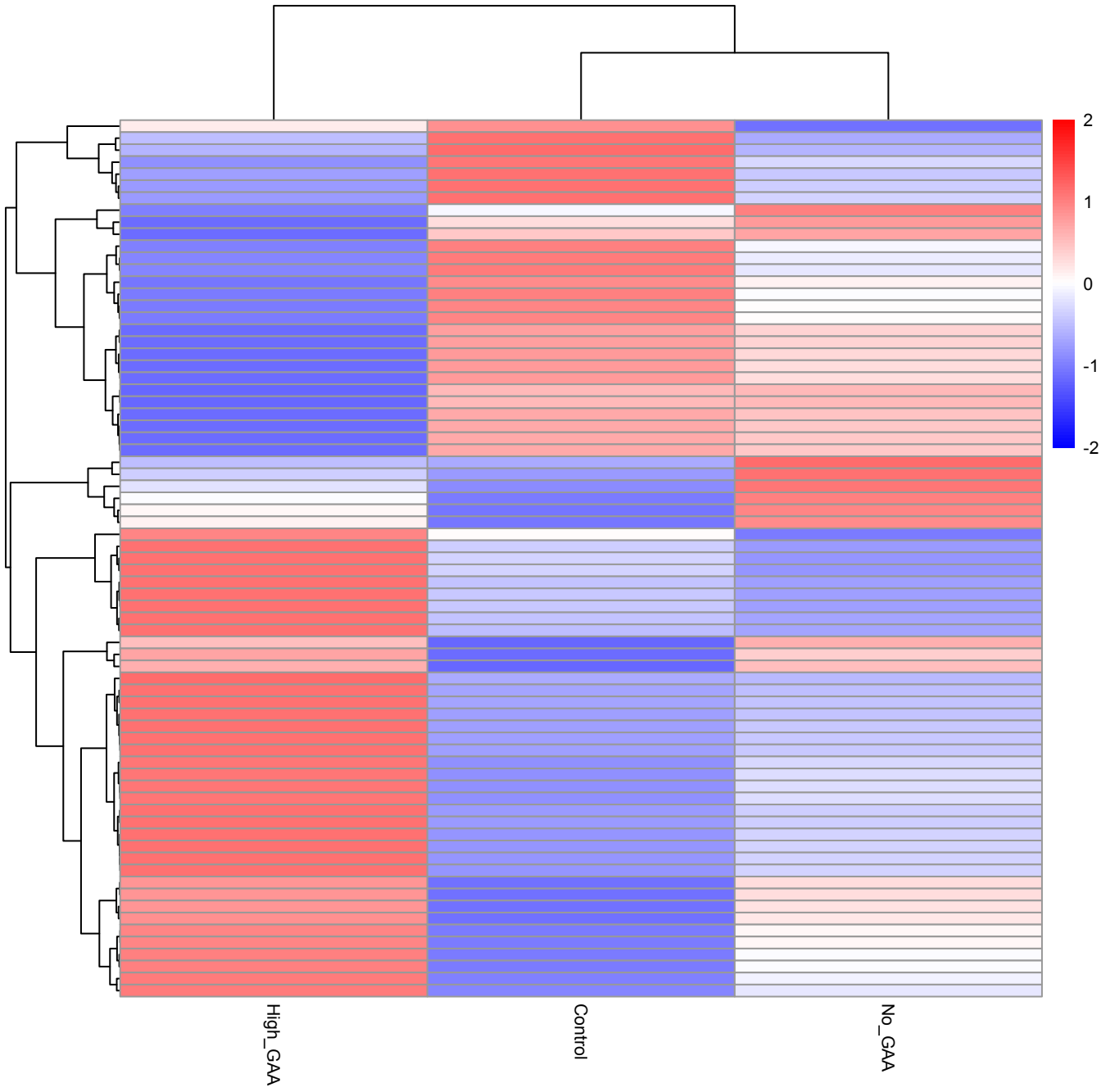

Supplement: Supplementary file 2 [file Data_Sheet_1.ZIP › Result-X101SC22030966-Z01-J001-B1-42 (quasi-targeted metabolomics)/4.MetDiffAnalysis/Heatmap_diff/Diff_Heatmap_all.cluster.pdf]

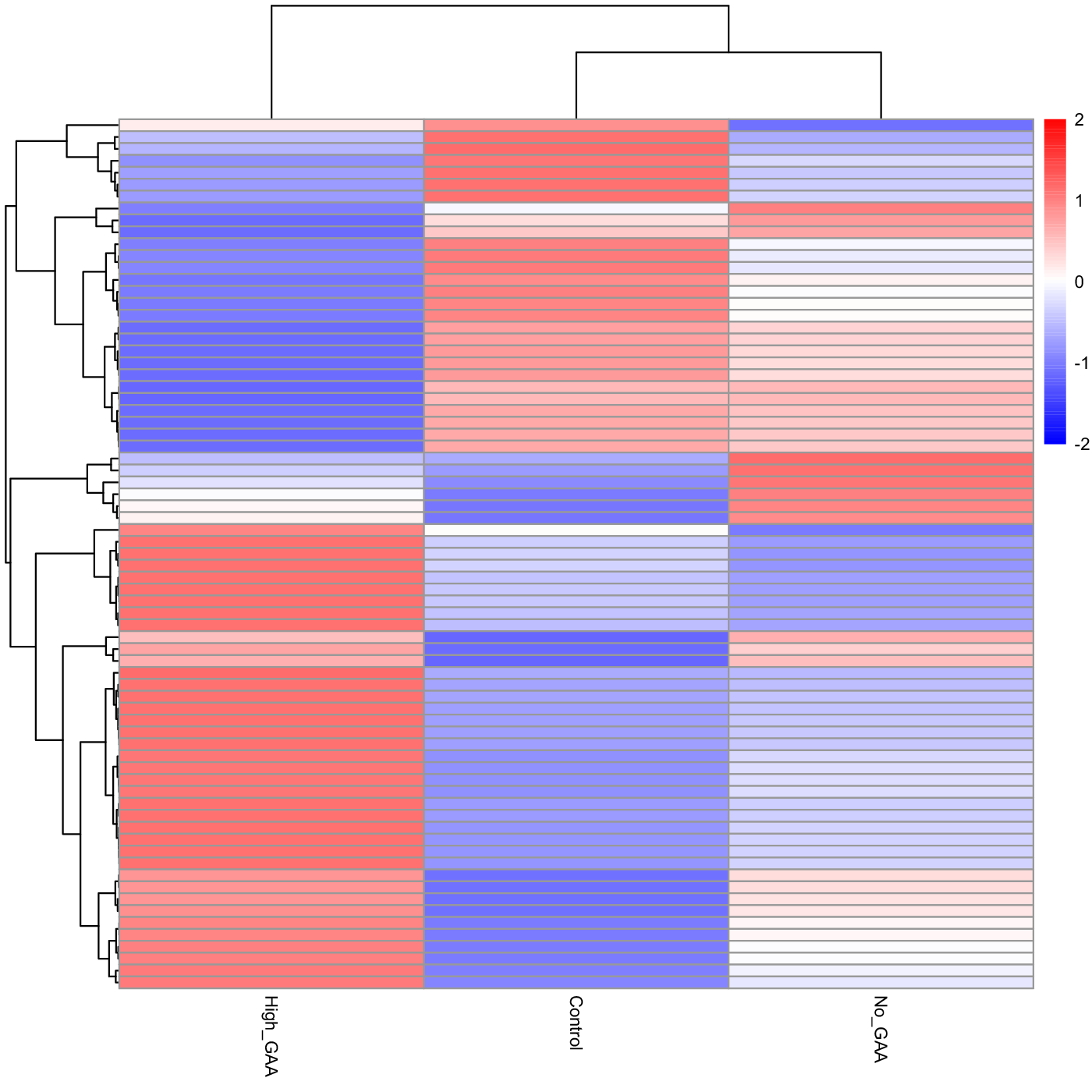

Supplement: Supplementary file 2 [file Data_Sheet_1.ZIP › Result-X101SC22030966-Z01-J001-B1-42 (quasi-targeted metabolomics)/4.MetDiffAnalysis/Heatmap_diff/Diff_Heatmap_all.cluster.png]

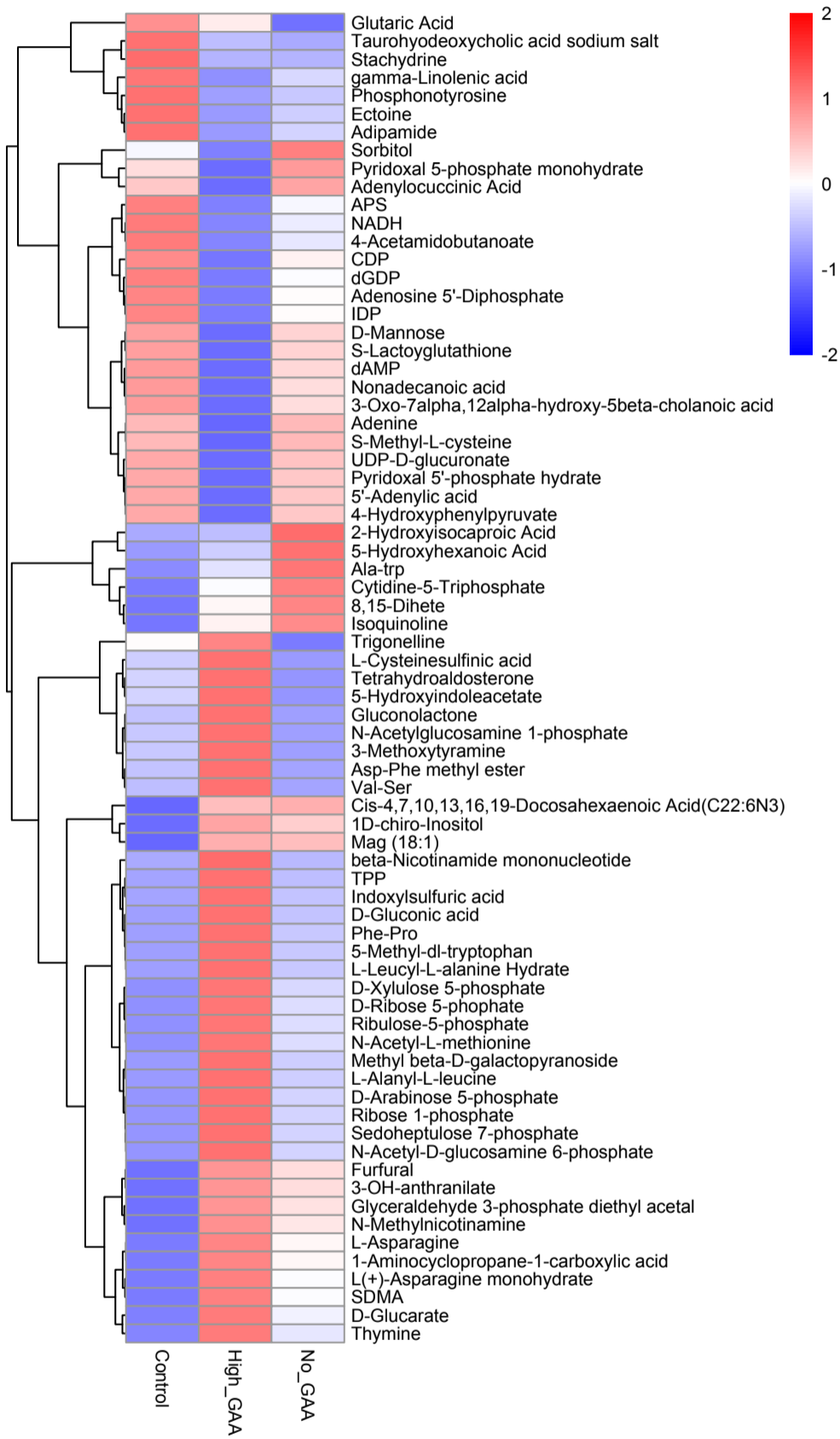

Supplement: Supplementary file 2 [file Data_Sheet_1.ZIP › Result-X101SC22030966-Z01-J001-B1-42 (quasi-targeted metabolomics)/4.MetDiffAnalysis/Heatmap_diff/Diff_Heatmap_all.detail.pdf]

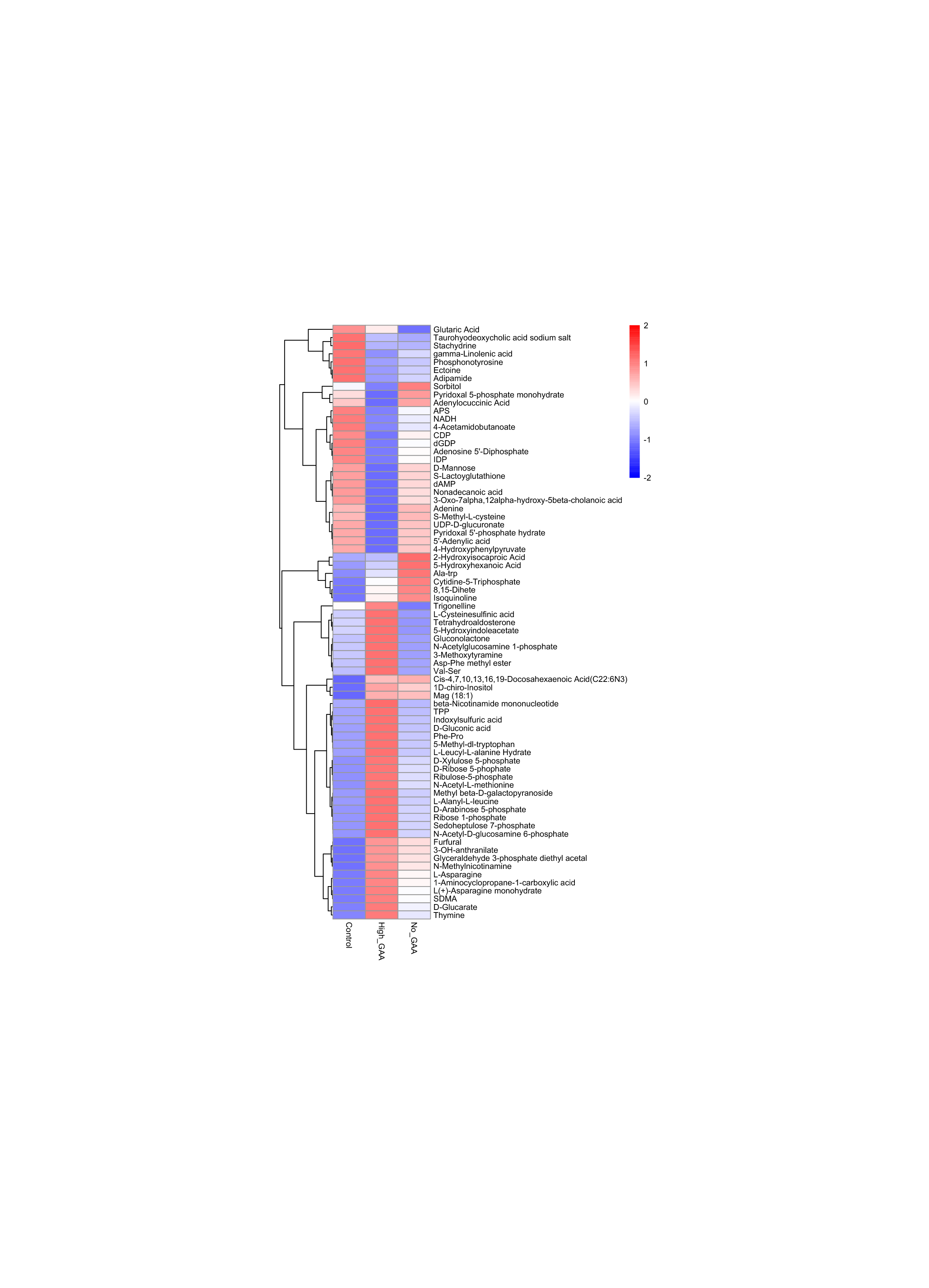

Supplement: Supplementary file 2 [file Data_Sheet_1.ZIP › Result-X101SC22030966-Z01-J001-B1-42 (quasi-targeted metabolomics)/4.MetDiffAnalysis/Heatmap_diff/Diff_Heatmap_all.detail.png]

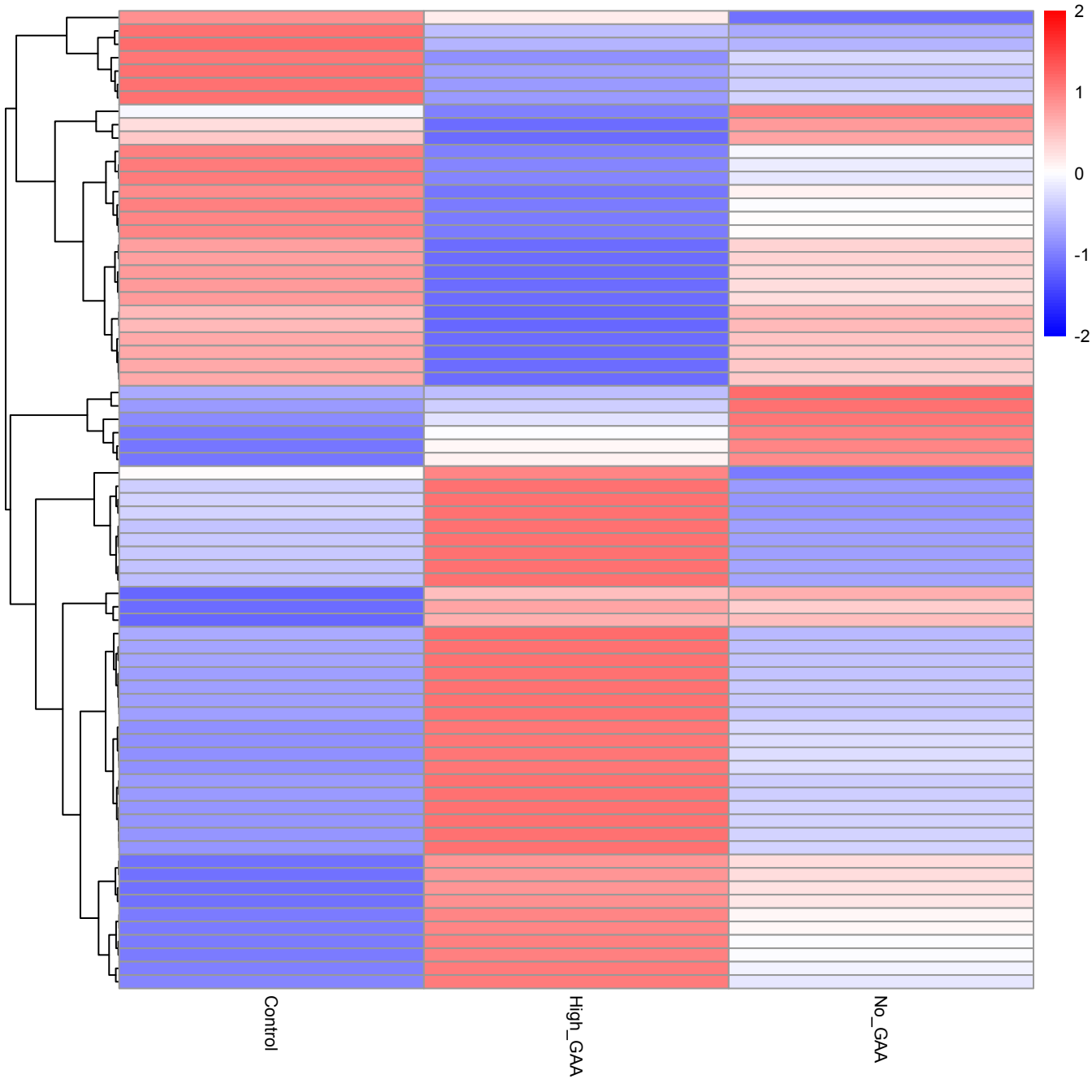

Supplement: Supplementary file 2 [file Data_Sheet_1.ZIP › Result-X101SC22030966-Z01-J001-B1-42 (quasi-targeted metabolomics)/4.MetDiffAnalysis/Heatmap_diff/Diff_Heatmap_all.pdf]

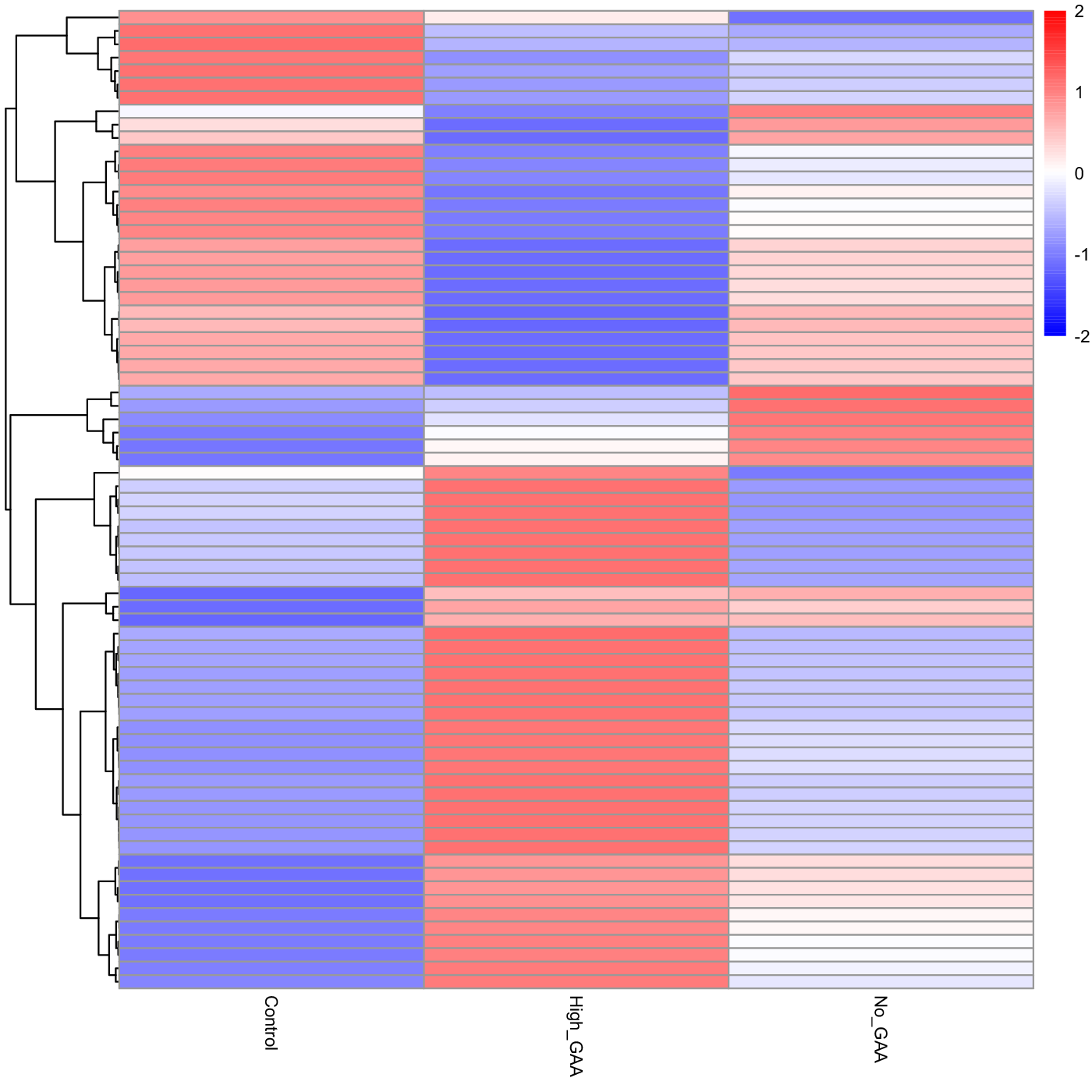

Supplement: Supplementary file 2 [file Data_Sheet_1.ZIP › Result-X101SC22030966-Z01-J001-B1-42 (quasi-targeted metabolomics)/4.MetDiffAnalysis/Heatmap_diff/Diff_Heatmap_all.png]

High\_GAA.vs.Control

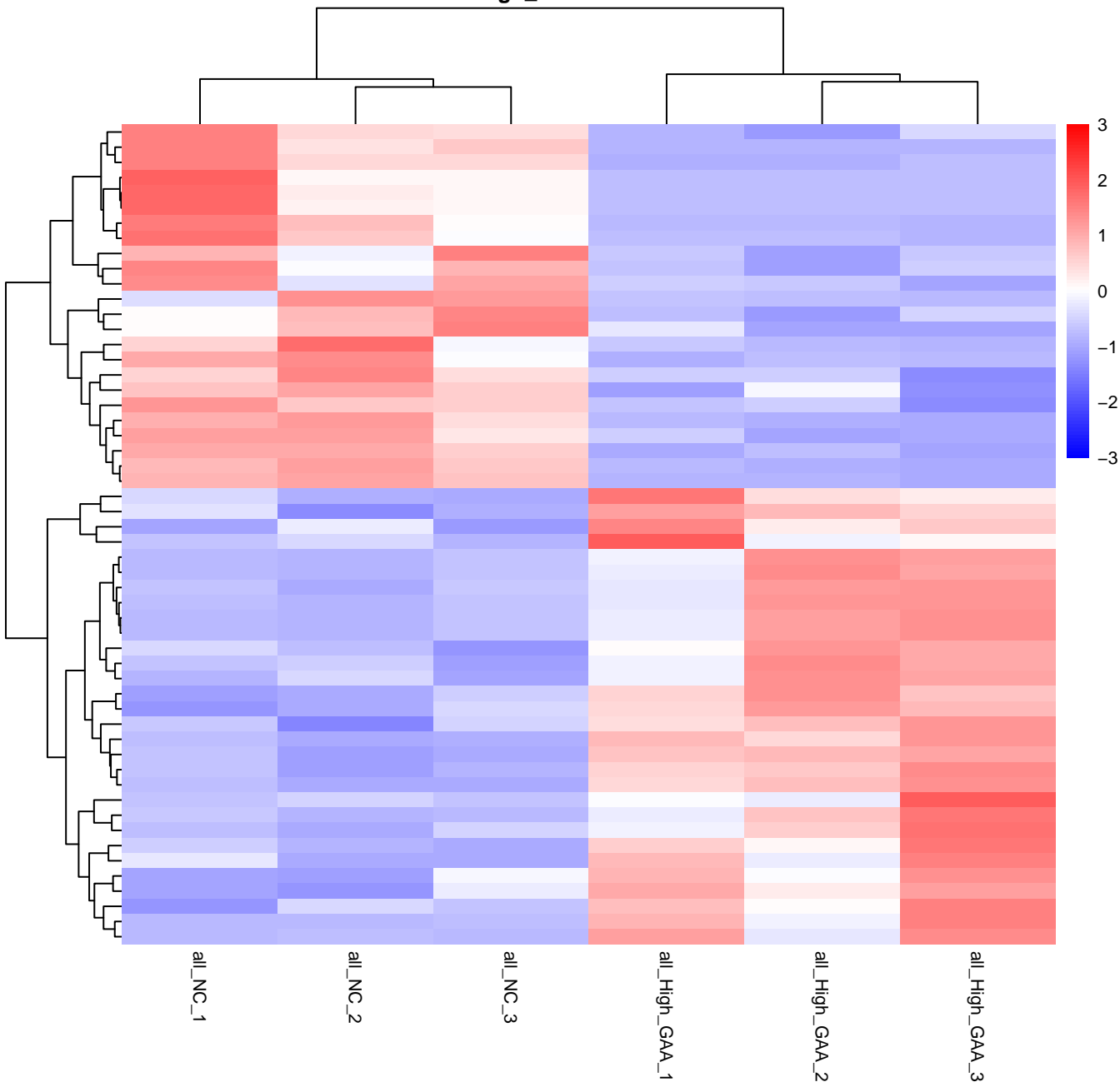

Supplement: Supplementary file 2 [file Data_Sheet_1.ZIP › Result-X101SC22030966-Z01-J001-B1-42 (quasi-targeted metabolomics)/4.MetDiffAnalysis/High_GAA.vs.Control/High_GAA.vs.Control_all_cluster_heatmap.pdf]

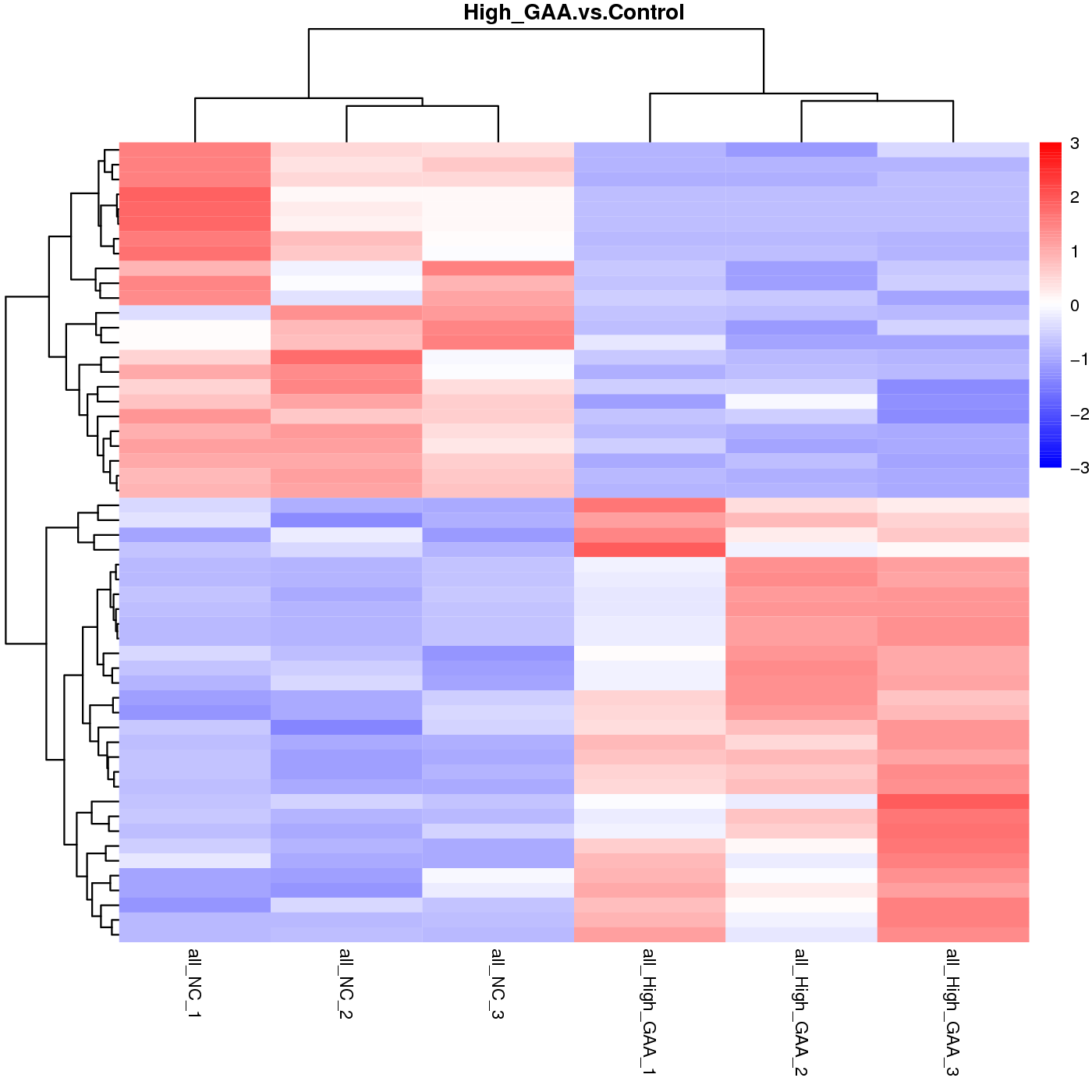

Supplement: Supplementary file 2 [file Data_Sheet_1.ZIP › Result-X101SC22030966-Z01-J001-B1-42 (quasi-targeted metabolomics)/4.MetDiffAnalysis/High_GAA.vs.Control/High_GAA.vs.Control_all_cluster_heatmap.png]

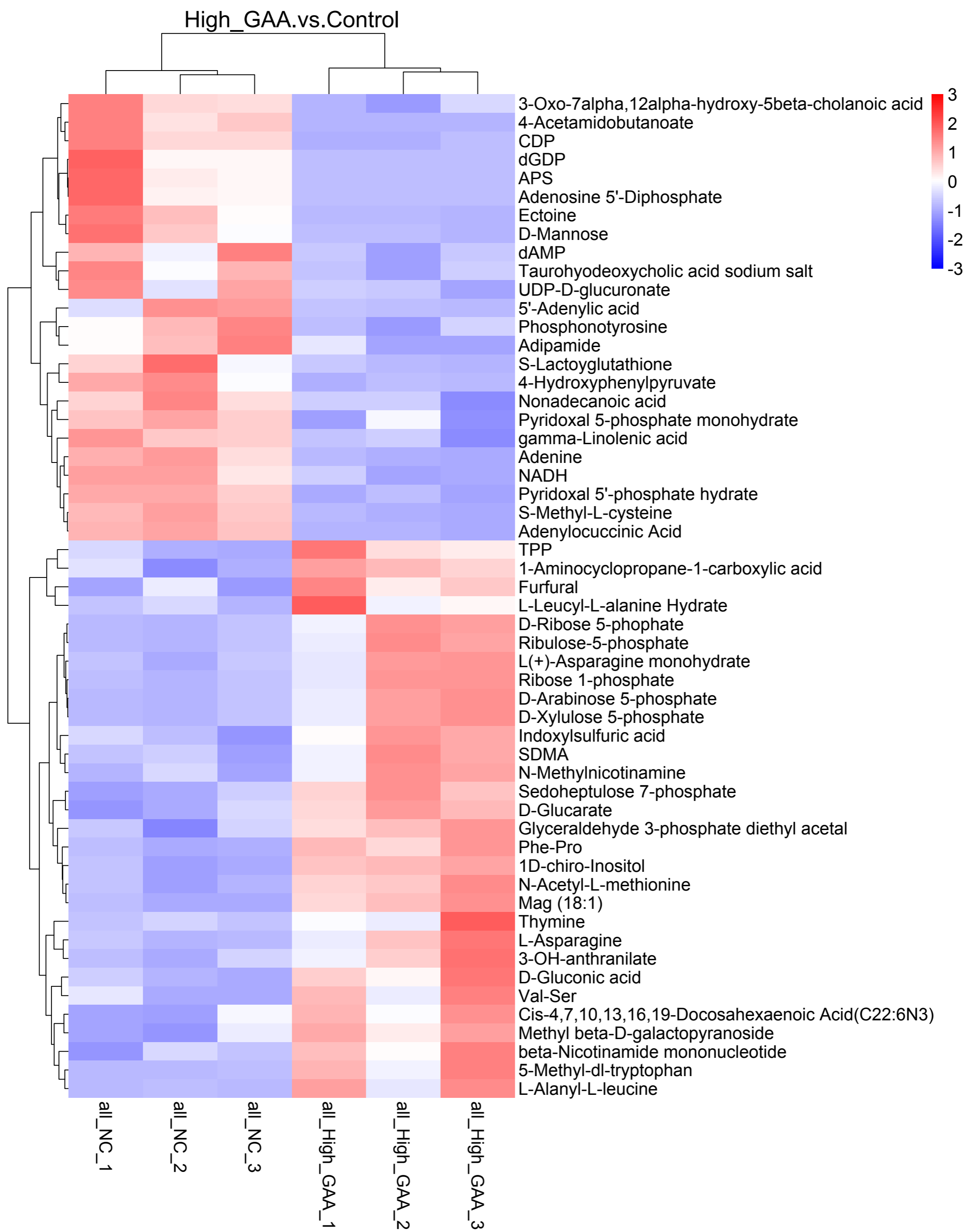

Supplement: Supplementary file 2 [file Data_Sheet_1.ZIP › Result-X101SC22030966-Z01-J001-B1-42 (quasi-targeted metabolomics)/4.MetDiffAnalysis/High_GAA.vs.Control/High_GAA.vs.Control_all_cluster_heatmap_detail.pdf]

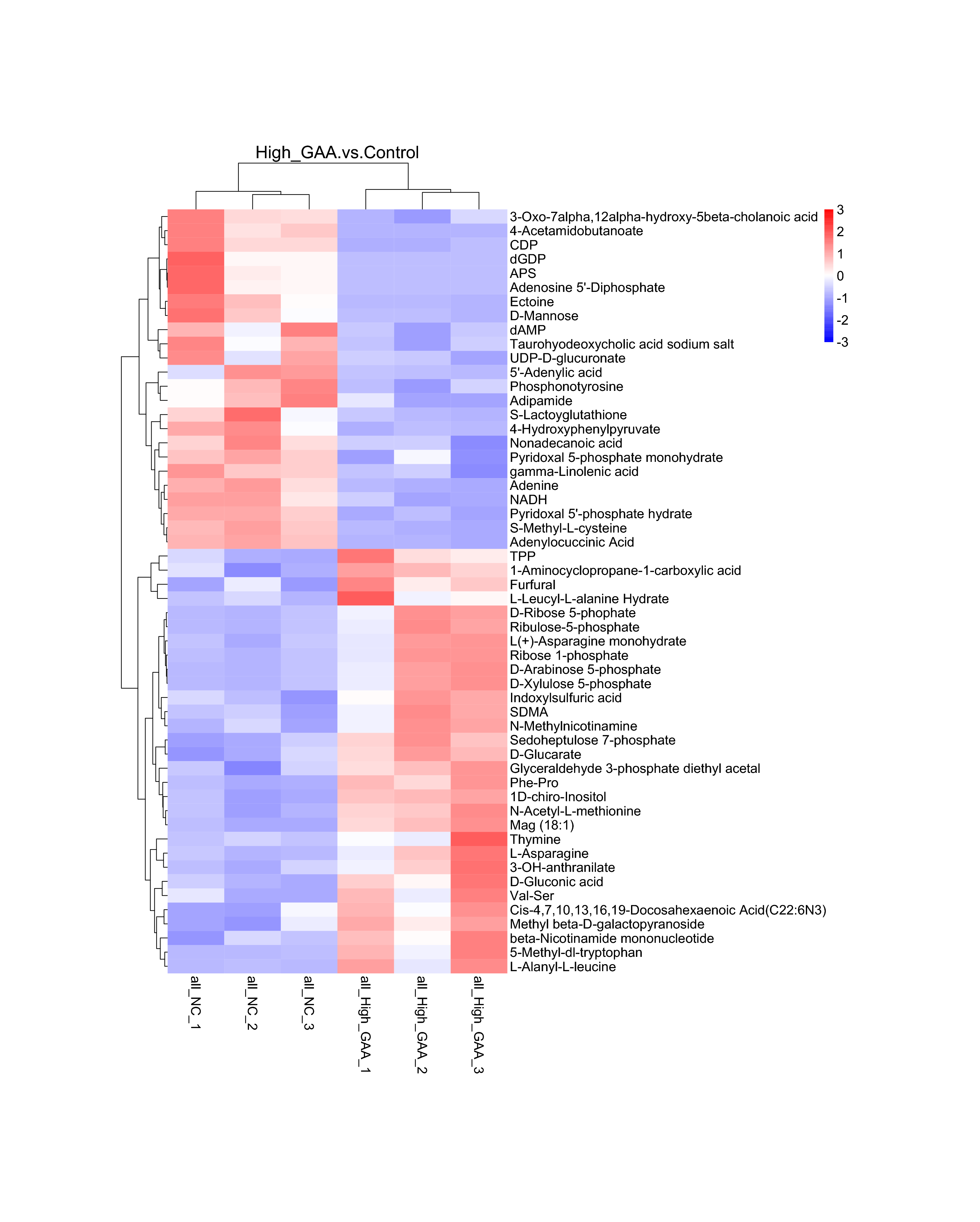

Supplement: Supplementary file 2 [file Data_Sheet_1.ZIP › Result-X101SC22030966-Z01-J001-B1-42 (quasi-targeted metabolomics)/4.MetDiffAnalysis/High_GAA.vs.Control/High_GAA.vs.Control_all_cluster_heatmap_detail.png]

High\_GAA.vs.Control

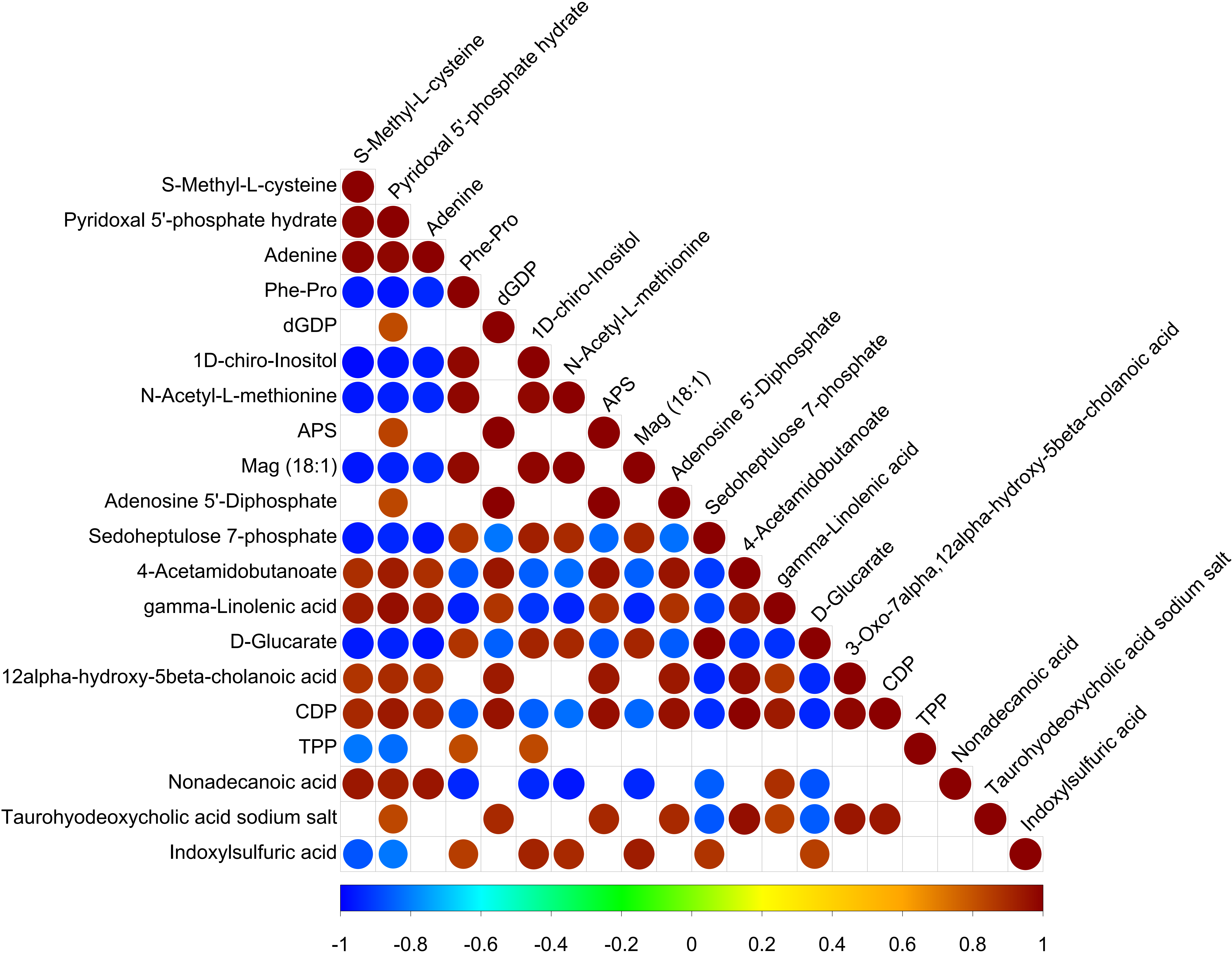

Supplement: Supplementary file 2 [file Data_Sheet_1.ZIP › Result-X101SC22030966-Z01-J001-B1-42 (quasi-targeted metabolomics)/4.MetDiffAnalysis/High_GAA.vs.Control/High_GAA.vs.Control_all_corr.pdf]

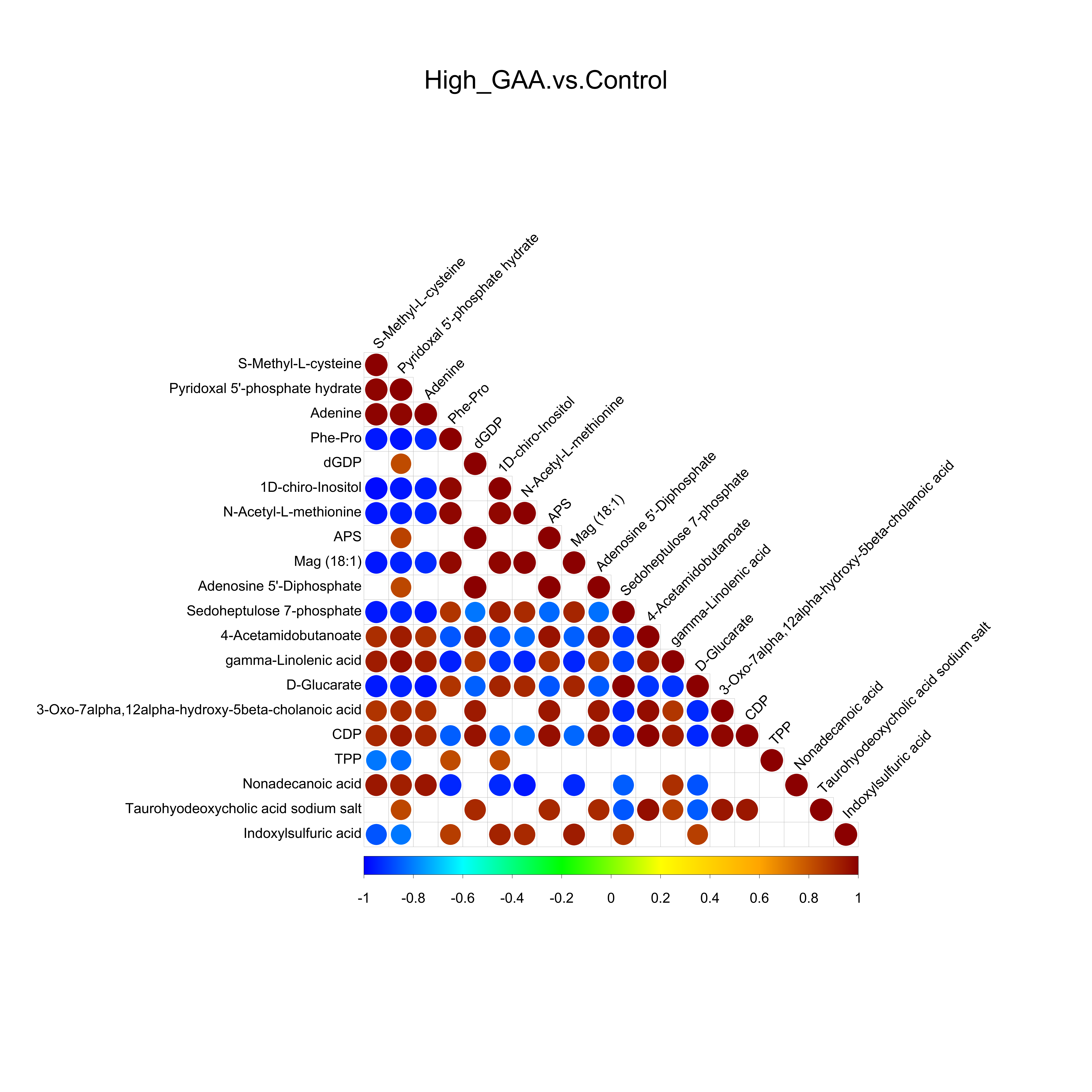

Supplement: Supplementary file 2 [file Data_Sheet_1.ZIP › Result-X101SC22030966-Z01-J001-B1-42 (quasi-targeted metabolomics)/4.MetDiffAnalysis/High_GAA.vs.Control/High_GAA.vs.Control_all_corr.png]

High\_GAA.vs.Control

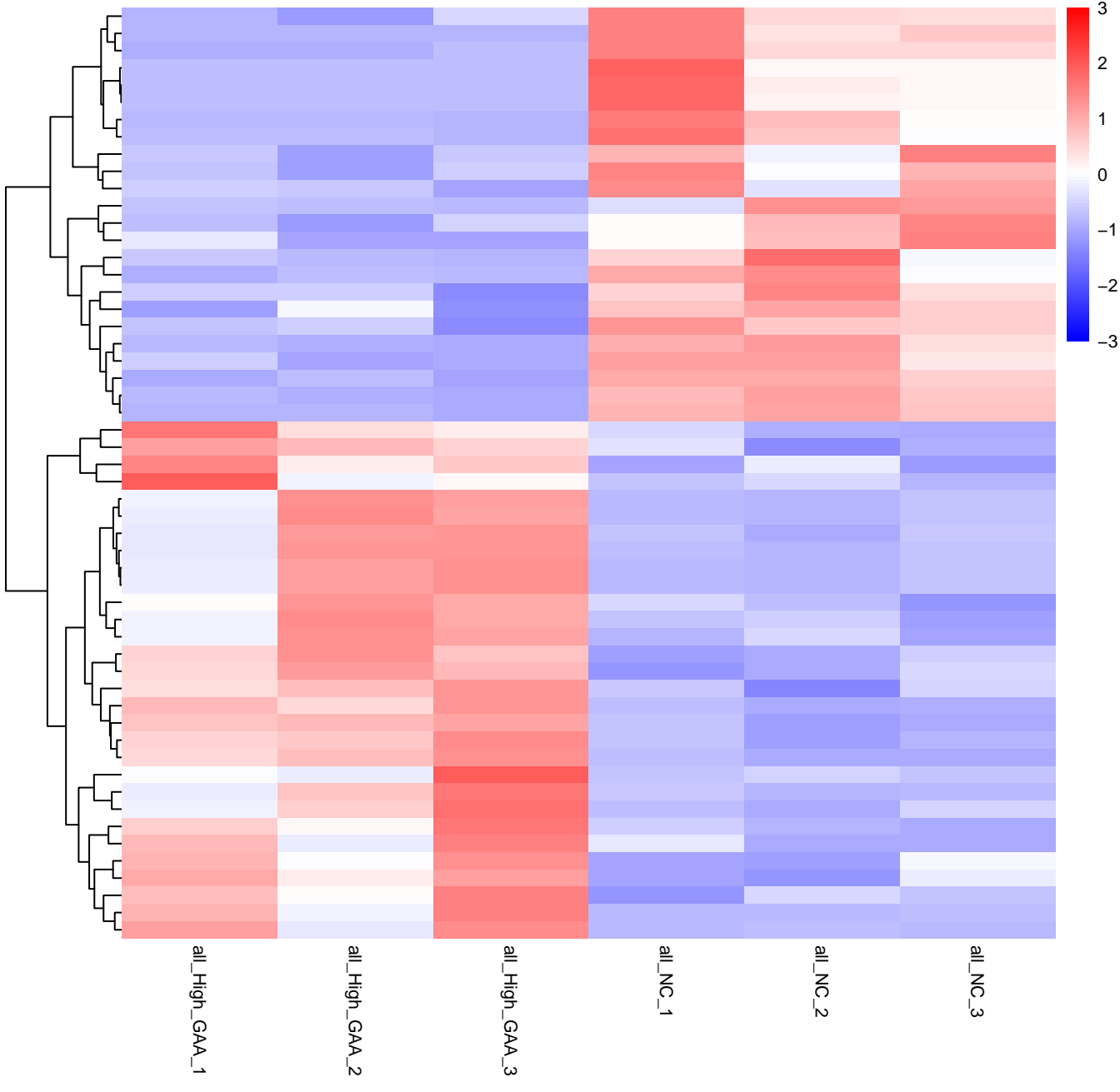

Supplement: Supplementary file 2 [file Data_Sheet_1.ZIP › Result-X101SC22030966-Z01-J001-B1-42 (quasi-targeted metabolomics)/4.MetDiffAnalysis/High_GAA.vs.Control/High_GAA.vs.Control_all_heatmap.pdf]

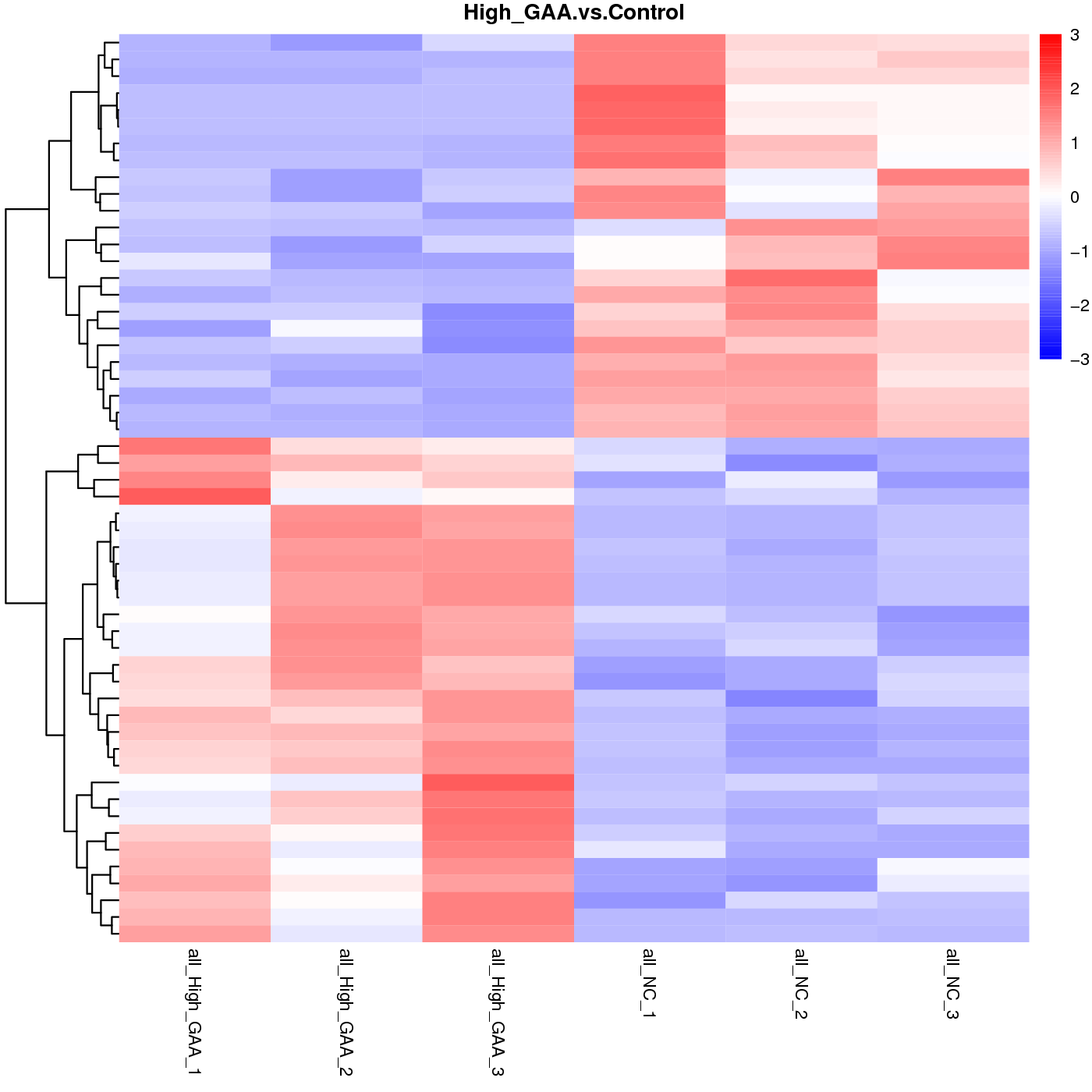

Supplement: Supplementary file 2 [file Data_Sheet_1.ZIP › Result-X101SC22030966-Z01-J001-B1-42 (quasi-targeted metabolomics)/4.MetDiffAnalysis/High_GAA.vs.Control/High_GAA.vs.Control_all_heatmap.png]

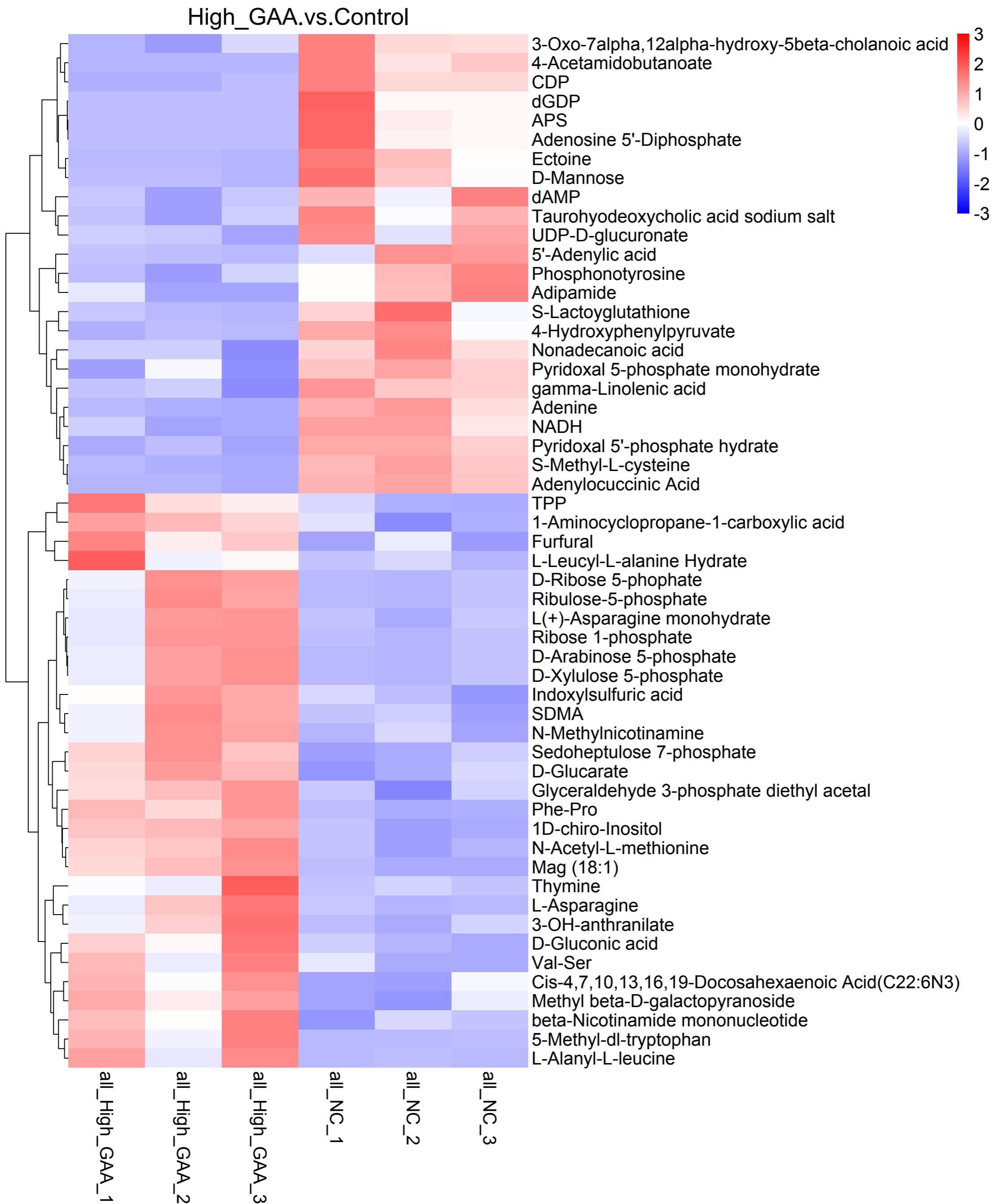

Supplement: Supplementary file 2 [file Data_Sheet_1.ZIP › Result-X101SC22030966-Z01-J001-B1-42 (quasi-targeted metabolomics)/4.MetDiffAnalysis/High_GAA.vs.Control/High_GAA.vs.Control_all_heatmap_detail.pdf]

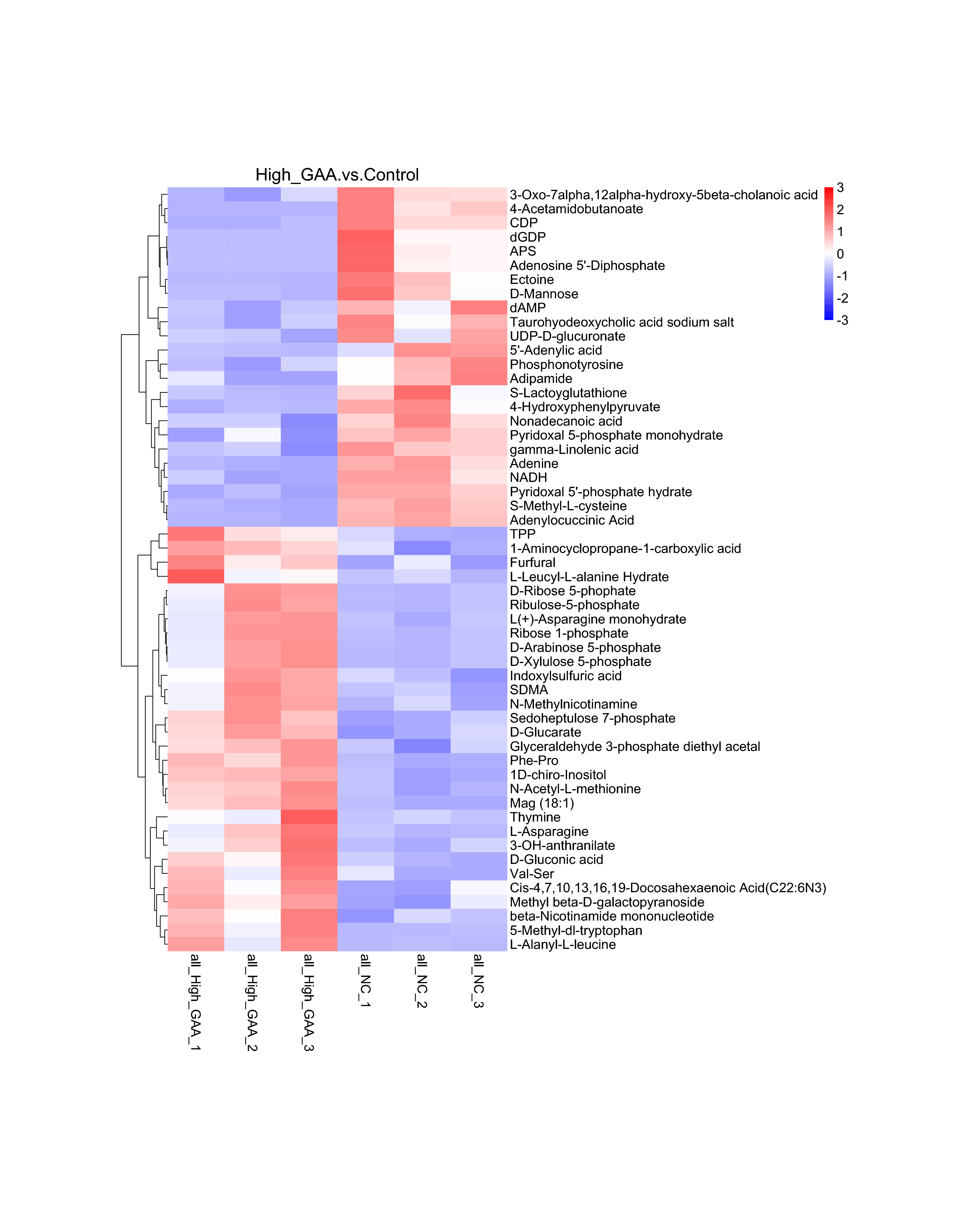

Supplement: Supplementary file 2 [file Data_Sheet_1.ZIP › Result-X101SC22030966-Z01-J001-B1-42 (quasi-targeted metabolomics)/4.MetDiffAnalysis/High_GAA.vs.Control/High_GAA.vs.Control_all_heatmap_detail.png]

High\_GAA.vs.Control

Metabolites

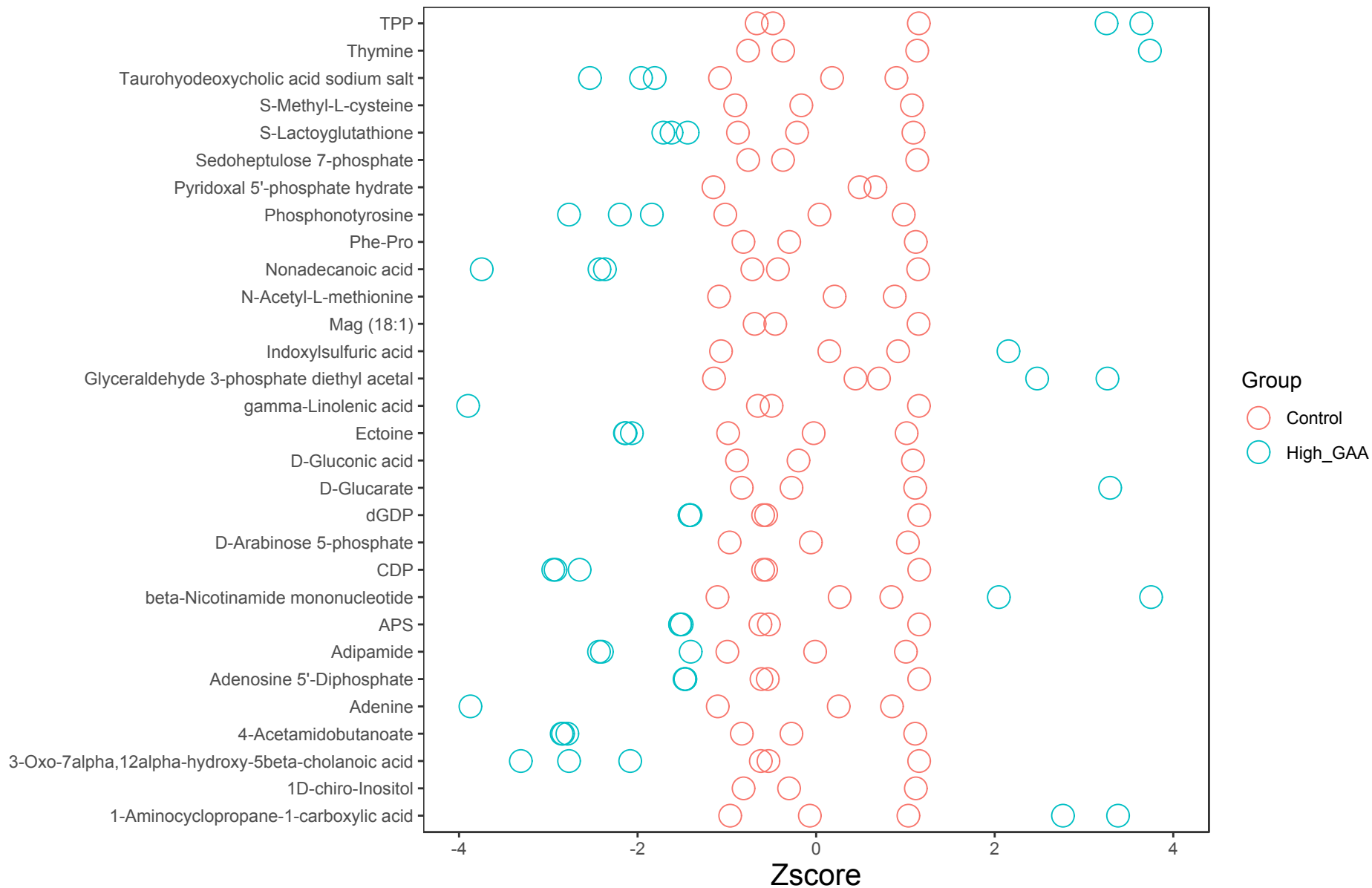

Supplement: Supplementary file 2 [file Data_Sheet_1.ZIP › Result-X101SC22030966-Z01-J001-B1-42 (quasi-targeted metabolomics)/4.MetDiffAnalysis/High_GAA.vs.Control/High_GAA.vs.Control_all_zscore.pdf]

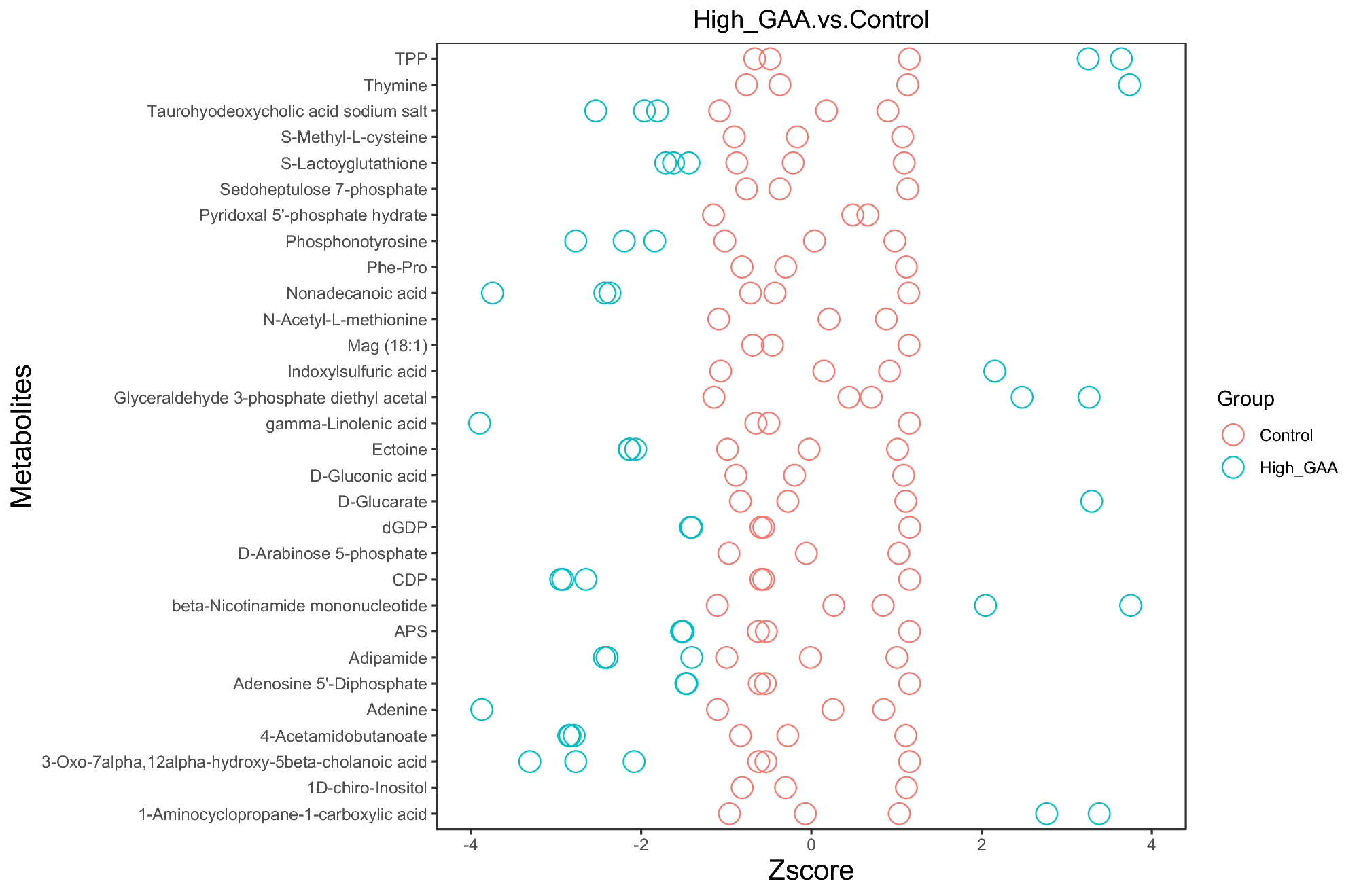

Supplement: Supplementary file 2 [file Data_Sheet_1.ZIP › Result-X101SC22030966-Z01-J001-B1-42 (quasi-targeted metabolomics)/4.MetDiffAnalysis/High_GAA.vs.Control/High_GAA.vs.Control_all_zscore.png]

High\_GAA.vs.Control

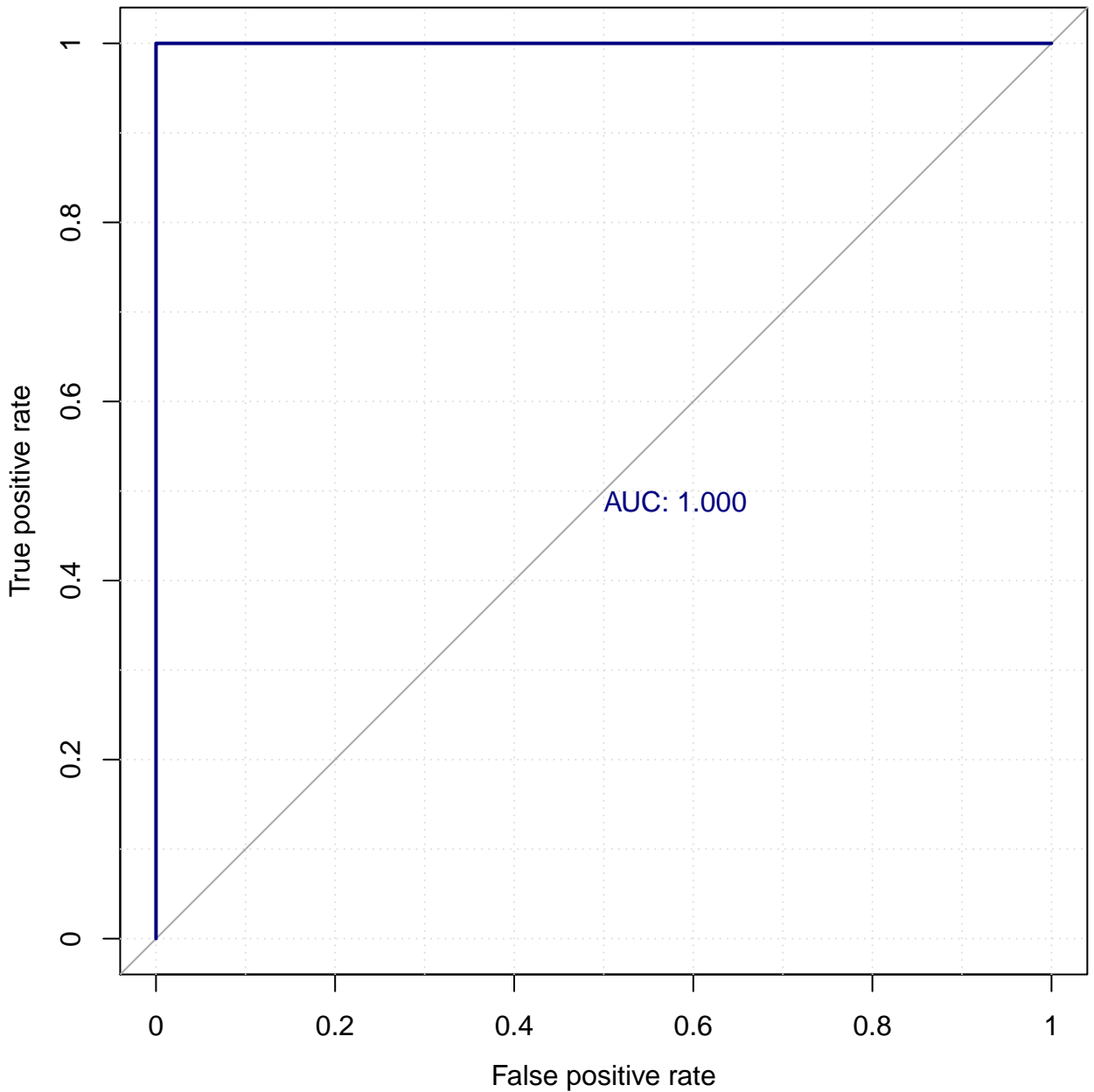

Supplement: Supplementary file 2 [file Data_Sheet_1.ZIP › Result-X101SC22030966-Z01-J001-B1-42 (quasi-targeted metabolomics)/4.MetDiffAnalysis/High_GAA.vs.Control/ROC_all/Com_10_neg_ROC.pdf]

High\_GAA.vs.Control

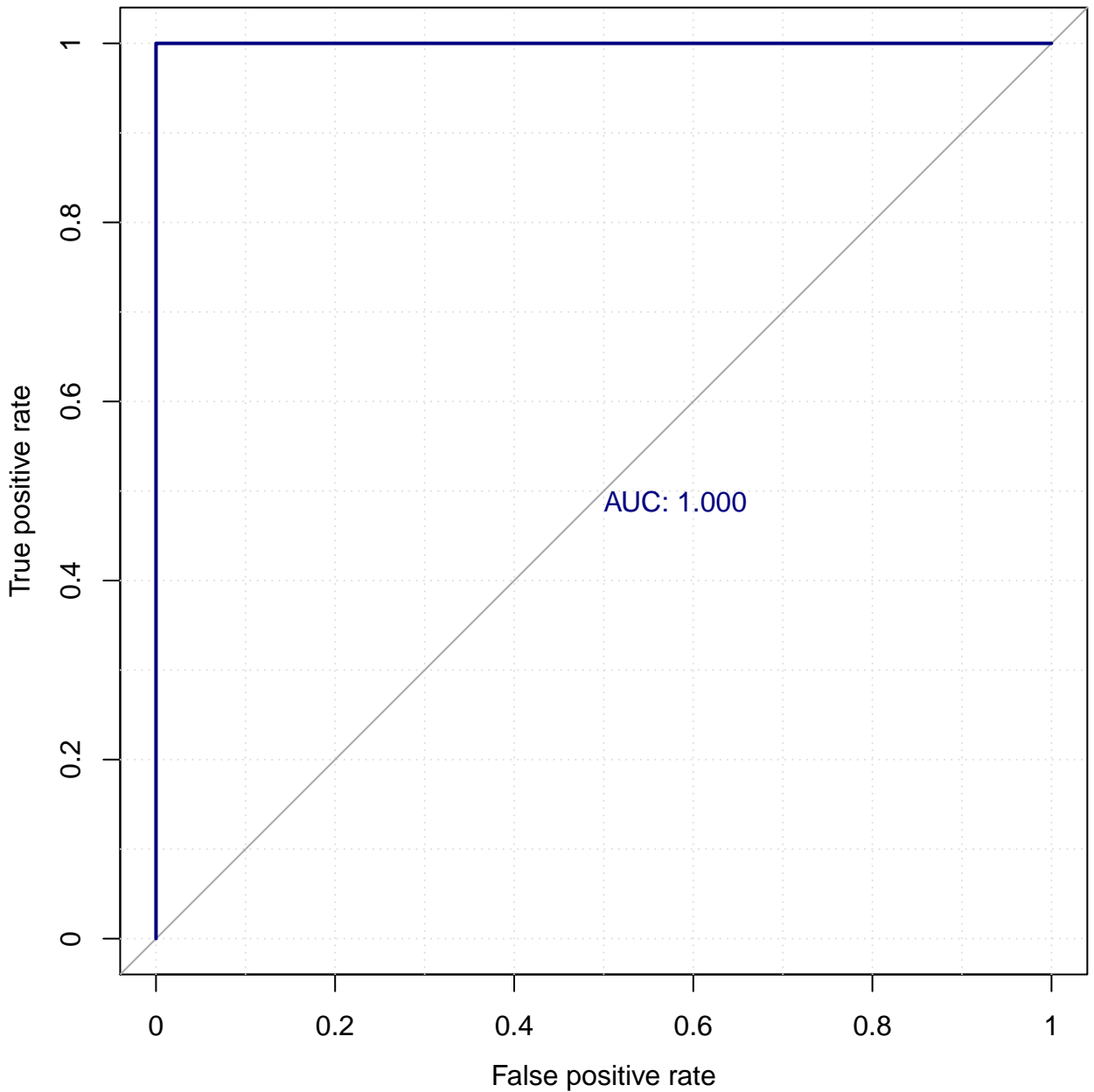

Supplement: Supplementary file 2 [file Data_Sheet_1.ZIP › Result-X101SC22030966-Z01-J001-B1-42 (quasi-targeted metabolomics)/4.MetDiffAnalysis/High_GAA.vs.Control/ROC_all/Com_140_neg_ROC.pdf]

High\_GAA.vs.Control

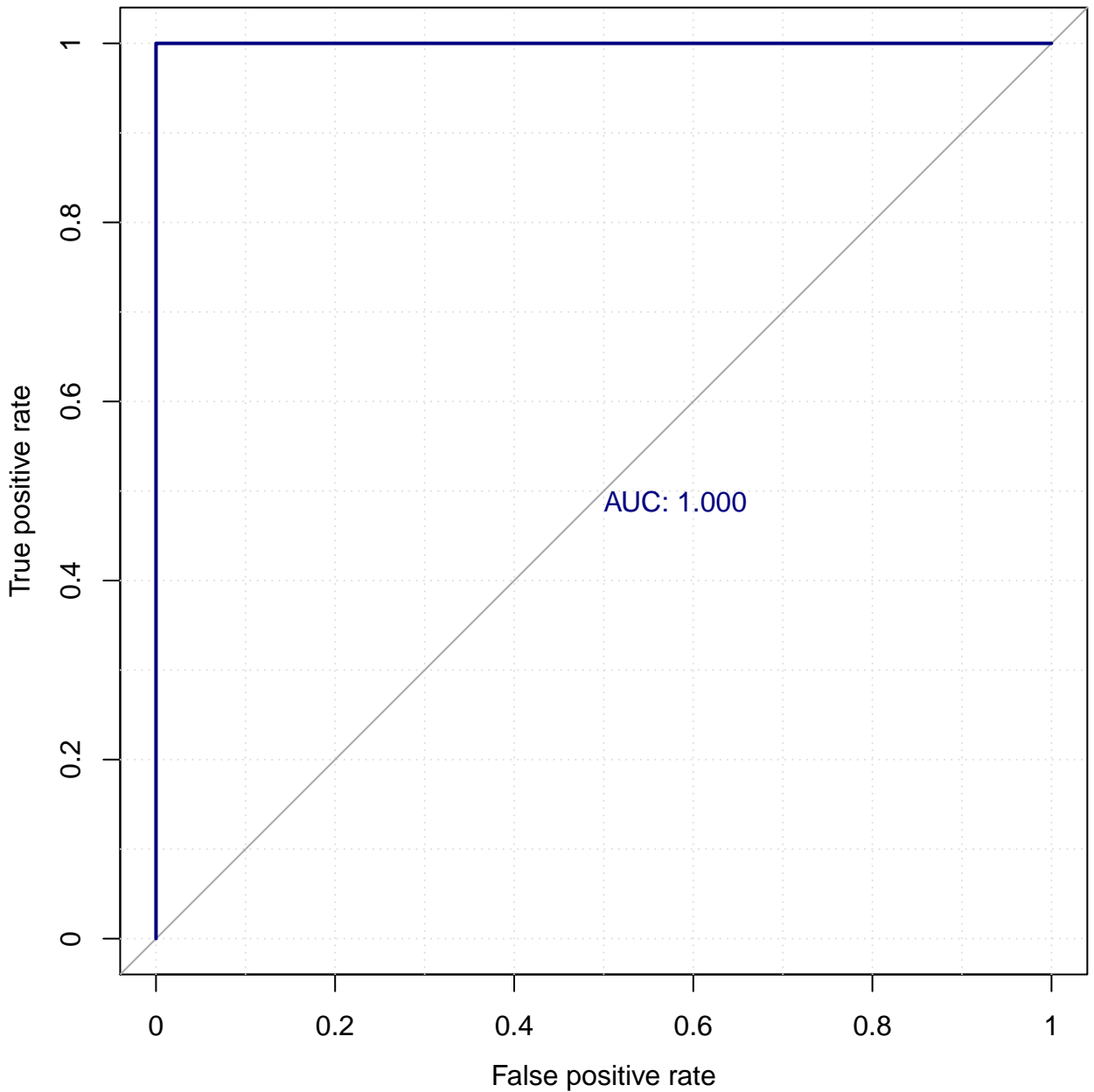

Supplement: Supplementary file 2 [file Data_Sheet_1.ZIP › Result-X101SC22030966-Z01-J001-B1-42 (quasi-targeted metabolomics)/4.MetDiffAnalysis/High_GAA.vs.Control/ROC_all/Com_148_neg_ROC.pdf]

High\_GAA.vs.Control

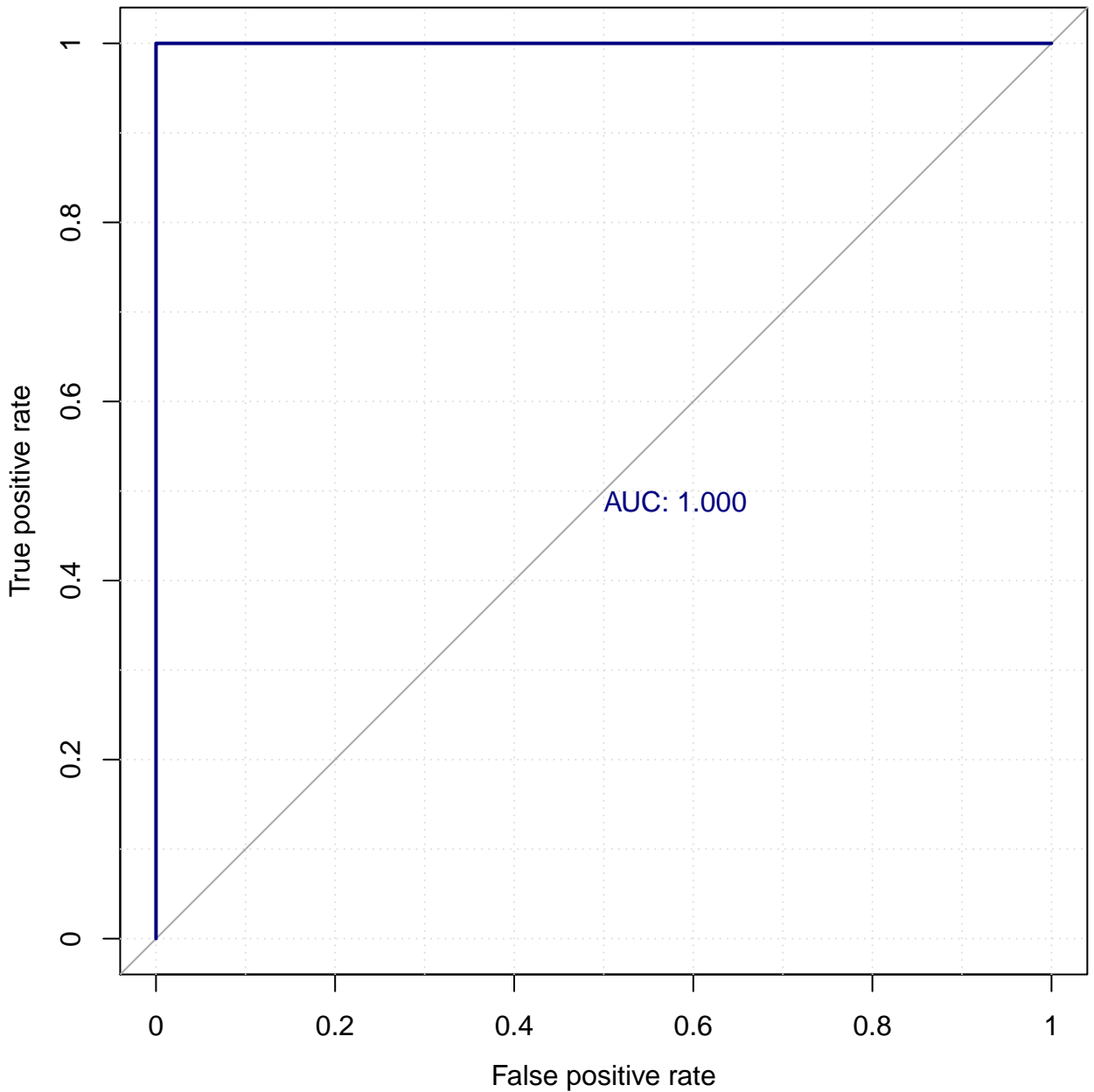

Supplement: Supplementary file 2 [file Data_Sheet_1.ZIP › Result-X101SC22030966-Z01-J001-B1-42 (quasi-targeted metabolomics)/4.MetDiffAnalysis/High_GAA.vs.Control/ROC_all/Com_15_neg_ROC.pdf]

High\_GAA.vs.Control

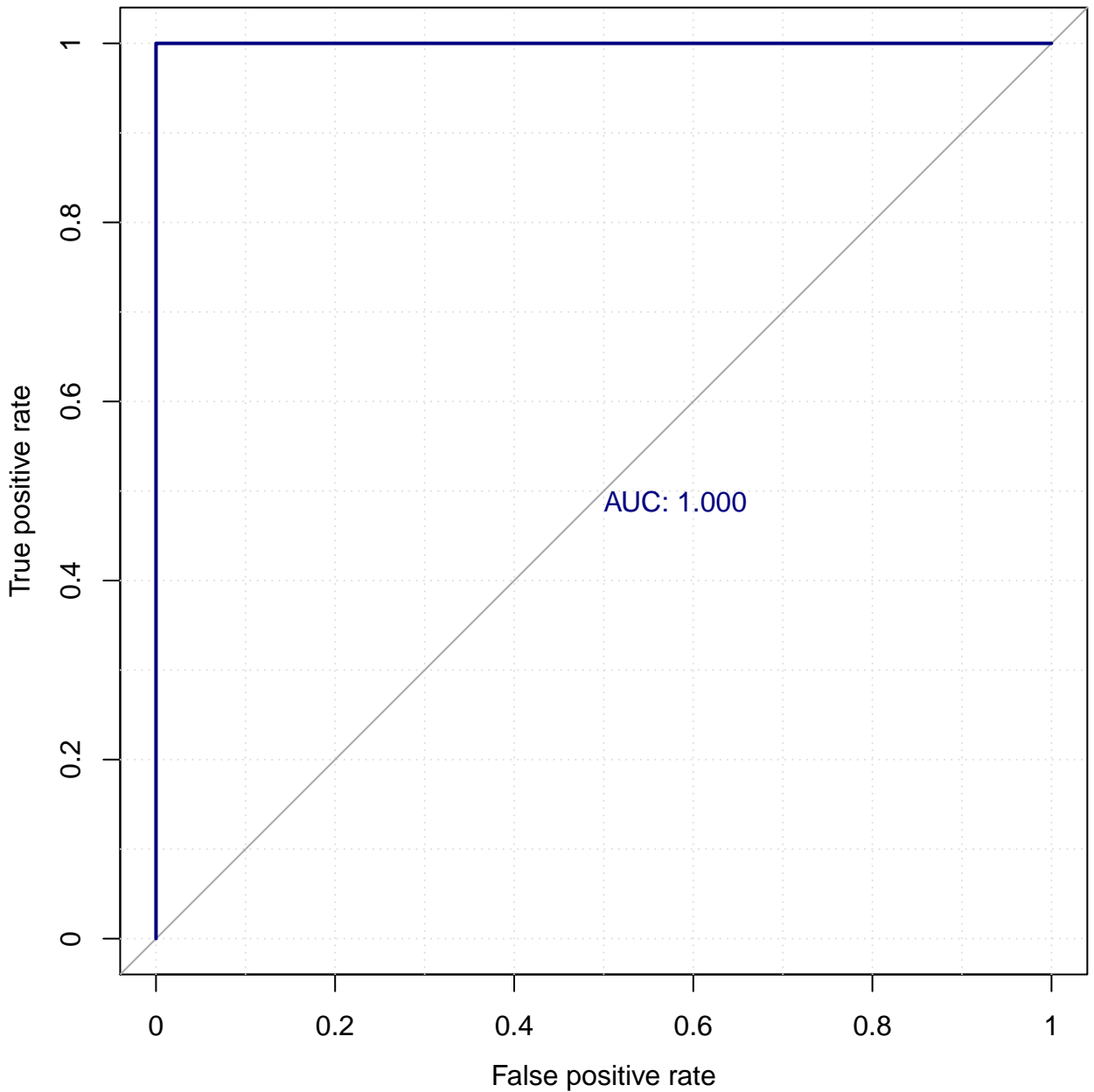

Supplement: Supplementary file 2 [file Data_Sheet_1.ZIP › Result-X101SC22030966-Z01-J001-B1-42 (quasi-targeted metabolomics)/4.MetDiffAnalysis/High_GAA.vs.Control/ROC_all/Com_173_neg_ROC.pdf]

High\_GAA.vs.Control

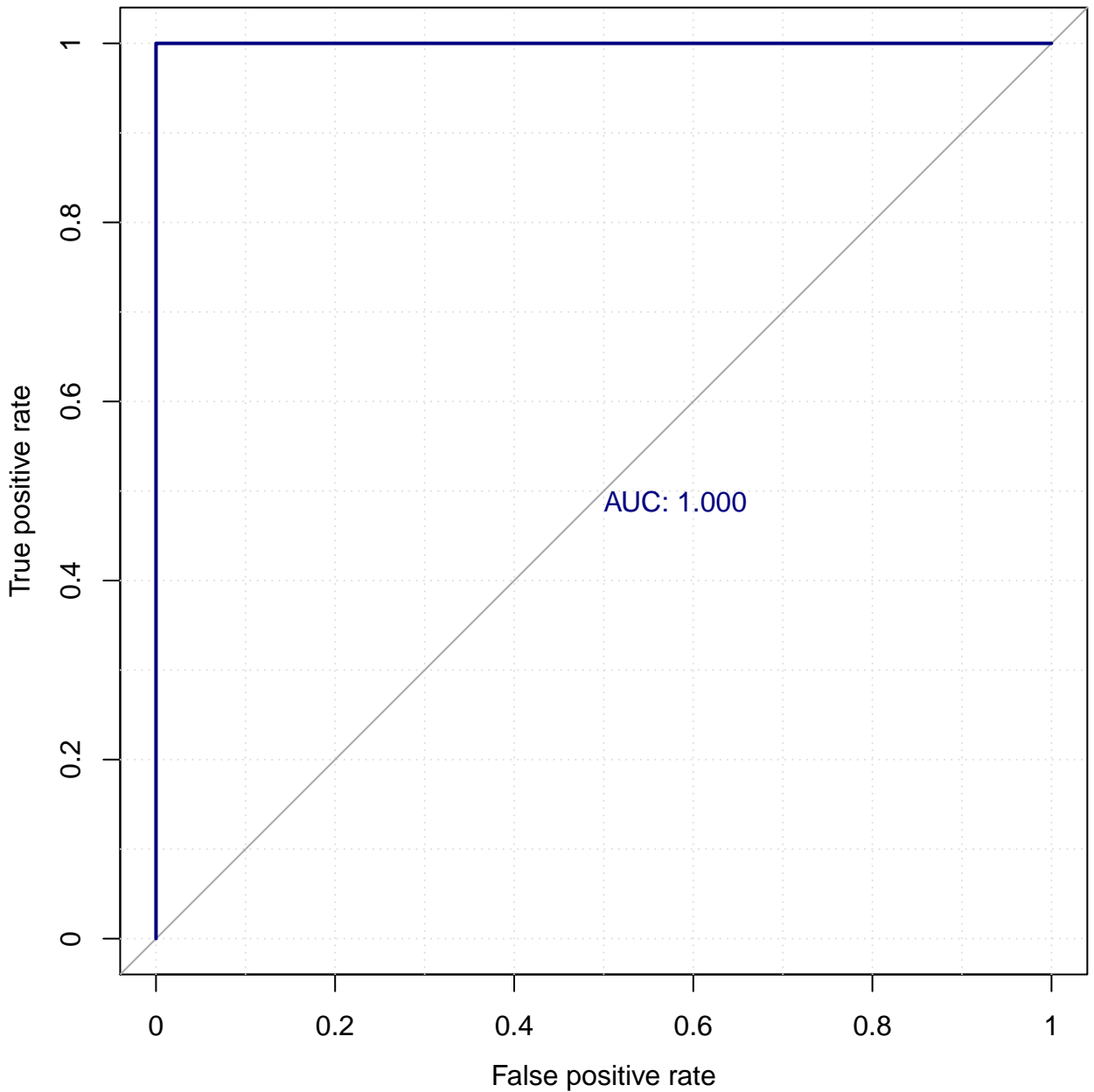

Supplement: Supplementary file 2 [file Data_Sheet_1.ZIP › Result-X101SC22030966-Z01-J001-B1-42 (quasi-targeted metabolomics)/4.MetDiffAnalysis/High_GAA.vs.Control/ROC_all/Com_220_neg_ROC.pdf]

High\_GAA.vs.Control

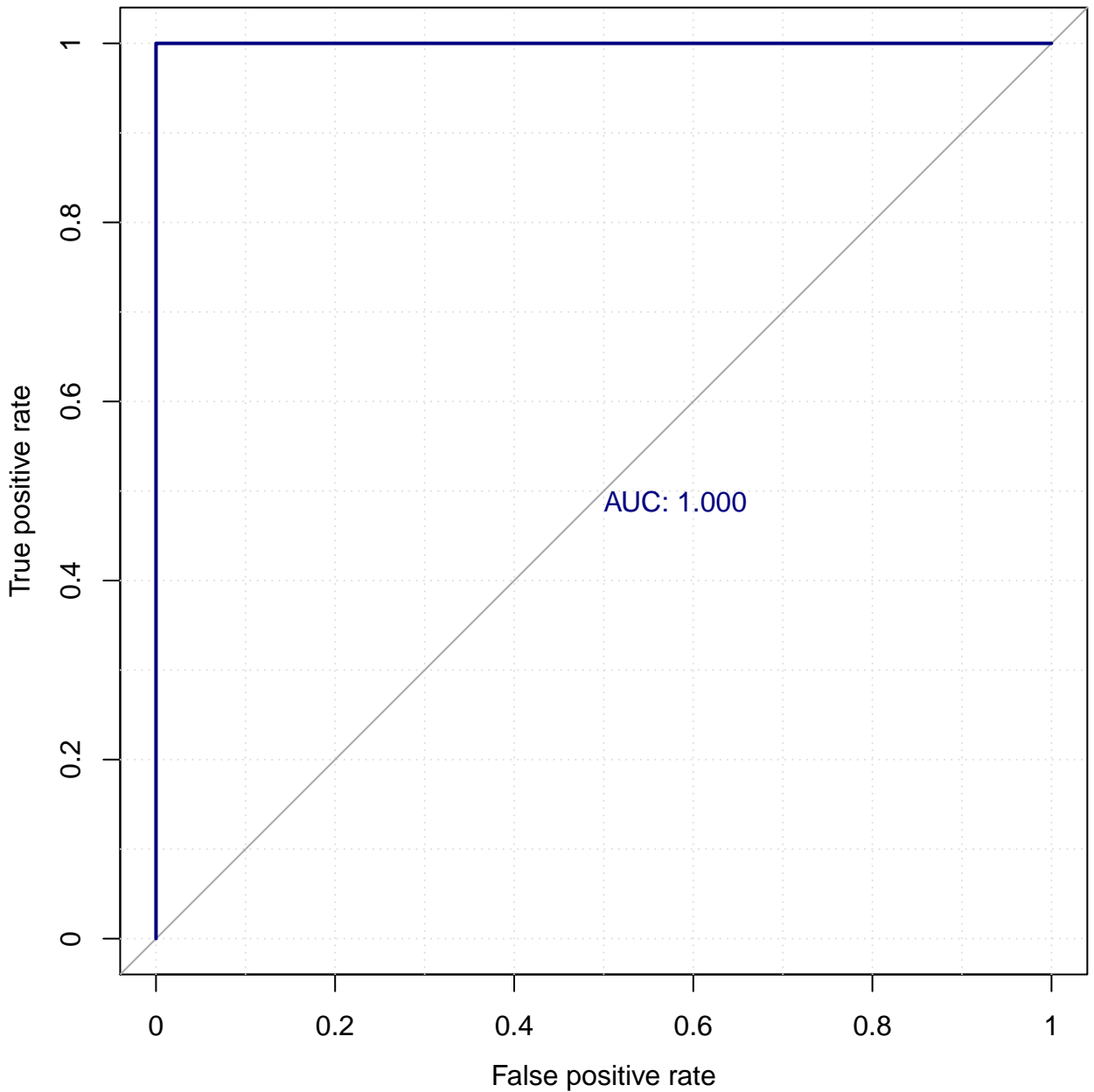

Supplement: Supplementary file 2 [file Data_Sheet_1.ZIP › Result-X101SC22030966-Z01-J001-B1-42 (quasi-targeted metabolomics)/4.MetDiffAnalysis/High_GAA.vs.Control/ROC_all/Com_228_neg_ROC.pdf]

High\_GAA.vs.Control

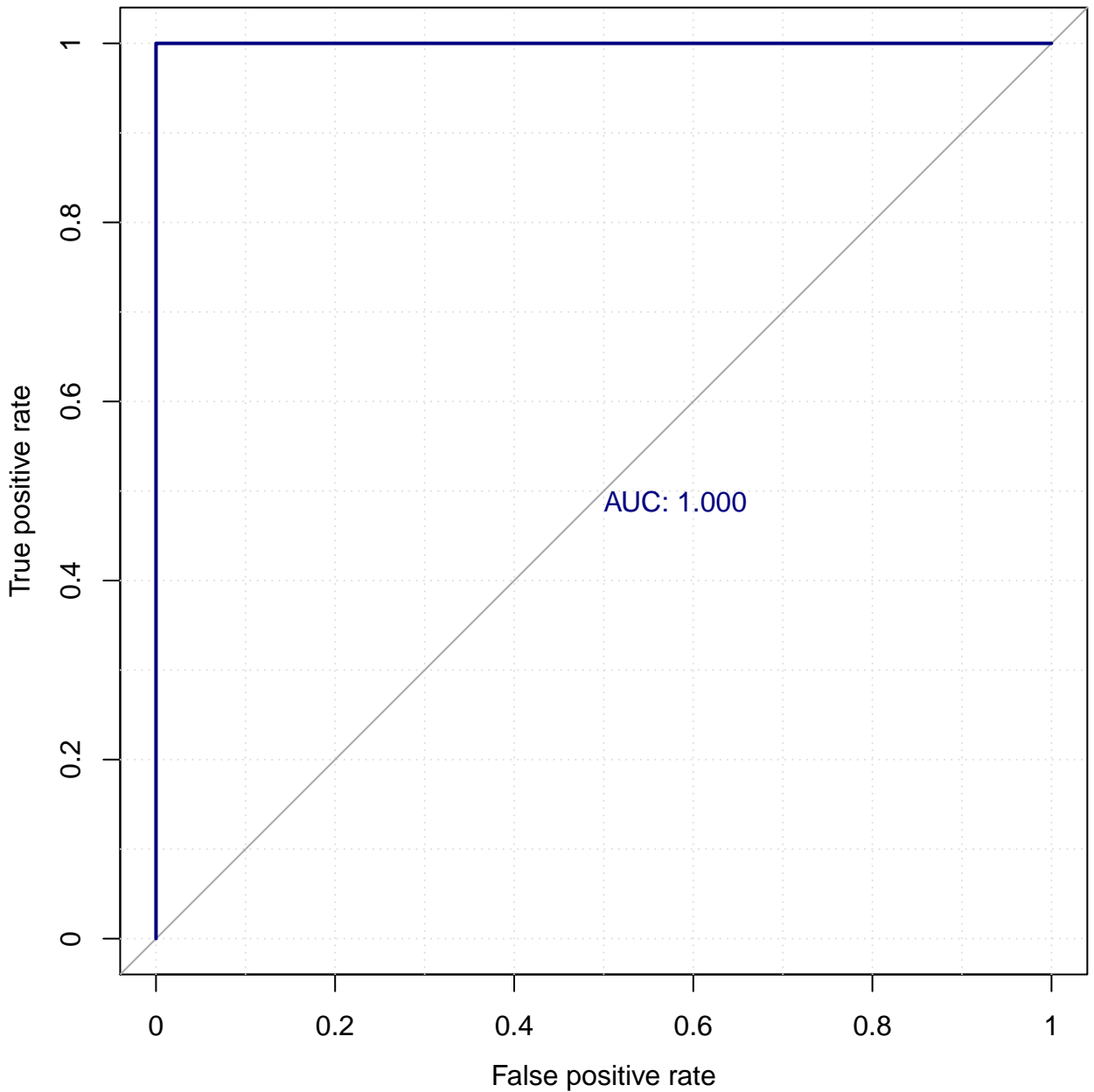

Supplement: Supplementary file 2 [file Data_Sheet_1.ZIP › Result-X101SC22030966-Z01-J001-B1-42 (quasi-targeted metabolomics)/4.MetDiffAnalysis/High_GAA.vs.Control/ROC_all/Com_247_neg_ROC.pdf]

High\_GAA.vs.Control

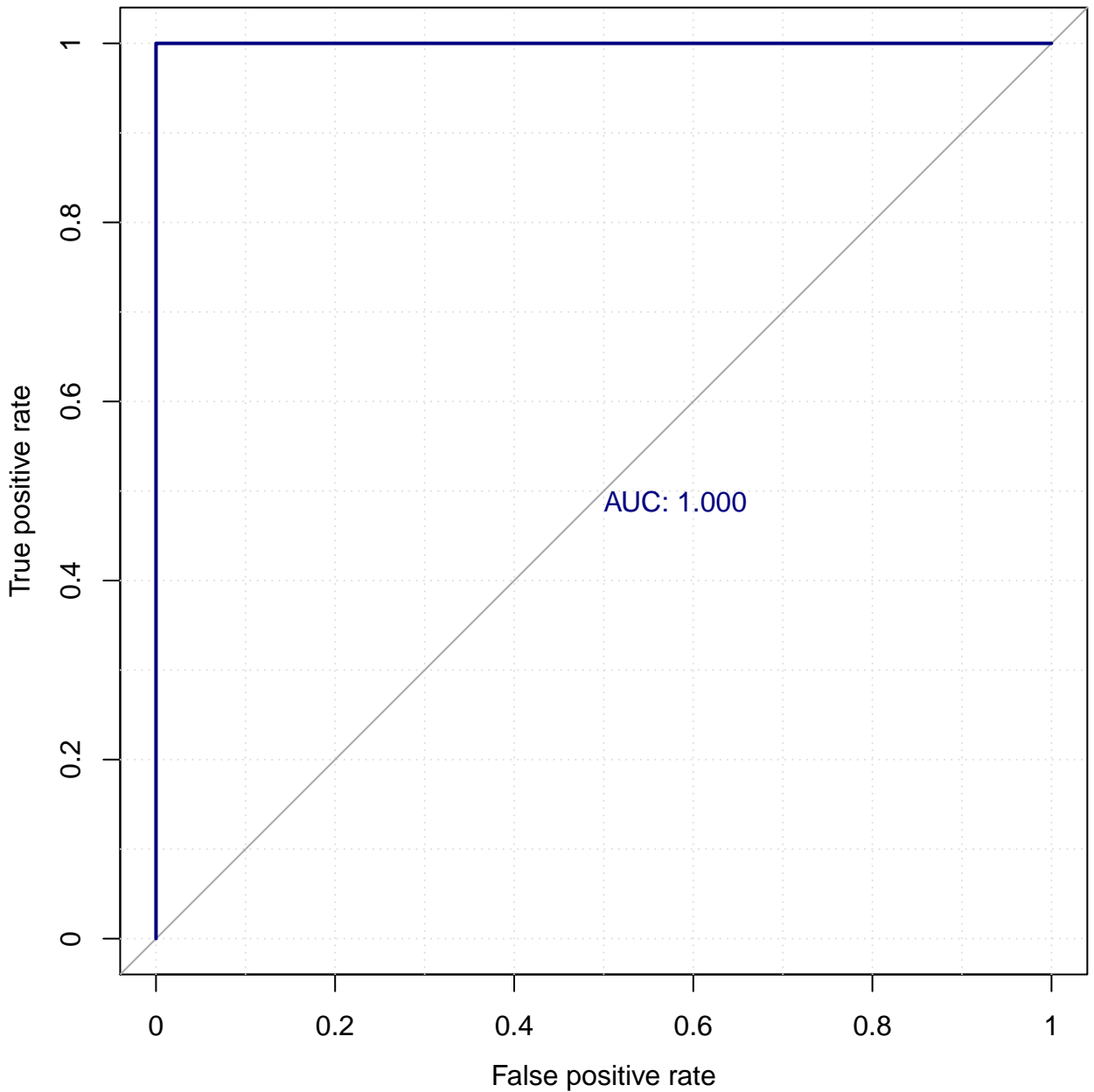

Supplement: Supplementary file 2 [file Data_Sheet_1.ZIP › Result-X101SC22030966-Z01-J001-B1-42 (quasi-targeted metabolomics)/4.MetDiffAnalysis/High_GAA.vs.Control/ROC_all/Com_277_neg_ROC.pdf]

High\_GAA.vs.Control

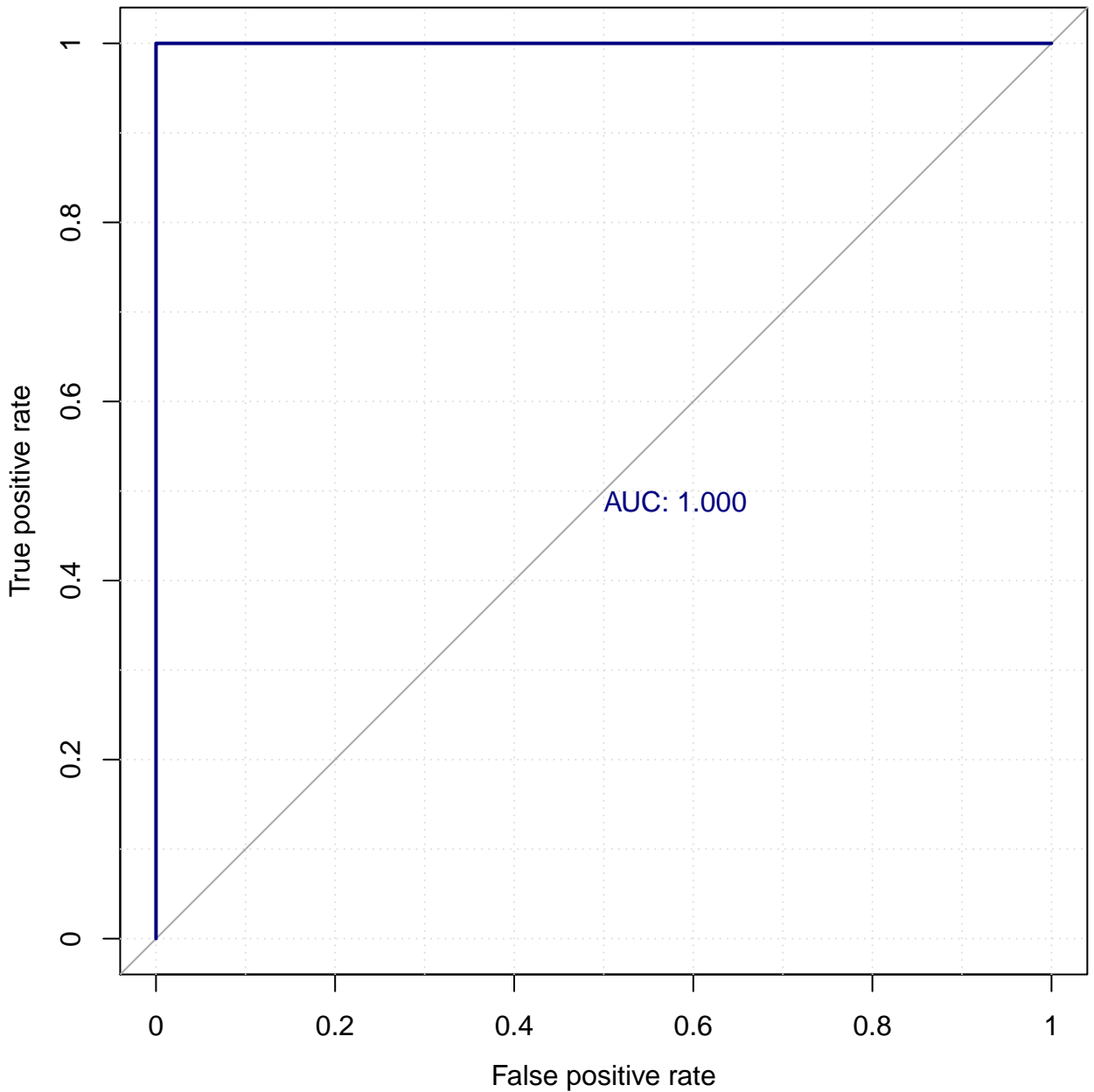

Supplement: Supplementary file 2 [file Data_Sheet_1.ZIP › Result-X101SC22030966-Z01-J001-B1-42 (quasi-targeted metabolomics)/4.MetDiffAnalysis/High_GAA.vs.Control/ROC_all/Com_30_neg_ROC.pdf]

High\_GAA.vs.Control

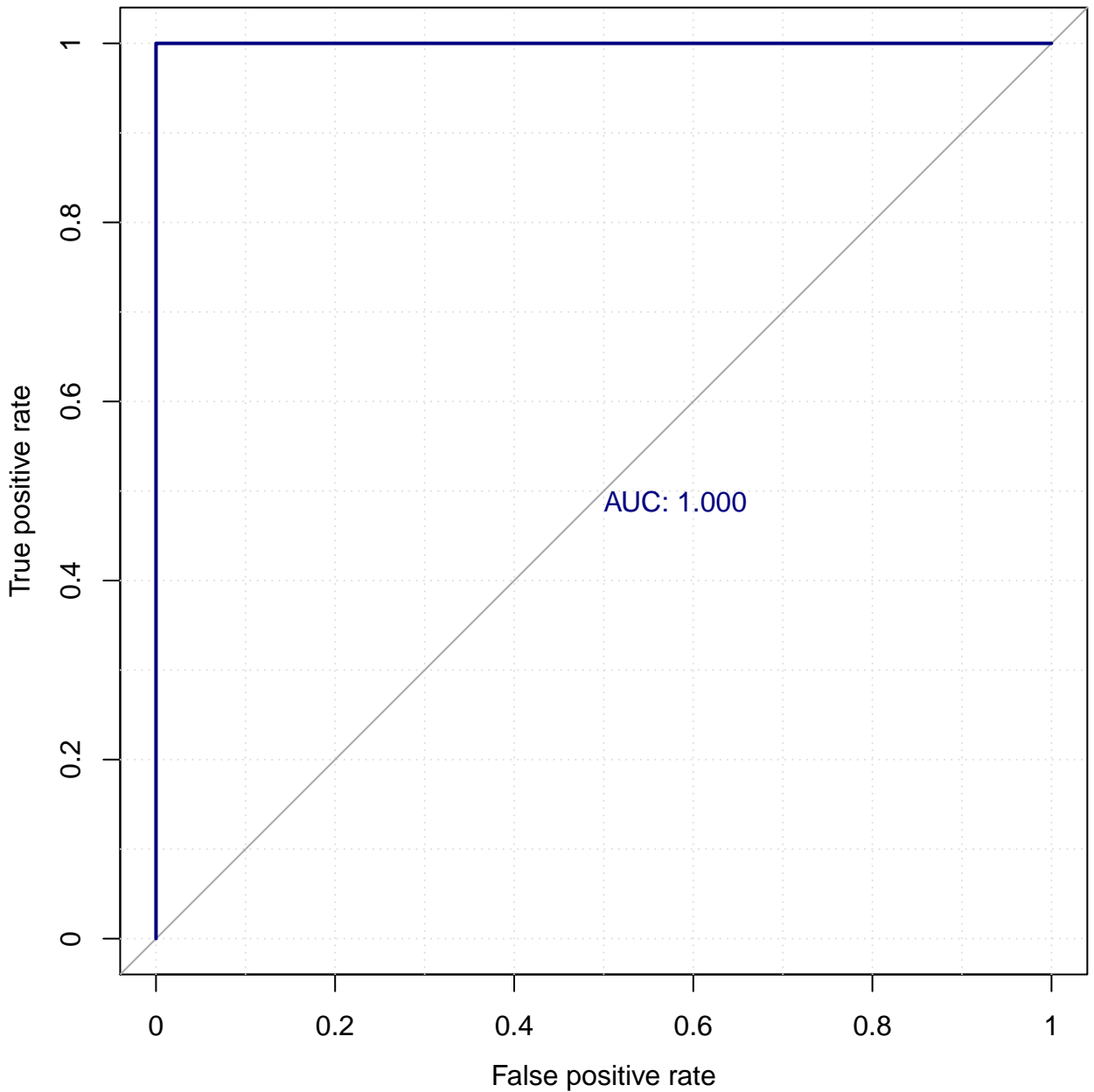

Supplement: Supplementary file 2 [file Data_Sheet_1.ZIP › Result-X101SC22030966-Z01-J001-B1-42 (quasi-targeted metabolomics)/4.MetDiffAnalysis/High_GAA.vs.Control/ROC_all/Com_371_pos_ROC.pdf]

High\_GAA.vs.Control

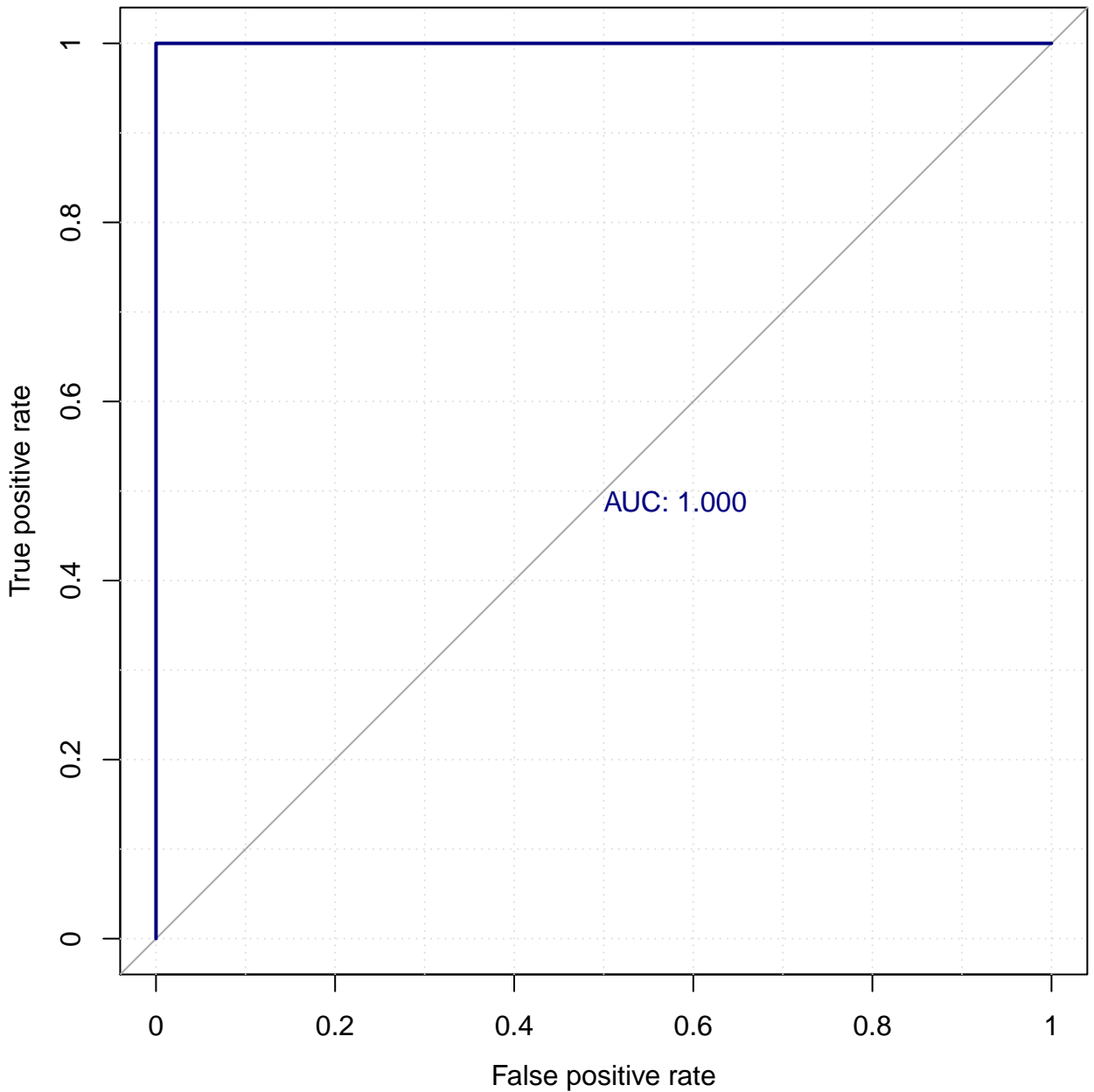

Supplement: Supplementary file 2 [file Data_Sheet_1.ZIP › Result-X101SC22030966-Z01-J001-B1-42 (quasi-targeted metabolomics)/4.MetDiffAnalysis/High_GAA.vs.Control/ROC_all/Com_409_pos_ROC.pdf]

High\_GAA.vs.Control

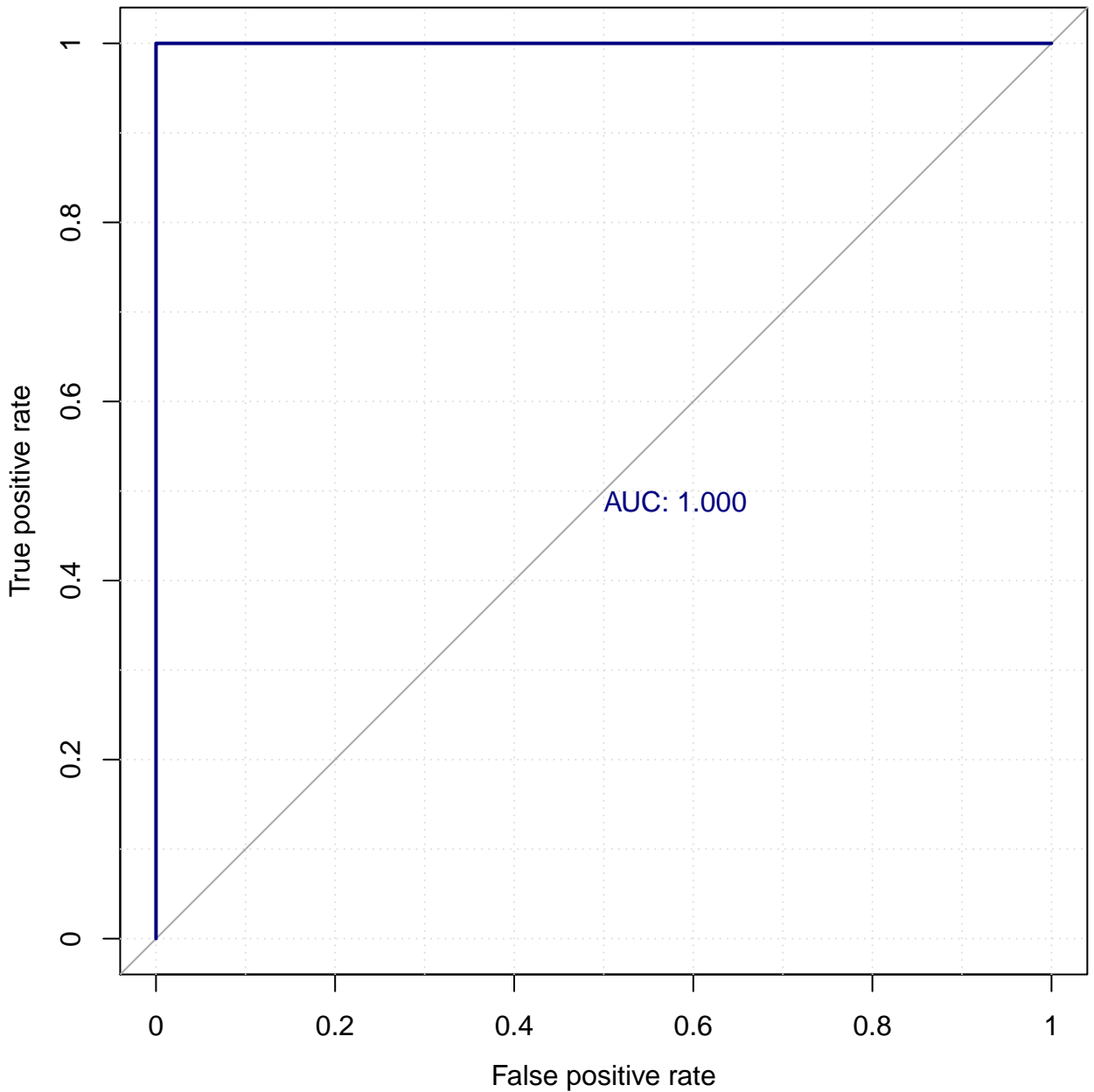

Supplement: Supplementary file 2 [file Data_Sheet_1.ZIP › Result-X101SC22030966-Z01-J001-B1-42 (quasi-targeted metabolomics)/4.MetDiffAnalysis/High_GAA.vs.Control/ROC_all/Com_459_pos_ROC.pdf]

High\_GAA.vs.Control

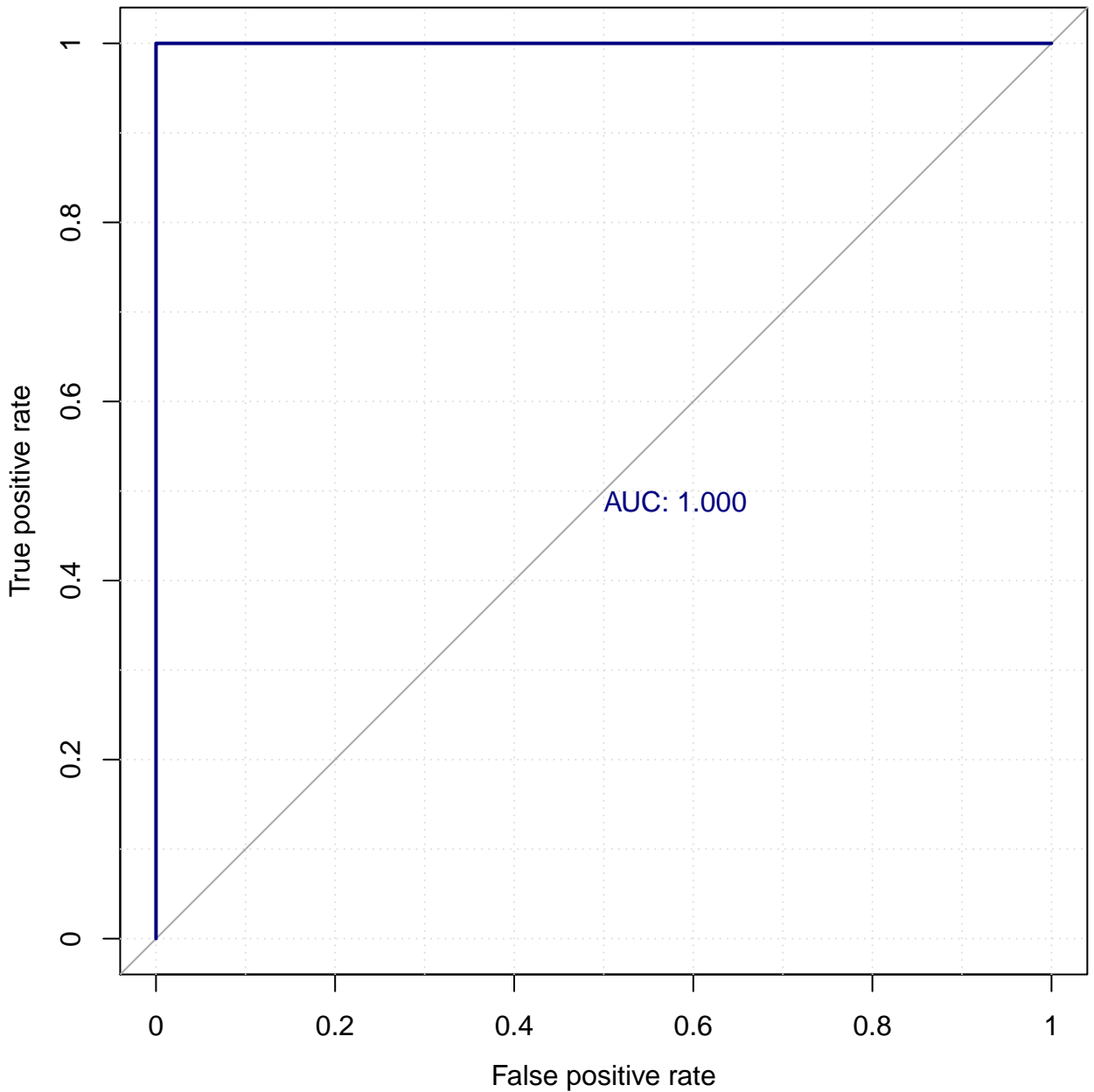

Supplement: Supplementary file 2 [file Data_Sheet_1.ZIP › Result-X101SC22030966-Z01-J001-B1-42 (quasi-targeted metabolomics)/4.MetDiffAnalysis/High_GAA.vs.Control/ROC_all/Com_587_pos_ROC.pdf]

High\_GAA.vs.Control

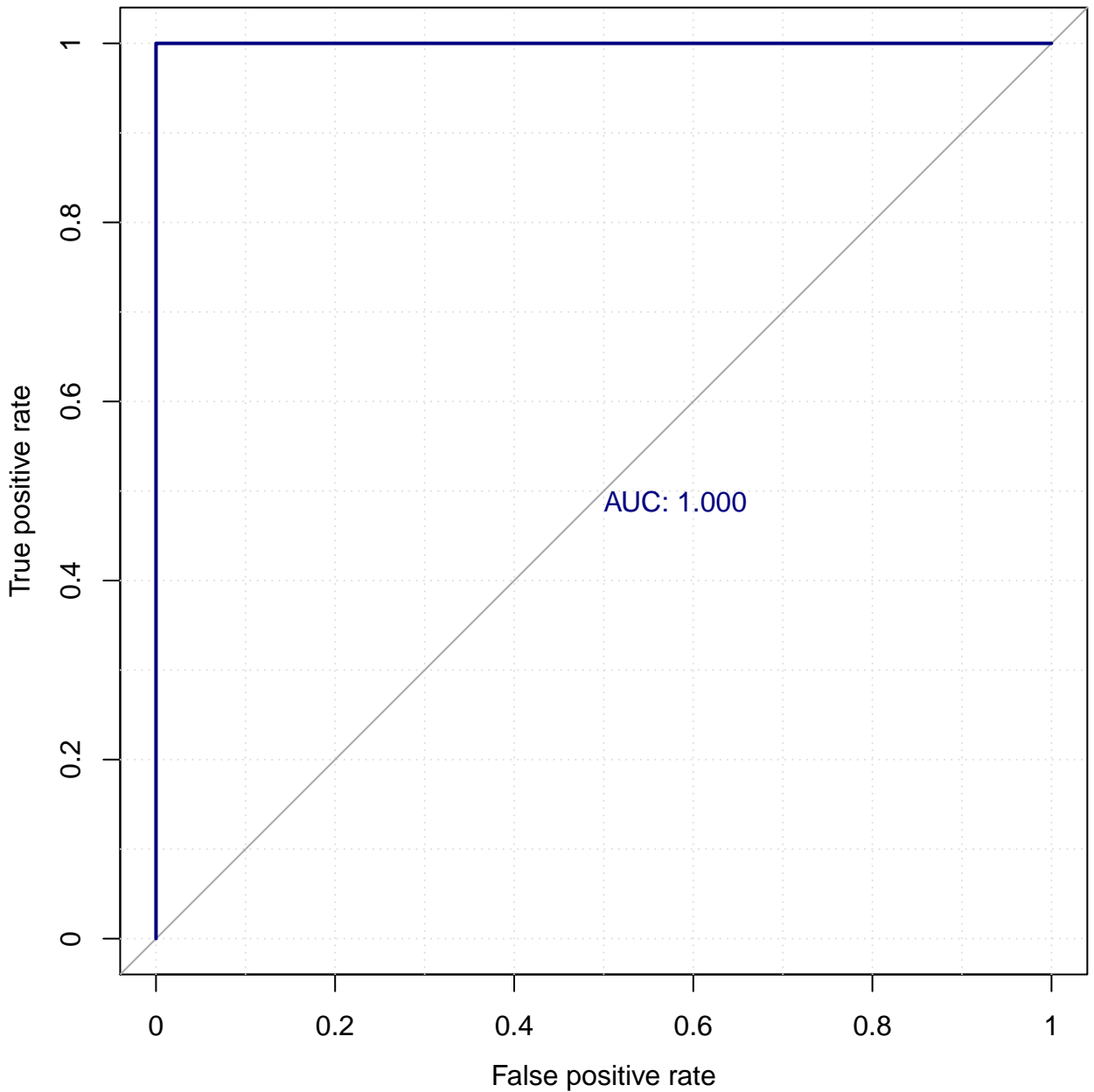

Supplement: Supplementary file 2 [file Data_Sheet_1.ZIP › Result-X101SC22030966-Z01-J001-B1-42 (quasi-targeted metabolomics)/4.MetDiffAnalysis/High_GAA.vs.Control/ROC_all/Com_60_neg_ROC.pdf]

High\_GAA.vs.Control

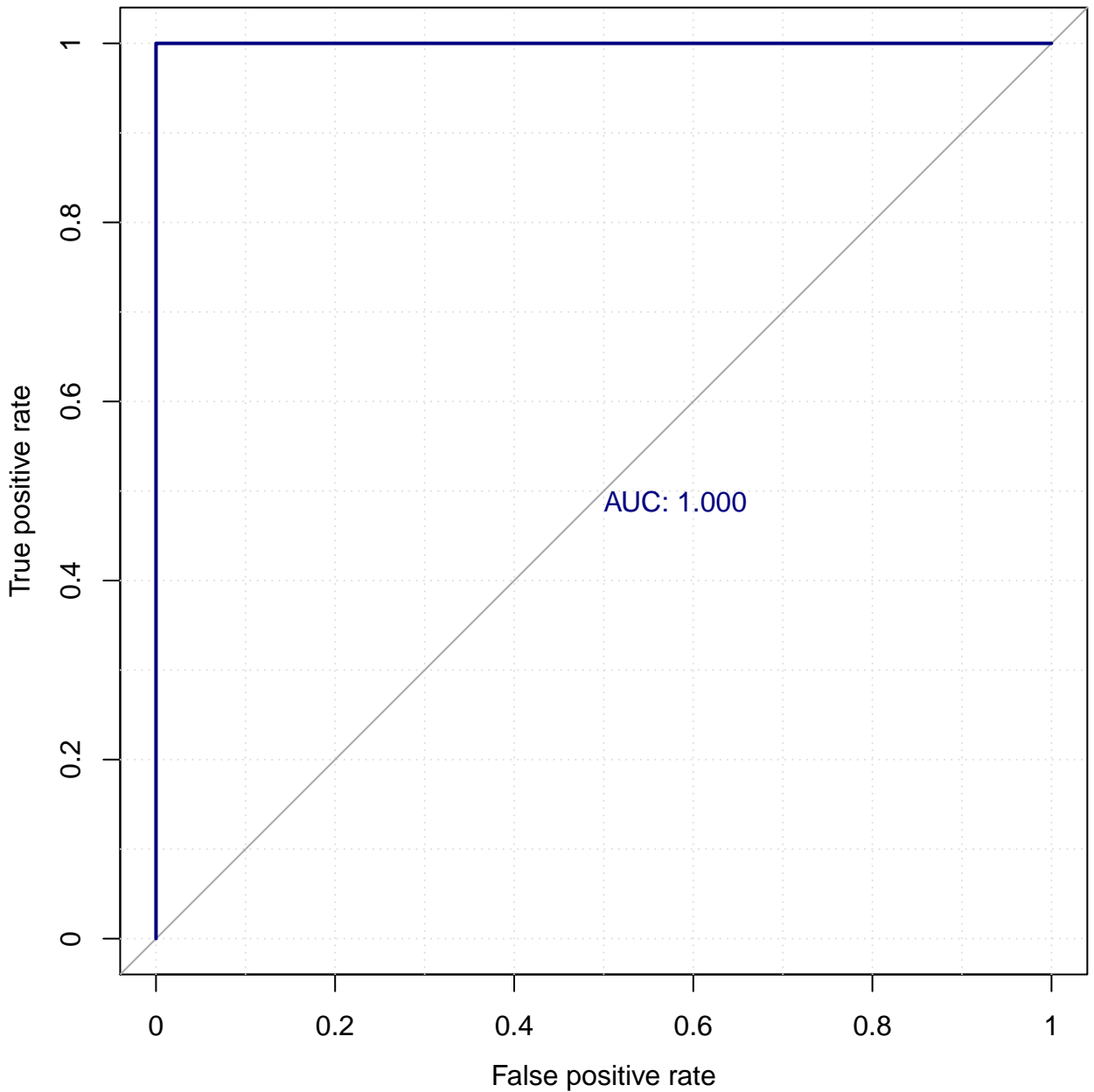

Supplement: Supplementary file 2 [file Data_Sheet_1.ZIP › Result-X101SC22030966-Z01-J001-B1-42 (quasi-targeted metabolomics)/4.MetDiffAnalysis/High_GAA.vs.Control/ROC_all/Com_651_pos_ROC.pdf]

High\_GAA.vs.Control

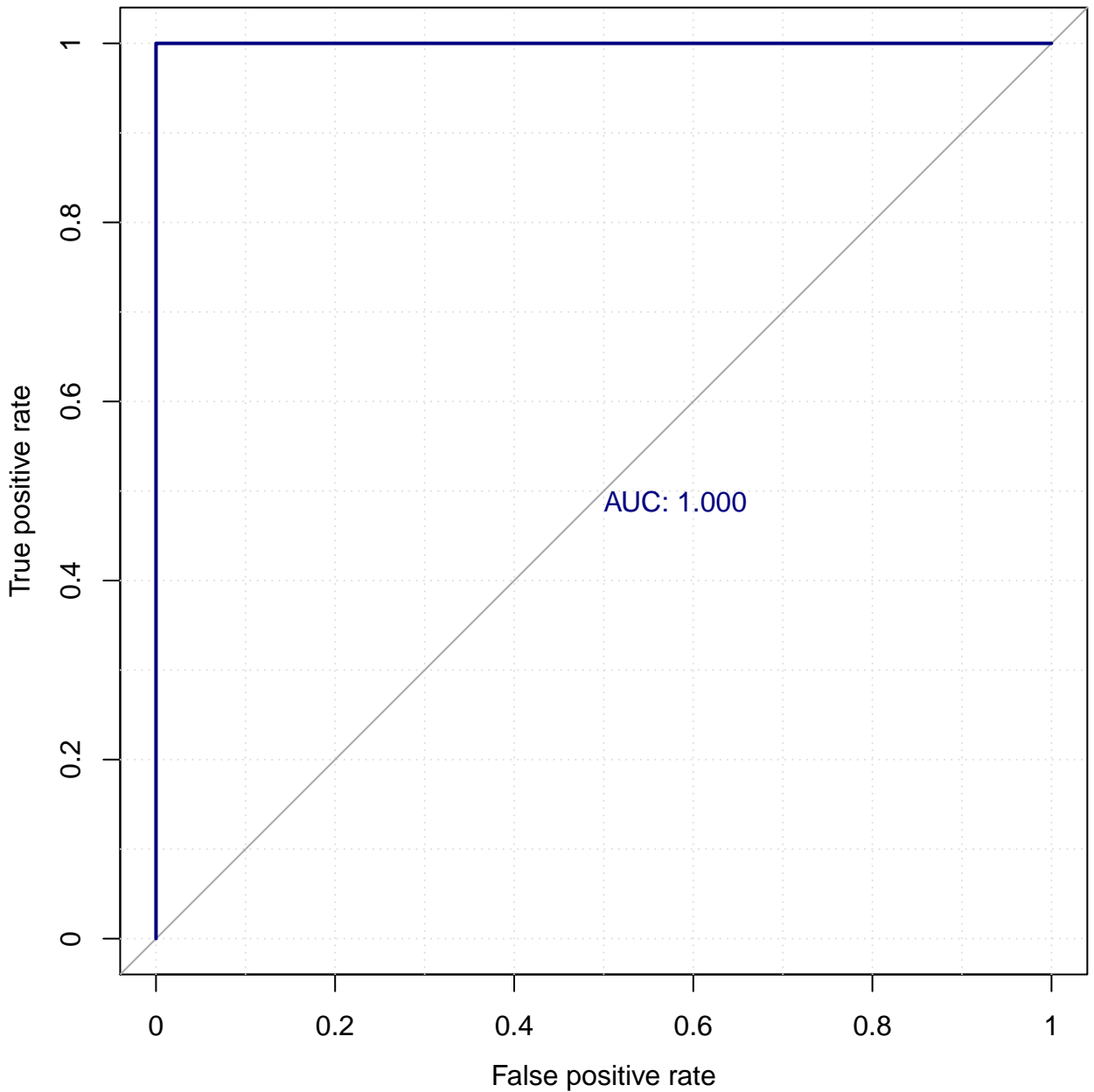

Supplement: Supplementary file 2 [file Data_Sheet_1.ZIP › Result-X101SC22030966-Z01-J001-B1-42 (quasi-targeted metabolomics)/4.MetDiffAnalysis/High_GAA.vs.Control/ROC_all/Com_83_neg_ROC.pdf]

High\_GAA.vs.No\_GAA

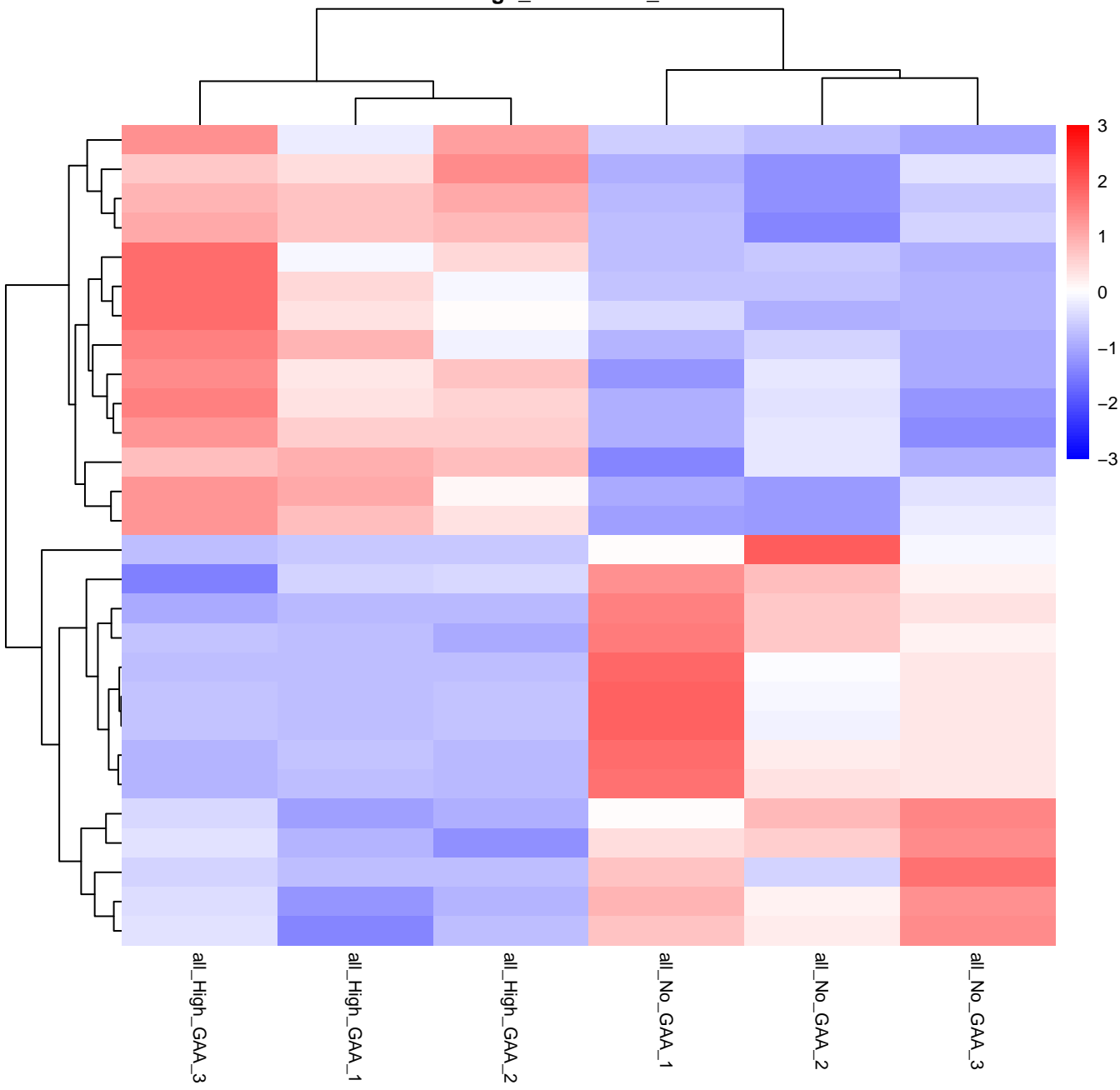

Supplement: Supplementary file 2 [file Data_Sheet_1.ZIP › Result-X101SC22030966-Z01-J001-B1-42 (quasi-targeted metabolomics)/4.MetDiffAnalysis/High_GAA.vs.No_GAA/High_GAA.vs.No_GAA_all_cluster_heatmap.pdf]

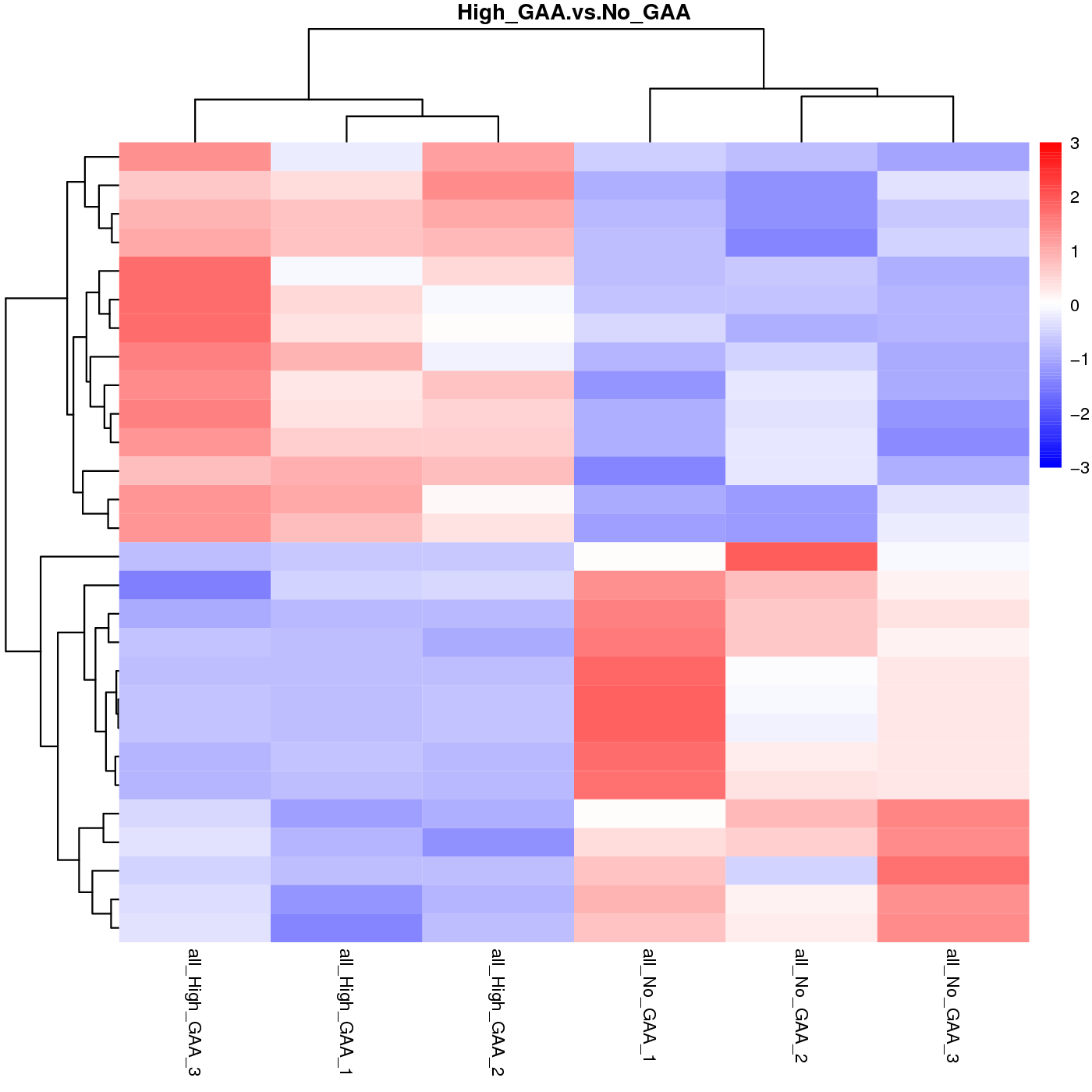

Supplement: Supplementary file 2 [file Data_Sheet_1.ZIP › Result-X101SC22030966-Z01-J001-B1-42 (quasi-targeted metabolomics)/4.MetDiffAnalysis/High_GAA.vs.No_GAA/High_GAA.vs.No_GAA_all_cluster_heatmap.png]
