# Supplementary material for: Association analysis of transcriptome and quasi-targeted metabolomics reveals the regulation mechanism underlying broiler muscle tissue development at different levels of dietary guanidinoacetic acid
Source: Front Vet Sci. 2024 Apr 25;11:1384028. doi: 10.3389/fvets.2024.1384028 (PMC11080945; doi:10.3389/fvets.2024.1384028)
Supplement: Supplementary file 2 [file Data_Sheet_1.ZIP › Result-X101SC22030966-Z01-J001-B1-42 (quasi-targeted metabolomics)/4.MetDiffAnalysis/No_GAA.vs.Control/No_GAA.vs.Control_all_heatmap_detail.pdf]

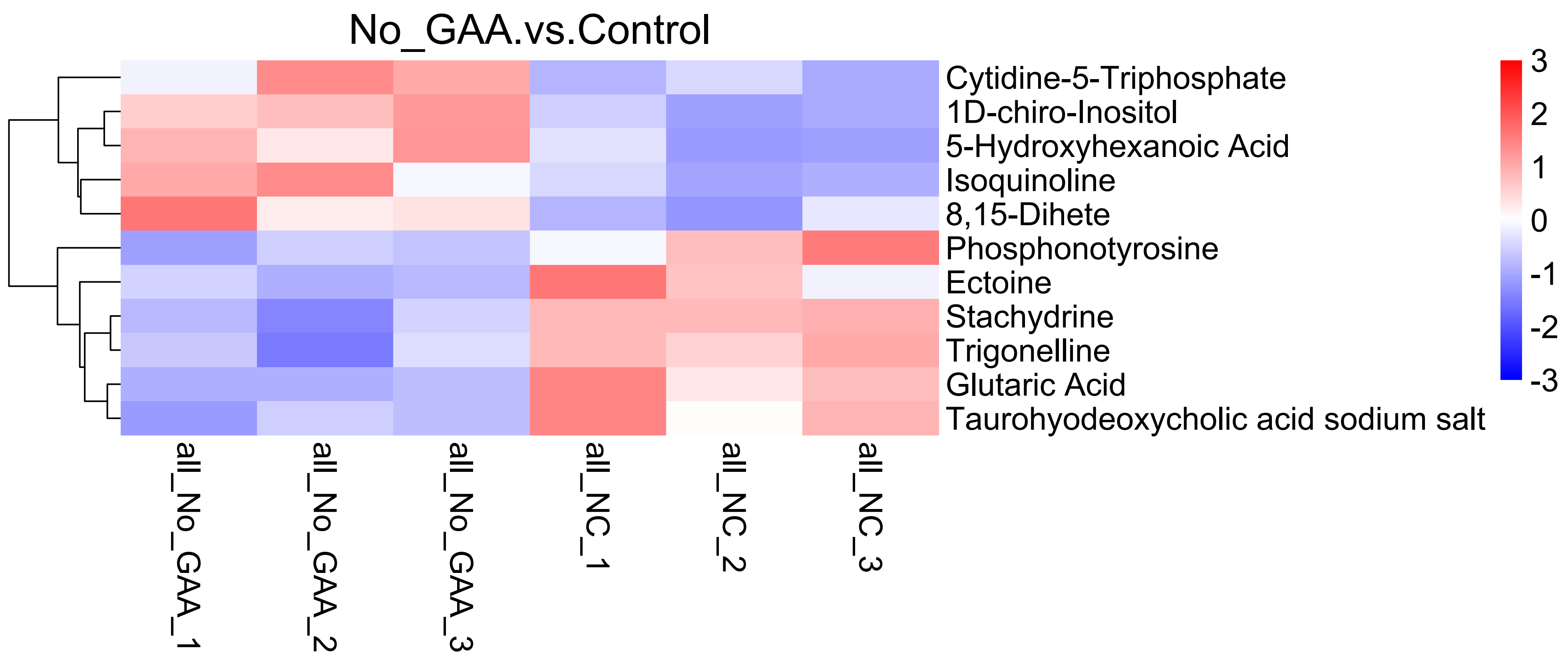
No_GAA.vs.Control
Cytidine-5-Triphosphate
1D-chiro-Inositol
5-Hydroxyhexanoic Acid
Isoquinoline
8,15-Dihete
Phosphonotyrosine
Ectoine
Stachydrine
Trigonelline
Glutaric Acid
Taurohyodeoxycholic acid sodium salt
all_No_GAA_1
all_No_GAA_2
all_No_GAA_3
all_NC_1
all_NC_2
all_NC_3
3
2
1
0
-1
-2
-3
