# Supplementary material for: Association analysis of transcriptome and quasi-targeted metabolomics reveals the regulation mechanism underlying broiler muscle tissue development at different levels of dietary guanidinoacetic acid
Source: Front Vet Sci. 2024 Apr 25;11:1384028. doi: 10.3389/fvets.2024.1384028 (PMC11080945; doi:10.3389/fvets.2024.1384028)
Supplement: Supplementary file 2 [file Data_Sheet_1.ZIP › Result-X101SC22030966-Z01-J001-B1-42 (quasi-targeted metabolomics)/4.MetDiffAnalysis/No_GAA.vs.Control/No_GAA.vs.Control_all_zscore.pdf]

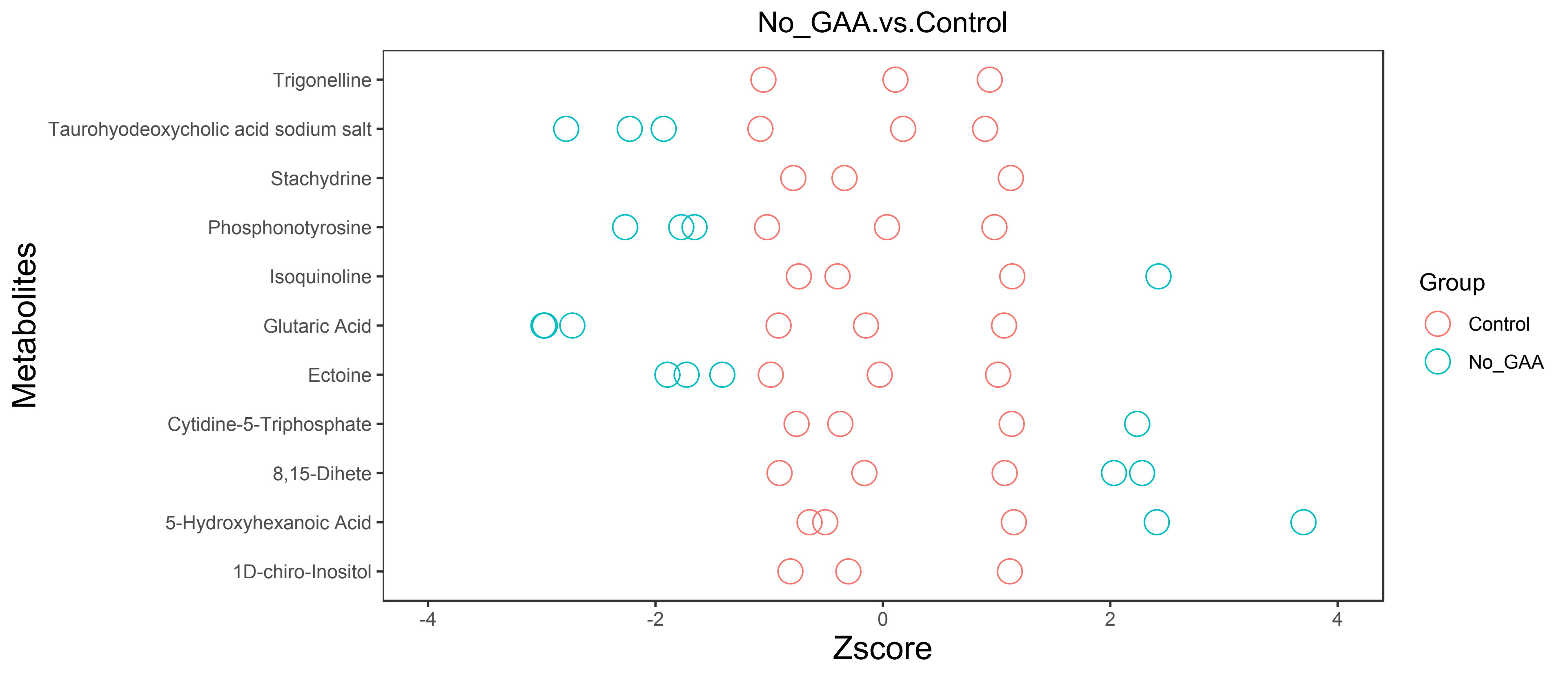
No_GAA.vs.Control
Metabolites
Trigonelline
Taurohyodeoxycholic acid sodium salt
Stachydrine
Phosphonotyrosine
Isoquinoline
Glutaric Acid
Ectoine
Cytidine-5-Triphosphate
8,15-Dihete
5-Hydroxyhexanoic Acid
1D-chiro-Inositol
-4
-2
0
2
4
Zscore
Group
Control
No_GAA
