# Supplementary material for: Association analysis of transcriptome and quasi-targeted metabolomics reveals the regulation mechanism underlying broiler muscle tissue development at different levels of dietary guanidinoacetic acid
Source: Front Vet Sci. 2024 Apr 25;11:1384028. doi: 10.3389/fvets.2024.1384028 (PMC11080945; doi:10.3389/fvets.2024.1384028)

# MetKEGGEnrichment Readme

**|-- 5.MetKEGGEnrichment　　　　【差异代谢物 KEGG 富集分析结果目录】**
| -- *.vs.*　　　　　　　　　　　　　　　　　【比较的样品对目录】
| |-- *.vs.*_all.KEGG_enrichment.xls　　　　　　【差异代谢物 KEGG 富集结果列表】
| |-- *.vs.*_all.KEGG_Enrich.scatterplot.{png,pdf} 【KEGG 富集气泡图】
| |-- *.vs.*_all_KEGG_map　　　　　　　　　　【KEGG 通路图】

***.vs.*_all.KEGG_enrichment.xls**

第一列：MapID，富集的 KEGG PATHWAY 的 ID；
第二列：MapTitle，富集的 KEGG PATHWAY 名称；
第三列：Pvalue，富集分析的 Pvalue；
第四列：x，与该通路相关的差异代谢物的数目；
第五列：y，与该通路相关的背景（所有）代谢物的数目；
第六列：n，KEGG 注释的差异代谢物数目；
第七列：N，KEGG 注释的背景（所有）代谢物的数目；
第八列：EnrichDirect，富集指向,Over 代表富集；
第九列：MetaIDs，富集到的代谢物 list；
第十列：KEGG_cpd_id，KEGG 数据库代谢物的 ID；

***.vs.*_all.KEGG_Enrich.scatterplot.{png,pdf}**

KEGG 富集气泡图：根据 KEGG 富集结果，选取 P-value 值由小到大排序 Top20 的通路绘制气泡图。横坐标为 x/y（相应代谢通路中差异代谢物的数目/该通路中鉴定出总代谢物数目），值越大，表示该通路中差异代谢物富集程度越高，纵坐标为 KEGG 通路名称。点的颜色代表超几何检验的 P-value 值，值越小，说明检验的可靠性越大、越具统计学意义。点的大小代表相应通路中差异代谢物的数目，越大，该通路内差异代谢物就越多。

***.vs.*_all_KEGG_map**

**KEGG 通路图解释说明：**

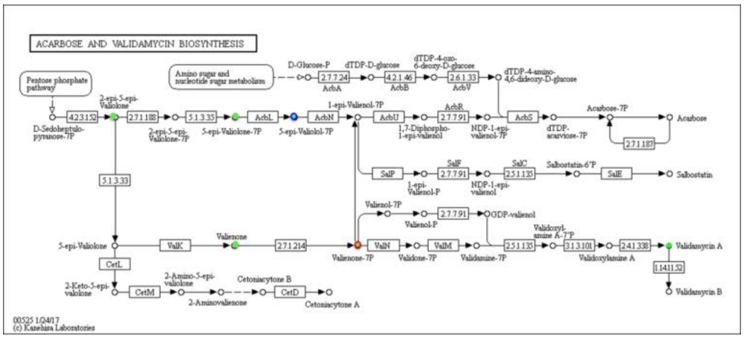


**KEGG 富集通路图**

KEGG PATHWAY Database（https://www.genome.jp/KEGG/pathway.html）是根据相关知识手绘的代谢通路数据库，目前分为以下几类：1.新陈代谢、2.遗传信息加工、3.环境信息加工、4.细胞过程、5.生物体系统、6.人类疾病、7.药物开发

**说明：**

绿色：注释到的代谢物
蓝色框：下调差异代谢物

红色框：上调差异代谢物

黄色圆圈：同时包含上下调的代谢物

下方为图例解释，仅供参考；其他说明详见官网解释：

https://www.genome.jp/KEGG/document/help_pathway.html

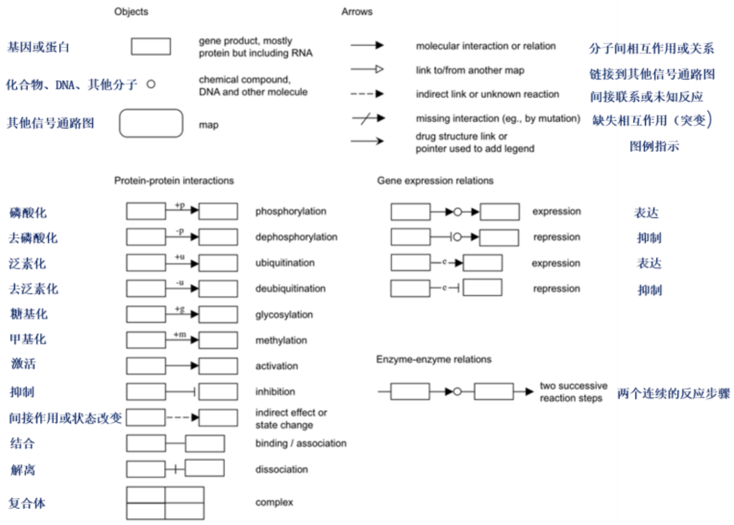

Supplement: Supplementary file 2 [file Data_Sheet_1.ZIP › Result-X101SC22030966-Z01-J001-B1-42 (quasi-targeted metabolomics)/5.MetKeggEnrichment/5-MetKeggEnrichment-readme.pdf]
