# Supplementary material for: Association analysis of transcriptome and quasi-targeted metabolomics reveals the regulation mechanism underlying broiler muscle tissue development at different levels of dietary guanidinoacetic acid
Source: Front Vet Sci. 2024 Apr 25;11:1384028. doi: 10.3389/fvets.2024.1384028 (PMC11080945; doi:10.3389/fvets.2024.1384028)
Supplement: Supplementary file 2 [file Data_Sheet_1.ZIP › Result-X101SC22030966-Z01-J001-B1-42 (quasi-targeted metabolomics)/5.MetKeggEnrichment/High_GAA.vs.Control/High_GAA.vs.Control_all.KEGG_Enrich.scatterplot.pdf]

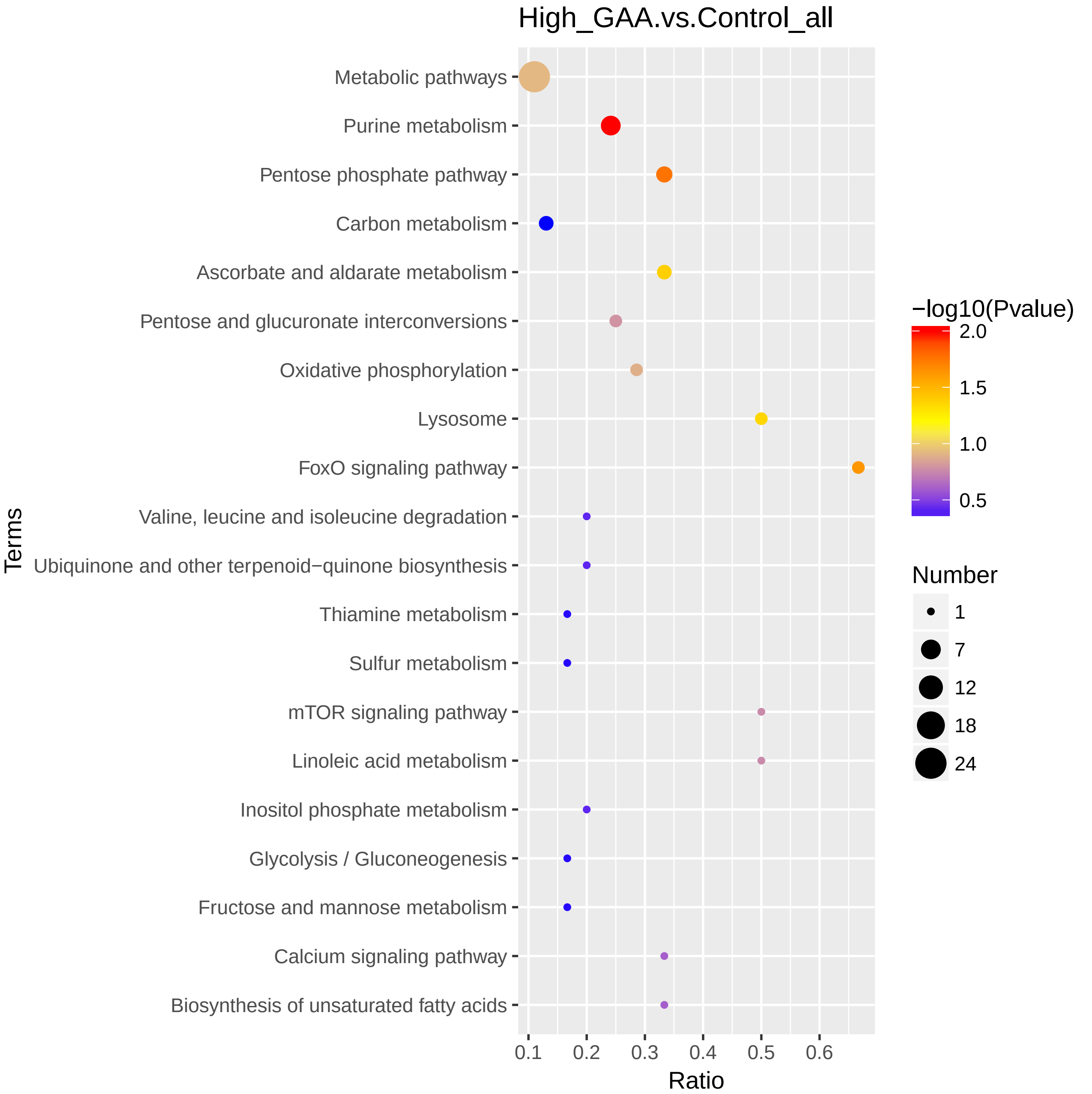
High_GAA.vs.Control_all
Metabolic pathways
Purine metabolism
Pentose phosphate pathway
Carbon metabolism
Ascorbate and aldarate metabolism
Pentose and glucuronate interconversions
Oxidative phosphorylation
Lysosome
FoxO signaling pathway
Valine, leucine and isoleucine degradation
Ubiquinone and other terpenoid−quinone biosynthesis
Thiamine metabolism
Sulfur metabolism
mTOR signaling pathway
Linoleic acid metabolism
Inositol phosphate metabolism
Glycolysis / Gluconeogenesis
Fructose and mannose metabolism
Calcium signaling pathway
Biosynthesis of unsaturated fatty acids
Terms
0.1
0.2
0.3
0.4
0.5
0.6
Ratio
−log10(Pvalue)
2.0
1.5
1.0
0.5
Number
1
7
12
18
24
