# Supplementary material for: Association analysis of transcriptome and quasi-targeted metabolomics reveals the regulation mechanism underlying broiler muscle tissue development at different levels of dietary guanidinoacetic acid
Source: Front Vet Sci. 2024 Apr 25;11:1384028. doi: 10.3389/fvets.2024.1384028 (PMC11080945; doi:10.3389/fvets.2024.1384028)
Supplement: Supplementary file 2 [file Data_Sheet_1.ZIP › Result-X101SC22030966-Z01-J001-B1-42 (quasi-targeted metabolomics)/5.MetKeggEnrichment/High_GAA.vs.Control/High_GAA.vs.Control_all_KEGG_map/KEGG.map.html]

Pathway Enrichment


  

# The most enriched pathway terms of High\_GAA.vs.Control\_all

Statistic method: hypergeometric test

FDR correction method: Benjamini and Hochberg

| MapID | MapTitle | Pvalue | AdjustedPv | x | y | n | N | EnrichDirect | GeneIDs |
| --- | --- | --- | --- | --- | --- | --- | --- | --- | --- |
| map00230 | Purine metabolism | 0.00999037851977188 | 0.295896254668881 | 7 | 29 | 27 | 292 | Over | Com\_10\_neg Com\_133\_neg Com\_158\_neg Com\_161\_neg Com\_226\_neg Com\_378\_pos Com\_562\_pos |
| map00030 | Pentose phosphate pathway | 0.0172682867929701 | 0.295896254668881 | 4 | 12 | 27 | 292 | Over | Com\_158\_neg Com\_159\_neg Com\_163\_neg Com\_84\_neg |
| map04068 | FoxO signaling pathway | 0.0233602306317538 | 0.295896254668881 | 2 | 3 | 27 | 292 | Over | Com\_10\_neg Com\_226\_neg |
| map00053 | Ascorbate and aldarate metabolism | 0.040748976967406 | 0.334773061932659 | 3 | 9 | 27 | 292 | Over | Com\_164\_neg Com\_244\_neg Com\_84\_neg |
| map04142 | Lysosome | 0.0440490870964025 | 0.334773061932659 | 2 | 4 | 27 | 292 | Over | Com\_144\_neg Com\_226\_neg |
| map01100 | Metabolic pathways | 0.120576216490046 | 0.671345855105211 | 24 | 218 | 27 | 292 | Over | Com\_10\_neg Com\_133\_neg Com\_144\_neg Com\_148\_neg Com\_159\_neg Com\_15\_neg Com\_161\_neg Com\_163\_neg Com\_164\_neg Com\_220\_neg Com\_226\_neg Com\_242\_neg Com\_244\_neg Com\_254\_neg Com\_267\_neg Com\_319\_pos Com\_378\_pos Com\_434\_pos Com\_489\_pos Com\_562\_pos Com\_650\_pos Com\_654\_pos Com\_84\_neg Com\_95\_neg |
| map00190 | Oxidative phosphorylation | 0.12946424926691 | 0.671345855105211 | 2 | 7 | 27 | 292 | Over | Com\_226\_neg Com\_267\_neg |
| map00040 | Pentose and glucuronate interconversions | 0.162968421252362 | 0.671345855105211 | 2 | 8 | 27 | 292 | Over | Com\_244\_neg Com\_84\_neg |
| map00591 | Linoleic acid metabolism | 0.176669961869792 | 0.671345855105211 | 1 | 2 | 27 | 292 | Over | Com\_220\_neg |
| map04150 | mTOR signaling pathway | 0.176669961869792 | 0.671345855105211 | 1 | 2 | 27 | 292 | Over | Com\_10\_neg |
| map01040 | Biosynthesis of unsaturated fatty acids | 0.253324827488812 | 0.802195287047904 | 1 | 3 | 27 | 292 | Over | Com\_220\_neg |
| map04020 | Calcium signaling pathway | 0.253324827488812 | 0.802195287047904 | 1 | 3 | 27 | 292 | Over | Com\_267\_neg |
| map00130 | Ubiquinone and other terpenoid-quinone biosynthesis | 0.386544338155276 | 0.868590136700365 | 1 | 5 | 27 | 292 | Over | Com\_95\_neg |
| map00280 | Valine, leucine and isoleucine degradation | 0.386544338155276 | 0.868590136700365 | 1 | 5 | 27 | 292 | Over | Com\_148\_neg |
| map00562 | Inositol phosphate metabolism | 0.386544338155276 | 0.868590136700365 | 1 | 5 | 27 | 292 | Over | Com\_371\_pos |
| map00010 | Glycolysis / Gluconeogenesis | 0.444256194844501 | 0.868590136700365 | 1 | 6 | 27 | 292 | Over | Com\_148\_neg |
| map00051 | Fructose and mannose metabolism | 0.444256194844501 | 0.868590136700365 | 1 | 6 | 27 | 292 | Over | Com\_144\_neg |
| map00730 | Thiamine metabolism | 0.444256194844501 | 0.868590136700365 | 1 | 6 | 27 | 292 | Over | Com\_148\_neg |
| map00920 | Sulfur metabolism | 0.444256194844501 | 0.868590136700365 | 1 | 6 | 27 | 292 | Over | Com\_133\_neg |
| map01200 | Carbon metabolism | 0.457152703526508 | 0.868590136700365 | 3 | 23 | 27 | 292 | Over | Com\_159\_neg Com\_163\_neg Com\_84\_neg |
| map01230 | Biosynthesis of amino acids | 0.534841192740031 | 0.893326141707745 | 4 | 34 | 27 | 292 | Over | Com\_159\_neg Com\_15\_neg Com\_84\_neg Com\_95\_neg |
| map00640 | Propanoate metabolism | 0.544400533086728 | 0.893326141707745 | 1 | 8 | 27 | 292 | Over | Com\_148\_neg |
| map00020 | Citrate cycle (TCA cycle) | 0.58771456691299 | 0.893326141707745 | 1 | 9 | 27 | 292 | Over | Com\_148\_neg |
| map00400 | Phenylalanine, tyrosine and tryptophan biosynthesis | 0.58771456691299 | 0.893326141707745 | 1 | 9 | 27 | 292 | Over | Com\_95\_neg |
| map00620 | Pyruvate metabolism | 0.58771456691299 | 0.893326141707745 | 1 | 9 | 27 | 292 | Over | Com\_148\_neg |
| map00520 | Amino sugar and nucleotide sugar metabolism | 0.637557561393294 | 0.931814897420968 | 2 | 15 | 27 | 292 | Over | Com\_144\_neg Com\_244\_neg |
