# Supplementary material for: Association analysis of transcriptome and quasi-targeted metabolomics reveals the regulation mechanism underlying broiler muscle tissue development at different levels of dietary guanidinoacetic acid
Source: Front Vet Sci. 2024 Apr 25;11:1384028. doi: 10.3389/fvets.2024.1384028 (PMC11080945; doi:10.3389/fvets.2024.1384028)
Supplement: Supplementary file 2 [file Data_Sheet_1.ZIP › Result-X101SC22030966-Z01-J001-B1-42 (quasi-targeted metabolomics)/5.MetKeggEnrichment/High_GAA.vs.Control/High_GAA.vs.Control_all_KEGG_map/src/04621.html]

map04621


Close
