# Supplementary material for: Association analysis of transcriptome and quasi-targeted metabolomics reveals the regulation mechanism underlying broiler muscle tissue development at different levels of dietary guanidinoacetic acid
Source: Front Vet Sci. 2024 Apr 25;11:1384028. doi: 10.3389/fvets.2024.1384028 (PMC11080945; doi:10.3389/fvets.2024.1384028)
Supplement: Supplementary file 2 [file Data_Sheet_1.ZIP › Result-X101SC22030966-Z01-J001-B1-42 (quasi-targeted metabolomics)/5.MetKeggEnrichment/High_GAA.vs.No_GAA/High_GAA.vs.No_GAA_all.KEGG_Enrich.scatterplot.pdf]

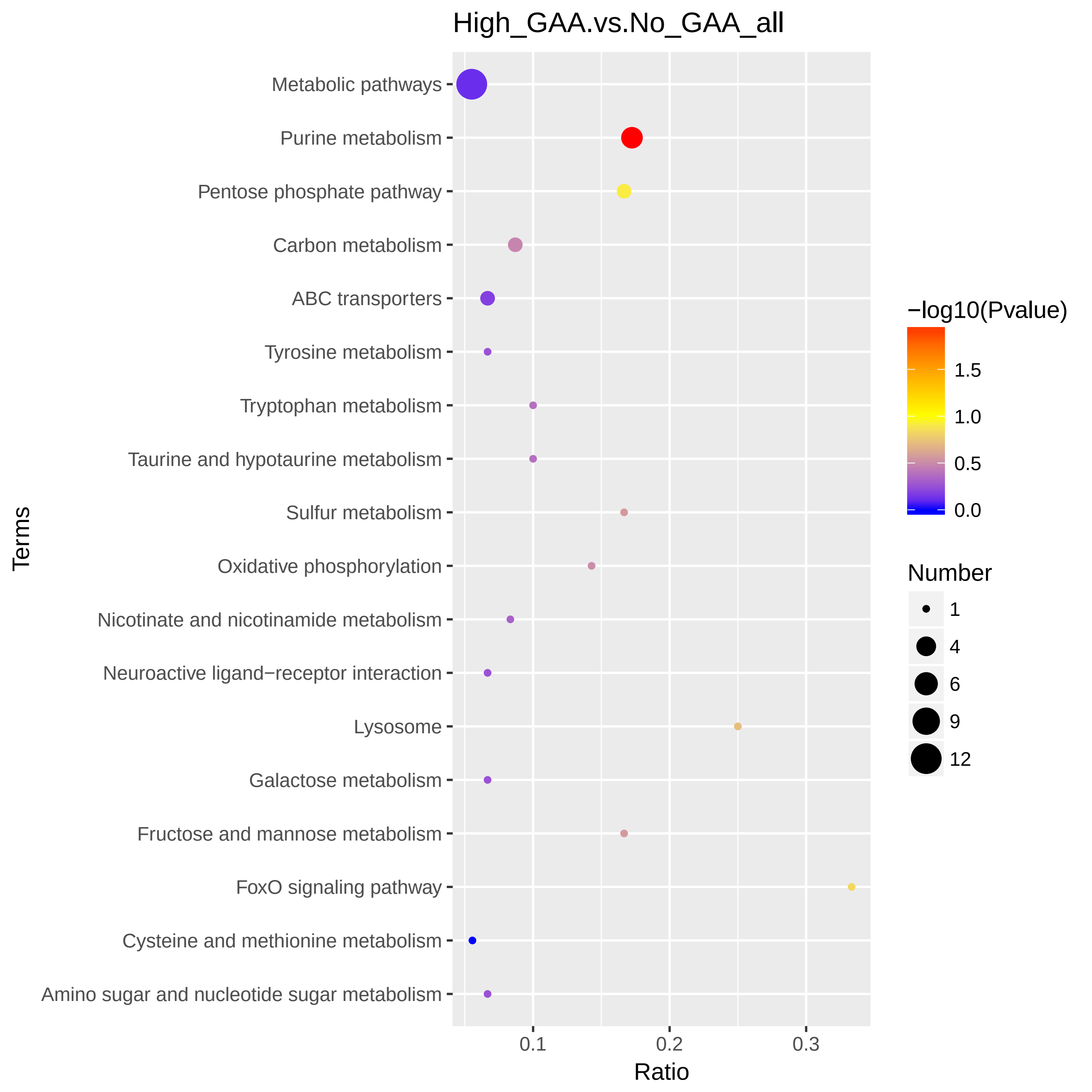

High_GAA.vs.No_GAA_all
Metabolic pathways
Purine metabolism
Pentose phosphate pathway
Carbon metabolism
ABC transporters
Tyrosine metabolism
Tryptophan metabolism
Taurine and hypotaurine metabolism
Sulfur metabolism
Oxidative phosphorylation
Nicotinate and nicotinamide metabolism
Neuroactive ligand−receptor interaction
Lysosome
Galactose metabolism
Fructose and mannose metabolism
FoxO signaling pathway
Cysteine and methionine metabolism
Amino sugar and nucleotide sugar metabolism
Terms
0.1
0.2
0.3
Ratio
−log10(Pvalue)
1.5
1.0
0.5
0.0
Number
1
4
6
9
12
