# Supplementary material for: Association analysis of transcriptome and quasi-targeted metabolomics reveals the regulation mechanism underlying broiler muscle tissue development at different levels of dietary guanidinoacetic acid
Source: Front Vet Sci. 2024 Apr 25;11:1384028. doi: 10.3389/fvets.2024.1384028 (PMC11080945; doi:10.3389/fvets.2024.1384028)
Supplement: Supplementary file 2 [file Data_Sheet_1.ZIP › Result-X101SC22030966-Z01-J001-B1-42 (quasi-targeted metabolomics)/5.MetKeggEnrichment/High_GAA.vs.No_GAA/High_GAA.vs.No_GAA_all_KEGG_map/KEGG.map.html]

Pathway Enrichment


  

# The most enriched pathway terms of High\_GAA.vs.No\_GAA\_all

Statistic method: hypergeometric test

FDR correction method: Benjamini and Hochberg

| MapID | MapTitle | Pvalue | AdjustedPv | x | y | n | N | EnrichDirect | GeneIDs |
| --- | --- | --- | --- | --- | --- | --- | --- | --- | --- |
| map00230 | Purine metabolism | 0.0101095502086792 | 0.202191004173583 | 5 | 29 | 15 | 292 | Over | Com\_133\_neg Com\_161\_neg Com\_226\_neg Com\_293\_neg Com\_378\_pos |
| map00030 | Pentose phosphate pathway | 0.12066826825563 | 0.740903693226869 | 2 | 12 | 15 | 292 | Over | Com\_159\_neg Com\_92\_neg |
| map04068 | FoxO signaling pathway | 0.146806169009832 | 0.740903693226869 | 1 | 3 | 15 | 292 | Over | Com\_226\_neg |
| map04142 | Lysosome | 0.191089585843232 | 0.740903693226869 | 1 | 4 | 15 | 292 | Over | Com\_226\_neg |
| map00051 | Fructose and mannose metabolism | 0.273295928745342 | 0.740903693226869 | 1 | 6 | 15 | 292 | Over | Com\_275\_neg |
| map00920 | Sulfur metabolism | 0.273295928745342 | 0.740903693226869 | 1 | 6 | 15 | 292 | Over | Com\_133\_neg |
| map00190 | Oxidative phosphorylation | 0.311409778636321 | 0.740903693226869 | 1 | 7 | 15 | 292 | Over | Com\_226\_neg |
| map01200 | Carbon metabolism | 0.334236036459606 | 0.740903693226869 | 2 | 23 | 15 | 292 | Over | Com\_159\_neg Com\_92\_neg |
| map00380 | Tryptophan metabolism | 0.414856946583972 | 0.740903693226869 | 1 | 10 | 15 | 292 | Over | Com\_392\_pos |
| map00430 | Taurine and hypotaurine metabolism | 0.414856946583972 | 0.740903693226869 | 1 | 10 | 15 | 292 | Over | Com\_197\_neg |
| map00760 | Nicotinate and nicotinamide metabolism | 0.475555514251115 | 0.740903693226869 | 1 | 12 | 15 | 292 | Over | Com\_537\_pos |
| map00052 | Galactose metabolism | 0.555677769920152 | 0.740903693226869 | 1 | 15 | 15 | 292 | Over | Com\_275\_neg |
| map00350 | Tyrosine metabolism | 0.555677769920152 | 0.740903693226869 | 1 | 15 | 15 | 292 | Over | Com\_673\_pos |
| map00520 | Amino sugar and nucleotide sugar metabolism | 0.555677769920152 | 0.740903693226869 | 1 | 15 | 15 | 292 | Over | Com\_296\_neg |
| map04080 | Neuroactive ligand-receptor interaction | 0.555677769920152 | 0.740903693226869 | 1 | 15 | 15 | 292 | Over | Com\_226\_neg |
| map02010 | ABC transporters | 0.658017358665684 | 0.822521698332105 | 2 | 30 | 15 | 292 | Over | Com\_275\_neg Com\_288\_neg |
| map01100 | Metabolic pathways | 0.767724752948989 | 0.903205591704693 | 12 | 218 | 15 | 292 | Over | Com\_133\_neg Com\_159\_neg Com\_161\_neg Com\_197\_neg Com\_226\_neg Com\_242\_neg Com\_275\_neg Com\_296\_neg Com\_378\_pos Com\_392\_pos Com\_673\_pos Com\_92\_neg |
| map00270 | Cysteine and methionine metabolism | 1 | 1 | 1 | 18 | 15 | 292 | Over | Com\_197\_neg |
