# Supplementary material for: Association analysis of transcriptome and quasi-targeted metabolomics reveals the regulation mechanism underlying broiler muscle tissue development at different levels of dietary guanidinoacetic acid
Source: Front Vet Sci. 2024 Apr 25;11:1384028. doi: 10.3389/fvets.2024.1384028 (PMC11080945; doi:10.3389/fvets.2024.1384028)
Supplement: Supplementary file 2 [file Data_Sheet_1.ZIP › Result-X101SC22030966-Z01-J001-B1-42 (quasi-targeted metabolomics)/5.MetKeggEnrichment/No_GAA.vs.Control/No_GAA.vs.Control_all_KEGG_map/KEGG.map.html]

Pathway Enrichment


  

# The most enriched pathway terms of No\_GAA.vs.Control\_all

Statistic method: hypergeometric test

FDR correction method: Benjamini and Hochberg

| MapID | MapTitle | Pvalue | AdjustedPv | x | y | n | N | EnrichDirect | GeneIDs |
| --- | --- | --- | --- | --- | --- | --- | --- | --- | --- |
| map00071 | Fatty acid degradation | 0.0272560372828696 | 0.13418128688031 | 1 | 2 | 4 | 292 | Over | Com\_263\_neg |
| map00562 | Inositol phosphate metabolism | 0.067090643440155 | 0.13418128688031 | 1 | 5 | 4 | 292 | Over | Com\_371\_pos |
| map00760 | Nicotinate and nicotinamide metabolism | 0.155275445409748 | 0.200811526856102 | 1 | 12 | 4 | 292 | Over | Com\_537\_pos |
| map00310 | Lysine degradation | 0.167342939046752 | 0.200811526856102 | 1 | 13 | 4 | 292 | Over | Com\_263\_neg |
| map00260 | Glycine, serine and threonine metabolism | 0.237055537874832 | 0.237055537874832 | 1 | 19 | 4 | 292 | Over | Com\_319\_pos |
