# Supplementary material for: Association analysis of transcriptome and quasi-targeted metabolomics reveals the regulation mechanism underlying broiler muscle tissue development at different levels of dietary guanidinoacetic acid
Source: Front Vet Sci. 2024 Apr 25;11:1384028. doi: 10.3389/fvets.2024.1384028 (PMC11080945; doi:10.3389/fvets.2024.1384028)
Supplement: Supplementary file 2 [file Data_Sheet_1.ZIP › Result-X101SC22030966-Z01-J001-B1-42 (quasi-targeted metabolomics)/Met_results_dir.html]

Directory Tree


# Directory Tree

../results
  
 ├── 1.MetQuant-QC:代谢物定性定量结果目录
  
 ├── 2.MetAnnotation:代谢物注释结果目录
  
 │ ├── KEGG
  
 │ ├── HMDB
  
 │ ├── Lipidmaps
  
 │
  
 ├── 3.MetDiffScreening:差异代谢物筛选结果目录
  
 │ ├── No\_GAA.vs.Control
  
 │ ├── High\_GAA.vs.Control
  
 │ ├── High\_GAA.vs.No\_GAA
  
 │
  
 ├── 4.MetDiffAnalysis:差异代谢物分析结果目录
  
 │ ├── No\_GAA.vs.Control
  
 │ ├── High\_GAA.vs.Control
  
 │ ├── High\_GAA.vs.No\_GAA
  
 │ ├── Venn\_diff
  
 │ ├── Heatmap\_diff
  
 │
  
 ├── 5.MetKeggEnrichment:差异代谢物Kegg富集分析结果目录
  
 │ ├── No\_GAA.vs.Control
  
 │ ├── High\_GAA.vs.Control
  
 │ ├── High\_GAA.vs.No\_GAA

---

tree v1.5.3 © 1996 - 2009 by Steve Baker and Thomas Moore   
HTML output hacked and copyleft © 1998 by Francesc Rocher   
Charsets / OS/2 support © 2001 by Kyosuke Tokoro
