# Supplementary material for: Association analysis of transcriptome and quasi-targeted metabolomics reveals the regulation mechanism underlying broiler muscle tissue development at different levels of dietary guanidinoacetic acid
Source: Front Vet Sci. 2024 Apr 25;11:1384028. doi: 10.3389/fvets.2024.1384028 (PMC11080945; doi:10.3389/fvets.2024.1384028)
Supplement: Supplementary file 2 [file Data_Sheet_1.ZIP › Result-X101SC22030966-Z01-J001-B1-42 (quasi-targeted metabolomics)/Result_list-Software_readme.pdf]

Result_list-Software_readme

1. **结果目录结构：**

|-- 1.MetQuant-QC 【代谢物定性定量结果目录】

| |-- meta_intensity_all.xls 【所有代谢物定量结果列表】

| |-- sam_qc_infor_all.xls 【样本信息及总 PCA 图中样本编号信息】

| |-- Samples_QC_all-PCA[.3D].{png,pdf} 【QC 及所有代谢样本的总 PCA 图】

| |-- Samples_ QC _all-pcaloading.{png,pdf} 【QC 及所有代谢样本总 PCA 图的载荷图】

| |-- Samples_all-PCA[.3D].{png,pdf} 【所有代谢样本的总 PCA 图】

| |-- Samples_all-pcaloading.{png,pdf} 【所有代谢样本总 PCA 图的载荷图】

| |-- QC-TIC_neg{pos}.png 【QC 及样本的 TIC 图】

| |-- cor_pearson_all.{png,pdf,xls} 【QC 样本相关性分析结果】

|-- 2. MetAnnotation 【代谢物注释结果目录】

| -- KEGG 【基于 KEGG 数据库注释代谢物通路结果】

| |--meta_all_kegg_anno_category.xls 【KEGG 通路注释结果】

| |--meta_all.KEGG.Anno. {png,pdf} 【KEGG 注释结果统计图】

| |---meta_all.KEGG.Anno.xls 【代谢物 KEGG 注释结果列表】

| -- HMDB 【基于 HMDB 数据库注释代谢物分类结果】

| |--meta_all_hmdb_anno_category.xls【HMDB 分类注释结果】

| |--meta_all.HMDB.Anno.{png,pdf} 【HMDB 分类注释结果统计图】

| |-- meta_all.HMDB.Anno.xls 【代谢物 HMDB 注释结果列表】

| -- Lipidmaps 【基于 Lipidmaps 数据库注释代谢物分类结果】

| |--meta_all_lipidmaps_anno_category.xls【Lipidmaps 分类注释结果】

| |--meta_all.Lipidmaps.Anno.{png,pdf} 【Lipidmaps 分类注释结果统计图】

| |-- meta_all.Lipidmaps.Anno.xls 【代谢物 Lipidmaps 注释结果列表】

|-- 3.MetDiffScreening 【差异代谢物筛选结果目录】

| -- *.vs.* 【样本比较对目录】

| |-- *.vs.*_all.xls 【样本比较对定量分析结果】

| |-- *.vs.*_all_Diff.xls 【样本比较对差异代谢物分析】

| |-- *.vs.*_all-PCA[.3D].{png,pdf} 【样本比较对 PCA 分析】

| |-- *.vs.*_all-PCA-pcaloading.{png,pdf} 【样本比较对 PCA loading 分析】

| |-- *.vs.*_all-PLSDA-{score,valid}.{png,pdf}【比较对 PLSDA 分析】

| |-- *.vs.*_all-PLSDA-loading.{png,pdf}【比较对 PLSDA loading 分析】

| |-- *.vs.*_all.xls.volcano.{png,pdf} 【样本比较对火山图分析】

|-- 4.MetDiffAnalysis 【差异代谢物分析结果目录】

| -- Venn_diff 【基于比较多的差异代谢物 venn 图】

| |--Diff_Venn_all.{png,svg}【总差异代谢物 venn 图】

| -- Heatmap_diff 【总差异代谢物热图结果】

| |--Diff_Heatmap_all{_cluster,cluster_detail}.{png,pdf}【总差异代谢物聚类热图】

| |--Diff_Heatmap_all{.detail}.{png,pdf}【总差异代谢物不聚类热图】

| -- *.vs.* 【比较的样本对目录】

| |-- *.vs.*_all.corr.xls 【差异代谢物相关性列表】

| |-- *.vs.*_all_Pvalue.xls 【基于相关性分析的 p 值列表】

| |-- *.vs.*_all_zscore.xls 【差异代谢物 zscore 分析列表】
| |-- *.vs.*_all_zscore.{png,pdf} 【差异代谢物 z-score 图】
| |-- | |*.vs.*_all_cluster_heatmap[,_detail].{png,pdf} 【差异代谢物聚类热图】
| |-- *.vs.*_all_heatmap[._detail].{png,pdf} 【差异代谢物不聚类热图】
| |-- *.vs.*_all_corr.{png,pdf} 【差异代谢物相关性分析图】
| |-- ROC_all 【差异代谢物 ROC 曲线图】
|-- **5.MetKeggEnrichment 【差异代谢物 KEGG 富集分析结果目录】**
| -- *.vs.* 【比较的样品对目录】
| |-- *.vs.*_all.kegg_enrichment.xls【差异代谢物 kegg 富集结果列表】
| |-- *.vs.*_all.KEGG_Enrich.scatterplot.{png,pdf}【kegg 富集气泡图】
| |-- *.vs.*_all_KEGG_map 【kegg 通路图】
|--Met_results_dir.html 【网页版结果文件链接】

2. 分析过程使用的软件版本汇总

分析	内容	软件	版本
**代谢物定性定量**	代谢物定性	Python	Python-3.5.0
	代谢物定量		
**数据质控**	QC样本质控	R	R-3.4.3
	总样品PCA分析		
**代谢物注释**	KEGG\HMDB\LIPIDMAPS	python	Python-2.7.6
		R	R-3.4.3
**差异代谢物筛选**	主成分分析（PCA）	python、R	Python-3.5.0、R-3.4.3
	偏最小二乘法判别分析（PLS-DA）		
	火山图		
**差异代谢物分析**	差异代谢物Venn图分析		
	差异代谢物聚类分析		
	差异代谢物相关性分析		
	Z-score分析		
**KEGG富集分析**	KEGG富集	python	Python-3.5.0
		R	R-3.4.3
**差异代谢物ROC曲线分析**	ROC曲线	R	R-3.4.3
**关联分析**	相关性分析	python	Python-3.5.0
		R	R-3.4.3
	KEGG共有通路筛选	python	Python-3.5.0
		R	R-3.4.3
**备注**	**所有分析内容，数据处理主要用Python，图表绘制用R语言**		
